# A systematic study on the kinetics of H-shift reactions in pristine acyl peroxy radicals†

**DOI:** 10.1039/d3cp01833d

**Published:** 2023-09-11

**Authors:** Prasenjit Seal, Shawon Barua, Siddharth Iyer, Avinash Kumar, Matti Rissanen

**Affiliations:** a Aerosol Physics Laboratory, Physics Unit, Tampere University 33720 Tampere Finland prasenjit.seal@tuni.fi matti.rissanen@tuni.fi; b Department of Chemistry, University of Helsinki P. O. Box 55 FI-00014 Helsinki Finland

## Abstract

A series of acyl peroxy radical H-shifts were systematically studied using computational approaches. Acyl peroxy radicals were categorized into small- (ethanal–pentanal), medium- (hexanal and heptanal) and large-sized (octanal and nonanal) molecules. The H-shifts spanning from 1,4 to 1,9 were inspected for each studied system. For all acyl peroxy radicals, it is the combination of barrier heights and quantum mechanical tunneling that explains the yield of the peracid alkyl radical product. We used the ROHF-ROCCSD(T)-F12a/VDZ-F12//ωB97X-D/aug-cc-pVTZ level of theory to estimate the barrier heights and the subsequent rate coefficients with the exception of the smallest acyl peroxy radical ethanal, for which MN15 density functional was applied. The estimated multiconformer H-shift rate coefficients were found to be in the range of 10^−2^ s^−1^ to 10^−1^ s^−1^ for the fastest H-migrations. The determined rates imply that these H-shift reactions are often competitive with other RO_2_ loss processes and should be considered as a path to functionalization in modelling not only rural but also urban air quality.

## Abstract 2

A systematic study is carried out for the first time on H-shifts in pristine acyl peroxy radicals. The fastest rates were found to be in the range of 10^−2^ to 10^−1^ s^−1^, remaining competitive even under moderately polluted atmospheric conditions.

## Graphical abstract

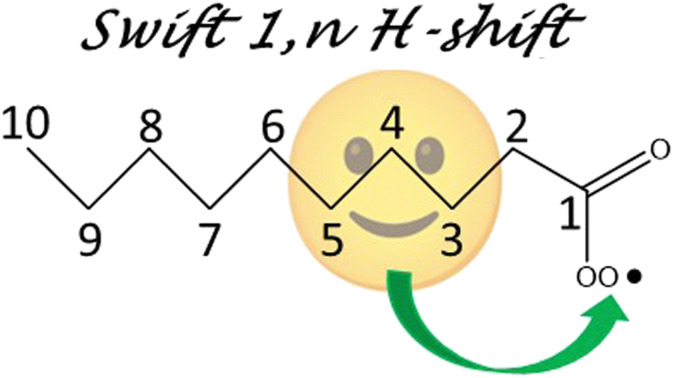

## 1 Introduction

Studies on acyl peroxy radicals or RCH_2_C(=O)OO˙ gained importance due to the fact that they are crucial for the understanding of atmospheric NO_*x*_ distribution.^1^ Acyl peroxy (–AcOO) radicals are produced from aldehydes primarily *via* reactions with OH radicals and by photolysis of ketones and substituted aldehydes.^2,3^ Owing to the low bond strength and being highly labile, the H atom of the –CHO group can easily be abstracted by the OH radical to form an acyl radical. This is followed by either the elimination of CO to produce alkyl radicals with one less carbon atom or the concomitant addition of molecular O_2_, which leads to the formation of the –AcOO radical. These radicals can react with NO_2_ (reversible addition) to form acyl peroxy nitrates (APN), which are key compounds in the long-range transport of NO_*x*_.^1–7^ Acyl peroxy radicals can also react with NO to form acyloxy radicals that have the tendency to fragment instantaneously to lower alkyl radicals by elimination of CO_2_. However, the –AcOO radicals can also undergo isomerization by an intramolecular H-shift reaction from different positions of the carbon chain to produce peracid alkyl radicals, representing a channel that has been frequently neglected. The carbon-centered peracid radicals can then further autoxidize and form highly oxygenated organic molecules (HOMs) that are the condensable gas-phase compounds forming a large fraction of the atmospheric secondary organic aerosol (SOA).^8,9^ These –AcOO isomerization reactions are always in competition with bimolecular RO_2_ reactions, which they need to outcompete to allow for HOM formation. In a clean environment with low NO_*x*_ concentration, isomerization rates are frequently higher than bimolecular losses, whereas under moderately to highly polluted atmospheric conditions with high NO_*x*_ concentrations (*i.e.*, above about 2 × 10^10^ molecules cm^−3^ or 1 ppb), a competitive H-shift rate must be on the order of 0.1 s^−1^ (*i.e.*, RO_2_ loss rate in 1 ppb of NO is around 10^−2^ to 10^−1^ s^−1^ with *k*_RO_2_+NO_: ∼10^−12^ to 10^−11^ cm^3^ molecules^−1^ s^−1^). Hence, H-shift rates of around 10^−2^ s^−1^ and higher are generally fast enough for propagating the autoxidation chain.

Only a handful of previous investigations have reported H-shift reactions in substituted –AcOO radicals of varying chain lengths.^1,4,5^ Knap and Jørgensen^1^ investigated H-shift reactions in substituted –AcOO radicals using high-level quantum chemical methods. The estimated rate coefficients reported for these –AcOO radicals were found to be 2–3 orders of magnitude higher than those of the corresponding aliphatic peroxy radicals. This illustrates the importance of the reactivity of –AcOO radicals to form more stable peracid radicals. Møller *et al.*^10^ also reported higher rate coefficients for multi-substituted acyl peroxy radicals compared to aliphatic peroxy radicals in their work. Vereecken and Nozière,^5^ recently, have reported a series of data sets of H migration rate coefficients in peroxy radicals and constructed a corresponding structure–activity relationship (SAR). For –AcOO radicals, they tentatively summarized the difference in the rates of –AcOO radicals and aliphatic peroxy radicals with a temperature-dependent factor of exp (900 K/*T*) in favor of –AcOO radicals.

Despite previous theoretical interest, no experimental data appear to be available for the H-shift reactions of pristine –AcOO radicals, *i.e.*, species without any other functionalities on the C chain except the terminal acyl peroxy moiety. The fast H-shift rates estimated for the substituted –AcOO radicals raise the question of how much of the reactivity results from the –AcOO group, and how much is offered by the inductively coupled substituent groups. Thereby, in order to fill this gap, we performed a detailed analysis of the H-shift reactions in these pristine radicals to elucidate their potential role in atmospheric oxidation radical chain propagation, and closely related ambient SOA generation.

## 2 Computational methodology

### 2.1 Structure search

For performing a systematic study of the H-shift reactions in –AcOO radicals starting from ethanal up to nonanal, we followed a three-point strategy, *viz.*, conformer sampling along with single-point quantum chemical (QC) calculations, optimization at a moderate density functional theoretical (DFT) level followed by higher-level DFT optimization (labeled high-DFT henceforth), and finally energy-refinement at the coupled-cluster level of theory. Since the species involved are open-chain hydrocarbons connected by multiple single bonds, conformational sampling is essential and was done *via* molecular mechanics using the Merck Molecular Force Field (MMFF) method implemented in the Spartan′20 code.^6,11^ The conformers were generated by varying all torsional angles of each molecular species by 120°. For the large-sized octanal and nonanal –AcOO radicals, we performed a Monte Carlo conformational search whereas for the medium- and small-sized radicals, a SYSTEMATIC search algorithm was carried out in Spartan. We then performed single-point quantum chemical calculations for these Spartan-generated conformers at the B3LYP/6-31+G* level of theory using the Gaussian 16 suite of programs^12^ and used a cut-off of 5.0 kcal mol^−1^ in relative electronic energy with respect to minimum. This was done not only to separate out low-lying energy conformers that will be used for further calculations and energy refinements, but also to enhance the computational feasibility. With the increase in the system size, the number of initial conformers also increases, and consequently also the expense to perform QC calculations on all such conformers. For the transition state (TS) corresponding to each of the H-shifts, we first constrained the H atom at an approximate distance from the relevant C and O atoms and then optimized the structure at the B3LYP/6-31+G* level of theory. The optimized geometry was then used as an input for an unconstrained TS calculation. Once the TS geometry was found, MMFF conformer sampling was carried out using Spartan′20 with the O⋯H and H⋯C bond lengths constrained. It is noteworthy to mention that partial bonds with torsions enabled were added to these two crucial bonds prior to the conformer sampling. The partial bonds resulted in improved MMFF optimization, which in turn gave geometries that are closer to the local energy minima during conformer sampling.^13^

**Fig. 1:**
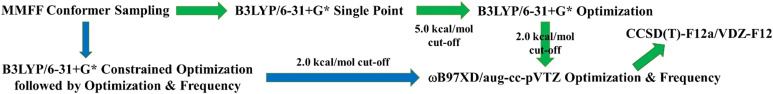
A schematic representation of the strategy that was followed in the structure search. The green arrows are that of the reactants and products, and the blue arrows are of the TS, which then combined with the reactants and products in the final energy refinement step.

After the initial sorting of the conformers, two sets of optimizations were performed, the first of which was done at the B3LYP/6-31+G* (moderate-DFT) level of theory, and structures within 2.0 kcal mol^−1^ of the minimum were then further re-optimized at ωB97X-D/aug-cc-pVTZ (high-DFT) to get the global minimum (GM) geometry.^14–16^ In the case of ethanal-AcOO radicals, although we found the transition state at B3LYP/6-31+G*, reoptimizing the TS at ωB97X-D/aug-cc-pVTZ became difficult and resulted in a completely different geometry other than H-shift. Hence, we used the MN15 density functional instead of ωB97X-D that was successful in yielding an H-shifted TS. Finally, we performed energy refinement of the global minimum structure for all the species at the ROHF-ROCCSD(T)-F12a/VDZ-F12 level of theory employing MOLPRO 2021.2.^17^ This refinement was performed to achieve accurate and reliable energies for the estimation of the H-shift rate coefficients. The strategy is presented schematically in Fig. 1.

### 2.2 Rate coefficient calculations

The use of conventional transition state theory (TST) is by far the most common way to estimate unimolecular rate coefficients. In the present work, we used multiconformer transition state theory (MC-TST), single structure TST (SS-TST), and master equation simulations for estimating H-shift rates in pristine –AcOO radicals. Amongst these, due to the nature of the reactant peroxy radicals with multiple potential reaction pathways and chemically active spatial structures, MC-TST coupled with intrinsic reaction coordinate (IRC) calculations for connecting the correct reacting conformers (*i.e.*, IRC-MC-TST) is primarily focused and discussed in detail in the main text while the others are depicted in the ESI.†.^6,18^ Since it is easier to obtain direct connections (through an IRC calculation) than characterizing all conformers to obtain the global minima on both sides of the TS, there seems little practical value in implementing tunneling using energies of the global minima energies. In the next two sections, we provide the basic TST equations, where eqn (1) includes all distinguishable conformers and their partition functions and eqn (2) includes the contribution from the GM geometry only. Using eqn (1), we obtain IRC-MC-TST and GM-MC-TST rates whereas using eqn (2), we estimate the SS-TST rate coefficients for these reactions.

#### A. MC-TST

In the MC-TST approach, we included all the multiple conformer contributions within 2 kcal mol^−1^ of the global minima at ωB97X-D/aug-cc-pVTZ to determine the overall reaction rate. Using the MC-TST that includes quantum mechanical tunneling,^19^ we arrive at eqn (1)1
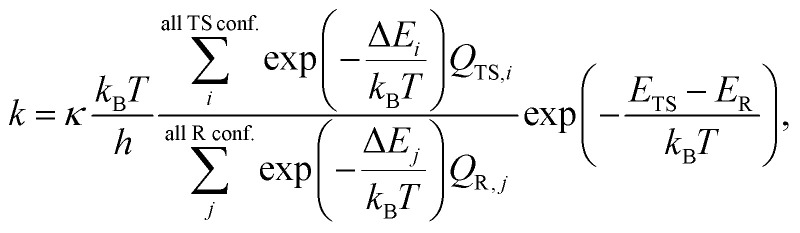
where the summations in both the numerator and the denominator were over all conformers of the TSs and reactants, respectively. In eqn (1), *κ* is the Eckart tunneling coefficient and is calculated manually using a MATLAB code that we provide in the ESI.† We assume that tunneling is the same for all reaction paths. Δ*E*_*i*_ represents the zero-point-corrected energy of transition state conformer *i* relative to the lowest-energy transition state conformer, and *Q*_TS,*i*_ is the total partition function of transition state conformer *i*. Δ*E*_*j*_ and *Q*_R,*j*_ are the corresponding values for the reactant conformer *j*. The energy values, *E*_TS_ and *E*_R_ in the final factor refer to the zero-point-corrected energies of the lowest-energy conformers of the TS and the reactant, respectively.

#### B. Single-structure TST and master equation simulations

For comparison and to see the sensitivity of different methodologies adopted, we also implemented the SS-TST approach and master equation simulations besides the MC-TST. The single-structure transition state theory (SS-TST) treatment, unlike the multiconformer way, considers only the GM geometry that we obtained at the ωB97X-D/aug-cc-pVTZ level of theory and the final energies after CCSD(T) corrections. For the SS-TST, eqn (1) can be rewritten as follows:2
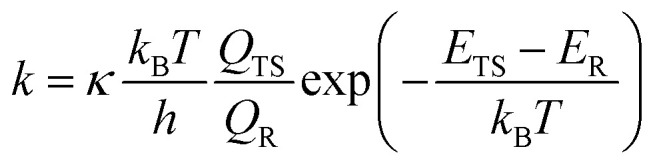
where the corresponding contributions are only from the GM structure. Similar to eqn (1), the Eckart tunneling coefficient in eqn (2) was also calculated manually using a MATLAB code (see the ESI†).

The master equation simulations use the Master Equation Solver for Multi-Energy well Reactions (MESMER) code^20^ to estimate the rate coefficients for these H-shift reactions. MESMER is a single-conformer implementation code. A detailed explanation of how we used MESMER here along with the results obtained from the code is presented in the ESI.†

#### C. Eckart tunneling factor

While estimating *k via* MC-TST treatment, we computed the Eckart tunneling factor, *κ* of eqn (1) with two approaches using our MATLAB code. In the first approach, intrinsic reaction coordinate (IRC) calculations were performed at the corresponding B3LYP/6-31+G* optimized structure of the global minimum TS geometry to obtain the reactant and product wells connecting the global minimum TS.^6^ The IRC-obtained reactants and products were then optimized using the ωB97X-D/aug-cc-pVTZ electronic structure method followed by CCSD(T) energy refinement. The obtained rate coefficients are denoted as *k*_IRC-MC-TST_. For the second approach, we calculated *κ* considering only the global minimum energies for the reactants, TSs, and products ignoring the fact that the GM reactants and products have the global minimum TS in the PES. The rate coefficients obtained thereafter are denoted as *k*_GM-MC-TST_. A detailed explanation of the use of these two MC-TST approaches is given in the next section.

## 3 Results and discussion

**Fig. 2:**
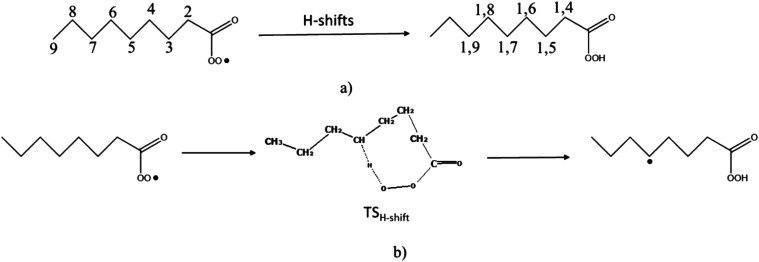
(a) Possible 1,n H-shifts in –AcOO radicals, and (b) a 1,7 H-shift shown as an example for the octanal-AcOO radical.

The H-shifts in the acyl peroxy radicals are schematically presented in Fig. 2. The carbon atom of the functional group –(C=O)OO˙ is considered as 1 and the number increases as we move further from the group, *e.g.*, the α-C atom adjacent to the functional group is labeled as 2, β-C as 3, and so on as shown in the figure. The labeling of the 1,n H-shift shown on the right-hand side of Fig. 2a follows a different convention. The terminal O atom of the peroxy radical is considered as 1 and the C atoms from where the H will be abstracted as n. Hence, an H-shift from C2 (α-C atom) is termed a 1,4 H-shift while the abstraction of H from C3 is called a 1,5 H-shift, and so on.

We categorized the acyl peroxy radicals studied here into small-, medium- and large-sized systems. The small-sized included ethanal-pentanal –AcOO radicals, the medium-sized systems included hexanal and heptanal radicals, whereas the large-sized –AcOO radicals were those obtained from octanal and nonanal.

Table 1 presents the barrier heights (global minimum TS) and energies of the global minimum structure of the product peracid radicals with respect to the corresponding global minimum reactant radicals. Both zero-point corrected (Δ*E*) electronic energies and Gibbs free energy (Δ*G*) values are provided along with the branching ratios of the peracid (–AcOOH) product radicals obtained from MESMER and the Eckart tunneling factor, *κ*_GM_, for all the possible H-shifts of –AcOO radicals starting from the ethanal-AcOO radical to the nonanal-AcOO radical. The cartesian coordinates, absolute values of the energies along with frequencies for each of the systems are provided in the ESI.† This table reveals that for all the H-shifts in –AcOO radicals, it is the combination of the tunneling correction and barrier height that dictates the branching ratios of the products and hence the rate coefficients, where sometimes the tunnelling is large enough to outcompete a smaller barrier with a small tunnelling contribution. A case in point is that observed for the H-shifts in heptanal-AcOO. With an Eckart tunneling of 105 and a barrier of 19.0 kcal mol^−1^, the 1,6 H-shift in heptanal-AcOO yields 55% product acid radical whereas with a lower barrier of 17.4 kcal mol^−1^ and a tunneling of 75, the 1,7 H-shift yields 39% of the product radical. Similarly, for the 1,8 H-shift, with a barrier of 18.7 kcal mol^−1^ and an Eckart tunneling of 86, we can get only 5% of the peracid radical. The Eckart tunneling contributions range from 68 for the 1,8 H-shift of the octanal-AcOO radical up to 2098 in the 1,4 H-shift of the ethanal-AcOO radical. Despite large tunneling contributions, the reactions with high barriers were found to have negligible product yields. In these H-shift reactions, we considered barriers higher than 20 kcal mol^−1^ to be a ‘high barrier’. As an example, for the butanal-AcOO radical, although the tunneling is 372 for the primary 1,6 H-shift, the high barrier of 22.8 kcal mol^−1^ leads to only 9.33% product yield. Another such instance is the 1,8 H-shift of the hexanal-AcOO radical, where the branching ratio of the radical product is only 0.1%, and results from the high barrier of 22.2 kcal mol^−1^, notwithstanding the appreciable tunneling correction of 286. This can also be attributed to the fact that H atoms on primary carbon atoms tend to have higher bond dissociation energies than secondary.

**Table 1:** Computed energies of the global minimum structures of TSs and that of product peracid radicals (Pdt) (in kcal mol^−1^) with respect to the corresponding reactants for different H-shift reactions of –AcOO radical series. Both zero-point corrected electronic energies (Δ*E*) and Gibbs free energies (Δ*G*) are provided. The branching ratio, BR (in %) obtained from MESMER along with the GM Eckart tunneling factor, *κ*_GM_ obtained from the MATLAB code are also presented

Reactant radicals	Parameters	H-shifts											
		1,4		1,5		1,6		1,7		1,8		1,9	
		TS	Pdt	TS	Pdt	TS	Pdt	TS	Pdt	TS	Pdt	TS	Pdt
Ethanal-AcOO	Δ*E*	29.6	2.6	—	—	—	—	—	—	—	—	—	—
	Δ*G*	29.7	2.9										
	BR	—		—		—		—		—		—	
	*κ*_GM_	2098		—		—		—		—		—	
Propanal-AcOO	Δ*E*	26.8	−2.8	24.5	5.2	—	—	—	—	—	—	—	—
	Δ*G*	26.9	−2.8	25.4	4.7								
	BR	3.2		96.8		—		—		—		—	
	*κ*_GM_	136		257		—		—		—		—	
Butanal-AcOO	Δ*E*	26.5	−2.2	21.0	2.4	22.8	4.6	—	—	—	—	—	—
	Δ*G*	26.7	−2.7	21.9	1.8	23.9	3.9						
	BR	0.03		90.64		9.33		—		—		—	
	*κ*_GM_	124		114		372		—		—		—	
Pentanal-AcOO	Δ*E*	26.5	−2.3	20.6	2.7	19.5	1.7	20.7	4.3	—	—	—	—
	Δ*G*	26.6	−2.4	21.6	2.1	20.7	0.8	22.6	3.8				
	BR	0.0		11.0		82.8		6.2		—		—	
	*κ*_GM_	130		95		150		281		—		—	
Hexanal-AcOO	Δ*E*	27.1	−2.1	20.5	2.9	19.1	1.8	18.1	1.4	22.2	4.7	—	—
	Δ*G*	27.8	−1.8	21.4	2.8	20.1	0.5	20.1	1.7	24.2	4.5		
	BR	0.0		5.1		52.7		42.1		0.1		—	
	*κ*_GM_	147		95		112		94		286		—	
Heptanal-AcOO	Δ*E*	27.0	−2.2	20.9	2.8	19.0	1.9	17.4	1.4	18.7	1.5	20.9	4.7
	Δ*G*	27.5	−1.9	22.5	3.0	19.7	2.6	19.7	2.2	21.0	1.0	23.6	5.0
	BR	0.0		0.5		55.3		39.2		4.8		0.2	
	*κ*_GM_	158		102		105		75		86		239	
Octanal-AcOO	Δ*E*	26.9	−2.3	20.5	2.9	19.5	1.5	17.7	1.6	18.4	1.7	19.9	1.9
	Δ*G*	27.6	−2.2	21.4	4.2	21.3	2.7	19.8	2.8	20.7	1.0	22.6	1.8
	BR	0.0		4.9		8.5		76.3		9.7		0.6	
	*κ*_GM_	123		86		129		107		68		98	
Nonanal-AcOO	ΔE	26.9	−2.3	20.5	2.5	19.1	1.0	17.6	1.4	18.1	1.8	20.7	2.3
	ΔG	27.6	−1.8	21.4	3.8	21.2	2.4	20.0	2.8	20.5	2.3	23.9	3.3
	BR	0.0		4.5		10.7		68.7		16.0		0.1	
	*κ*_GM_	134		86		130		107		69		166	

We computed the IRC-MC-TST unimolecular rate coefficients, *k*_IRC-MC-TST_, for all the –AcOO radicals studied, and presented them in Table 2. These values correspond to the current best estimates of –AcOO radical H-shift isomerization rates. We also provide the SAR-derived rate coefficients by Vereecken and Nozière^5^ for typical peroxy radical H-shift schemes for comparison. The rate coefficients obtained from the GM-MC-TST approach, the SS-TST (eqn (2) with *κ*_IRC_), and MESMER simulations are provided in Table S2 of the ESI.† The ratio between the SS-TST and MC-TST rate coefficients, *i.e.*, *k*_SS-TST_ : *k*_IRC-MC-TST_ is found to be 3.5 when averaged over all the H-shift reactions. This implies the fact that ignoring the presence of multiple conformers has the potential to give overestimated rates. However, since the higher energy conformers are similar in geometry to the lowest, this overestimation is within a factor of 5.

**Table 2:** Computed IRC-MC-TST rate coefficients (in s^−1^) and the SAR data obtained from Vereecken and Nozière's work (VN data) for all possible H-shifts in pristine –AcOO radicals

Reactant radicals	Rate coefficients (s^−1^)	H-shifts					
		1,4	1,5	1,6	1,7	1,8	1,9
Ethanal-AcOO	*k*_IRC-MC-TST_	7.3 × 10^−8^					
	*k*_VN data_	4.0 × 10^−10^	—	—	—	—	—
Propanal-AcOO	*k*_IRC-MC-TST_	5.7 × 10^−6^	8.4 × 10^−5^				
	*k*_VN data_	1.2 × 10^−7^	1.6 × 10^−4^	—	—	—	—
Butanal-AcOO	*k*_IRC-MC-TST_	5.2 × 10^−6^	9.3 × 10^−3^	6.7 × 10^−4^			
	*k*_VN data_	1.2 × 10^−7^	1.6 × 10^−2^	8.1 × 10^−5^	—	—	—
Pentanal-AcOO	*k*_IRC-MC-TST_	4.2 × 10^−6^	1.3 × 10^−2^	5.2 × 10^−2^	4.4 × 10^−3^		
	*k*_VN data_	1.2 × 10^−7^	1.6 × 10^−2^	1.8 × 10^−2^	2.1 × 10^−5^	—	—
Hexanal-AcOO	*k*_IRC-MC-TST_	3.4 × 10^−7^	1.9 × 10^−2^	9.7 × 10^−2^	8.8 × 10^−2^	1.8 × 10^−4^	
	*k*_VN data_	1.2 × 10^−7^	1.6 × 10^−2^	1.8 × 10^−2^	1.8 × 10^−3^	1.7 × 10^−6^	—
Heptanal-AcOO	*k*_IRC-MC-TST_	8.9 × 10^−7^	1.0 × 10^−2^	2.4 × 10^−1^	1.5 × 10^−1^	2.0 × 10^−2^	4.2 × 10^−4^
	*k*_VN data_	1.2 × 10^−7^	1.6 × 10^−2^	1.8 × 10^−2^	1.8 × 10^−3^	2.1 × 10^−5^	—
Octanal-AcOO	*k*_IRC-MC-TST_	1.5 × 10^−6^	1.3 × 10^−2^	1.1 × 10^−1^	9.5 × 10^−2^	2.6 × 10^−2^	1.3 × 10^−3^
	*k*_VN data_	1.2 × 10^−7^	1.6 × 10^−2^	1.8 × 10^−2^	1.8 × 10^−3^	2.1 × 10^−5^	—
Nonanal-AcOO	*k*_IRC-MC-TST_	3.3 × 10^−6^	2.4 × 10^−2^	2.1 × 10^−1^	1.8 × 10^−1^	3.6 × 10^−2^	5.2 × 10^−4^
	*k*_VN data_	1.2 × 10^−7^	1.6 × 10^−2^	1.8 × 10^−2^	1.8 × 10^−3^	2.1 × 10^−5^	—

For the MC-TST method, we estimated the unimolecular rate coefficients using the IRC and GM approaches as mentioned in the methodology section. As the IRC-MC-TST approach connects the correct reactant and product wells, it inherently results in a more realistic description of the rate coefficients than the GM-MC-TST approach, which only connects the global minima. Yet here, the high-DFT conformers of reactants, products, and TSs all lie within 2.0 kcal mol^−1^ with respect to their corresponding GM geometries, and thus one can expect that the IRC-obtained structural orientations should not differ much compared to their GM geometries. However, while computing the energies, we observed that although the forward barrier heights between the two approaches didn’t change as such, the reverse barriers, on the other hand, changed considerably. This difference in the barrier heights is attributed to the geometries of the IRC-obtained products that differ from the corresponding GM geometries. The reactants obtained by IRC have similar structural orientations to that of their corresponding GM. The zero-point corrected forward barrier heights (FBH) and reverse barrier heights (RBH) from the two approaches, along with the corresponding Eckart tunneling factors for different H-shift reactions of –AcOO radical series, are presented in Table S3 (ESI†). The difference in FBH between GM and IRC approaches ranges from 0.0 kcal mol^−1^ in the case of the 1,4 H-shift of ethanal-AcOO and the 1,6 H-shift of heptanal-AcOO to 3.7 kcal mol^−1^ for 1,9 H-shift of nonanal-AcOO radicals. In contrast, for the RBH, the difference ranges from 5.2 kcal mol^−1^ in the case of the 1,9 H-shift of heptanal-AcOO to 15.9 kcal mol^−1^ for the 1,4 H-shift of ethanal-AcOO radicals. This reduction in RBH for the IRC approach reduces the Eckart tunneling factor, *κ*_IRC_ considerably compared to the GM-MC-TST (*κ*_GM_), and therefore, the computed *k*_IRC-MC-TST_ value is always lower than *k*_GM-MC-TST_ as seen in Table S2 of the ESI.† In the case of tunneling factor, this appreciable reduction in the *κ*_IRC_ value is mainly observed for the primary H-shifts. The obtained IRC Eckart tunneling factor is 2.6 times lower than the GM Eckart tunneling factor for 1,9 H-shift of heptanal-AcOO while the same is lowered by 4.4 times for 1,7 H-shift of pentanal-AcOO. It is to be noted here that while obtaining the *k*_IRC-MC-TST_ and *k*_GM-MC-TST_ values from eqn (1), only the tunnelling factor differs, whereas all the other terms remain the same. Hence, this lowering of the rate coefficients is directly proportional to the lowering of the Eckart tunneling factor, *κ*_IRC_ with respect to *κ*_GM_.

**Fig. 3:**
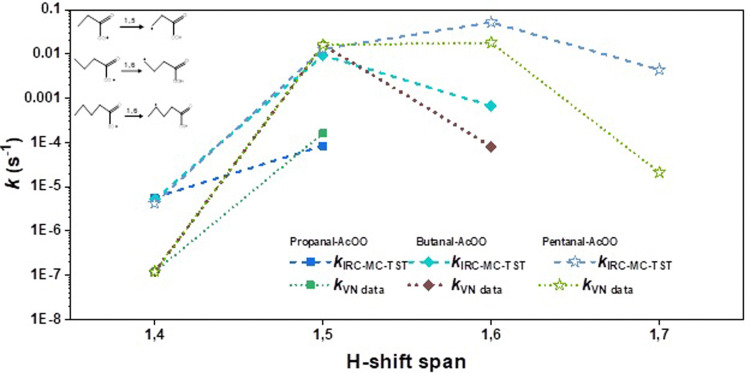
Variation in the rate coefficients for the small-sized acyl peroxy radicals, *i.e.*, propanal, butanal, and pentanal with H-shift span. The dashed lines were obtained from the IRC-MC-TST approach. The values based on the SAR data by Vereecken and Nozière are shown here with dotted lines. For clarity, we also provided the reaction schemes for the 1,5 H-shift in propanal-AcOO, 1,6 H-shifts in butanal-AcOO, and the 1,6 H-shift in pentanal-AcOO.

**Fig. 4:**
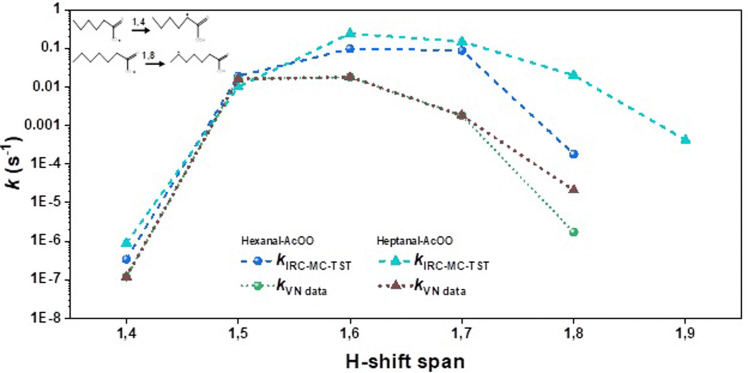
Variation in the rate coefficients for the medium-sized acyl peroxy radicals, *i.e.*, hexanal and heptanal with H-shift span. The dashed lines were obtained from the IRC-MC-TST approach. The SAR data obtained from Vereecken and Nozière's paper are shown here with dotted lines. For clarity, we also provide the reaction schemes for the 1,4 H-shift in hexanal-AcOO, and the 1,8 H-shift in heptanal-AcOO.

**Fig. 5:**
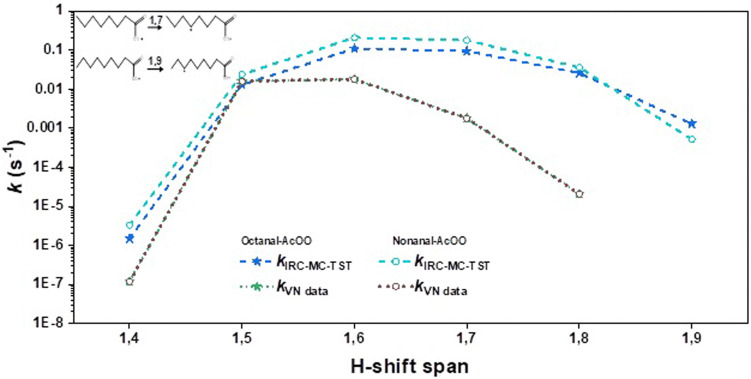
Variation in the rate coefficients for the large-sized acyl peroxy radicals octanal and nonanal with H-shift span. The dashed lines were obtained from the IRC-MC-TST approach. The SAR data obtained from Vereecken and Nozière's paper are shown here with dotted lines. For clarity, we also provide the reaction schemes for the 1,7 H-shift in octanal-AcOO, and the 1,9 H-shift in nonanal-AcOO.

The estimated *k*_IRC-MC-TST_ values presented in Table 2 are shown in Fig. 3–5 for clarity. Since the other approaches discussed in this work were used mainly to see the sensitivity of the methodologies and how the rates derived using them differ, we present graphically the variation of *k* with the H-shift span in Figs. S1-S4 of the ESI.† Comparing the current results with the only previous data, the SAR formulation of Vereecken and Nozière,^5^ we find that our H-shift rates obtained from different sources show a similar trend to the function of the H-shift span yet are higher by one to two orders of magnitude than the SAR data. An exception is provided by the 1,5 H-shifts where the SAR rate coefficients are systematically higher than the *k*_IRC-MC-TST_ rates for all but the hexanal- and nonanal-derived-AcOO radicals (Table 2). The current *k*_IRC-MC-TST_ and *k*_VN data_ agree well for the 1,5 and 1,6 H-shift spans (the VN data based on their predictions for the acyl peroxy on data for 1,5 and 1,6H-shifts) with an average factor of 3 that is well within the indicated uncertainty of the present work and the SAR formulation. Large deviations, however, were observed for the 1,4/1,7/1,8 H-shift spans where the factors are as high as 45, 99, and 1003, respectively. The geometric mean of the *k*_IRC-MC-TST_ : *k*_VN data_ ratio across all the values is about 16. These are tabulated in Table S4 of the ESI.† The deviations observed are likely due to the scarceness of the experimental rate data, and thus on the lack of representative systems to base the SAR on.

Vereecken and Nozière derived an empirical correction factor of exp (900 K/*T*) (*i.e.*, about a factor of 20 at 298 K) in favor of the –AcOO radical H-shift rates over the corresponding aliphatic peroxy radical H-shifts:^5^3
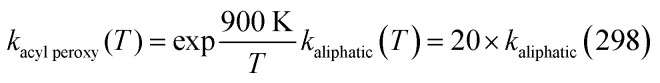
A similar procedure repeated here led to new empirical correction factors of exp (1700 K/*T*) for the 1,4 and 1,7 H-shift spans and exp (2500 K/*T*) for the 1,8 H-shift span, which when applied to *k*_aliphatic_(*T*) of Vereecken and Nozière improves their *k*_acyl peroxy_(*T*) to match with our current best estimates of the H-shift rate coefficients:4
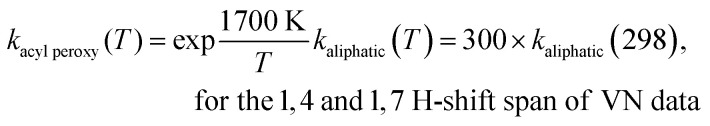
5
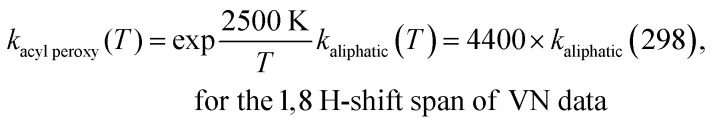
Table S4 and Fig. S5–S7 of the ESI,† presents our *k*_IRC-MC-TST_ and *k*_VN data_ (with the new empirical correction factors) both in a table format and graphically. A close inspection of the results reveals that the large deviations that were observed between the IRC-MC-TST and VN rates for 1,4/1,7/1,8 H-shift spans in Fig. 3–5 reduced appreciably in Fig. S5–S7 of the ESI,† with the introduction of new improved empirical correction factors to the VN data. A comparison between the factors, Avg_1,*n*_, as given in Table S4 of the ESI† also justifies the above observations, where the Avg_1,*n*_ value reduced from 45, 99, and 1003 to 3, 7, and 5 (presented in parentheses) for 1,4/1,7/1,8 H-shift spans, respectively. For ethanal-AcOO radical, since there is only one available 1,4 H-shift rate coefficient, we have not included the value in Fig. 3. It has been observed that with the –AcOO radical H-shifts, 1,6 is easily the fastest, yet with increasing carbon chain length such as for octanal- and nonanal-AcOO, the 1,7 and even the 1,8 H-shift can be faster.

A close inspection of *k*_IRC-MC-TST_ in Table 2 deciphers the role of inductive effects influencing the rate coefficients of these H-shift reactions. We observed this effect between the primary 1,n H-shift in a particular –AcOO radical and a corresponding secondary 1,n H-shifts in the next two higher –AcOO radical series when –CH_3_ and –CH_2_CH_3_ groups, respectively, were added to that radical. For instance, the secondary 1,6 H-shift *k*_IRC-MC-TST_ in pentanal-AcOO radical (*i.e.*, the –CH_3_ group added to the butanal-AcOO radical), is 78 times higher than the primary 1,6 H-shift of butanal-AcOO. In the case of the hexanal-AcOO radical (when a –CH_2_CH_3_ group is added to the butanal-AcOO radical), the 1,6 H-shift *k*_IRC-MC-TST_ is 145 times higher than in butanal-AcOO. When comparing the 1,9 H-shifts for the three highest –AcOO radicals (*i.e.*, heptanal-, octanal- and nonanal-), in heptanal-AcOO the 1,9 H-shift corresponds to a primary H-shift while in octanal- and nonanal-AcOO radicals, they correspond to secondary H-shifts. In the octanal-AcOO radical (*i.e.*, adding a –CH_3_ group to the heptanal-AcOO radical), there is a 3-fold increase in *k*_IRC-MC-TST_ whereas the 1,9 H-shift rate in the nonanal-AcOO radical (adding –CH_2_CH_3_ group to heptanal-AcOO) increases *k*_IRC-MC-TST_ only by 1.2-fold. The magnitude of the inductive effect appears substantial also for the 1,8 H-shifts in octanal-, heptanal, and hexanal-AcOO radicals. The 1,8 H-shift in heptanal-AcOO radical is 111 times faster than that of hexanal-AcOO radical and the 1,8 H-shift for octanal-AcOO radical is yet a factor of 144 higher. A detailed description of the H-shift reactions for the –AcOO radicals is provided in Table S5 (ESI†) along with the *k*^secondary^_IRC-MC-TST_/*k*^primary^_IRC-MC-TST_ ratio.

## 4 Conclusions

Herein, we performed a systematic study of the rate coefficients of H-shifts in pristine –AcOO radicals, starting from ethanal up to nonanal. Our results indicate that Eckart tunneling plays an important role in these reactions, as expected for reactions involving migration of the light H-atom. For all –AcOO radicals, it is the combination of barrier heights and tunneling contributions that affect the determined H-shift rate coefficients. For 1,5 and 1,6 H-shifts, there are no statistically significant differences found between the current *k*_IRC-MC-TST_ values and the previous Vereecken and Nozière SAR predictions. However, the differences observed in the rate coefficients for 1,4, 1,7 and 1,8 H-shift spans are likely attributable to the scarceness of the experimental data and the lack of representative systems to base the SAR on. Adopting new empirical correction factors of exp (1700 K/*T*) for 1,4 and 1,7 H-shifts and exp (2500 K/*T*) for 1,8 H-shifts to the SAR data, our *k*_IRC-MC-TST_ and SAR data agree well within a factor of 5. The fastest H-shift isomerization rates were found to range from 10^−2^ to 10^−1^ s^−1^ with the ROHF-ROCCSD(T)-F12a/VDZ-F12//ωB97X-D/aug-cc-pVTZ methodology, remaining competitive even under moderately polluted atmospheric conditions, and hence of high relevance in the context of atmospheric chemistry.

## Conflicts of interest

There are no conflicts to declare.

## Supplementary Material

CP-025-D3CP01833D-s001
