# Supplementary material for: A systematic study on the kinetics of H-shift reactions in pristine acyl peroxy radicals
Source: Phys Chem Chem Phys. 2023 Sep 11;25(41):28205–12. doi: 10.1039/d3cp01833d (PMC10599409; doi:10.1039/d3cp01833d)
Supplement: CP-025-D3CP01833D-s001 [file CP-025-D3CP01833D-s001.pdf]

Electronic Supplementary Material (ESI) for Physical Chemistry Chemical Physics.
This journal is © the Owner Societies 2023

Electronic Supplementary Information (ESI)

A systematic study on the kinetics of H-shift reactions in pristine acyl peroxy radicals

Prasenjit Seal,*[a] Shawon Barua,[a] Siddharth Iyer,[a] Avinash Kumar[a] and Matti Rissanen*[ab]

[a] *Aerosol Physics Laboratory, Physics Unit, Tampere University, 33720 Tampere, Finland. E-mail: prasenjit.seal@tuni.fi, matti.rissanen@tuni.fi*

[b] *Department of Chemistry, University of Helsinki, P.O. Box 55, FI-00014, Helsinki, Finland*

Contents

1	Master equation simulation (MESMER) methodology and results	S-3
2	Computed rate coefficients (in s^{-1}) obtained from MESMER, SS-TST and MC-TST (both IRC and GM approaches) rate equations and the SAR data obtained from Vereecken and Nozière's work (VN data) for all possible H-shifts in pristine –AcOO radicals.	S-5
3	Zero-point corrected forward and reverse barrier heights for different H-shift reactions of –AcOO radical series along with the Eckart tunneling factor, κ computed using IRC and GM approaches following MC-TST treatment	S-7
4	Variation in the GM-MC-TST, IRC-MC-TST rate coefficients and SAR data for pentanal – octanal acyl peroxy radicals with H-shift span	S-9
5	Variation in the rate coefficients for all the acyl peroxy radicals with H-shift span. The solid lines were obtained from the MESMER simulations while the dashed ones were obtained from the TST equations. The values based on the SAR data by Vereecken and Nozière are shown with dotted lines. For clarity, we also provided reaction schemes for some of the H-shifts in these acyl peroxy radicals.	S-10
6	Ratio of the current best estimate $k_{\text{IRC-MC-TST}}$ and SAR data of Vereecken and Nozière with and without the new improved empirical correction factors for 1,4/1,7/1,8 H-shift spans	S-12
7	Variation in $k_{\text{IRC-MC-TST}}$ and $k_{\text{VN data}}$ for all the acyl peroxy radicals with H-shift span. The dashed lines were obtained from the IRC-MC-TST approach while the SAR data obtained from Vereecken and Nozière with new empirical factors are shown here with dotted lines.	S-14
8	Qualitative inspection of the influence of inductive effects in the determined H-shift rates investigated for a series of primary 1,n- H-shifts in comparison to the corresponding 1,n- secondary H-shifts with methyl and ethyl substitutions.	S-16
9	Coupled cluster + zero-point correction energies (in a.u.), and frequencies (in cm^{-1}) obtained from ωB97X-D/aug-cc-pVTZ of the global minimum geometry of each of the species involved	S-17
10	Cartesian coordinates of the global minimum geometry of each of the species involved	S-49
11	Matlab Code for calculating Eckart Tunneling Coefficient	S-84
12	MESMER Input files for all the possible H-shifts in pristine acyl peroxy radicals	S-88

1. Master equation simulation (MESMER) methodology and results

One of the ways that can be used to estimate rate coefficients is the master equation simulations as were performed here with MESMER 6.0 code on the stationary points along the potential energy surface (PES) [*J. Phys. Chem. A*, 2012, **116**, 9545–9560]. Although MESMER is designed for pressure-dependent, multi-well reaction schemes, yet one can use the code to perform a single-well rate coefficient estimation as well. Transition states were treated using Rice-Ramsperger-Kassel-Marcus (RRKM) theory to calculate the micro-canonical rate coefficients and to determine the branching ratios of peracid radical formations via unimolecular isomerization. It is noteworthy to mention that in the present work, we considered the acyl peroxy radicals to be thermalized. Eckart tunneling was used in our calculations as tunneling has a profound impact on H-shift reaction rate coefficients. Energy transfer occurred *via* collisions between modeled species and N_2 bath gas. Such collisions were treated using Lennard-Jones (L-J) potential model and the L-J parameters (σ, ϵ) were used to account for the associated collisional frequency. MESMER utilizes the exponential down (ΔE_{down}) model as given in equation 1 for simulating the collisional energy transfer.

$$\Delta E_{down} = \Delta E_{down,\,ref}\left(\frac{T}{T_{ref}}\right)^{n} \qquad (1)$$

In equation 1, ΔE_{down} is the average downward energy transferred per collision, $\Delta E_{down,\,ref}$ is the value of ΔE_{down} at T_{ref} (=298 K) and n is an independent parameter associated with the temperature dependence of ΔE_{down}.The Lennard–Jones parameters were used for the reactant – AcOO radicals since they are the actual intermediates in the aldehyde autoxidation process. We used the L-J parameters [*J. Chem. Phys.*, 1983, **78**, 6709-6717] of the nearest alkanes for each of the –AcOO radicals and the corresponding values were tabulated in Table S1.

Table S1 The Lennard-Jones parameters used in the MESMER calculation

Reactant radicals	σ (Å)	ϵ (K)
Ethanal–AcOO	5.4	307

Propanal–AcOO	5.9	327
Butanal–AcOO	6.3	343
Pentanal–AcOO	6.7	351
Hexanal–AcOO	7.0	359
Heptanal–AcOO	7.3	362
Octanal–AcOO	7.7	363
Nonanal–AcOO	8.0	362

For N_2 bath gas, the MESMER recommended values for ΔE_{down} are between 175 and 275 cm^{-1}. We used a ΔE_{down} value of 225 cm^{-1} in our calculations. In addition, a grain size of 100 and a value of 25 k_BT for the energy spanned by the grains were used. All the MESMER input files for estimating the rate coefficients of these H-shift reactions are provided from pages S-88 upto S-249.

Hindered Rotor (HR) effect

We did not apply hindered rotation corrections (HR) in MESMER, as it has been suggested to have only a minor influence when used in combination with SS-TST [*J. Phys. Chem. A*, 2016, **120**, 10072−10087]. Nevertheless, we did perform a test computation with the 1,6 H-shift of pentanal-AcOO radical to illustrate the magnitude of this HR effect as given below. The results obtained with the HR reduces the MESMER derived rate coefficient from 1.0 s^{-1} to 0.45 s^{-1}, and thus decreases the difference between the other methodologies.

Reaction	$k_{MESMER}^{no\,HR}$	k_{MESMER}^{HR}
Pentanal-AcylOO-rad→ Pentanal-16-rad	1.00376	0.453425

2. **Table S2** Computed rate coefficients (in s^{-1}) obtained from MESMER, SS-TST and MC-TST (both IRC and GM approaches) rate equations and the SAR data obtained from Vereecken and Nozière's work (VN data) for all possible H-shifts in pristine –AcOO radicals.

Reactant radicals	Rate coefficients (s^{-1})	H-shifts					
		1,4	1,5	1,6	1,7	1,8	1,9
Ethanal-AcOO	k_{MESMER}	3.9×10^{-6}	–	–	–	–	–
	$k_{SS\text{-}TST}$	1.1×10^{-7}					
	$k_{GM\text{-}MC\text{-}TST}$	1.3×10^{-6}					
	$k_{IRC\text{-}MC\text{-}TST}$	7.3×10^{-8}					
	$k_{VN\ data}$	4.0×10^{-10}					
Propanal-AcOO	k_{MESMER}	2.1×10^{-5}	6.3×10^{-4}	–	–	–	–
	$k_{SS\text{-}TST}$	1.1×10^{-5}	1.6×10^{-4}				
	$k_{GM\text{-}MC\text{-}TST}$	7.8×10^{-6}	2.3×10^{-4}				
	$k_{IRC\text{-}MC\text{-}TST}$	5.7×10^{-6}	8.4×10^{-5}				
	$k_{VN\ data}$	1.2×10^{-7}	1.6×10^{-4}				
Butanal-AcOO	k_{MESMER}	2.9×10^{-5}	9.5×10^{-2}	9.7×10^{-3}	–	–	–
	$k_{SS\text{-}TST}$	1.7×10^{-5}	3.0×10^{-2}	2.1×10^{-3}			
	$k_{GM\text{-}MC\text{-}TST}$	7.0×10^{-6}	1.9×10^{-2}	1.9×10^{-3}			
	$k_{IRC\text{-}MC\text{-}TST}$	5.2×10^{-6}	9.3×10^{-3}	6.7×10^{-4}			
	$k_{VN\ data}$	1.2×10^{-7}	1.6×10^{-2}	8.1×10^{-5}			
Pentanal-AcOO	k_{MESMER}	3.5×10^{-5}	1.3×10^{-1}	1.00	7.5×10^{-2}	–	–
	$k_{SS\text{-}TST}$	1.7×10^{-5}	5.0×10^{-2}	2.3×10^{-1}	1.1×10^{-2}		
	$k_{GM\text{-}MC\text{-}TST}$	5.7×10^{-6}	2.5×10^{-2}	1.5×10^{-1}	1.9×10^{-2}		
	$k_{IRC\text{-}MC\text{-}TST}$	4.2×10^{-6}	1.3×10^{-2}	5.2×10^{-2}	4.4×10^{-3}		
	$k_{VN\ data}$	1.2×10^{-7}	1.6×10^{-2}	1.8×10^{-2}	2.1×10^{-5}		
Hexanal-AcOO	k_{MESMER}	5.2×10^{-6}	1.9×10^{-1}	2.03	1.62	4.7×10^{-3}	–
	$k_{SS\text{-}TST}$	2.4×10^{-6}	9.5×10^{-2}	5.1×10^{-1}	3.9×10^{-1}	7.4×10^{-4}	
	$k_{GM\text{-}MC\text{-}TST}$	5.0×10^{-7}	3.9×10^{-2}	2.6×10^{-1}	2.5×10^{-1}	7.5×10^{-4}	
	$k_{IRC\text{-}MC\text{-}TST}$	3.4×10^{-7}	1.9×10^{-2}	9.7×10^{-2}	8.8×10^{-2}	1.8×10^{-4}	
	$k_{VN\ data}$	1.2×10^{-7}	1.6×10^{-2}	1.8×10^{-2}	1.8×10^{-3}	1.7×10^{-6}	
Heptanal-AcOO	k_{MESMER}	9.1×10^{-6}	2.9×10^{-2}	3.41	2.41	3.0×10^{-1}	1.1×10^{-2}
	$k_{SS\text{-}TST}$	4.8×10^{-6}	1.0×10^{-2}	1.00	6.5×10^{-1}	7.8×10^{-2}	2.5×10^{-3}
	$k_{GM\text{-}MC\text{-}TST}$	1.3×10^{-6}	2.0×10^{-2}	5.9×10^{-1}	3.9×10^{-1}	5.9×10^{-2}	1.1×10^{-3}
	$k_{IRC\text{-}MC\text{-}TST}$	8.9×10^{-7}	1.0×10^{-2}	2.4×10^{-1}	1.5×10^{-1}	2.0×10^{-2}	4.2×10^{-4}
	$k_{VN\ data}$	1.2×10^{-7}	1.6×10^{-2}	1.8×10^{-2}	1.8×10^{-3}	2.1×10^{-5}	–
Octanal-AcOO	k_{MESMER}	6.3×10^{-6}	1.8×10^{-1}	3.1×10^{-1}	2.75	3.5×10^{-1}	2.2×10^{-2}
	$k_{SS\text{-}TST}$	3.8×10^{-6}	6.2×10^{-2}	9.1×10^{-2}	5.6×10^{-1}	9.0×10^{-2}	5.1×10^{-3}
	$k_{GM\text{-}MC\text{-}TST}$	2.1×10^{-6}	2.5×10^{-2}	2.6×10^{-1}	3.0×10^{-1}	7.4×10^{-2}	3.8×10^{-3}

	$k_{IRC\text{-}MC\text{-}TST}$	1.5×10^{-6}	1.3×10^{-2}	1.1×10^{-1}	9.5×10^{-2}	2.6×10^{-2}	1.3×10^{-3}
	$k_{VN\ data}$	1.2×10^{-7}	1.6×10^{-2}	1.8×10^{-2}	1.8×10^{-3}	2.1×10^{-5}	–
Nonanal-AcOO	k_{MESMER}	5.8×10^{-6}	1.4×10^{-1}	3.4×10^{-1}	2.19	5.1×10^{-1}	4.1×10^{-3}
	$k_{SS\text{-}TST}$	3.1×10^{-6}	5.3×10^{-2}	1.2×10^{-1}	4.9×10^{-1}	1.5×10^{-1}	7.6×10^{-4}
	$k_{GM\text{-}MC\text{-}TST}$	4.7×10^{-6}	4.6×10^{-2}	5.0×10^{-1}	5.5×10^{-1}	1.0×10^{-1}	2.1×10^{-3}
	$k_{IRC\text{-}MC\text{-}TST}$	3.3×10^{-6}	2.4×10^{-2}	2.1×10^{-1}	1.8×10^{-1}	3.6×10^{-2}	5.2×10^{-4}
	$k_{VN\ data}$	1.2×10^{-7}	1.6×10^{-2}	1.8×10^{-2}	1.8×10^{-3}	2.1×10^{-5}	–

3. **Table S3** The zero-point corrected forward barrier height (FBH) and reverse barrier height (RBH) for different H-shift reactions of –AcOO radical series along with the Eckart tunneling factor, κ computed using intrinsic coordinate (IRC) and global minimum (GM) approaches following multiconfromer transition state theory (MC-TST) treatment.

Reactant radicals	Approach	H-shifts											
		1,4		1,5		1,6		1,7		1,8		1,9	
		FBH	RBH	FBH	RBH	FBH	RBH	FBH	RBH	FBH	RBH	FBH	RBH
Ethanal–AcOO	GM	29.6	27.0										
	IRC	29.6	11.1										
	κ_{IRC}	115.3											
	κ_{GM}	2097.9											
Propanal–AcOO	GM	26.8	29.6	24.5	19.3								
	IRC	25.5	23.6	23.3	12.6								
	κ_{IRC}	99.2		93.6									
	κ_{GM}	136.1		257.0									
Butanal–AcOO	GM	26.5	28.7	21.0	18.6	22.8	18.2						
	IRC	25.6	22.8	19.8	12.2	22.5	12.3						
	κ_{IRC}	91.5		55.4		131.6							
	κ_{GM}	123.9		113.5		372.2							
Pentanal–AcOO	GM	26.5	28.8	20.6	17.9	19.5	17.8	20.7	16.4				
	IRC	25.6	22.9	19.5	11.7	19.3	10.0	19.7	9.0				
	κ_{IRC}	95.4		47.6		51.2		63.4					
	κ_{GM}	129.5		94.7		149.6		280.5					
Hexanal–AcOO	GM	27.1	29.2	20.5	17.6	19.1	17.3	18.1	16.7	22.2	17.5		
	IRC	25.5	22.5	18.9	11.3	17.7	9.7	15.9	8.8	21.3	9.8		
	κ_{IRC}	99.7		45.7		41.6		33.6		68.6			
	κ_{GM}	147.0		94.9		111.7		94.4		285.9			
Heptanal–AcOO	GM	27.0	29.2	20.9	18.1	19.0	17.1	17.4	16.0	18.7	17.2	20.9	16.2
	IRC	25.4	22.4	18.7	11.9	19.0	10.0	15.4	8.4	17.3	8.5	18.0	11.0
	κ_{IRC}	105.2		50.0		42.2		28.8		28.6		90.2	
	κ_{GM}	157.5		102.0		104.5		75.3		85.8		238.9	
Octanal–AcOO	GM	26.9	29.2	20.5	17.6	19.5	18.0	17.7	16.1	18.4	16.7	19.9	18.0
	IRC	26.0	22.8	19.5	11.5	18.2	11.2	16.1	8.3	16.8	8.0	18.9	9.1
	κ_{IRC}	89.2		44.6		55.9		34.4		23.6		32.9	

	κ_{GM}	122.9		85.7		129.0		107.1		68.1		98.4	
Nonanal–AcOO	GM	26.9	29.2	20.5	18.0	19.1	18.1	17.6	16.2	18.1	16.3	20.7	18.4
	IRC	25.9	22.8	19.4	11.6	18.0	11.0	16.2	8.3	16.6	8.0	17.0	9.1
	κ_{IRC}	95.6		43.5		54.9		34.4		24.8		42.0	
	κ_{GM}	133.7		85.5		130.2		107.1		69.3		166.2	

4. **Variation in the GM-MC-TST, IRC-MC-TST rate coefficients and SAR data for pentanal – octanal acyl peroxy radicals with H-shift span**

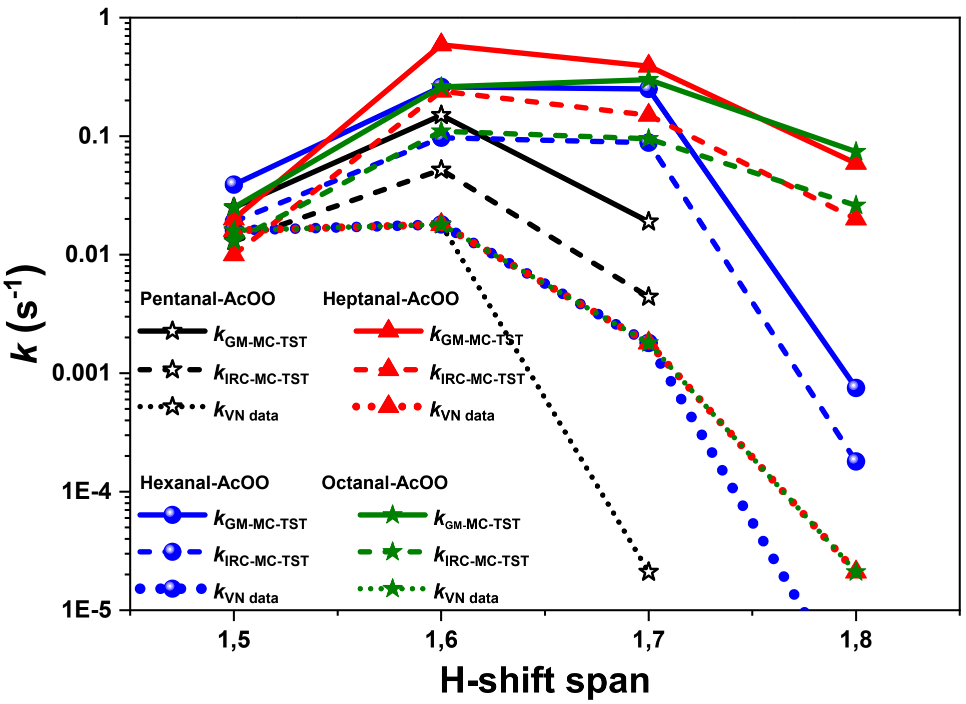

Fig. S1 Variation in the rate coefficients for pentanal – octanal acyl peroxy radicals with H-shift span. The solid (GM-MC-TST) and dashed lines (IRC-MC-TST) were obtained from the MC-TST equation 2. The values based on the SAR data by Vereecken and Nozière are presented in dotted lines.

5. **Variation in the rate coefficients for all the acyl peroxy radicals with H-shift span. The solid lines were obtained from the MESMER simulations while the dashed ones were obtained from the TST equations. The values based on the SAR data by Vereecken and Nozière are shown with dotted lines. For clarity, we also provided reaction schemes for some of the H-shifts in these acyl peroxy radicals.**

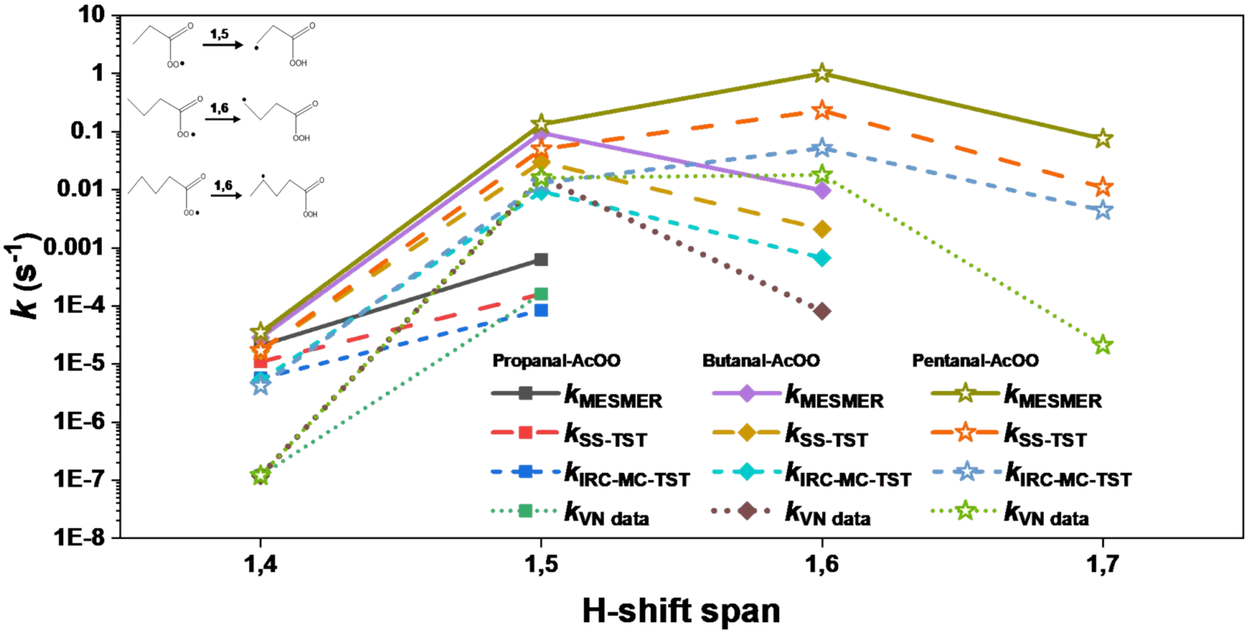

Fig. S2 Variation in the rate coefficients for the small-sized acyl peroxy radicals, *i.e.*, propanal, butanal, and pentanal with H-shift span. For clarity, we also provided the reaction schemes for 1,5 H-shift in propanal-AcOO, 1,6 H-shifts in butanal-AcOO, and 1,6 H-shift in pentanal-AcOO.

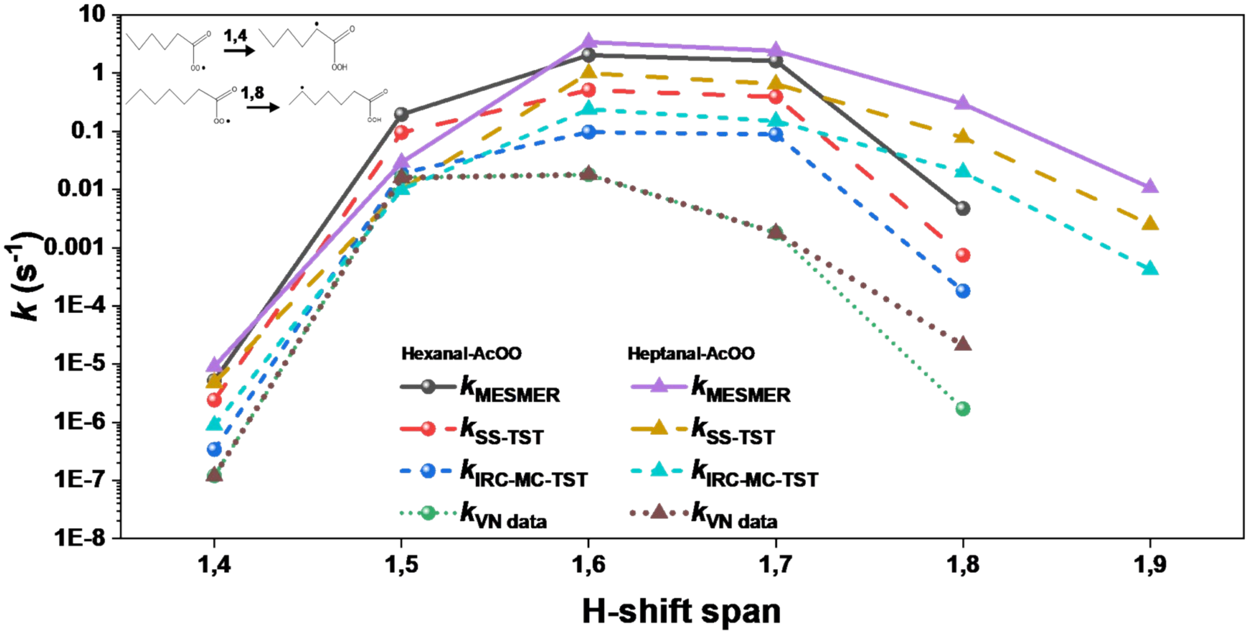

Fig. S3 Variation in the rate coefficients for the medium-sized acyl peroxy radicals, *i.e.*, hexanal and heptanal with H-shift span. For clarity, we also provide the reaction schemes for 1,4 H-shift in hexanal-AcOO, and 1,8 H-shift in heptanal-AcOO.

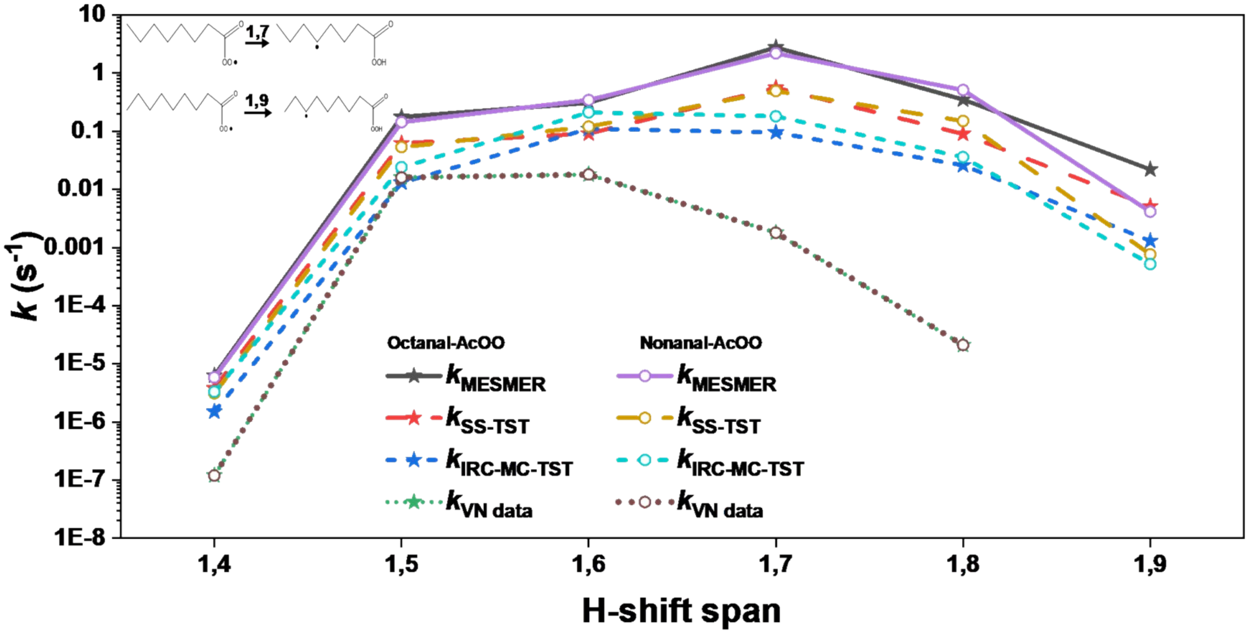

Fig. S4 Variation in the rate coefficients for the large sized acyl peroxy radicals octanal and nonanal with H-shift span. For clarity, we also provide the reaction schemes for 1,7 H-shift in octanal-AcOO, and 1,9 H-shift in nonanal-AcOO.

6. **Table S4** Ratio of the current best estimate $k_{IRC\text{-}MC\text{-}TST}$ and SAR data of Vereecken and Nozière with and without the new improved empirical correction factors for 1,4/1,7/1,8 H-shift spans. In the parentheses, are the factors obtained with the new empirical corrections for 1,4/1,7/1,8 H-shift spans.

**Reactant radicals**	**H-shifts**	$\boldsymbol{k_{IRC\text{-}MC\text{-}TST}}$	$\boldsymbol{k_{VN\ data}}$*	$\boldsymbol{k_{VN\ data\_new\ ef}}$†	$\boldsymbol{k_{IRC\text{-}MC\text{-}TST}}$: $\boldsymbol{k_{VN\ data}}$	$\boldsymbol{k_{IRC\text{-}MC\text{-}TST}}$: $\boldsymbol{k_{VN\ data\_new\ ef}}$	$\boldsymbol{Avg_{1,4}}$	$\boldsymbol{Avg_{1,5}}$	$\boldsymbol{Avg_{1,6}}$	$\boldsymbol{Avg_{1,7}}$	$\boldsymbol{Avg_{1,8}}$
Ethanal-AcOO	1,4	7.3×10^{-8}	4.0×10^{-10}	5.9×10^{-9}	182.5	12.3	44.8 (3.0)	0.9	7.9	98.9 (6.6)	1002.7 (4.6)
Propanal-AcOO	1,4	5.7×10^{-6}	1.2×10^{-7}	1.8×10^{-6}	47.5	3.2					
	1,5	8.4×10^{-5}	1.6×10^{-4}	1.6×10^{-4}	0.5	0.5					
Butanal-AcOO	1,4	5.2×10^{-6}	1.2×10^{-7}	1.8×10^{-6}	43.3	3.0					
	1,5	9.3×10^{-3}	1.6×10^{-2}	1.6×10^{-2}	0.6	0.6					
	1,6	6.7×10^{-4}	8.1×10^{-5}	8.1×10^{-5}	8.3	8.3					
Pentanal-AcOO	1,4	4.2×10^{-6}	1.2×10^{-7}	1.8×10^{-6}	35.0	2.4					
	1,5	1.3×10^{-2}	1.6×10^{-2}	1.6×10^{-2}	0.8	0.8					
	1,6	5.2×10^{-2}	1.8×10^{-2}	1.8×10^{-2}	2.9	2.9					
	1,7	4.4×10^{-3}	2.1×10^{-5}	3.1×10^{-4}	209.5	14.2					
Hexanal-AcOO	1,4	3.4×10^{-7}	1.2×10^{-7}	1.8×10^{-6}	2.8	0.2					
	1,5	1.9×10^{-2}	1.6×10^{-2}	1.6×10^{-2}	1.2	1.2					
	1,6	9.7×10^{-2}	1.8×10^{-2}	1.8×10^{-2}	5.4	5.5					
	1,7	8.8×10^{-2}	1.8×10^{-3}	2.7×10^{-2}	48.9	3.2					
	1,8	1.8×10^{-4}	1.7×10^{-6}	3.8×10^{-4}	105.9	0.5					
Heptanal-AcOO	1,4	8.9×10^{-7}	1.2×10^{-7}	1.8×10^{-6}	7.4	0.5					
	1,5	1.0×10^{-2}	1.6×10^{-2}	1.6×10^{-2}	0.6	0.6					
	1,6	2.4×10^{-1}	1.8×10^{-2}	1.8×10^{-2}	13.3	13.6					
	1,7	1.5×10^{-1}	1.8×10^{-3}	2.7×10^{-2}	83.3	5.5					

	1,8	2.0×10^{-2}	2.1×10^{-5}	4.5×10^{-3}	952.4	4.4
Octanal-AcOO	1,4	1.5×10^{-6}	1.2×10^{-7}	1.8×10^{-6}	12.5	0.9
	1,5	1.3×10^{-2}	1.6×10^{-2}	1.6×10^{-2}	0.8	0.8
	1,6	1.1×10^{-1}	1.8×10^{-2}	1.8×10^{-2}	6.1	6.2
	1,7	9.5×10^{-2}	1.8×10^{-3}	2.7×10^{-2}	52.8	3.5
	1,8	2.6×10^{-2}	2.1×10^{-5}	4.5×10^{-3}	1238.1	5.7
Nonanal-AcOO	1,4	3.3×10^{-6}	1.2×10^{-7}	1.8×10^{-6}	27.5	1.9
	1,5	2.4×10^{-2}	1.6×10^{-2}	1.6×10^{-2}	1.5	1.5
	1,6	2.1×10^{-1}	1.8×10^{-2}	1.8×10^{-2}	11.7	11.9
	1,7	1.8×10^{-1}	1.8×10^{-3}	2.7×10^{-2}	100.0	6.6
	1,8	3.6×10^{-2}	2.1×10^{-5}	4.5×10^{-3}	1714.3	7.9

*obtained from L. Vereecken and B. Nozière, *Atmos. Chem. Phys.*, 2020, **20**, 7429–7458.

†obtained from L. Vereecken and B. Nozière, *Atmos. Chem. Phys.*, 2020, **20**, 7429–7458 using the following modified equations

$k_{acyl\,peroxy}(T) = exp\frac{1700K}{T}k_{aliphatic}(T) = 300 * k_{aliphatic}(298)$, for 1,4 and 1,7 H-shift span

$k_{acyl\,peroxy}(T) = exp\frac{2500K}{T}k_{aliphatic}(T) = 4400 * k_{aliphatic}(298)$ for 1,8 H-shift span

7. Variation in $k_{IRC-MC-TST}$ and $k_{VN\ data}$ for all the acyl peroxy radicals with H-shift span. The dashed lines were obtained from the IRC-MC-TST approach while the SAR data obtained from Vereecken and Nozière with new empirical factors are shown here with dotted lines.

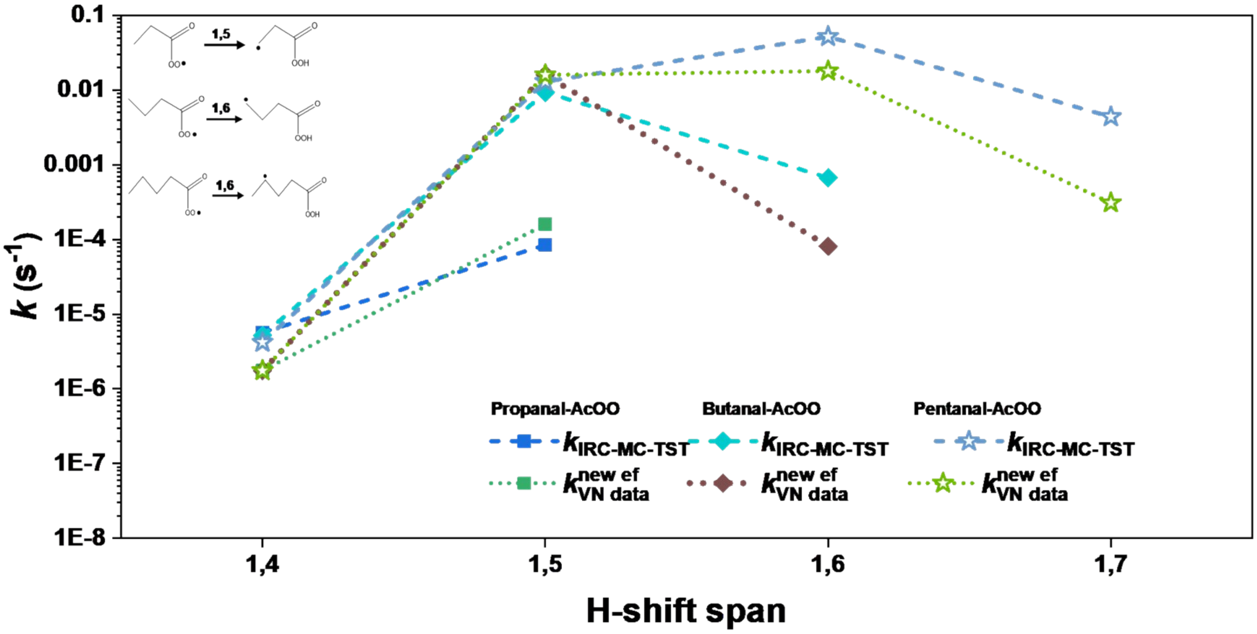

Fig. S5 Variation in the rate coefficients for the small-sized acyl peroxy radicals, *i.e.*, propanal, butanal, and pentanal with H-shift span. For clarity, we also provided the reaction schemes for 1,5 H-shift in propanal-AcOO, 1,6 H-shifts in butanal-AcOO, and 1,6 H-shift in pentanal-AcOO.

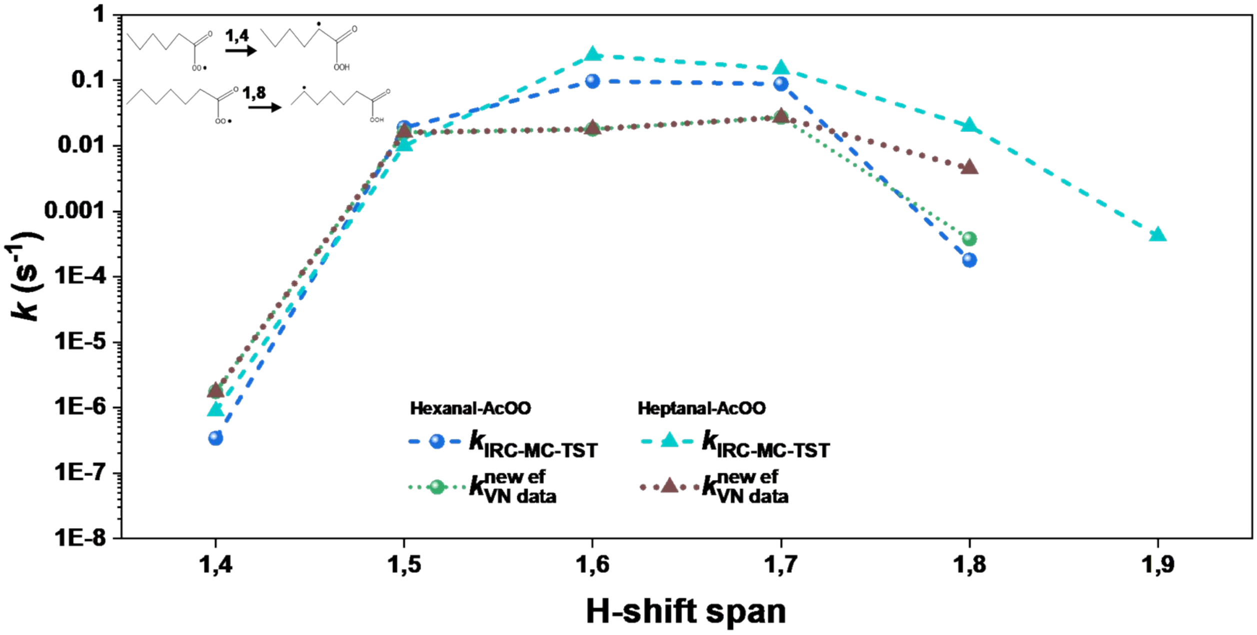

Fig. S6 Variation in the rate coefficients for the medium-sized acyl peroxy radicals, *i.e.*, hexanal and heptanal with H-shift span. For clarity, we also provide the reaction schemes for 1,4 H-shift in hexanal-AcOO, and 1,8 H-shift in heptanal-AcOO.

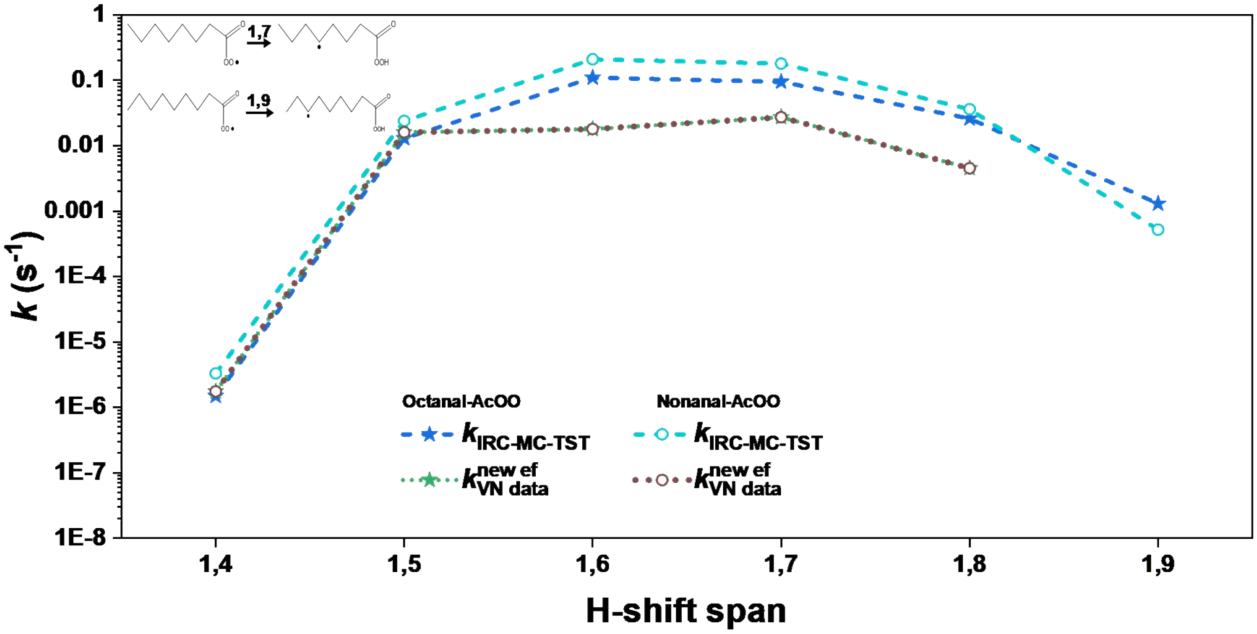

Fig. S7 Variation in the rate coefficients for the large sized acyl peroxy radicals octanal and nonanal with H-shift span. For clarity, we also provide the reaction schemes for 1,7 H-shift in octanal-AcOO, and 1,9 H-shift in nonanal-AcOO.

8. Table S5 Qualitative inspection of the influence of inductive effects in the determined H-shift rates investigated for a series of primary 1,n- H-shifts in comparison to the corresponding 1,n- secondary H-shifts with methyl and ethyl substitutions.

Reactions of interest	Addition of $-CH_3$		Addition of $-CH_2CH_3$	
(1º radical)	Reaction (2º radical)	$k_{IRC-MC-TST}^{secondary} / k_{IRC-MC-}^{primar}$	Reaction (2º radical)	$k_{IRC-MC-TST}^{secondary} / k_{IRC-MC-}^{primar}$
1,5 H-shift in Propanal-AcOO	1,5 H-shift in Butanal-AcOO	110.7	1,5 H-shift in Pentanal-AcOO	154.8
1,6 H-shift in Butanal-AcOO	1,6 H-shift in Pentanal-AcOO	77.6	1,6 H-shift in Hexanal-AcOO	144.8
1,7 H-shift in Pentanal-AcOO	1,7 H-shift in Hexanal-AcOO	20.0	1,7 H-shift in Heptanal-AcOO	34.1
1,8 H-shift in Hexanal-AcOO	1,8 H-shift in Heptanal-AcOO	111.1	1,8 H-shift in Octanal-AcOO	144.4
1,9 H-shift in Heptanal-AcOO	1,9 H-shift in Octanal-AcOO	3.1	1,9 H-shift in Nonanal-AcOO	1.2

9. Coupled cluster + zero-point correction energies (in a.u.), and frequencies (in cm^{-1}) obtained from ωB97X-D/aug-cc-pVTZ of the global minimum geometry of each of the species involved

Species	Formula	Energies (a.u.)	Frequencies (cm^{-1})
**Ethanal-AcOO**	$CH_3C(=O)OO•$	-303.1685493	143.92 211.58 339.42 500.44 543.44 574.84 786.97 993.82 1038.80 1178.35 1255.80 1386.05 1442.38 1450.85 1925.95 3099.79 3168.56 3208.93
**Ethanal-1,4-TS**		-303.121457	**-1903.81** 130.40 269.53 489.20 539.65 612.68 686.25 865.66 1024.10 1026.56 1033.63 1095.48 1199.02 1404.19 1741.14 1920.21 3156.26 3240.52
**Ethanal-1,4-rad**	$C•H_2C(=O)OOH$	-303.1644164	209.27 325.89 340.52 429.76 501.99 642.97 687.18 778.64 900.73 1025.38 1084.74 1308.29 1444.45 1491.46 1757.93 3225.87 3340.13 3507.38
**Propanal-AcOO**	$CH_3CH_2C(=O)OO•$	-342.3931836	95.05 137.99 219.09 234.67 332.22 526.80 553.24 602.71 766.85 813.39 1012.89 1057.92 1108.82 1120.59 1251.13 1285.07 1383.71 1432.36 1454.37 1503.45 1507.84 1923.28 3070.82 3083.19 3115.45 3149.46 3151.42
**Propanal-1,4-TS**		-342.3504947	**-1629.08** 79.65 180.88 196.44 247.34 502.32 562.36 673.81 690.16 800.16 819.13 1016.54 1043.57 1081.24 1129.30 1179.07 1188.00 1339.19 1411.38 1487.14 1495.06 1770.18 1917.31 3047.98 3119.88 3132.89 3152.86
**Propanal-1,4-rad**	$CH_3C•HC(=O)OOH$	-342.39756	85.91 118.81 215.18

			218.44 390.74 467.83<br>499.33 605.19 702.84<br>744.87 927.99 993.18<br>1004.26 1090.90 1156.55<br>1211.62 1404.21 1438.17<br>1476.12 1505.10 1533.05<br>1752.28 3028.46 3075.98<br>3155.81 3216.65 3539.89
**Propanal-1,5-TS**		-342.3540741	**-1798.87** 132.40 206.27<br>360.09 479.66 526.26<br>551.99 620.37 700.15<br>780.52 847.85 986.73<br>1056.44 1080.15 1112.84<br>1193.85 1212.10 1262.19<br>1330.06 1453.02 1473.83<br>1565.52 1877.05 3078.66<br>3117.34 3132.42 3212.65
**Propanal-1,5-rad**	C•H_2CH_2C(=O)OOH	-342.3848601	35.59 160.62 192.34<br>244.89 376.31 443.91<br>471.61 474.43 611.30<br>686.74 902.50 935.52<br>1004.06 1045.79 1122.72<br>1194.38 1239.11 1403.37<br>1444.93 1456.77 1524.80<br>1829.28 2981.39 3078.37<br>3181.75 3296.63 3567.84
**Butanal-AcOO**	$CH_3CH_2CH_2C$(=O)OO•	-381.6176886	78.03 103.47 139.47<br>156.21 251.57 304.63<br>366.30 530.20 562.11<br>591.26 752.98 826.26<br>889.29 931.07 1052.37<br>1074.42 1126.34 1143.83<br>1251.00 1257.41 1331.10<br>1335.83 1414.63 1427.83<br>1450.98 1499.31 1507.83<br>1514.25 1922.87 3047.03<br>3067.79 3075.72 3094.98<br>3109.16 3126.99 3131.93
**Butanal-1,4-TS**		-381.5753931	**-1623.87** 68.67 82.36<br>163.26 210.84 248.97<br>324.18 522.36 575.61<br>671.33 691.23 745.08<br>864.76 891.31 952.52

			1017.23 1068.93 1113.29 1146.37 1178.26 1188.67 1284.36 1300.45 1398.83 1427.05 1480.90 1506.28 1509.21 1763.78 1914.31 3038.94 3053.38 3086.87 3124.56 3128.02 3138.72
**Butanal-1,4-rad**	$CH_3CH_2C{\bullet}HC(=O)OOH$	-381.6212488	24.46 90.12 172.42 200.53 255.35 349.19 380.97 460.99 501.03 609.59 707.72 764.72 807.61 925.29 970.79 1031.01 1058.09 1094.87 1169.18 1209.35 1279.93 1341.65 1418.29 1468.02 1484.26 1504.93 1511.11 1521.95 1749.60 3010.16 3054.16 3110.90 3132.37 3137.38 3204.15 3539.82
**Butanal-1,5-TS**		-381.584304	**-1716.03** 100.40 131.71 193.57 243.69 340.65 480.23 494.21 544.27 591.40 687.78 778.03 859.22 899.82 932.65 1042.04 1072.43 1106.57 1143.83 1195.13 1224.41 1251.10 1318.52 1372.02 1423.51 1457.16 1487.66 1497.24 1570.30 1874.76 3030.88 3076.03 3099.48 3128.02 3133.63 3146.41
**Butanal-1,5-rad**	$CH_3C{\bullet}HCH_2C(=O)OOH$	-381.6139483	40.29 72.53 115.97 167.11 265.33 301.77 387.22 442.75 444.76 488.82 650.72 790.77 882.21 917.15 959.95 998.30 1024.33 1093.19 1155.33 1215.42 1283.08 1328.53 1389.87 1425.68 1470.57 1478.29 1487.49 1517.81 1813.55 2997.91 3055.07 3064.69 3131.11 3133.99 3205.11 3557.11
**Butanal-1,6-TS**		-381.58142	**-1878.23** 84.50 188.28 227.76 331.03 393.55

			459.04 513.47 581.24<br>641.73 721.01 767.95<br>886.14 898.79 933.53<br>1052.80 1061.61 1098.27<br>1117.25 1163.68 1220.28<br>1251.38 1297.27 1362.81<br>1371.95 1444.69 1468.58<br>1489.48 1498.88 1874.32<br>3050.47 3094.37 3104.76<br>3110.55 3162.39 3196.82
**Butanal-1,6-rad**	C•$H_2CH_2CH_2C(=O)OOH$	-381.610351	26.11 91.04 122.38 149.29<br>241.53 344.63 350.91<br>442.62 478.61 482.89<br>632.24 739.92 784.75<br>902.80 960.00 1005.19<br>1035.51 1104.34 1121.66<br>1172.68 1241.00 1301.10<br>1356.60 1416.79 1471.42<br>1475.06 1480.38 1512.01<br>1822.02 3001.65 3052.53<br>3064.73 3126.79 3161.59<br>3267.78 3566.02
**Pentanal-AcOO**	$CH_3CH_2CH_2CH_2C(=O)OO$•	-420.8424063	53.55 86.94 115.11 132.54<br>145.49 246.44 253.57<br>309.88 429.15 529.33<br>560.28 606.17 738.68<br>808.87 820.34 928.02<br>946.26 1041.25 1062.72<br>1088.34 1135.63 1146.75<br>1242.83 1250.30 1301.30<br>1321.05 1344.37 1382.75<br>1424.57 1426.61 1450.95<br>1496.41 1502.71 1506.13<br>1516.02 1921.44 3034.54<br>3045.83 3057.30 3064.55<br>3071.17 3093.72 3111.96<br>3117.58 3126.25
**Pentanal-1,4-TS**		-420.8002329	**-1628.40** 50.45 68.40<br>123.16 126.13 205.43<br>243.88 283.81 371.60<br>520.21 592.19 675.86<br>690.98 732.98 806.61<br>883.16 921.62 941.50<br>1017.35 1050.38 1082.67

			1129.50 1151.43 1175.84<br>1189.17 1263.74 1276.19<br>1335.25 1364.77 1414.03<br>1426.08 1477.46 1499.68<br>1506.55 1513.07 1765.74<br>1914.69 3026.74 3044.65<br>3048.10 3069.92 3089.27<br>3119.46 3126.69 3130.33
**Pentanal-1,4-rad**	$CH_3CH_2CH_2C{\bullet}HC(=O)OOH$	-420.8460175	27.79 61.67 109.85 183.83<br>213.84 247.92 317.12<br>375.15 411.27 473.61<br>499.91 619.44 712.29<br>763.01 794.78 857.50<br>899.57 976.73 994.14<br>1059.29 1084.72 1103.69<br>1168.12 1204.07 1254.22<br>1289.11 1354.65 1379.27<br>1427.41 1461.50 1488.78<br>1499.29 1506.25 1509.97<br>1526.96 1749.92 3035.27<br>3047.47 3051.41 3088.98<br>3112.63 3120.80 3128.54<br>3204.73 3538.70
**Pentanal-1,5-TS**		-420.8095115	**-1699.90** 70.23 83.04<br>119.55 213.81 238.90<br>284.02 380.94 479.28<br>505.23 552.45 590.36<br>701.91 755.32 790.28<br>857.15 918.13 971.47<br>1035.16 1054.80 1076.28<br>1110.25 1155.22 1191.20<br>1221.72 1246.45 1289.94<br>1315.05 1336.86 1418.03<br>1426.75 1457.78 1477.91<br>1506.60 1509.35 1577.06<br>1873.91 3013.31 3050.84<br>3066.59 3076.36 3119.57<br>3122.99 3130.97 3134.84
**Pentanal-1,5-rad**	$CH_3CH_2C{\bullet}HCH_2C(=O)OOH$	-420.838131	33.03 47.83 74.40 160.12<br>238.35 246.16 261.37<br>321.96 407.61 431.88<br>463.40 498.94 653.91<br>769.00 801.29 848.77<br>930.41 978.37 1026.98<br>1042.02 1069.11 1102.55

			1148.81 1207.86 1271.88<br>1285.60 1320.40 1348.15<br>1422.52 1430.28 1470.68<br>1475.25 1505.86 1509.90<br>1525.96 1813.58 2987.17<br>3012.57 3050.65 3066.10<br>3120.65 3130.58 3132.70<br>3190.34 3565.16
**Pentanal-1,6-TS**		-420.8113119	**-1778.60** 47.78 123.39<br>185.05 207.61 260.28<br>330.84 389.82 397.90<br>512.38 528.29 581.85<br>735.52 768.57 844.50<br>871.14 907.98 972.82<br>1017.39 1074.87 1105.17<br>1113.39 1134.64 1178.22<br>1218.14 1244.19 1298.72<br>1335.24 1365.91 1397.70<br>1417.15 1444.74 1477.51<br>1487.04 1495.39 1497.45<br>1872.18 3025.91 3045.88<br>3083.29 3095.08 3100.43<br>3105.67 3140.74 3161.88
**Pentanal-1,6-rad**	CH_3C•$HCH_2CH_2C(=O)OOH$	-420.8397415	26.19 41.88 83.08 116.08<br>194.26 219.94 296.95<br>339.20 384.37 429.27<br>469.65 504.66 631.20<br>718.69 855.77 897.95<br>949.58 980.52 1000.23<br>1021.16 1057.93 1120.75<br>1150.32 1195.85 1231.80<br>1284.22 1331.70 1397.57<br>1411.06 1438.05 1471.34<br>1480.21 1482.95 1489.55<br>1530.93 1823.17 2977.15<br>2983.51 3049.67 3056.43<br>3079.66 3127.64 3133.55<br>3180.74 3556.69
**Pentanal-1,7-TS**		-420.809389	**-1889.29** 89.88 151.68<br>199.17 281.15 307.68<br>363.74 460.51 480.33<br>504.51 581.80 589.02<br>730.13 772.75 810.75<br>831.35 918.14 967.17<br>984.75 1068.00 1081.39

			1100.13 1136.22 1150.70 1190.06 1240.35 1264.24 1305.74 1366.38 1383.92 1404.12 1448.80 1472.06 1482.28 1489.03 1502.99 1872.09 3021.63 3058.93 3070.08 3095.05 3109.41 3115.58 3169.18 3184.72
**Pentanal-1,7-rad**	C•$H_2CH_2CH_2CH_2C$(=O)OOH	-420.8355378	20.87 54.06 129.33 144.00 199.25 225.86 309.05 379.64 426.33 464.44 490.91 502.26 625.35 705.95 807.00 854.77 901.52 952.77 990.60 1003.02 1051.45 1105.78 1130.01 1181.47 1212.10 1273.81 1296.75 1372.34 1397.19 1410.52 1470.38 1471.32 1475.87 1496.20 1516.37 1817.50 2973.47 3053.69 3066.13 3073.35 3113.75 3128.69 3152.78 3259.78 3556.86
**Hexanal-AcOO**	$CH_3CH_2CH_2CH_2CH_2C$(=O)OO•	-460.0669611	52.98 61.04 86.53 128.84 137.68 148.07 221.32 244.60 248.42 397.87 435.34 529.57 562.51 610.25 735.20 769.36 821.66 861.61 921.48 984.03 1031.03 1061.10 1075.88 1090.14 1140.20 1149.43 1234.55 1250.68 1282.63 1300.24 1344.26 1346.00 1353.73 1408.19 1422.62 1430.11 1451.59 1493.98 1496.94 1506.03 1506.95 1516.41 1922.97 3022.86 3039.56 3044.47 3051.73 3059.64 3071.94 3076.86 3095.70 3113.64 3115.47 3123.97
**Hexanal-1,4-TS**		-460.0238594	**-1637.38** 34.78 63.86 102.14 153.56 182.85 210.20 287.08 300.13

			395.09 481.84 513.09 546.30 651.31 692.96 728.31 787.82 825.15 878.87 905.79 975.94 983.31 1018.45 1054.59 1103.44 1122.98 1145.37 1174.10 1183.67 1236.42 1270.30 1327.71 1341.97 1374.79 1402.92 1407.05 1427.44 1465.71 1499.90 1501.65 1512.82 1517.69 1768.73 1913.28 3032.52 3041.92 3051.44 3059.58 3080.80 3087.84 3100.13 3113.53 3125.31 3126.15
**Hexanal-1,4-rad**	$CH_3CH_2CH_2CH_2C$•HC(=O)OOH	-460.0703293	37.66 51.74 93.05 149.55 174.51 216.81 273.37 284.16 366.06 392.20 444.90 485.76 501.25 625.26 711.52 764.45 769.74 819.03 878.89 917.97 965.06 999.91 1016.43 1082.48 1109.90 1112.38 1158.11 1193.80 1239.85 1261.67 1321.75 1349.24 1385.11 1395.29 1429.11 1462.71 1484.79 1497.72 1501.54 1509.26 1521.89 1529.47 1747.02 3032.42 3037.77 3045.67 3046.20 3079.28 3087.89 3116.84 3123.78 3131.37 3204.30 3543.60
**Hexanal-1,5-TS**		-460.0343137	**-1704.50** 59.27 71.05 73.63 153.05 181.31 243.45 246.27 354.04 400.29 479.95 503.27 553.82 590.44 703.45 739.02 780.38 837.03 879.38 939.04 954.20 1029.87 1055.39 1069.36 1081.49 1127.64 1154.57 1190.28 1219.26 1239.85 1271.52 1294.14 1323.44 1343.54 1381.22 1425.26 1426.71 1457.98 1474.88

			1499.50 1506.78 1512.86 1574.94 1873.74 3001.29 3041.58 3046.11 3054.03 3075.72 3082.65 3116.94 3121.45 3127.91 3130.63
**Hexanal-1,5-rad**	$CH_3CH_2CH_2C{\bullet}HCH_2C(=O)OOH$	-460.0623119	29.20 45.90 75.83 125.82 157.00 230.62 251.30 299.65 316.72 372.13 421.89 436.88 485.16 497.78 653.63 778.50 784.72 840.87 872.57 913.00 939.12 997.06 1030.46 1063.82 1080.45 1114.49 1151.85 1207.51 1248.00 1283.30 1296.40 1324.87 1354.04 1382.12 1421.37 1430.00 1473.27 1478.25 1498.62 1505.06 1512.35 1517.77 1813.82 2990.23 3044.64 3045.33 3063.37 3068.56 3085.60 3116.44 3126.15 3136.57 3189.86 3561.26
**Hexanal-1,6-TS**		-460.0365356	-**1743.82** 28.09 81.42 106.27 167.70 230.92 249.65 304.48 358.81 397.48 440.62 517.05 542.16 583.08 735.20 761.95 769.44 869.45 900.09 947.92 952.73 1021.62 1056.54 1075.45 1102.92 1125.52 1143.03 1174.72 1206.24 1231.96 1284.19 1307.01 1311.27 1367.47 1373.59 1412.27 1426.27 1447.72 1477.50 1488.58 1497.65 1506.64 1509.88 1871.60 3006.72 3049.45 3050.43 3063.66 3081.44 3095.04 3105.43 3122.01 3133.27 3161.17
**Hexanal-1,6-rad**	$CH_3CH_2C{\bullet}HCH_2CH_2C(=O)OOH$	-460.0641677	21.39 27.54 41.76 101.07 163.98 196.55 234.63 260.38 328.62 366.85 408.01 417.24 460.38

			496.84 631.62 714.82 774.50 873.31 902.98 949.23 973.44 1008.97 1044.20 1066.17 1072.33 1126.38 1155.25 1191.84 1227.69 1273.50 1281.67 1305.02 1378.99 1400.15 1422.01 1444.25 1468.91 1478.68 1483.01 1504.71 1509.58 1511.51 1822.04 2962.70 2979.87 3017.16 3049.36 3062.24 3077.37 3119.36 3128.68 3132.20 3162.66 3551.15
**Hexanal-1,7-TS**		-460.0381727	**-1733.34** 70.32 118.03 167.80 175.57 214.42 284.12 326.82 336.22 411.22 505.55 518.79 569.28 603.29 711.75 760.77 805.75 846.64 886.77 916.62 960.47 1004.16 1034.29 1082.75 1097.69 1120.71 1134.00 1170.81 1210.45 1231.63 1272.14 1322.82 1361.80 1373.11 1393.38 1404.49 1423.83 1452.15 1475.24 1487.91 1491.51 1493.40 1514.02 1861.37 3014.20 3026.47 3064.70 3076.76 3097.83 3102.05 3108.01 3112.53 3143.95 3157.97
**Hexanal-1,7-rad**	$CH_3C{\bullet}HCH_2CH_2CH_2C(=O)OOH$	-460.0646598	46.71 56.33 90.43 118.02 128.08 201.64 255.65 265.19 339.86 378.04 410.01 441.70 488.79 518.68 624.76 728.62 821.51 863.02 906.56 938.19 965.89 1000.04 1016.70 1020.56 1064.03 1120.32 1160.65 1195.56 1229.23 1257.78 1303.55 1359.88 1371.99 1401.04 1401.86 1426.29 1476.40 1479.06 1489.31 1490.89 1501.14 1523.95 1814.74

			2994.33 3012.72 3050.60 3064.36 3072.08 3077.43 3111.45 3119.93 3128.52 3198.56 3559.79
**Hexanal-1,8-TS**		-460.0315614	**-1858.46** 70.53 126.16 142.58 174.66 209.19 318.89 375.72 385.85 448.24 516.20 522.82 582.87 639.78 703.22 745.39 779.75 837.02 849.15 917.68 982.60 1006.61 1038.72 1076.43 1084.51 1112.95 1132.75 1149.19 1203.89 1239.79 1258.59 1282.14 1319.45 1363.64 1384.01 1398.65 1408.04 1430.71 1463.59 1476.72 1494.39 1503.77 1507.50 1866.45 3013.88 3045.87 3057.03 3066.00 3090.00 3097.05 3101.06 3107.50 3175.79 3176.29
**Hexanal-1,8-rad**	C•$H_2CH_2CH_2CH_2CH_2C$(=O)OOH	-460.0595377	27.85 40.59 75.71 144.09 168.81 214.05 236.98 308.91 339.92 403.11 438.53 481.49 501.20 515.75 636.03 714.97 791.71 818.68 870.96 928.96 946.37 954.84 1022.24 1030.86 1080.92 1112.90 1133.72 1157.78 1218.43 1235.78 1286.33 1321.45 1372.81 1387.92 1402.87 1407.96 1470.41 1473.94 1489.44 1494.85 1503.42 1508.39 1820.31 2971.57 3046.55 3053.20 3066.13 3083.99 3089.36 3113.17 3139.10 3152.04 3256.25 3553.57
**Heptanal-AcOO**	$CH_3CH_2CH_2CH_2CH_2CH_2C$(=O)OO•	-499.2916265	34.81 52.45 69.38 102.55 130.14 139.34 160.94 176.41 226.40 245.18 318.97 416.94 465.33

			529.95 563.74 609.24 733.32 749.85 809.39 826.79 901.65 913.07 1008.09 1033.20 1044.90 1077.91 1081.10 1091.06 1142.24 1152.73 1228.63 1249.61 1268.85 1284.78 1330.16 1330.38 1344.56 1350.82 1385.00 1419.33 1421.75 1430.53 1452.60 1493.85 1494.33 1501.19 1505.86 1510.29 1517.58 1921.65 3021.62 3026.30 3036.74 3043.01 3047.06 3058.68 3061.10 3073.10 3079.61 3095.97 3113.10 3115.12 3120.91
**Heptanal-1,4-TS**		-499.2485566	**-1645.41** 25.87 32.38 77.55 118.99 148.64 173.32 202.75 232.76 293.63 318.10 411.63 490.44 519.79 542.63 645.51 691.88 739.00 778.13 803.11 856.91 890.30 925.08 956.59 1003.80 1017.64 1024.16 1086.33 1111.04 1129.95 1145.18 1175.99 1182.54 1224.52 1263.24 1312.45 1323.83 1349.36 1360.65 1389.95 1392.36 1422.89 1428.23 1463.31 1494.46 1497.91 1509.67 1512.14 1515.52 1767.72 1912.86 3028.11 3031.72 3038.82 3047.05 3051.02 3070.87 3076.70 3081.73 3099.89 3118.78 3124.40 3127.89
**Heptanal-1,4-rad**	$CH_3CH_2CH_2CH_2CH_2C$•HC(=O)OOH	-499.2951792	29.04 36.53 79.44 90.44 144.08 181.22 236.42 244.17 261.93 307.12 371.11 394.98 456.95 498.75 503.02 624.94 711.69 745.50 769.04 805.45 850.20 890.95 930.75 956.75 1001.84

			1038.05 1049.47 1088.29<br>1107.59 1122.65 1160.41<br>1192.65 1237.36 1250.23<br>1302.68 1331.23 1346.60<br>1364.70 1389.76 1418.56<br>1422.44 1463.79 1489.13<br>1495.39 1500.62 1503.98<br>1506.79 1515.29 1532.28<br>1747.53 3023.63 3034.33<br>3038.25 3041.78 3046.29<br>3065.31 3085.29 3090.45<br>3113.39 3122.44 3126.68<br>3204.03 3531.90
**Heptanal-1,5-TS**		-499.258317	**-1706.73** 37.51 76.63<br>86.02 116.55 170.50<br>222.22 252.98 290.50<br>304.74 383.03 465.86<br>482.86 505.97 565.46<br>590.46 707.64 744.26<br>775.77 811.85 851.56<br>873.05 912.53 983.49<br>1003.58 1035.86 1059.92<br>1066.17 1110.82 1128.68<br>1147.55 1180.62 1222.45<br>1230.49 1252.85 1278.72<br>1314.97 1325.44 1342.05<br>1385.03 1398.34 1424.16<br>1429.23 1459.40 1475.18<br>1496.21 1499.42 1509.42<br>1518.76 1583.56 1875.09<br>3010.41 3038.04 3045.60<br>3047.31 3063.72 3076.64<br>3078.10 3088.19 3118.13<br>3118.48 3129.99 3131.64
**Heptanal-1,5-rad**	$CH_3CH_2CH_2CH_2C{\bullet}HCH_2C(=O)OOH$	-499.2871682	28.15 40.23 61.23 91.69<br>136.57 179.15 226.58<br>253.02 262.91 294.63<br>330.03 398.13 424.62<br>439.52 487.33 561.27<br>644.72 747.16 783.50<br>822.81 846.91 901.07<br>924.04 952.63 986.50<br>1012.21 1040.14 1068.59<br>1115.12 1117.26 1157.97<br>1185.33 1248.53 1263.27<br>1282.45 1326.26 1337.08

			1341.48 1364.51 1404.04 1421.07 1423.07 1471.07 1486.44 1496.67 1503.14 1507.21 1515.43 1526.40 1817.45 3004.54 3034.19 3042.33 3045.82 3059.82 3062.46 3080.45 3094.87 3113.98 3122.10 3125.40 3206.66 3555.56
**Heptanal-1,6-TS**		-499.2613129	**-1736.78** 9.87 74.53 78.48 129.25 146.88 207.69 243.00 269.61 356.73 384.49 419.34 452.13 508.92 578.66 606.64 731.75 740.10 773.86 829.91 886.85 900.54 919.22 982.67 1001.10 1048.59 1074.64 1088.28 1106.21 1119.96 1138.40 1167.89 1182.20 1230.69 1270.09 1291.51 1297.01 1341.63 1349.18 1374.45 1386.87 1421.32 1424.36 1467.55 1478.08 1482.27 1499.78 1501.16 1506.71 1513.02 1872.31 2996.71 3040.96 3044.87 3050.80 3055.65 3084.15 3096.01 3103.50 3116.10 3117.02 3126.12 3163.28
**Heptanal-1,6-rad**	$CH_3CH_2CH_2C{\bullet}HCH_2CH_2C(=O)OOH$	-499.2885605	38.09 46.17 73.72 93.59 154.55 181.61 217.83 239.30 274.65 327.38 341.40 373.98 430.68 463.05 495.18 510.14 633.46 725.15 770.22 848.92 857.53 895.95 914.87 960.62 992.59 1024.87 1041.42 1055.47 1090.40 1128.29 1149.60 1196.96 1216.44 1257.61 1287.41 1293.09 1336.16 1359.90 1380.57 1393.51 1423.07 1433.83 1477.51 1485.19 1488.62 1501.23 1503.49 1512.42 1520.30

			1820.03 2995.99 3010.05 3044.34 3049.93 3073.96 3077.43 3083.67 3089.89 3115.66 3125.45 3135.36 3179.95 3552.92
**Heptanal-1,7-TS**		-499.2639135	**-1710.89** 57.46 83.78 117.24 161.07 198.74 239.05 252.64 288.15 333.26 379.96 422.45 506.55 527.17 571.03 604.99 711.39 761.45 769.03 831.65 834.21 911.91 925.36 968.86 1010.05 1035.05 1063.20 1088.48 1098.01 1120.43 1137.92 1172.88 1206.98 1231.31 1263.60 1297.98 1319.00 1327.05 1371.87 1389.07 1402.87 1417.98 1427.17 1451.95 1475.85 1481.41 1490.09 1506.40 1508.14 1511.10 1860.87 3004.08 3015.60 3051.29 3064.64 3075.48 3078.26 3093.28 3097.77 3112.07 3124.38 3136.01 3157.47
**Heptanal-1,7-rad**	$CH_3CH_2C{\bullet}HCH_2CH_2CH_2C(=O)OOH$	-499.2894119	43.98 57.44 82.77 104.19 116.92 191.92 233.72 237.86 255.14 295.73 341.50 366.91 427.40 445.09 494.03 533.19 621.37 724.59 778.32 813.33 867.93 907.73 937.46 964.05 1008.61 1034.93 1052.92 1062.97 1069.94 1121.97 1158.24 1195.99 1226.31 1253.48 1281.15 1297.27 1332.01 1364.24 1383.81 1403.08 1422.96 1434.99 1471.28 1477.33 1488.58 1497.54 1504.28 1508.83 1534.04 1812.87 2971.43 3007.28 3021.07 3049.62 3065.74 3071.25 3077.03 3112.37 3119.78 3127.27 3128.64

			3186.04 3555.41
**Heptanal-1,8-TS**		-499.2617743	**-1706.70** 59.61 84.33 121.23 141.13 196.52 235.02 241.44 284.80 366.18 383.12 416.94 465.90 521.82 555.56 592.22 694.97 770.74 779.10 859.33 880.79 896.13 916.28 957.29 1021.70 1049.68 1083.30 1096.10 1108.44 1123.23 1133.26 1159.97 1173.07 1237.40 1265.28 1286.53 1304.37 1332.81 1382.58 1394.10 1398.04 1410.38 1419.06 1447.77 1482.75 1490.22 1493.52 1497.55 1500.59 1503.21 1862.01 3015.57 3029.54 3044.88 3055.63 3061.72 3087.63 3092.94 3096.20 3102.03 3104.55 3140.11 3168.22
**Heptanal-1,8-rad**	$CH_3C•HCH_2CH_2CH_2CH_2C(=O)OOH$	-499.2892745	22.50 34.05 53.02 79.81 125.46 137.71 210.97 217.99 251.99 289.37 322.41 344.03 408.21 459.99 489.46 567.48 625.33 700.89 791.60 830.12 864.06 906.65 953.32 987.72 991.86 1009.11 1029.01 1069.52 1083.75 1133.48 1161.48 1177.89 1219.11 1251.98 1281.08 1327.45 1343.43 1376.93 1388.17 1405.01 1417.39 1429.07 1473.48 1476.38 1482.54 1489.77 1496.31 1509.70 1516.11 1816.93 2970.47 2991.86 3028.92 3042.50 3060.35 3066.01 3069.35 3082.03 3107.74 3123.97 3124.84 3203.82 3559.94
**Heptanal-1,9-TS**		-499.2584072	**-1867.51** 61.14 90.08 171.93 183.32 235.10

			275.36 293.34 327.49 330.97 397.20 445.08 479.17 515.67 560.36 618.60 668.74 738.65 796.98 840.44 875.57 887.55 931.26 965.10 1002.82 1045.50 1063.94 1074.31 1105.24 1122.19 1134.69 1140.72 1183.90 1216.79 1244.82 1266.04 1302.91 1326.48 1348.60 1374.14 1395.86 1406.79 1419.43 1453.79 1471.31 1472.33 1480.84 1493.91 1503.31 1521.34 1874.72 3017.37 3036.31 3060.12 3065.72 3072.01 3075.77 3081.26 3095.36 3107.28 3109.82 3132.44 3180.78
**Heptanal-1,9-rad**	C•$H_2CH_2CH_2CH_2CH_2CH_2$C(=O)OOH	-499.2841152	30.35 38.39 56.71 122.32 162.56 173.66 178.73 237.89 269.54 326.96 347.81 412.29 441.60 485.41 501.52 514.40 643.26 727.11 765.95 820.31 858.22 894.65 908.98 940.59 981.39 993.90 1034.18 1079.55 1091.31 1112.67 1135.29 1160.84 1211.79 1225.56 1283.96 1303.25 1337.14 1347.90 1380.00 1393.05 1394.90 1420.30 1470.52 1472.62 1488.69 1496.35 1498.90 1508.58 1514.10 1822.26 2965.90 3030.46 3036.19 3044.13 3056.77 3070.49 3088.82 3091.67 3108.00 3145.03 3153.16 3258.90 3555.44
**Octanal-AcOO**	$CH_3CH_2CH_2CH_2CH_2CH_2CH_2$C(=O)OO•	-538.5162965	35.74 41.52 56.36 93.40 101.65 136.50 145.72 154.26 158.02 202.96 245.57 272.62 358.57

			435.13 481.75 529.94 563.25 611.25 733.64 740.98 777.19 826.73 846.25 912.60 933.65 1018.98 1025.86 1049.17 1060.61 1078.70 1090.42 1093.76 1142.21 1154.48 1223.57 1248.71 1259.69 1269.92 1311.76 1315.88 1336.52 1348.62 1351.19 1361.80 1401.87 1420.99 1423.63 1429.73 1451.81 1492.46 1493.32 1497.63 1503.82 1505.31 1512.18 1517.91 1921.74 3020.46 3022.46 3029.65 3037.26 3041.93 3044.75 3053.36 3059.15 3069.23 3072.80 3082.09 3096.11 3111.73 3115.02 3120.43
**Octanal-1,4-TS**		-538.473369	**-1618.87** 22.63 40.57 59.93 83.31 106.19 181.89 197.82 205.69 222.49 249.53 294.04 338.07 442.45 506.83 526.31 568.75 661.47 689.23 726.82 763.94 793.88 837.81 858.46 882.74 930.68 958.46 1016.90 1021.13 1042.55 1056.37 1080.25 1119.30 1134.89 1149.69 1170.79 1184.52 1218.46 1254.64 1298.97 1314.59 1335.95 1338.55 1349.38 1360.65 1401.53 1413.71 1421.57 1424.25 1467.78 1492.43 1498.86 1503.19 1505.49 1506.62 1516.80 1766.20 1912.45 3027.16 3030.47 3037.18 3039.79 3042.30 3050.98 3060.31 3074.78 3079.32 3085.18 3098.96 3111.45 3122.79 3135.06
**Octanal-1,4-rad**	$CH_3CH_2CH_2CH_2CH_2CH_2C$•HC(=O)OOH	-538.5200175	25.02 28.86 60.70 79.13 113.73 134.65 183.80

		201.17 237.74 254.50<br>287.47 364.57 384.37<br>405.86 450.68 494.44<br>500.54 621.72 711.71<br>739.31 769.12 776.13<br>841.35 868.93 905.83<br>933.28 970.17 996.48<br>1019.22 1059.68 1075.93<br>1089.99 1116.54 1127.67<br>1160.32 1191.65 1232.53<br>1245.06 1283.60 1308.19<br>1334.44 1348.88 1357.41<br>1387.14 1392.05 1421.68<br>1426.51 1464.49 1489.41<br>1494.89 1496.34 1503.86<br>1505.48 1506.34 1516.21<br>1522.78 1746.96 3021.03<br>3028.09 3035.04 3036.01<br>3042.02 3047.10 3060.58<br>3067.46 3086.49 3092.38<br>3112.37 3120.18 3126.96<br>3203.86 3526.83
**Octanal-1,5-TS**	-538.4836187	**-1688.96** 37.02 44.12<br>58.33 97.74 117.62 129.88<br>163.50 217.60 246.25<br>264.23 302.85 399.31<br>456.69 480.79 519.70<br>555.19 590.91 704.14<br>733.58 751.16 782.40<br>829.33 889.85 917.42<br>922.80 997.89 1020.86<br>1056.82 1059.82 1067.09<br>1081.92 1089.35 1139.06<br>1159.69 1192.35 1221.02<br>1232.26 1245.29 1273.31<br>1299.42 1318.37 1332.27<br>1344.98 1347.91 1381.86<br>1418.84 1422.54 1429.74<br>1458.10 1475.11 1494.03<br>1497.05 1506.36 1506.79<br>1516.37 1580.40 1873.66<br>3002.40 3023.22 3030.88<br>3038.16 3043.10 3050.16<br>3055.65 3072.08 3076.06<br>3086.40 3113.29 3121.41<br>3121.81 3130.94

**Octanal-1,5-rad**	$CH_3CH_2CH_2CH_2CH_2C{\bullet}HCH_2C(=O)OOH$	-538.5116612	41.29 56.56 68.58 76.27 145.85 162.58 186.60 227.71 261.14 267.00 294.56 324.54 356.21 418.57 437.39 458.22 482.86 515.70 642.47 738.37 783.55 794.73 840.95 873.93 895.85 930.81 966.34 972.99 1012.44 1048.59 1052.18 1092.73 1116.73 1122.67 1152.78 1182.93 1224.12 1251.29 1299.47 1304.62 1323.07 1330.09 1341.82 1356.90 1392.26 1416.63 1419.47 1426.94 1470.80 1486.15 1492.40 1497.19 1502.89 1506.73 1517.89 1523.99 1817.58 2988.05 3030.69 3039.21 3041.49 3043.07 3058.04 3065.34 3068.84 3084.94 3092.71 3114.24 3122.27 3129.48 3194.81 3561.71
**Octanal-1,6-TS**		-538.485198	**-1753.64** 32.34 45.38 78.95 114.80 151.72 183.86 237.48 254.98 289.40 306.95 358.23 396.75 414.87 492.90 513.70 549.90 583.49 733.50 745.13 769.56 807.64 857.64 871.94 906.41 915.97 978.16 1011.07 1039.72 1047.97 1073.51 1090.01 1122.41 1134.01 1140.79 1170.87 1196.93 1249.00 1259.48 1271.96 1299.06 1312.63 1332.45 1366.18 1373.81 1390.98 1398.48 1417.35 1428.39 1457.47 1472.64 1483.71 1494.97 1496.19 1500.13 1512.16 1518.43 1872.16 3004.28 3036.62 3045.25 3047.24 3050.76 3059.94 3075.27 3079.24

			3088.10 3093.48 3104.15 3117.91 3129.43 3161.67
**Octanal-1,6-rad**	$CH_3CH_2CH_2CH_2C{\bullet}HCH_2CH_2C(=O)OOH$	-538.5138592	41.99 51.51 69.73 91.30 136.26 159.79 173.79 221.56 254.11 259.29 294.93 333.57 349.26 403.67 430.76 457.55 480.30 535.93 633.52 726.15 747.70 815.81 843.15 887.45 904.07 939.79 964.90 977.25 1006.83 1046.65 1051.95 1070.06 1117.23 1130.61 1146.93 1196.59 1215.69 1249.28 1263.49 1289.74 1327.15 1333.19 1348.14 1362.48 1391.83 1408.65 1419.81 1432.54 1477.97 1485.86 1489.28 1498.41 1503.16 1506.10 1517.49 1537.67 1819.19 2997.99 3005.20 3036.73 3040.02 3043.26 3068.25 3077.34 3077.88 3086.40 3091.36 3110.78 3120.62 3135.50 3180.67 3565.15
**Octanal-1,7-TS**		-538.4881739	**-1766.33** 51.96 71.89 75.28 113.49 153.78 200.27 217.63 245.14 274.86 288.40 351.33 412.34 423.41 510.07 551.66 580.99 588.79 728.98 738.62 758.51 808.67 818.50 852.87 908.50 936.93 953.22 1004.93 1028.66 1065.62 1081.86 1087.35 1105.81 1137.49 1145.23 1173.87 1205.34 1234.73 1248.65 1276.56 1295.08 1315.04 1342.48 1362.66 1376.99 1388.75 1408.35 1419.32 1424.91 1462.18 1475.79 1485.95 1499.51 1501.67 1506.49 1510.39 1512.95 1869.45 2996.64 3020.43

			3043.68 3044.55 3059.12 3068.06 3076.29 3083.46 3097.96 3110.62 3114.66 3115.75 3125.62 3171.86
**Octanal-1,7-rad**	$CH_3CH_2CH_2C{\bullet}HCH_2CH_2CH_2C(=O)OOH$	-538.5137578	39.54 66.47 76.46 87.35 112.73 161.94 194.52 212.79 247.04 260.97 301.52 320.49 353.38 366.35 432.27 457.06 478.21 543.56 620.73 726.77 773.69 827.76 851.57 873.35 905.75 934.25 943.24 987.98 1013.64 1055.91 1066.92 1077.86 1096.30 1128.85 1158.37 1188.78 1228.55 1244.98 1257.41 1293.81 1314.23 1342.54 1364.77 1383.80 1386.71 1403.78 1426.38 1436.98 1468.52 1476.97 1488.06 1496.46 1499.38 1507.38 1515.77 1522.51 1816.53 2975.68 2995.01 3005.96 3044.91 3045.19 3064.65 3070.82 3076.31 3080.65 3111.69 3117.81 3123.82 3127.30 3189.69 3564.11
**Octanal-1,8-TS**		-538.4870457	**-1677.33** 49.82 74.77 88.63 126.60 132.53 191.54 229.31 249.79 265.71 357.23 366.94 400.19 420.27 470.78 520.56 554.16 592.32 694.54 767.81 775.45 785.43 872.93 881.19 930.10 937.11 966.00 1004.37 1046.93 1064.93 1095.13 1097.51 1108.84 1131.01 1135.05 1168.24 1170.85 1228.93 1253.31 1280.78 1287.47 1310.39 1326.34 1367.84 1388.44 1397.99 1404.97 1419.89 1424.76 1447.50 1476.97 1488.85 1497.56 1502.14

			1504.25 1506.08 1511.49 1861.86 3008.47 3018.62 3046.24 3049.57 3055.27 3061.56 3067.32 3080.37 3092.15 3096.32 3103.67 3121.45 3133.32 3164.42
**Octanal-1,8-rad**	$CH_3CH_2C{\bullet}HCH_2CH_2CH_2CH_2C(=O)OOH$	-538.5135741	19.23 25.64 31.38 73.84 81.70 131.27 203.84 211.76 230.83 232.11 272.58 302.07 321.48 362.84 419.89 469.26 494.01 581.69 625.05 700.17 773.73 801.40 839.65 851.02 911.96 947.52 979.66 1000.91 1040.99 1052.49 1063.33 1075.02 1087.04 1129.29 1163.01 1179.57 1216.24 1251.33 1280.17 1287.26 1317.99 1334.51 1349.00 1382.21 1404.29 1415.89 1420.19 1432.36 1475.62 1476.21 1479.89 1494.39 1504.31 1508.17 1508.88 1520.40 1817.18 2964.41 2971.71 3004.81 3042.33 3046.48 3060.33 3064.22 3070.04 3081.47 3108.30 3115.14 3124.73 3125.88 3182.62 3562.37
**Octanal-1,9-TS**		-538.4846642	**-1709.37** 51.06 74.07 128.55 149.87 180.55 192.20 207.19 248.68 292.58 316.00 365.67 414.79 424.11 490.11 515.01 555.67 600.03 699.07 742.23 772.15 825.33 845.54 888.58 892.71 950.37 970.28 1000.52 1045.09 1060.79 1082.76 1094.65 1119.15 1129.87 1132.41 1166.34 1174.31 1234.44 1247.47 1268.64 1307.90 1318.65 1341.96 1376.79 1390.34 1403.67 1405.03 1413.47

			1421.12 1457.08 1483.66 1486.45 1489.21 1496.58 1498.84 1504.79 1510.97 1860.85 3011.44 3030.43 3037.46 3055.80 3057.22 3060.27 3077.76 3083.95 3089.03 3099.12 3104.28 3108.02 3140.17 3157.49
**Octanal-1,9-rad**	$CH_3C•HCH_2CH_2CH_2CH_2CH_2C(=O)OOH$	-538.5133189	27.81 39.12 43.33 61.80 114.07 127.65 150.77 173.02 227.20 239.57 281.32 338.05 359.00 391.09 446.16 455.19 503.22 546.39 641.20 723.47 763.48 834.13 838.19 889.50 912.97 941.32 962.51 993.22 1009.54 1017.82 1046.97 1090.39 1101.14 1129.05 1159.22 1171.90 1199.17 1238.94 1281.80 1294.58 1331.48 1342.92 1366.41 1388.23 1394.69 1397.89 1416.69 1424.43 1476.87 1485.12 1488.03 1489.90 1496.35 1499.52 1508.47 1540.25 1823.56 2990.58 2994.45 3034.17 3041.36 3047.78 3056.13 3066.23 3076.41 3085.64 3092.64 3106.81 3116.23 3142.23 3196.71 3558.70
**Nonanal-AcOO**	$CH_3CH_2CH_2CH_2CH_2CH_2CH_2CH_2C(=O)OO•$	-577.7409351	22.19 35.08 46.64 75.45 94.60 121.50 124.23 141.64 151.63 166.23 189.18 226.54 244.88 343.52 357.55 468.74 485.13 529.91 563.96 611.89 732.27 736.44 758.90 808.41 826.54 878.69 911.74 958.90 1014.70 1037.69 1038.43 1056.32 1072.17 1089.09 1090.61 1096.86 1143.54

			1155.87 1221.03 1246.84 1253.99 1261.41 1298.53 1303.92 1335.24 1336.81 1343.88 1350.60 1352.71 1384.31 1413.17 1420.55 1425.68 1430.03 1452.49 1491.68 1492.45 1495.97 1499.42 1505.51 1506.43 1513.58 1518.16 1921.68 3019.44 3021.81 3023.30 3030.94 3036.40 3042.01 3042.95 3049.31 3058.75 3059.43 3073.13 3073.27 3083.23 3096.15 3111.98 3115.13 3119.36
**Nonanal-1,4-TS**		-577.6981309	**-1628.11** 15.45 34.01 55.34 78.55 89.60 130.36 158.86 183.53 210.05 219.36 249.22 263.52 328.24 406.27 431.08 505.92 524.40 567.66 661.06 689.12 729.63 750.70 780.18 827.84 842.30 872.71 918.89 922.33 966.33 1007.76 1016.59 1032.72 1067.94 1078.10 1086.51 1119.19 1138.39 1152.25 1170.85 1184.14 1212.12 1242.26 1285.39 1301.49 1322.92 1325.50 1345.78 1350.48 1354.19 1385.97 1401.22 1419.96 1422.55 1427.15 1467.23 1492.21 1495.96 1500.98 1505.58 1506.80 1509.53 1518.49 1766.07 1911.25 3021.66 3027.00 3031.24 3035.16 3040.03 3042.81 3048.27 3052.86 3065.51 3075.45 3079.31 3086.38 3098.40 3113.23 3120.66 3134.99
**Nonanal-1,4-rad**	$CH_3CH_2CH_2CH_2CH_2CH_2CH_2C{\bullet}HC(=O)OOH$	-577.7446294	22.76 29.39 62.18 63.82 96.25 113.91 144.32 166.45 185.65 242.27 251.06 257.63 334.41

		367.38 384.08 417.44
		468.23 500.90 512.30
		621.67 711.52 738.26
		757.18 769.21 811.44
		852.15 892.00 913.16
		927.55 985.50 999.76
		1018.02 1047.29 1070.81
		1088.26 1091.41 1118.32
		1130.89 1159.82 1189.50
		1227.47 1240.98 1269.35
		1289.50 1317.79 1342.67
		1344.53 1349.33 1367.62
		1389.72 1408.81 1420.61
		1427.03 1463.02 1488.69
		1492.64 1495.23 1498.86
		1503.33 1505.51 1508.98
		1517.03 1532.85 1746.85
		3017.85 3021.27 3030.17
		3035.68 3036.10 3041.01
		3046.73 3048.41 3064.81
		3073.47 3086.14 3092.31
		3110.82 3119.23 3125.26
		3205.14 3539.70
**Nonanal-1,5-TS**	-577.7083104	**-1683.96** 26.95 37.57
		52.43 75.42 98.00 113.57
		144.64 164.33 194.77
		235.15 244.05 284.87
		332.33 431.94 471.12
		483.89 520.18 555.33
		590.52 704.29 732.22
		740.51 773.65 794.62
		864.05 894.70 916.08
		940.94 1009.12 1021.88
		1041.66 1061.22 1069.80
		1076.50 1085.42 1090.11
		1143.59 1159.99 1192.59
		1221.00 1228.97 1238.31
		1266.14 1285.96 1305.04
		1321.48 1335.66 1343.82
		1351.64 1359.39 1400.99
		1421.19 1423.69 1429.44
		1457.84 1474.69 1493.09
		1493.78 1500.39 1505.67
		1509.27 1516.84 1585.68
		1873.69 3001.16 3021.02
		3024.80 3032.50 3037.25

			3042.13 3046.44 3053.26 3061.56 3075.20 3076.01 3086.90 3112.05 3120.31 3122.09 3130.48
**Nonanal-1,5-rad**	$CH_3CH_2CH_2CH_2CH_2CH_2C$•$HCH_2C(=O)OOH$	-577.7370236	27.14 36.37 65.37 77.82 123.13 132.01 148.38 169.49 225.51 235.23 254.28 274.56 317.36 348.76 404.54 413.92 432.61 449.64 481.51 512.03 641.61 736.41 771.32 786.74 829.79 848.92 893.09 915.06 926.50 962.84 983.09 1003.37 1030.57 1064.33 1079.15 1096.56 1118.86 1129.09 1152.81 1179.99 1221.73 1240.57 1280.96 1298.45 1309.16 1325.01 1335.30 1341.45 1346.49 1387.96 1394.05 1419.84 1422.08 1426.16 1470.39 1485.22 1492.84 1497.56 1500.66 1504.19 1505.33 1511.97 1520.81 1817.70 2985.94 3024.12 3032.89 3036.83 3040.70 3041.93 3057.86 3059.27 3065.53 3070.39 3084.64 3088.24 3112.43 3120.74 3128.59 3195.10 3554.66
**Nonanal-1,6-TS**		-577.710426	**-1757.36** 30.34 37.14 44.98 107.89 119.72 159.57 212.81 226.65 248.08 278.43 295.19 305.96 370.54 401.02 433.26 502.28 512.97 556.82 583.15 733.03 737.16 769.23 783.00 846.41 860.63 892.04 902.97 937.14 959.72 1011.45 1050.09 1055.44 1062.50 1076.30 1090.84 1124.21 1137.03 1143.56 1172.24 1192.88 1234.17 1254.59 1263.11 1291.90

			1309.10 1320.00 1338.15 1352.87 1366.31 1380.89 1398.43 1413.25 1420.82 1420.87 1450.60 1479.26 1482.57 1495.84 1496.34 1498.12 1505.04 1507.91 1516.74 1872.70 3003.80 3032.06 3037.58 3042.21 3044.39 3048.20 3061.12 3066.64 3076.19 3084.95 3089.72 3093.33 3104.27 3115.43 3123.76 3161.66
**Nonanal-1,6-rad**	$CH_3CH_2CH_2CH_2CH_2C{\bullet}HCH_2CH_2C(=O)OOH$	-577.7392926	31.30 48.15 63.66 86.54 109.01 114.32 153.13 171.23 195.69 226.91 248.97 274.48 326.07 344.40 385.07 403.90 431.64 460.78 488.01 527.13 631.77 723.73 737.35 784.50 840.63 866.32 905.55 922.77 927.49 957.84 984.23 1002.28 1043.78 1053.30 1061.75 1086.79 1122.82 1134.82 1148.87 1194.59 1216.46 1242.54 1247.16 1285.76 1308.22 1318.36 1339.09 1340.14 1361.57 1382.90 1393.75 1416.76 1420.99 1433.07 1477.48 1484.92 1488.91 1493.37 1498.36 1505.47 1507.43 1518.42 1523.42 1820.02 2998.23 3006.66 3022.62 3032.69 3041.13 3042.01 3053.06 3068.39 3076.08 3077.56 3086.41 3090.75 3112.66 3119.64 3134.56 3180.07 3558.36
**Nonanal-1,7-TS**		-577.7129331	**-1765.73** 37.79 59.90 65.48 101.77 120.73 154.12 176.39 214.12 246.54 247.37 272.31 334.11 374.51 393.27 451.63 509.27 562.17 580.90 588.47 729.48

			734.76 753.73 777.56
			819.16 828.53 882.15
			912.46 939.37 969.88
			1010.99 1017.99 1051.82
			1079.78 1085.33 1093.02
			1108.49 1139.03 1147.18
			1175.36 1204.56 1231.13
			1240.33 1266.91 1281.88
			1310.53 1327.28 1342.68
			1346.98 1368.78 1385.73
			1398.19 1410.90 1423.18
			1425.50 1462.04 1475.99
			1484.26 1495.30 1500.42
			1503.01 1505.74 1508.87
			1515.01 1869.36 2997.74
			3020.12 3029.41 3036.84
			3044.29 3055.44 3063.55
			3068.56 3076.43 3084.64
			3099.18 3109.64 3114.77
			3115.42 3122.73 3169.18
**Nonanal-1,7-rad**	$CH_3CH_2CH_2CH_2C$•$HCH_2CH_2CH_2C(=O)OOH$	-577.7387381	29.97 56.05 71.24 77.16
			103.30 105.92 149.29
			182.10 230.17 242.04
			253.39 260.05 299.57
			311.50 353.54 371.85
			439.19 469.69 488.12
			563.72 618.25 724.96
			758.18 815.01 820.93
			864.78 878.21 931.40
			940.27 943.97 985.18
			1010.32 1042.59 1061.72
			1073.65 1090.02 1104.48
			1135.96 1158.75 1188.88
			1227.47 1237.68 1249.70
			1284.33 1303.19 1331.16
			1339.54 1352.82 1366.48
			1385.83 1404.14 1413.86
			1421.11 1436.36 1468.85
			1476.16 1487.88 1496.42
			1497.24 1503.48 1505.49
			1522.11 1528.95 1815.30
			2977.37 2995.28 3004.38
			3031.34 3039.92 3043.33
			3064.50 3064.77 3070.23
			3076.28 3086.14 3111.27
			3111.73 3118.80 3126.59

			3188.73 3564.07
**Nonanal-1,8-TS**		-577.712077	**-1687.27** 37.63 65.50 69.48 88.74 105.42 144.18 165.21 232.89 243.98 255.48 296.70 343.91 369.33 394.57 461.09 472.91 518.78 559.32 593.83 694.13 741.94 774.54 782.30 841.60 873.84 884.14 914.34 945.95 988.55 1003.67 1037.14 1062.61 1088.64 1092.65 1096.82 1108.62 1132.00 1135.01 1169.28 1170.72 1224.07 1248.39 1273.04 1279.27 1295.16 1318.63 1340.72 1350.82 1382.61 1393.51 1398.37 1410.01 1423.71 1424.83 1448.16 1472.74 1483.74 1489.53 1500.23 1500.91 1502.05 1506.40 1513.66 1861.76 2997.28 3017.06 3040.57 3043.72 3045.45 3054.71 3055.24 3061.64 3078.93 3085.93 3091.56 3095.70 3103.18 3115.11 3124.54 3164.44
**Nonanal-1,8-rad**	$CH_3CH_2CH_2C{\bullet}HCH_2CH_2CH_2CH_2C(=O)OOH$	-577.7380125	20.91 29.75 44.09 51.18 86.78 130.73 164.11 204.84 217.29 227.69 246.37 282.63 305.87 329.48 378.83 413.01 452.79 472.97 494.91 583.39 625.57 700.56 779.11 786.88 836.24 842.44 881.62 909.43 932.65 953.08 987.91 1013.80 1051.48 1067.46 1078.85 1083.72 1089.97 1133.14 1163.53 1181.65 1213.88 1246.29 1255.53 1279.96 1294.55 1326.15 1339.10 1351.80 1378.25 1386.77 1403.90 1415.78 1419.86 1436.62 1473.44

			1476.30 1477.00 1494.95 1499.04 1504.05 1507.95 1514.13 1524.79 1815.63 2954.12 2960.48 3037.91 3039.61 3044.51 3053.01 3059.89 3061.28 3070.39 3080.24 3082.87 3110.80 3111.46 3118.72 3125.44 3166.59 3559.23
**Nonanal-1,9-TS**		-577.7079898	**-1776.02** 49.77 72.65 85.44 131.30 139.97 176.62 187.84 217.04 242.27 274.57 336.00 365.10 377.45 385.91 441.28 460.64 512.73 577.86 600.85 699.06 754.61 766.37 779.73 799.91 856.63 882.23 896.98 950.10 986.54 993.77 1031.98 1046.10 1073.80 1082.75 1092.77 1106.96 1137.05 1152.93 1162.85 1193.39 1208.82 1234.15 1266.99 1289.24 1306.81 1329.67 1337.50 1375.17 1386.34 1394.52 1406.36 1417.06 1426.11 1428.61 1452.22 1476.65 1479.57 1491.36 1503.10 1504.14 1507.22 1507.90 1510.64 1865.74 3001.79 3010.73 3043.50 3047.29 3051.46 3056.69 3065.60 3073.28 3084.59 3087.40 3093.03 3100.98 3107.46 3124.77 3134.62 3198.23
**Nonanal-1,9-rad**	$CH_3CH_2C{\bullet}HCH_2CH_2CH_2CH_2CH_2C(=O)OOH$	-577.7373221	34.54 36.49 57.99 62.58 81.39 92.49 162.18 202.66 225.21 228.65 251.88 295.95 328.94 338.17 368.80 427.40 445.18 457.82 484.86 531.66 638.26 719.28 765.46 786.51 821.76 844.49 873.57 912.43 952.00 970.60 990.45 1023.33

1048.92 1058.42 1061.12
1084.65 1095.97 1130.09
1158.38 1175.54 1201.09
1234.11 1279.42 1289.84
1292.71 1316.07 1341.19
1349.57 1369.56 1388.50
1394.14 1416.10 1419.58
1437.64 1480.39 1485.07
1492.06 1493.60 1500.18
1501.77 1502.79 1506.65
1512.30 1817.90 2962.38
3023.26 3035.65 3038.87
3043.53 3061.54 3065.13
3068.53 3074.55 3080.11
3087.28 3111.34 3114.73
3120.78 3135.49 3157.64
3555.62

10. Cartesian coordinates of the global minimum geometry of each of the species involved

Species		Coordinates		
**Ethanal-AcOO**	C	-1.441707000	0.186549000	0.000000000
	H	-1.672779000	-0.417565000	0.874873000
	H	-2.020645000	1.103483000	0.000000000
	H	-1.672779000	-0.417565000	-0.874873000
	C	0.000000000	0.558925000	0.000000000
	O	0.491666000	1.638754000	0.000000000
	O	0.916874000	-0.528970000	0.000000000
	O	0.343516000	-1.702434000	0.000000000
**Ethanal-1,4-TS**	C	0.000000000	0.583442000	0.000000000
	O	0.244666000	1.747616000	0.000000000
	C	-1.350373000	-0.066108000	0.000000000
	O	0.973883000	-0.366911000	0.000000000
	O	0.374846000	-1.632600000	0.000000000
	H	-1.922334000	0.076256000	0.909061000
	H	-0.800250000	-1.241358000	0.000000000
	H	-1.922334000	0.076256000	-0.909061000
**Ethanal-1,4-rad**	C	-0.473923000	0.132072000	-0.000057000
	O	-0.209785000	1.320234000	-0.000002000
	C	-1.777775000	-0.467027000	0.000033000
	O	0.504537000	-0.801697000	-0.000047000
	H	-1.884497000	-1.537757000	0.000145000
	H	-2.635330000	0.181296000	0.000004000
	O	1.769181000	-0.192974000	0.000029000
	H	1.518548000	0.761695000	0.000158000
**Propanal-AcOO**	C	-2.065742000	-0.896490000	0.000000000
	H	-2.429023000	-1.922372000	0.000000000
	H	-2.460857000	-0.390792000	0.878971000
	H	-2.460857000	-0.390792000	-0.878971000
	C	-0.545728000	-0.888667000	0.000000000
	H	-0.135805000	-1.410024000	0.865943000
	H	-0.135805000	-1.410024000	-0.865943000
	C	0.000000000	0.504177000	0.000000000
	O	-0.586155000	1.530979000	0.000000000
	O	1.427433000	0.628193000	0.000000000
	O	2.070117000	-0.507937000	0.000000000
**Propanal-1,4-TS**	C	0.273710000	0.650626000	0.083502000
	O	0.145549000	1.827929000	-0.012879000
	C	-0.761926000	-0.352274000	0.524879000
	O	1.438266000	0.022386000	-0.216026000

	O	1.268616000	-1.362571000	-0.040690000
	H	-0.930195000	-0.315651000	1.599266000
	H	0.050674000	-1.310465000	0.329910000
	C	-2.005140000	-0.447497000	-0.311085000
	H	-2.563130000	0.489297000	-0.242028000
	H	-1.768301000	-0.620723000	-1.359509000
	H	-2.648368000	-1.249536000	0.045347000
**Propanal-1,4-rad**	C	-0.058002000	0.066469000	-0.000144000
	O	-0.219161000	1.271229000	-0.000251000
	C	1.187978000	-0.647388000	-0.000004000
	O	-1.113721000	-0.783641000	0.000086000
	H	1.146638000	-1.727506000	0.000194000
	C	2.482763000	0.060148000	0.000080000
	H	3.074318000	-0.221806000	0.875729000
	H	3.075582000	-0.223822000	-0.874031000
	H	2.343236000	1.138285000	-0.001166000
	O	-2.330746000	-0.074036000	0.000113000
	H	-2.007196000	0.851050000	0.000088000
**Propanal-1,5-TS**	C	-0.822449000	-0.142568000	0.030371000
	O	-1.975823000	-0.391386000	-0.120103000
	C	0.274903000	-1.137140000	0.364581000
	H	0.420663000	-1.110569000	1.445302000
	H	-0.121657000	-2.116471000	0.099894000
	C	1.560440000	-0.816210000	-0.342249000
	O	-0.405446000	1.146366000	-0.182735000
	O	0.889257000	1.419991000	0.251084000
	H	2.476307000	-1.126164000	0.148068000
	H	1.519795000	0.482030000	-0.175904000
	H	1.563629000	-0.953083000	-1.419544000
**Propanal-1,5-rad**	C	0.031465000	0.083762000	0.065888000
	O	0.190152000	1.275561000	0.113709000
	C	-1.267891000	-0.668025000	0.169634000
	H	-1.197615000	-1.583896000	-0.421097000
	H	-1.333026000	-1.007878000	1.213990000
	C	-2.445253000	0.150905000	-0.200434000
	O	1.061810000	-0.765450000	-0.064251000
	O	2.297616000	-0.079784000	-0.098100000
	H	-2.369692000	1.225591000	-0.249448000
	H	1.998096000	0.849842000	-0.038098000
	H	-3.404310000	-0.326136000	-0.326733000
**Butanal-AcOO**	C	-1.561744000	2.519026000	0.000000000
	H	-2.604241000	2.833907000	0.000000000
	H	-1.083206000	2.949383000	0.881054000

	H	-1.083206000	2.949383000	-0.881054000
	C	-1.452454000	1.000379000	0.000000000
	H	-1.960128000	0.586731000	0.871799000
	H	-1.960128000	0.586731000	-0.871799000
	C	0.000000000	0.543723000	0.000000000
	H	0.541305000	0.929281000	-0.866332000
	H	0.541305000	0.929281000	0.866332000
	C	0.122519000	-0.946049000	0.000000000
	O	-0.732495000	-1.762655000	0.000000000
	O	1.455380000	-1.474761000	0.000000000
	O	2.396912000	-0.570979000	0.000000000
**Butanal-1,4-TS**	C	1.016152000	0.546451000	0.090614000
	O	1.382656000	1.674176000	0.012302000
	C	-0.346013000	0.049582000	0.497816000
	O	1.828092000	-0.499341000	-0.207259000
	O	1.111623000	-1.697599000	-0.035735000
	H	-0.534665000	0.176973000	1.562771000
	H	0.018092000	-1.158430000	0.335265000
	C	-1.497772000	0.422292000	-0.395656000
	H	-1.597566000	1.511851000	-0.366405000
	H	-1.256445000	0.163892000	-1.427988000
	C	-2.804302000	-0.235583000	0.029243000
	H	-2.728817000	-1.322217000	-0.015768000
	H	-3.069130000	0.042099000	1.050097000
	H	-3.618821000	0.071494000	-0.624540000
**Butanal-1,4-rad**	C	0.604381000	0.113777000	-0.078897000
	O	0.847616000	1.293984000	0.082231000
	C	-0.676224000	-0.489746000	-0.317447000
	O	1.590168000	-0.816504000	-0.045280000
	H	-0.711374000	-1.565372000	-0.430154000
	C	-1.914498000	0.313001000	-0.414158000
	H	-1.695141000	1.349544000	-0.161866000
	H	-2.248374000	0.307348000	-1.458968000
	C	-3.043008000	-0.237359000	0.462878000
	H	-3.275265000	-1.270765000	0.203987000
	H	-2.767269000	-0.206386000	1.516316000
	H	-3.947293000	0.354864000	0.330931000
	O	2.841756000	-0.214501000	0.191512000
	H	2.584494000	0.728903000	0.257786000
**Butanal-1,5-TS**	C	-1.026384000	-0.389335000	0.029845000
	O	-1.865319000	-1.153816000	-0.328541000
	C	0.143233000	-0.697869000	0.944327000
	H	-0.128525000	-0.370473000	1.948551000
	H	0.243025000	-1.783099000	0.952230000
	C	1.402474000	-0.001358000	0.490475000

	C	2.030001000	-0.483853000	-0.785841000
	H	2.417269000	-1.498751000	-0.653071000
	H	1.304719000	-0.515976000	-1.600144000
	O	-1.049265000	0.882803000	-0.481374000
	O	-0.203569000	1.781717000	0.162338000
	H	2.100553000	0.224756000	1.292481000
	H	0.853552000	1.139040000	0.255751000
	H	2.858687000	0.153369000	-1.088015000
**Butanal-1,5-rad**	C	0.488277000	0.042843000	0.364443000
	O	0.580958000	1.239245000	0.476282000
	C	-0.714871000	-0.805696000	0.702382000
	H	-0.404351000	-1.844390000	0.800000000
	H	-1.085391000	-0.448332000	1.664992000
	C	-1.752923000	-0.644086000	-0.359795000
	C	-2.626159000	0.552480000	-0.391028000
	H	-3.406497000	0.460031000	-1.144021000
	H	-3.098649000	0.723913000	0.580507000
	O	1.479795000	-0.701455000	-0.147908000
	H	-1.700432000	-1.300402000	-1.217178000
	H	-2.051674000	1.460936000	-0.613227000
	O	2.579163000	0.098064000	-0.538770000
	H	2.261723000	0.988158000	-0.283910000
**Butanal-1,6-TS**	C	-1.107266000	-0.186294000	0.036439000
	O	-2.151752000	-0.622031000	-0.338675000
	C	-0.013537000	-0.979569000	0.703821000
	H	0.284656000	-0.507797000	1.639048000
	H	-0.442201000	-1.954033000	0.919128000
	C	1.206256000	-1.129776000	-0.232358000
	H	0.857923000	-1.421923000	-1.222808000
	H	1.813382000	-1.956549000	0.143780000
	C	2.057515000	0.111355000	-0.316200000
	O	-0.836757000	1.125599000	-0.275854000
	O	0.261393000	1.652072000	0.391975000
	H	2.744682000	0.262416000	0.512668000
	H	1.209612000	1.090876000	-0.078954000
	H	2.491063000	0.347596000	-1.282640000
**Butanal-1,6-rad**	C	0.589404000	0.109965000	0.116190000
	O	0.843454000	1.284318000	0.206404000
	C	-0.758280000	-0.537267000	0.242670000
	H	-0.750140000	-1.492080000	-0.280963000
	H	-0.898807000	-0.758997000	1.304104000
	C	-1.885216000	0.365544000	-0.253764000
	H	-1.735256000	0.593417000	-1.312747000
	H	-1.806276000	1.332582000	0.258855000
	C	-3.226133000	-0.229683000	-0.041973000

	O	1.547130000	-0.805230000	-0.099651000
	O	2.827902000	-0.210406000	-0.164565000
	H	-3.392544000	-0.965897000	0.732231000
	H	2.601412000	0.734427000	-0.046996000
	H	-4.084929000	0.155741000	-0.570715000
**Pentanal-AcOO**	C	-3.366360000	-1.502871000	0.000000000
	H	-4.404385000	-1.171800000	0.000000000
	H	-3.214448000	-2.128789000	0.880707000
	H	-3.214448000	-2.128789000	-0.880707000
	C	-2.407713000	-0.320001000	0.000000000
	H	-2.599186000	0.307646000	-0.874578000
	H	-2.599186000	0.307646000	0.874578000
	C	-0.947000000	-0.754115000	0.000000000
	H	-0.746266000	-1.378855000	-0.872491000
	H	-0.746266000	-1.378855000	0.872491000
	C	0.000000000	0.436707000	0.000000000
	H	-0.164439000	1.080664000	0.866330000
	H	-0.164439000	1.080664000	-0.866330000
	C	1.434237000	0.016059000	0.000000000
	O	1.890196000	-1.075025000	0.000000000
	O	2.406140000	1.070605000	0.000000000
	O	1.900425000	2.273894000	0.000000000
**Pentanal-1,4-TS**	C	1.538458000	0.561188000	0.058368000
	O	1.864186000	1.699620000	-0.042328000
	C	0.242545000	0.021243000	0.603174000
	O	2.341105000	-0.457612000	-0.341309000
	O	1.680825000	-1.677691000	-0.109140000
	H	0.165438000	0.134854000	1.683451000
	H	0.620242000	-1.173657000	0.388225000
	C	-1.007301000	0.366019000	-0.158254000
	H	-1.138201000	1.452787000	-0.110087000
	H	-0.874201000	0.120276000	-1.214320000
	C	-2.248789000	-0.328271000	0.392604000
	H	-2.101701000	-1.410099000	0.355608000
	H	-2.367164000	-0.067111000	1.447164000
	C	-3.508101000	0.046196000	-0.376209000
	H	-4.382527000	-0.459143000	0.031755000
	H	-3.689991000	1.120920000	-0.330081000
	H	-3.421692000	-0.231609000	-1.427601000
**Pentanal-1,4-rad**	C	0.924169000	-0.195883000	0.164158000
	O	1.049269000	0.438813000	1.193579000
	C	-0.235420000	-0.914005000	-0.283376000
	O	1.940609000	-0.292553000	-0.728069000
	O	3.072145000	0.417716000	-0.280613000

	H	-0.179490000	-1.406213000	-1.245305000
	H	2.744922000	0.768872000	0.573830000
	C	-1.490167000	-0.938780000	0.498272000
	H	-1.797996000	-1.979878000	0.634370000
	H	-1.312800000	-0.510953000	1.484964000
	C	-2.635104000	-0.179507000	-0.198848000
	H	-2.817778000	-0.623945000	-1.179882000
	H	-3.543351000	-0.331511000	0.386212000
	C	-2.354683000	1.309961000	-0.344923000
	H	-1.480352000	1.489976000	-0.972357000
	H	-3.200983000	1.824245000	-0.799021000
	H	-2.161133000	1.766890000	0.626315000
**Pentanal-1,5-TS**	C	-1.496941000	-0.299314000	-0.128694000
	O	-2.322489000	-0.929553000	-0.710282000
	C	-0.572494000	-0.813085000	0.958231000
	H	-1.007834000	-0.536743000	1.919271000
	H	-0.585588000	-1.899799000	0.876549000
	C	0.814443000	-0.236157000	0.826538000
	C	1.644941000	-0.681881000	-0.345219000
	H	1.876304000	-1.745372000	-0.210584000
	H	1.050664000	-0.619570000	-1.260516000
	C	2.933427000	0.113964000	-0.503117000
	H	3.550558000	0.043170000	0.393669000
	H	2.717156000	1.167785000	-0.680274000
	O	-1.285671000	0.999543000	-0.513901000
	O	-0.499851000	1.745044000	0.360883000
	H	1.358738000	-0.151530000	1.765310000
	H	0.444342000	0.975469000	0.599916000
	H	3.519493000	-0.254841000	-1.343374000
**Pentanal-1,5-rad**	C	1.059070000	0.112695000	0.383158000
	O	1.107986000	-0.959505000	0.931413000
	C	-0.020386000	1.157880000	0.536148000
	H	-0.242017000	1.221830000	1.602956000
	H	0.359336000	2.117698000	0.190158000
	C	-1.229612000	0.739460000	-0.234037000
	C	-2.189812000	-0.243715000	0.325979000
	H	-1.662951000	-1.184004000	0.539873000
	H	-2.535629000	0.107842000	1.305884000
	C	-3.381553000	-0.516152000	-0.582879000
	H	-3.945704000	0.397503000	-0.772772000
	H	-4.057782000	-1.242899000	-0.134703000
	O	1.982910000	0.495596000	-0.511043000
	H	-1.281743000	1.007410000	-1.281524000
	H	-3.053277000	-0.912906000	-1.544298000
	O	2.953324000	-0.512498000	-0.717598000

	H	2.639773000	-1.202231000	-0.097966000
**Pentanal-1,6-TS**	C	-1.495809000	-0.099302000	-0.128717000
	O	-2.437841000	-0.402227000	-0.794551000
	C	-0.687425000	-1.040211000	0.727407000
	H	-0.597769000	-0.653997000	1.741728000
	H	-1.243623000	-1.972882000	0.751369000
	C	0.710068000	-1.266175000	0.114912000
	H	0.602024000	-1.456452000	-0.954591000
	H	1.128878000	-2.175841000	0.552279000
	C	1.672311000	-0.120177000	0.341143000
	C	2.657565000	0.182307000	-0.750662000
	H	3.328625000	-0.670012000	-0.899535000
	H	3.269907000	1.049835000	-0.511640000
	O	-1.039819000	1.191711000	-0.251056000
	O	-0.117849000	1.559959000	0.718544000
	H	2.099488000	-0.115997000	1.344351000
	H	0.887946000	0.895484000	0.465393000
	H	2.148346000	0.365660000	-1.697356000
**Pentanal-1,6-rad**	C	-0.984876000	0.369388000	0.203177000
	O	-1.114036000	0.036715000	1.354101000
	C	0.028410000	1.332064000	-0.344394000
	H	-0.050499000	1.365055000	-1.430011000
	H	-0.234648000	2.318024000	0.041596000
	C	1.442668000	0.950124000	0.094251000
	H	1.484406000	0.921754000	1.186843000
	H	2.120312000	1.761100000	-0.207723000
	C	1.898414000	-0.348860000	-0.466437000
	C	3.074164000	-1.050683000	0.101923000
	H	2.990317000	-1.146760000	1.188503000
	H	4.006137000	-0.500143000	-0.086210000
	O	-1.786193000	-0.105375000	-0.763155000
	O	-2.753455000	-0.994370000	-0.241613000
	H	1.476909000	-0.685408000	-1.405762000
	H	3.195281000	-2.046764000	-0.320159000
	H	-2.511425000	-0.994822000	0.707143000
**Pentanal-1,7-TS**	C	0.771040000	-1.507056000	0.264176000
	H	1.455106000	-1.876485000	1.030030000
	H	0.400548000	-2.376839000	-0.278273000
	C	1.531453000	-0.614505000	-0.718671000
	H	0.897717000	-0.380871000	-1.578445000
	C	2.063385000	0.660214000	-0.132447000
	H	2.578673000	1.320733000	-0.823772000
	C	-0.416464000	-0.831379000	0.979094000
	H	-1.031920000	-1.593052000	1.451172000
	H	-0.068651000	-0.139535000	1.739937000

	C	-1.295020000	-0.126275000	-0.018053000
	O	-2.183144000	-0.626700000	-0.637228000
	O	-0.996552000	1.183332000	-0.333646000
	H	2.371880000	-1.187094000	-1.126771000
	O	-0.080959000	1.794530000	0.511437000
	H	0.989632000	1.366711000	0.188205000
	H	2.565884000	0.571135000	0.828829000
**Pentanal-1,7-rad**	C	1.556315000	-0.989503000	0.196225000
	H	2.350273000	-1.258388000	0.895471000
	H	1.320832000	-1.882058000	-0.382773000
	C	2.055687000	0.113940000	-0.738634000
	H	1.273366000	0.374939000	-1.456829000
	C	2.530153000	1.329315000	-0.031632000
	H	3.021525000	1.246525000	0.929248000
	C	0.327765000	-0.581218000	1.005902000
	H	0.064134000	-1.368847000	1.715097000
	H	0.511249000	0.326416000	1.579760000
	C	-0.879032000	-0.378660000	0.136097000
	O	-1.204857000	-1.041887000	-0.816831000
	O	-1.616901000	0.660345000	0.555333000
	H	2.870462000	-0.303720000	-1.347055000
	O	-2.780046000	0.820429000	-0.231834000
	H	-2.666401000	0.095435000	-0.879990000
	H	2.523664000	2.295359000	-0.514018000
**Hexanal-AcOO**	C	-3.193143000	2.987216000	0.000000000
	H	-3.336055000	4.067432000	0.000000000
	H	-3.695744000	2.583990000	0.880737000
	H	-3.695744000	2.583990000	-0.880737000
	C	-1.714936000	2.621449000	0.000000000
	H	-1.227489000	3.060911000	0.874111000
	H	-1.227489000	3.060911000	-0.874111000
	C	-1.477911000	1.116145000	0.000000000
	H	-1.964980000	0.674237000	0.874975000
	H	-1.964980000	0.674237000	-0.874975000
	C	0.000000000	0.747098000	0.000000000
	H	0.490993000	1.182042000	-0.872396000
	H	0.490993000	1.182042000	0.872396000
	C	0.209251000	-0.760173000	0.000000000
	H	-0.260360000	-1.230390000	0.866221000
	H	-0.260360000	-1.230390000	-0.866221000
	C	1.657628000	-1.128832000	0.000000000
	O	2.605809000	-0.422566000	0.000000000
	O	1.956557000	-2.531626000	0.000000000
	O	0.908367000	-3.309111000	0.000000000

**Hexanal-1,4-TS**	C	1.633960000	-0.262020000	-0.078668000
	O	2.196212000	-1.169349000	-0.601553000
	C	0.610453000	-0.323579000	1.024727000
	O	1.862125000	1.028765000	-0.433857000
	O	1.006107000	1.864182000	0.304126000
	H	1.091616000	-0.400921000	1.998690000
	H	0.385368000	0.925720000	0.910566000
	C	-0.572876000	-1.235511000	0.850279000
	H	-0.212257000	-2.258645000	1.000317000
	H	-1.270512000	-1.046014000	1.668020000
	C	-1.281523000	-1.151393000	-0.500584000
	H	-0.597079000	-1.486352000	-1.282627000
	H	-2.104329000	-1.869870000	-0.485513000
	C	-1.828276000	0.229071000	-0.865285000
	H	-1.004931000	0.928051000	-1.025866000
	H	-2.339522000	0.144577000	-1.825639000
	C	-2.787201000	0.808556000	0.167533000
	H	-3.605660000	0.116986000	0.377724000
	H	-2.284467000	1.023691000	1.112130000
	H	-3.221001000	1.743248000	-0.185536000
**Hexanal-1,4-rad**	C	1.228654000	-0.205980000	0.203841000
	O	1.225750000	0.686980000	1.029921000
	C	0.198553000	-1.175495000	-0.037787000
	O	2.281668000	-0.383456000	-0.631061000
	O	3.284702000	0.575417000	-0.387132000
	H	0.360408000	-1.888943000	-0.835069000
	H	2.879514000	1.085648000	0.345012000
	C	-1.067945000	-1.180596000	0.724441000
	H	-1.203361000	-2.170314000	1.171787000
	H	-1.006846000	-0.460916000	1.539195000
	C	-2.291644000	-0.889100000	-0.165382000
	H	-2.371344000	-1.670568000	-0.924648000
	H	-3.185359000	-0.966018000	0.458164000
	C	-2.259545000	0.477889000	-0.845794000
	H	-1.389044000	0.534702000	-1.505661000
	H	-3.135194000	0.557363000	-1.492932000
	C	-2.237946000	1.652439000	0.125586000
	H	-1.320492000	1.672843000	0.713788000
	H	-2.304505000	2.599042000	-0.409886000
	H	-3.081503000	1.600692000	0.816992000
**Hexanal-1,5-TS**	C	-1.893790000	-0.404622000	-0.227985000
	O	-2.552360000	-1.209049000	-0.807831000
	C	-1.181726000	-0.609024000	1.095231000
	H	-1.814279000	-0.189617000	1.878496000
	H	-1.123408000	-1.687208000	1.243015000
	C	0.172754000	0.053711000	1.104679000

	C	1.240232000	-0.534212000	0.225181000
	H	1.502057000	-1.524740000	0.619758000
	H	0.838002000	-0.710402000	-0.777122000
	C	2.495193000	0.326234000	0.132035000
	H	2.888241000	0.498666000	1.137375000
	H	2.223236000	1.306224000	-0.266643000
	O	-1.675959000	0.807432000	-0.830949000
	O	-1.120671000	1.778127000	-0.001530000
	H	0.513847000	0.368054000	2.089249000
	H	-0.205564000	1.158842000	0.566050000
	C	3.571726000	-0.303655000	-0.740595000
	H	3.208514000	-0.458344000	-1.757617000
	H	4.456328000	0.329749000	-0.794939000
	H	3.878607000	-1.273888000	-0.346421000
**Hexanal-1,5-rad**	C	1.182166000	-0.510110000	0.292476000
	O	1.199883000	-0.008924000	1.388278000
	C	0.363879000	-1.701687000	-0.145222000
	H	0.893939000	-2.234296000	-0.932176000
	H	0.273943000	-2.348290000	0.729775000
	C	-0.974650000	-1.233315000	-0.614648000
	C	-2.006163000	-0.709201000	0.318036000
	H	-2.841277000	-1.419556000	0.375230000
	H	-1.592264000	-0.640726000	1.327469000
	C	-2.562223000	0.659128000	-0.098426000
	H	-3.377115000	0.925073000	0.577473000
	H	-3.001930000	0.575853000	-1.095703000
	O	1.880445000	0.001474000	-0.732973000
	H	-1.142844000	-1.157243000	-1.681169000
	C	-1.506345000	1.757132000	-0.090476000
	H	-1.051735000	1.852533000	0.896641000
	H	-0.706663000	1.542919000	-0.802124000
	H	-1.937625000	2.720964000	-0.359482000
	O	2.567833000	1.178395000	-0.354781000
	H	2.318303000	1.243524000	0.589424000
**Hexanal-1,6-TS**	C	1.882031000	-0.147797000	-0.184359000
	O	2.800408000	-0.113215000	-0.944786000
	C	1.377249000	1.011404000	0.635748000
	H	1.283075000	0.727924000	1.682758000
	H	2.128321000	1.790843000	0.544615000
	C	0.022924000	1.513223000	0.092244000
	H	0.103432000	1.636609000	-0.989408000
	H	-0.158332000	2.506557000	0.508266000
	C	-1.147657000	0.610896000	0.421747000
	C	-2.179500000	0.383831000	-0.648434000
	H	-1.679912000	0.031325000	-1.554444000
	H	-2.620422000	1.354400000	-0.908431000

	O	1.153747000	-1.312983000	-0.145461000
	O	0.256554000	-1.383829000	0.911159000
	H	-1.569116000	0.792833000	1.412021000
	H	-0.593814000	-0.527192000	0.650583000
	C	-3.274601000	-0.590214000	-0.237137000
	H	-3.802875000	-0.234687000	0.648684000
	H	-4.005357000	-0.717396000	-1.034467000
	H	-2.853349000	-1.569060000	-0.006314000
**Hexanal-1,6-rad**	C	-1.478951000	0.318188000	0.159405000
	O	-1.549901000	0.077280000	1.338001000
	C	-0.732586000	1.451922000	-0.481746000
	H	-0.771023000	1.345715000	-1.564878000
	H	-1.262237000	2.368041000	-0.215647000
	C	0.712534000	1.508179000	0.016293000
	H	0.713177000	1.614563000	1.104333000
	H	1.167408000	2.428844000	-0.374847000
	C	1.509637000	0.317908000	-0.379440000
	C	2.756388000	-0.049363000	0.337785000
	H	2.554119000	-0.086773000	1.414755000
	H	3.501816000	0.751468000	0.220410000
	O	-2.104919000	-0.438927000	-0.754941000
	H	1.266476000	-0.186914000	-1.308377000
	C	3.359983000	-1.370584000	-0.123067000
	H	4.272789000	-1.600023000	0.425600000
	H	2.658317000	-2.190972000	0.029792000
	H	3.606850000	-1.335729000	-1.185064000
	O	-2.826492000	-1.491848000	-0.147810000
	H	-2.619227000	-1.337754000	0.796545000
**Hexanal-1,7-TS**	C	0.419071000	1.675348000	0.177771000
	H	0.528040000	2.747147000	0.012357000
	H	0.345610000	1.545815000	1.259017000
	C	1.679286000	0.998405000	-0.364365000
	H	2.547202000	1.466717000	0.114895000
	C	1.789212000	-0.494129000	-0.161957000
	H	2.624383000	-0.927690000	-0.711395000
	C	-0.908542000	1.245080000	-0.477318000
	H	-1.646655000	2.026737000	-0.312613000
	H	-0.776538000	1.101942000	-1.547081000
	C	-1.513684000	0.020527000	0.161182000
	O	-2.254782000	0.039228000	1.097668000
	C	1.649079000	-1.038035000	1.232909000
	H	1.716246000	-2.124141000	1.241012000
	H	2.451728000	-0.645791000	1.866151000
	H	0.703958000	-0.758058000	1.697702000
	O	-1.178303000	-1.222774000	-0.317882000
	H	1.768748000	1.216261000	-1.430884000

	O	-0.285410000	-1.206213000	-1.375608000
	H	0.798700000	-0.974043000	-0.831928000
**Hexanal-1,7-rad**	C	1.358202000	-1.371831000	0.350536000
	H	1.437529000	-0.951559000	1.354147000
	H	1.898608000	-2.319209000	0.357149000
	C	2.016334000	-0.425473000	-0.671563000
	H	1.939690000	-0.877033000	-1.663202000
	C	1.449342000	0.950632000	-0.689490000
	H	0.766285000	1.232407000	-1.479128000
	C	-0.111290000	-1.663331000	0.044348000
	H	-0.239244000	-1.988929000	-0.987409000
	H	-0.472348000	-2.462206000	0.694481000
	C	-0.999442000	-0.483981000	0.310669000
	O	-1.082889000	0.137416000	1.341432000
	C	1.688535000	1.890687000	0.433737000
	H	1.464761000	2.919302000	0.154121000
	H	1.061651000	1.649979000	1.302433000
	H	2.726955000	1.844696000	0.775403000
	O	-1.767399000	-0.192039000	-0.751458000
	H	3.083449000	-0.376415000	-0.426956000
	O	-2.613665000	0.908854000	-0.488322000
	H	-2.365802000	1.114901000	0.436313000
**Hexanal-1,8-TS**	C	-1.516506000	-0.031659000	-0.072905000
	O	-2.328011000	-0.522146000	-0.797981000
	C	-0.754875000	-0.774349000	0.992426000
	H	-0.227260000	-0.093538000	1.651245000
	H	-1.500988000	-1.308487000	1.579241000
	C	0.198814000	-1.793445000	0.354811000
	H	-0.394938000	-2.529964000	-0.187274000
	H	0.704964000	-2.325350000	1.163002000
	C	1.243332000	-1.211723000	-0.598007000
	C	2.201873000	-0.184738000	0.028783000
	H	3.220438000	-0.385139000	-0.323128000
	H	2.242689000	-0.314842000	1.113275000
	C	1.911937000	1.250975000	-0.305390000
	H	0.737293000	1.598505000	0.172772000
	H	1.758316000	1.460309000	-1.362436000
	O	-1.241036000	1.299197000	-0.294258000
	O	-0.351500000	1.872470000	0.601007000
	H	0.742318000	-0.764773000	-1.461121000
	H	1.821418000	-2.047560000	-0.992688000
	H	2.552681000	1.984301000	0.178654000
**Hexanal-1,8-rad**	C	1.315647000	0.407440000	0.244453000
	O	1.977463000	1.082023000	-0.503863000
	C	0.082936000	0.831533000	0.985263000

	H	-0.283269000	0.003818000	1.589596000
	H	0.384350000	1.630971000	1.663144000
	C	-0.995279000	1.346895000	0.027294000
	H	-0.611178000	2.225291000	-0.491674000
	H	-1.842712000	1.674694000	0.631227000
	C	-1.460840000	0.317892000	-1.001576000
	C	-2.040589000	-0.965776000	-0.402095000
	H	-1.267829000	-1.515102000	0.142991000
	H	-2.315384000	-1.630737000	-1.233628000
	C	-3.220462000	-0.744892000	0.469371000
	H	3.005555000	-0.448252000	-0.684917000
	H	-3.894921000	0.080426000	0.281787000
	O	1.626332000	-0.874955000	0.496014000
	O	2.784042000	-1.275089000	-0.209435000
	H	-0.629995000	0.059708000	-1.662809000
	H	-2.217637000	0.786729000	-1.634640000
	H	-3.518150000	-1.481927000	1.200935000
**Heptanal-AcOO**	C	-5.062371000	0.050865000	0.000000000
	H	-5.151208000	0.689354000	0.880655000
	H	-5.151208000	0.689354000	-0.880655000
	H	-5.907645000	-0.637094000	0.000000000
	C	-3.735382000	-0.696169000	0.000000000
	H	-3.682864000	-1.350598000	0.874416000
	H	-3.682864000	-1.350598000	-0.874416000
	C	-2.530562000	0.236638000	0.000000000
	H	-2.581000000	0.892464000	0.874776000
	H	-2.581000000	0.892464000	-0.874776000
	C	-1.197997000	-0.500841000	0.000000000
	H	-1.147391000	-1.156323000	0.875138000
	H	-1.147391000	-1.156323000	-0.875138000
	C	0.000000000	0.439943000	0.000000000
	H	-0.042127000	1.094579000	0.872477000
	H	-0.042127000	1.094579000	-0.872477000
	C	1.318263000	-0.319829000	0.000000000
	H	1.401511000	-0.979124000	-0.866354000
	H	1.401511000	-0.979124000	0.866354000
	C	2.497676000	0.598299000	0.000000000
	O	2.520857000	1.780599000	0.000000000
	O	3.789367000	-0.025147000	0.000000000
	O	3.761781000	-1.330084000	0.000000000
**Heptanal-1,4-TS**	C	-1.857010000	-0.194584000	0.506138000
	O	-2.117997000	-0.054679000	1.657296000
	C	-1.521076000	0.876387000	-0.499837000
	O	-1.824996000	-1.417395000	-0.082151000

	O	-1.435004000	-1.269287000	-1.424347000
	H	-2.422328000	1.350240000	-0.885742000
	H	-1.246887000	-0.005594000	-1.376014000
	C	-0.416613000	1.838030000	-0.153037000
	H	-0.228089000	2.474355000	-1.020653000
	H	-0.810462000	2.498276000	0.627237000
	C	0.881413000	1.198027000	0.331648000
	H	1.536692000	1.993213000	0.694960000
	H	0.665400000	0.565805000	1.195519000
	C	1.616810000	0.399712000	-0.741282000
	H	0.996573000	-0.436084000	-1.075583000
	H	1.769344000	1.041783000	-1.613795000
	C	2.966285000	-0.147143000	-0.280505000
	H	3.469835000	-0.596163000	-1.138812000
	H	3.599762000	0.682157000	0.047046000
	C	2.861571000	-1.183096000	0.833471000
	H	3.838663000	-1.604831000	1.068055000
	H	2.462817000	-0.752776000	1.752403000
	H	2.204369000	-2.003491000	0.539430000
**Heptanal-1,4-rad**	C	-1.545960000	0.182322000	0.184131000
	O	-1.348860000	-0.579183000	1.112099000
	C	-0.789758000	1.351831000	-0.162022000
	O	-2.573204000	-0.016510000	-0.677727000
	O	-3.305471000	-1.169026000	-0.330374000
	H	-1.095819000	1.902634000	-1.041640000
	H	-2.814199000	-1.468661000	0.463073000
	C	0.405335000	1.767306000	0.602301000
	H	0.282731000	2.811658000	0.905110000
	H	0.484206000	1.171060000	1.509820000
	C	1.699447000	1.661054000	-0.228873000
	H	1.623928000	2.333703000	-1.086307000
	H	2.523900000	2.029476000	0.385774000
	C	2.015423000	0.250171000	-0.718510000
	H	1.203419000	-0.104596000	-1.361945000
	H	2.903965000	0.296052000	-1.353642000
	C	2.251710000	-0.766200000	0.393930000
	H	3.036497000	-0.392365000	1.058167000
	H	1.350628000	-0.867723000	1.001423000
	C	2.645051000	-2.134862000	-0.145700000
	H	1.862156000	-2.537274000	-0.790854000
	H	3.562640000	-2.077801000	-0.734204000
	H	2.808734000	-2.848146000	0.661707000
**Heptanal-1,5-TS**	C	2.097102000	-0.103401000	-0.269627000
	O	2.917083000	-0.133561000	-1.131743000
	C	1.609131000	1.126078000	0.470941000
	H	2.133213000	1.165617000	1.426575000

	H	1.918989000	1.982907000	-0.126811000
	C	0.117428000	1.080569000	0.693222000
	C	-0.760005000	1.234877000	-0.518285000
	H	-0.569276000	2.228682000	-0.941302000
	H	-0.448441000	0.525625000	-1.290088000
	C	-2.253203000	1.091097000	-0.233700000
	H	-2.799831000	1.300471000	-1.156190000
	H	-2.551938000	1.861192000	0.482681000
	O	1.455528000	-1.272291000	0.053624000
	O	0.700972000	-1.211079000	1.221847000
	H	-0.222454000	1.620182000	1.575641000
	H	0.072896000	-0.145531000	1.076872000
	C	-2.676966000	-0.279288000	0.294972000
	H	-2.209040000	-0.464741000	1.265316000
	H	-3.752074000	-0.252528000	0.482382000
	C	-2.355694000	-1.431521000	-0.649222000
	H	-2.767247000	-2.367234000	-0.272780000
	H	-2.779018000	-1.253680000	-1.639870000
	H	-1.281207000	-1.575983000	-0.762059000
**Heptanal-1,5-rad**	C	1.588095000	-0.316037000	0.082686000
	O	2.103867000	-0.071660000	1.143228000
	C	1.021068000	-1.643842000	-0.360744000
	H	0.834896000	-1.617487000	-1.433902000
	H	1.794355000	-2.386888000	-0.154928000
	C	-0.228449000	-1.951661000	0.399878000
	C	-1.572468000	-1.743859000	-0.201633000
	H	-1.662540000	-2.350527000	-1.110782000
	H	-2.337374000	-2.099961000	0.490401000
	C	-1.871687000	-0.280953000	-0.578749000
	H	-2.850524000	-0.240189000	-1.063133000
	H	-1.147296000	0.057860000	-1.323743000
	O	1.419267000	0.628468000	-0.856285000
	H	-0.124508000	-2.163775000	1.454595000
	C	-1.851428000	0.672587000	0.609593000
	H	-2.609337000	0.360287000	1.333596000
	H	-0.891084000	0.594445000	1.126464000
	C	-2.086204000	2.119993000	0.200852000
	H	-2.085769000	2.782400000	1.066276000
	H	-1.304159000	2.459716000	-0.479672000
	H	-3.045694000	2.233816000	-0.307098000
	O	1.879602000	1.889765000	-0.410688000
	H	2.213572000	1.660351000	0.480583000
**Heptanal-1,6-TS**	C	1.967194000	-0.567126000	-0.311747000
	O	2.459941000	-1.118706000	-1.247724000
	C	2.103559000	0.892298000	0.029879000
	H	2.336496000	1.005691000	1.086455000

	H	2.932027000	1.269857000	-0.562937000
	C	0.819486000	1.679725000	-0.315253000
	H	0.668079000	1.623108000	-1.395332000
	H	1.005652000	2.725515000	-0.072293000
	C	-0.436421000	1.192664000	0.389703000
	C	-1.479547000	0.520503000	-0.461517000
	H	-1.006672000	-0.267379000	-1.056652000
	H	-1.853003000	1.256210000	-1.186393000
	C	-2.645743000	-0.058866000	0.328935000
	H	-2.259797000	-0.791473000	1.041024000
	H	-3.109325000	0.735361000	0.920234000
	O	1.125796000	-1.316950000	0.477116000
	O	0.688051000	-0.658531000	1.617368000
	H	-0.844636000	1.892063000	1.119067000
	H	-0.014302000	0.257461000	1.162901000
	C	-3.690306000	-0.711396000	-0.565884000
	H	-4.108506000	0.008388000	-1.271755000
	H	-3.253181000	-1.527379000	-1.143212000
	H	-4.512466000	-1.120745000	0.020114000
**Heptanal-1,6-rad**	C	1.400142000	-0.426462000	0.283287000
	O	1.279884000	0.110404000	1.356053000
	C	0.915597000	-1.795318000	-0.091255000
	H	1.163079000	-1.998385000	-1.131895000
	H	1.456624000	-2.507146000	0.533891000
	C	-0.596221000	-1.920481000	0.147158000
	H	-0.808530000	-1.709580000	1.197049000
	H	-0.864343000	-2.971785000	-0.016724000
	C	-1.401680000	-1.029273000	-0.731449000
	C	-2.528407000	-0.217384000	-0.199984000
	H	-3.144695000	-0.836526000	0.461840000
	H	-3.172798000	0.108224000	-1.019335000
	C	-2.071698000	1.021632000	0.595456000
	H	-2.949627000	1.480624000	1.055026000
	H	-1.421096000	0.702866000	1.412716000
	O	2.008025000	0.183827000	-0.745735000
	H	-1.133444000	-0.966401000	-1.778900000
	C	-1.345133000	2.046728000	-0.264078000
	H	-1.033531000	2.906793000	0.328206000
	H	-0.452449000	1.620938000	-0.724266000
	H	-1.988416000	2.408902000	-1.068492000
	O	2.457310000	1.475477000	-0.388373000
	H	2.151873000	1.527151000	0.540517000
**Heptanal-1,7-TS**	C	-0.886238000	1.684260000	-0.541200000
	H	-0.670244000	1.348605000	-1.556919000
	H	-1.430574000	2.622786000	-0.645933000
	C	0.408478000	1.987624000	0.215396000

	H	0.159085000	2.412069000	1.190441000
	C	1.362578000	0.835266000	0.417379000
	H	2.180502000	1.082279000	1.096080000
	C	-1.838183000	0.677361000	0.134307000
	H	-1.857965000	0.833943000	1.210061000
	H	-2.839398000	0.825908000	-0.263862000
	C	-1.514374000	-0.757302000	-0.198155000
	O	-1.935581000	-1.345718000	-1.148369000
	C	1.815716000	0.048938000	-0.784173000
	H	0.956133000	-0.320159000	-1.347309000
	H	2.336744000	0.740688000	-1.458358000
	O	-0.646297000	-1.454990000	0.606697000
	H	0.941790000	2.775268000	-0.329819000
	O	-0.144486000	-0.728674000	1.673610000
	H	0.704061000	0.001674000	1.146216000
	C	2.727758000	-1.116726000	-0.426042000
	H	3.614336000	-0.771201000	0.107824000
	H	2.205190000	-1.829769000	0.211397000
	H	3.056834000	-1.643573000	-1.320394000
**Heptanal-1,7-rad**	C	-0.505553000	2.092378000	0.404841000
	H	-0.709270000	1.714766000	1.407591000
	H	-0.608479000	3.177257000	0.445941000
	C	-1.531649000	1.527171000	-0.594309000
	H	-1.362962000	1.994132000	-1.566939000
	C	-1.497091000	0.045332000	-0.729459000
	H	-1.109688000	-0.398526000	-1.637729000
	C	0.937919000	1.755676000	0.032101000
	H	1.142298000	1.992437000	-1.011539000
	H	1.625636000	2.339470000	0.646921000
	C	1.275658000	0.317208000	0.295051000
	O	1.104461000	-0.285763000	1.326171000
	C	-2.025745000	-0.844751000	0.336181000
	H	-1.629077000	-0.531509000	1.309071000
	H	-3.114496000	-0.712048000	0.415591000
	O	1.870781000	-0.255578000	-0.763819000
	H	-2.525101000	1.847286000	-0.258549000
	O	2.216179000	-1.599406000	-0.493969000
	C	-1.703617000	-2.316270000	0.109461000
	H	-2.106763000	-2.937642000	0.908381000
	H	-2.123435000	-2.665972000	-0.834789000
	H	-0.624956000	-2.471093000	0.071451000
	H	1.915390000	-1.683038000	0.434331000
**Heptanal-1,8-TS**	C	1.398882000	-0.872465000	-0.243383000
	O	1.899178000	-1.189442000	-1.280546000
	C	1.825737000	0.307921000	0.589062000
	H	1.482556000	0.214879000	1.614719000

	H	2.914162000	0.267870000	0.576109000
	C	1.386371000	1.652786000	-0.010339000
	H	1.640697000	1.665593000	-1.072432000
	H	1.993965000	2.425666000	0.462133000
	C	-0.084901000	2.023588000	0.176126000
	C	-1.090974000	1.161392000	-0.607865000
	H	-0.577991000	0.554200000	-1.359062000
	H	-1.760993000	1.811027000	-1.182454000
	C	-1.962848000	0.272745000	0.240952000
	H	-1.148290000	-0.473134000	0.896984000
	H	-2.452092000	0.804435000	1.058771000
	O	0.363873000	-1.669538000	0.191797000
	O	-0.265281000	-1.221908000	1.340662000
	H	-0.197255000	3.066185000	-0.122769000
	H	-0.328387000	1.988286000	1.241342000
	C	-2.854138000	-0.691714000	-0.489450000
	H	-2.270132000	-1.334607000	-1.149083000
	H	-3.414030000	-1.321706000	0.199281000
	H	-3.573150000	-0.147108000	-1.109462000
**Heptanal-1,8-rad**	C	1.627806000	-0.112568000	-0.347978000
	O	1.333762000	0.952418000	-0.831211000
	C	0.938678000	-1.427084000	-0.575230000
	H	1.300567000	-1.798707000	-1.536434000
	H	1.259161000	-2.135742000	0.187935000
	C	-0.579025000	-1.272984000	-0.602054000
	H	-1.017990000	-2.221160000	-0.918225000
	H	-0.847703000	-0.533545000	-1.357843000
	C	-1.159471000	-0.864602000	0.746503000
	C	-2.673099000	-0.619203000	0.690250000
	H	-3.012476000	-0.406088000	1.714182000
	H	-3.178321000	-1.539605000	0.389556000
	C	-3.071615000	0.498634000	-0.210617000
	H	-3.880334000	0.351398000	-0.912052000
	O	2.675041000	-0.243642000	0.480432000
	H	-0.663937000	0.044349000	1.098282000
	H	-0.946381000	-1.638648000	1.487823000
	C	-2.417436000	1.828938000	-0.115381000
	H	-2.940797000	2.579400000	-0.705084000
	H	-2.377567000	2.181201000	0.922305000
	H	-1.377479000	1.803029000	-0.464801000
	O	3.351511000	0.984661000	0.660094000
	H	2.805727000	1.579836000	0.106868000
**Heptanal-1,9-TS**	C	0.817524000	-1.240710000	0.221320000
	O	0.656480000	-1.786720000	1.266642000
	C	2.004163000	-0.404602000	-0.192202000
	H	2.640715000	-0.325233000	0.687642000

	H	2.555003000	-0.968717000	-0.947663000
	C	1.659426000	0.980153000	-0.755148000
	H	1.049317000	0.859247000	-1.650076000
	H	2.593664000	1.434944000	-1.087992000
	C	0.987265000	1.945182000	0.227001000
	C	-0.375069000	1.516966000	0.778036000
	H	-0.694286000	2.253411000	1.517973000
	H	-0.270024000	0.586289000	1.336009000
	C	-1.477991000	1.360168000	-0.276042000
	H	-1.972655000	2.324345000	-0.440546000
	H	-1.052678000	1.089029000	-1.244604000
	O	-0.086357000	-1.271957000	-0.813109000
	O	-1.305829000	-1.812996000	-0.449534000
	H	0.882373000	2.912093000	-0.272411000
	H	1.661610000	2.110466000	1.071168000
	C	-2.515503000	0.337809000	0.089331000
	H	-1.945986000	-0.831396000	-0.123053000
	H	-3.374471000	0.276257000	-0.573859000
	H	-2.785818000	0.282842000	1.141643000
**Heptanal-1,9-rad**	C	1.721753000	-0.200771000	-0.044727000
	O	2.366226000	-0.174395000	0.973173000
	C	1.089480000	-1.402927000	-0.679303000
	H	0.676354000	-1.127035000	-1.647217000
	H	1.883643000	-2.130818000	-0.844914000
	C	0.017264000	-2.003379000	0.239851000
	H	0.504183000	-2.367162000	1.145058000
	H	-0.406549000	-2.872987000	-0.266457000
	C	-1.094896000	-1.029643000	0.620398000
	C	-1.916521000	-0.514139000	-0.556497000
	H	-2.370859000	-1.360245000	-1.078869000
	H	-1.267208000	-0.011828000	-1.278145000
	C	-3.005922000	0.469004000	-0.128840000
	H	-3.646404000	0.681229000	-0.997278000
	H	-3.667857000	-0.006564000	0.602802000
	O	1.462917000	0.917844000	-0.743160000
	O	2.025620000	2.056894000	-0.123666000
	H	-0.669300000	-0.180646000	1.163544000
	H	-1.763212000	-1.527717000	1.327054000
	C	-2.473062000	1.741507000	0.419369000
	H	2.451077000	1.650651000	0.659144000
	H	-1.499938000	2.105395000	0.116418000
	H	-3.090608000	2.387079000	1.026575000
**Octanal-AcOO**	C	-2.475650000	5.169719000	0.000000000
	H	-1.933521000	5.518670000	0.880699000

	H	-3.455004000	5.648035000	0.000000000
	H	-1.933521000	5.518670000	-0.880699000
	C	-2.595983000	3.651623000	0.000000000
	H	-3.167903000	3.328950000	0.874281000
	H	-3.167903000	3.328950000	-0.874281000
	C	-1.243848000	2.949091000	0.000000000
	H	-0.670048000	3.271198000	-0.874955000
	H	-0.670048000	3.271198000	0.874955000
	C	-1.352856000	1.429942000	0.000000000
	H	-1.926035000	1.107756000	0.874813000
	H	-1.926035000	1.107756000	-0.874813000
	C	0.000000000	0.730191000	0.000000000
	H	0.573516000	1.051618000	-0.875105000
	H	0.573516000	1.051618000	0.875105000
	C	-0.118274000	-0.788453000	0.000000000
	H	-0.686379000	-1.116430000	0.872499000
	H	-0.686379000	-1.116430000	-0.872499000
	C	1.245205000	-1.463672000	0.000000000
	H	1.839990000	-1.167216000	-0.866327000
	H	1.839990000	-1.167216000	0.866327000
	C	1.135961000	-2.954294000	0.000000000
	O	0.165391000	-3.629849000	0.000000000
	O	2.371549000	-3.682639000	0.000000000
	O	3.441614000	-2.935265000	0.000000000
**Octanal-1,4-TS**	C	1.992776000	0.933592000	0.152956000
	O	1.453943000	1.992868000	0.189768000
	C	1.735979000	-0.257976000	1.040675000
	O	2.956494000	0.645556000	-0.756197000
	O	3.369520000	-0.687238000	-0.578638000
	H	2.167999000	-0.126887000	2.030737000
	H	2.575650000	-0.954198000	0.384086000
	C	0.341615000	-0.817833000	1.020066000
	H	0.294041000	-1.685282000	1.681335000
	H	-0.311122000	-0.058142000	1.464338000
	C	-0.153691000	-1.181064000	-0.376784000
	H	-0.087306000	-0.302296000	-1.022734000
	H	0.517204000	-1.926379000	-0.808936000
	C	-1.581892000	-1.722209000	-0.393172000
	H	-1.841515000	-1.965610000	-1.426223000
	H	-1.613457000	-2.664733000	0.160284000
	C	-2.641165000	-0.776698000	0.173667000
	H	-3.619032000	-1.253765000	0.068407000
	H	-2.492659000	-0.646744000	1.250073000
	C	-2.683320000	0.594267000	-0.493343000
	H	-1.740990000	1.121244000	-0.324848000
	H	-2.770418000	0.464112000	-1.575873000

	C	-3.832133000	1.455125000	0.014717000
	H	-4.794776000	0.978417000	-0.178401000
	H	-3.752686000	1.614572000	1.091653000
	H	-3.839603000	2.432970000	-0.466049000
**Octanal-1,4-rad**	C	1.850074000	-0.116100000	0.214268000
	O	1.349135000	-0.879862000	1.017856000
	C	1.526817000	1.266073000	0.003102000
	O	2.832891000	-0.528415000	-0.623344000
	O	3.138749000	-1.887103000	-0.409883000
	H	2.061672000	1.790213000	-0.777907000
	H	2.514259000	-2.106447000	0.313000000
	C	0.458558000	1.947118000	0.764771000
	H	0.128738000	1.308713000	1.582665000
	H	0.868866000	2.860752000	1.205260000
	C	-0.735850000	2.342585000	-0.126759000
	H	-1.456433000	2.877141000	0.496302000
	H	-0.391164000	3.050423000	-0.884046000
	C	-1.423255000	1.163640000	-0.810816000
	H	-2.202913000	1.553890000	-1.470047000
	H	-0.707225000	0.651009000	-1.461574000
	C	-2.040659000	0.152053000	0.147943000
	H	-1.264198000	-0.286065000	0.779568000
	H	-2.734628000	0.668585000	0.819724000
	C	-2.776700000	-0.971961000	-0.571018000
	H	-3.563198000	-0.546221000	-1.200492000
	H	-2.081967000	-1.476170000	-1.248301000
	C	-3.380969000	-1.989115000	0.387598000
	H	-4.099162000	-1.514796000	1.058946000
	H	-3.899875000	-2.783888000	-0.148258000
	H	-2.607081000	-2.449848000	1.003597000
**Octanal-1,5-TS**	C	-2.884537000	-0.424980000	-0.387313000
	O	-3.465563000	-1.279136000	-0.978906000
	C	-2.393678000	-0.496820000	1.045729000
	H	-3.129530000	0.008586000	1.672331000
	H	-2.387640000	-1.554159000	1.309815000
	C	-1.040297000	0.149606000	1.201507000
	C	0.135395000	-0.537332000	0.565487000
	H	0.312017000	-1.479269000	1.100900000
	H	-0.113965000	-0.821005000	-0.461390000
	C	1.407308000	0.301857000	0.573307000
	H	1.649398000	0.583799000	1.602570000
	H	1.221875000	1.234324000	0.033009000
	O	-2.543244000	0.714806000	-1.068724000
	O	-2.098715000	1.761652000	-0.265501000
	H	-0.848644000	0.562108000	2.190231000
	H	-1.300677000	1.195638000	0.500475000

	C	2.596958000	-0.416690000	-0.048989000
	H	2.781600000	-1.350228000	0.492186000
	H	2.348972000	-0.703362000	-1.075597000
	C	3.869087000	0.421905000	-0.054714000
	H	3.681687000	1.352967000	-0.596119000
	H	4.114689000	0.709241000	0.971328000
	C	5.053067000	-0.303546000	-0.679800000
	H	4.842325000	-0.574470000	-1.715709000
	H	5.949253000	0.316499000	-0.672936000
	H	5.278989000	-1.223252000	-0.137324000
**Octanal-1,5-rad**	C	1.606257000	-0.393545000	0.337872000
	O	1.534757000	0.272705000	1.338519000
	C	1.309749000	-1.869429000	0.202437000
	H	1.680788000	-2.209500000	-0.766707000
	H	1.867369000	-2.375448000	0.988991000
	C	-0.156269000	-2.115503000	0.338751000
	C	-1.124807000	-1.752926000	-0.729183000
	H	-0.625375000	-1.164557000	-1.502926000
	H	-1.475014000	-2.663847000	-1.232528000
	C	-2.353299000	-1.000884000	-0.197305000
	H	-2.877478000	-1.651845000	0.507091000
	H	-3.043254000	-0.822684000	-1.026049000
	O	1.938033000	0.143119000	-0.848062000
	H	-0.531397000	-2.460315000	1.293110000
	C	-2.025993000	0.321171000	0.493105000
	H	-2.934454000	0.708300000	0.962042000
	H	-1.316870000	0.140130000	1.306828000
	C	-1.457083000	1.392035000	-0.431566000
	H	-0.558478000	1.024344000	-0.930502000
	H	-2.178832000	1.601551000	-1.226252000
	C	-1.116543000	2.677752000	0.309585000
	H	-0.693414000	3.424120000	-0.362419000
	H	-2.003464000	3.108967000	0.777496000
	H	-0.389427000	2.483191000	1.100363000
	O	2.153976000	1.536962000	-0.745043000
	H	1.973098000	1.683280000	0.205977000
**Octanal-1,6-TS**	C	2.385301000	-0.476895000	-0.140175000
	O	3.254682000	-0.819295000	-0.881466000
	C	2.253928000	0.884965000	0.491375000
	H	2.121179000	0.796788000	1.568512000
	H	3.189934000	1.398110000	0.290065000
	C	1.076644000	1.661135000	-0.133008000
	H	1.136817000	1.578057000	-1.219952000
	H	1.205239000	2.719537000	0.105552000
	C	-0.289253000	1.211303000	0.340565000
	C	-1.402877000	1.236853000	-0.670364000

	H	-1.137895000	0.587884000	-1.509537000
	H	-1.446387000	2.250146000	-1.089564000
	C	-2.778078000	0.869807000	-0.118933000
	H	-3.053582000	1.594487000	0.652115000
	H	-3.510400000	0.982627000	-0.922241000
	O	1.354513000	-1.364441000	0.058394000
	O	0.507055000	-1.017355000	1.101080000
	H	-0.563008000	1.621075000	1.315021000
	H	-0.084746000	-0.008250000	0.698849000
	C	-2.887884000	-0.541720000	0.458052000
	H	-3.905974000	-0.677901000	0.828007000
	H	-2.236182000	-0.640051000	1.329409000
	C	-2.560215000	-1.644500000	-0.540953000
	H	-1.511302000	-1.623104000	-0.836953000
	H	-2.750059000	-2.626797000	-0.110129000
	H	-3.169021000	-1.549566000	-1.442567000
**Octanal-1,6-rad**	C	-1.595243000	0.027557000	0.307284000
	O	-1.181592000	0.549499000	1.312373000
	C	-1.805395000	-1.440191000	0.082788000
	H	-2.560048000	-1.767858000	0.799190000
	H	-2.193364000	-1.603858000	-0.921223000
	C	-0.497447000	-2.217989000	0.297480000
	H	-0.745702000	-3.285362000	0.246895000
	H	-0.137884000	-2.033442000	1.311413000
	C	0.556997000	-1.876745000	-0.696240000
	C	1.956587000	-1.583582000	-0.285284000
	H	2.612862000	-1.604829000	-1.157458000
	H	2.310182000	-2.367679000	0.394771000
	C	2.114155000	-0.228603000	0.429233000
	H	1.447530000	-0.197923000	1.295210000
	H	3.132669000	-0.150702000	0.818803000
	O	-1.918183000	0.746546000	-0.778655000
	H	0.277491000	-1.835231000	-1.741704000
	C	1.819920000	0.966464000	-0.468739000
	H	0.832081000	0.849731000	-0.920642000
	H	2.531129000	0.977216000	-1.299641000
	C	1.879002000	2.290230000	0.280025000
	H	2.870156000	2.458791000	0.705315000
	H	1.647582000	3.129381000	-0.375811000
	H	1.162909000	2.296472000	1.104109000
	O	-1.702428000	2.127575000	-0.568710000
	H	-1.341428000	2.123484000	0.341427000
**Octanal-1,7-TS**	C	0.969050000	1.683483000	0.366542000
	H	0.236368000	2.209758000	0.979383000
	H	1.804369000	2.368225000	0.219901000
	C	0.375754000	1.360940000	-1.007678000

	H	1.168282000	1.015949000	-1.676063000
	C	-0.748635000	0.355304000	-1.025251000
	H	-1.049923000	0.080108000	-2.036580000
	C	1.469859000	0.466410000	1.168579000
	H	2.078845000	0.811607000	2.000443000
	H	0.637898000	-0.112716000	1.557420000
	C	2.359722000	-0.396826000	0.316148000
	O	3.531057000	-0.236802000	0.154224000
	C	-1.914513000	0.560277000	-0.095056000
	H	-1.564504000	0.636470000	0.938056000
	H	-2.372452000	1.531334000	-0.326642000
	O	1.774977000	-1.402602000	-0.422439000
	H	0.006624000	2.294011000	-1.448784000
	O	0.449299000	-1.662423000	-0.110901000
	H	-0.193581000	-0.749255000	-0.632784000
	C	-2.970446000	-0.535340000	-0.192384000
	H	-2.505786000	-1.499022000	0.028242000
	H	-3.329172000	-0.595707000	-1.222953000
	C	-4.144289000	-0.302989000	0.748394000
	H	-4.640368000	0.644452000	0.530470000
	H	-4.885851000	-1.096125000	0.659530000
	H	-3.812426000	-0.272025000	1.787527000
**Octanal-1,7-rad**	C	-1.385975000	-1.928389000	0.398237000
	H	-0.937943000	-1.829386000	1.387798000
	H	-2.077343000	-2.770663000	0.443932000
	C	-0.298202000	-2.229470000	-0.648699000
	H	-0.776735000	-2.344494000	-1.623724000
	C	0.783575000	-1.210242000	-0.725812000
	H	0.832725000	-0.548773000	-1.581014000
	C	-2.189655000	-0.667756000	0.083647000
	H	-2.558137000	-0.682704000	-0.941559000
	H	-3.055172000	-0.605647000	0.746198000
	C	-1.410741000	0.593824000	0.315540000
	O	-0.815178000	0.901364000	1.318054000
	C	1.804585000	-1.098529000	0.348176000
	H	1.320942000	-0.816787000	1.294705000
	H	2.233972000	-2.092489000	0.533115000
	O	-1.489808000	1.419861000	-0.740741000
	H	0.136023000	-3.203533000	-0.393615000
	O	-0.807544000	2.634292000	-0.501319000
	C	2.926669000	-0.104036000	0.056065000
	H	3.391305000	-0.359661000	-0.899733000
	H	3.702137000	-0.216722000	0.816116000
	C	2.457126000	1.345146000	0.029567000
	H	1.706318000	1.511725000	-0.743690000
	H	2.010240000	1.619789000	0.986825000

	H	3.287134000	2.024593000	-0.164056000
	H	-0.439507000	2.467327000	0.390417000
**Octanal-1,8-TS**	C	-1.501773000	-1.144074000	0.238543000
	O	-1.951481000	-1.556080000	1.265445000
	C	-2.183913000	-0.142472000	-0.656029000
	H	-1.776592000	-0.167731000	-1.661931000
	H	-3.221761000	-0.471745000	-0.687267000
	C	-2.149454000	1.286590000	-0.092802000
	H	-2.456037000	1.260333000	0.955165000
	H	-2.913105000	1.856277000	-0.623897000
	C	-0.820687000	2.030627000	-0.226331000
	C	0.330405000	1.490501000	0.642184000
	H	-0.048319000	0.792716000	1.394152000
	H	0.770953000	2.312402000	1.217544000
	C	1.452196000	0.838740000	-0.123200000
	H	0.901581000	-0.105723000	-0.798687000
	H	1.844180000	1.460617000	-0.931703000
	O	-0.273266000	-1.649075000	-0.124155000
	O	0.273958000	-1.073644000	-1.258532000
	H	-1.005523000	3.073716000	0.032074000
	H	-0.515963000	2.030125000	-1.276195000
	C	2.516477000	0.145682000	0.683921000
	H	2.041822000	-0.595576000	1.331729000
	H	2.971385000	0.885327000	1.354145000
	C	3.592448000	-0.514091000	-0.167699000
	H	4.346491000	-0.992152000	0.456099000
	H	3.157021000	-1.275856000	-0.814423000
	H	4.095979000	0.218643000	-0.800392000
**Octanal-1,8-rad**	C	-1.934413000	-0.102501000	0.368613000
	O	-1.385639000	-1.142518000	0.635931000
	C	-1.526919000	1.272490000	0.813260000
	H	-2.048414000	2.013483000	0.208026000
	H	-1.885751000	1.379773000	1.839277000
	C	-0.013851000	1.460379000	0.756948000
	H	0.463942000	0.680830000	1.352022000
	H	0.234628000	2.413249000	1.228089000
	C	0.536719000	1.433579000	-0.663837000
	C	2.066350000	1.531455000	-0.707627000
	H	2.380928000	2.479229000	-0.264764000
	H	2.367018000	1.578248000	-1.764710000
	C	2.769793000	0.402061000	-0.037325000
	H	3.646013000	0.609580000	0.563747000
	O	-3.042847000	-0.066249000	-0.386500000
	H	0.106294000	2.256728000	-1.239341000
	H	0.222408000	0.512488000	-1.161898000
	C	2.406822000	-1.014547000	-0.309133000

	H	2.400850000	-1.189075000	-1.394889000
	H	1.373736000	-1.208287000	0.007356000
	C	3.333550000	-2.018528000	0.365093000
	H	4.362788000	-1.888242000	0.027806000
	H	3.318642000	-1.890157000	1.448251000
	H	3.033779000	-3.042725000	0.145545000
	O	-3.452926000	-1.364005000	-0.768984000
	H	-2.753868000	-1.909276000	-0.354053000
**Octanal-1,9-TS**	C	-1.353688000	-1.166166000	0.302773000
	O	-1.661325000	-1.559966000	1.387722000
	C	-2.089789000	-0.099842000	-0.462901000
	H	-1.912878000	-0.192837000	-1.530460000
	H	-3.138783000	-0.313701000	-0.261350000
	C	-1.791219000	1.334391000	0.009071000
	H	-1.760106000	1.347972000	1.101039000
	H	-2.659353000	1.930958000	-0.269477000
	C	-0.532138000	2.003389000	-0.574019000
	C	0.635049000	2.166293000	0.400108000
	H	0.277362000	2.694353000	1.287404000
	H	1.382784000	2.818066000	-0.060039000
	C	1.327524000	0.881350000	0.842563000
	H	2.075211000	1.132723000	1.604505000
	H	0.628714000	0.210589000	1.352068000
	O	-0.268424000	-1.785109000	-0.276000000
	O	0.207468000	-1.150702000	-1.410935000
	H	-0.804588000	3.002109000	-0.917821000
	H	-0.204775000	1.463371000	-1.464088000
	C	2.038751000	0.134115000	-0.256428000
	H	1.122692000	-0.441209000	-0.949171000
	H	2.496206000	0.784583000	-1.004313000
	C	2.909542000	-1.013340000	0.175404000
	H	3.311476000	-1.556037000	-0.678381000
	H	3.751544000	-0.643238000	0.768273000
	H	2.348557000	-1.712624000	0.796088000
**Octanal-1,9-rad**	C	-1.931078000	-0.066476000	-0.017323000
	O	-2.486443000	0.057568000	1.044868000
	C	-1.534764000	-1.349941000	-0.682946000
	H	-2.452999000	-1.911413000	-0.858219000
	H	-1.085080000	-1.132663000	-1.649543000
	C	-0.590212000	-2.164542000	0.208652000
	H	-0.351637000	-3.090181000	-0.318951000
	H	-1.126444000	-2.443426000	1.116101000
	C	0.697592000	-1.436203000	0.584458000
	C	1.572431000	-1.044182000	-0.600444000
	H	1.037399000	-0.345231000	-1.247881000
	H	1.788263000	-1.928994000	-1.206050000

	C	2.898526000	-0.393531000	-0.168878000
	H	3.468362000	-1.107820000	0.428927000
	H	3.485651000	-0.204778000	-1.076735000
	O	-1.571167000	1.000662000	-0.751202000
	O	-1.924493000	2.210837000	-0.111500000
	H	1.273547000	-2.079783000	1.254036000
	H	0.456888000	-0.542158000	1.167583000
	C	2.725183000	0.880634000	0.582090000
	H	-2.342305000	1.871721000	0.706846000
	H	2.991174000	0.922919000	1.629270000
	C	2.030481000	2.038918000	-0.035188000
	H	0.938240000	1.924920000	-0.023119000
	H	2.255400000	2.969940000	0.484066000
	H	2.311413000	2.156350000	-1.086197000
**Nonanal-AcOO**	C	-6.404728000	0.095781000	0.000000000
	H	-7.246056000	0.788757000	0.000000000
	H	-6.497563000	-0.542334000	0.880620000
	H	-6.497563000	-0.542334000	-0.880620000
	C	-5.072735000	0.834019000	0.000000000
	H	-5.016105000	1.488309000	0.874322000
	H	-5.016105000	1.488309000	-0.874322000
	C	-3.873062000	-0.105454000	0.000000000
	H	-3.928455000	-0.761279000	0.874811000
	H	-3.928455000	-0.761279000	-0.874811000
	C	-2.534971000	0.622086000	0.000000000
	H	-2.479969000	1.277567000	0.874979000
	H	-2.479969000	1.277567000	-0.874979000
	C	-1.337288000	-0.318944000	0.000000000
	H	-1.391246000	-0.974259000	0.874771000
	H	-1.391246000	-0.974259000	-0.874771000
	C	0.000000000	0.410091000	0.000000000
	H	0.054720000	1.065260000	-0.875105000
	H	0.054720000	1.065260000	0.875105000
	C	1.191981000	-0.538255000	0.000000000
	H	1.145738000	-1.192613000	0.872486000
	H	1.145738000	-1.192613000	-0.872486000
	C	2.514951000	0.213277000	0.000000000
	H	2.602225000	0.872065000	0.866347000
	H	2.602225000	0.872065000	-0.866347000
	C	3.688737000	-0.712012000	0.000000000
	O	3.704783000	-1.894432000	0.000000000
	O	4.984258000	-0.096425000	0.000000000
	O	4.964716000	1.208642000	0.000000000
**Nonanal-1,4-TS**	C	2.142869000	1.092467000	0.221033000

O	1.410308000	2.019079000	0.357640000
C	2.151458000	-0.194025000	1.007530000
O	3.113500000	1.073355000	-0.725351000
O	3.782422000	-0.162944000	-0.669778000
H	2.583545000	-0.061740000	1.997396000
H	3.087475000	-0.656826000	0.278679000
C	0.892279000	-1.012763000	0.962848000
H	1.036847000	-1.924626000	1.545639000
H	0.121000000	-0.435359000	1.484630000
C	0.427710000	-1.348049000	-0.451399000
H	0.302196000	-0.422372000	-1.018183000
H	1.213389000	-1.910788000	-0.959693000
C	-0.870700000	-2.151498000	-0.489951000
H	-1.117775000	-2.348262000	-1.535909000
H	-0.701717000	-3.127418000	-0.026628000
C	-2.067838000	-1.480793000	0.184395000
H	-2.941847000	-2.123394000	0.051037000
H	-1.906801000	-1.424808000	1.265251000
C	-2.387821000	-0.087899000	-0.345927000
H	-1.553046000	0.591243000	-0.148685000
H	-2.493362000	-0.130183000	-1.435110000
C	-3.653139000	0.505197000	0.261689000
H	-4.497985000	-0.156758000	0.052356000
H	-3.548602000	0.531296000	1.350067000
C	-3.957344000	1.905004000	-0.254912000
H	-4.864614000	2.308279000	0.194856000
H	-3.138330000	2.590040000	-0.030024000
H	-4.095064000	1.899898000	-1.337598000

Nonanal-1,4-rad

C	2.149851000	-0.508511000	0.203786000
O	1.466395000	-1.058323000	1.046817000
C	2.256700000	0.899428000	-0.052566000
O	2.932221000	-1.226374000	-0.638866000
O	2.815784000	-2.606479000	-0.380312000
H	2.900179000	1.211701000	-0.864479000
H	2.178171000	-2.602228000	0.364103000
C	1.471963000	1.896374000	0.705979000
H	0.990094000	1.413316000	1.554344000
H	2.155012000	2.653435000	1.102707000
C	0.426513000	2.611398000	-0.173708000
H	-0.079164000	3.356075000	0.445094000
H	0.945876000	3.160432000	-0.962469000
C	-0.605646000	1.678307000	-0.801680000
H	-1.250508000	2.268221000	-1.458029000
H	-0.097914000	0.955366000	-1.448614000
C	-1.470577000	0.927367000	0.204301000
H	-0.843103000	0.295986000	0.837571000

	H	-1.959057000	1.648286000	0.868406000
	C	-2.527240000	0.054866000	-0.460644000
	H	-3.172142000	0.676131000	-1.090791000
	H	-2.035279000	-0.654497000	-1.133903000
	C	-3.385764000	-0.714862000	0.535293000
	H	-2.737675000	-1.334140000	1.160872000
	H	-3.875519000	-0.006783000	1.209597000
	C	-4.435463000	-1.589211000	-0.137511000
	H	-5.113258000	-0.988285000	-0.746622000
	H	-3.967606000	-2.325403000	-0.793550000
	H	-5.035335000	-2.129140000	0.595143000
**Nonanal-1,5-TS**	C	-3.435362000	-0.192371000	-0.543637000
	O	-4.024696000	-0.760036000	-1.408268000
	C	-2.989195000	-0.783240000	0.779758000
	H	-3.727218000	-0.502376000	1.532070000
	H	-3.023384000	-1.865234000	0.653856000
	C	-1.621673000	-0.286520000	1.176138000
	C	-0.449516000	-0.751866000	0.358780000
	H	-0.323002000	-1.829641000	0.524780000
	H	-0.674105000	-0.639312000	-0.706166000
	C	0.849152000	-0.027272000	0.690851000
	H	1.066394000	-0.143099000	1.757081000
	H	0.713766000	1.043941000	0.517815000
	O	-3.039369000	1.100124000	-0.773041000
	O	-2.586784000	1.778728000	0.355238000
	H	-1.446768000	-0.256389000	2.249912000
	H	-1.828367000	0.948718000	0.884690000
	C	2.033812000	-0.526919000	-0.125268000
	H	2.165702000	-1.599975000	0.046114000
	H	1.811205000	-0.413114000	-1.190561000
	C	3.333083000	0.198822000	0.198233000
	H	3.200013000	1.271742000	0.027519000
	H	3.555944000	0.085737000	1.264111000
	C	4.520408000	-0.296347000	-0.618416000
	H	4.296267000	-0.181950000	-1.682396000
	H	4.651348000	-1.368476000	-0.447888000
	C	5.814141000	0.435475000	-0.287262000
	H	6.075362000	0.310789000	0.765119000
	H	6.647082000	0.063948000	-0.883949000
	H	5.717450000	1.505587000	-0.478605000
**Nonanal-1,5-rad**	C	1.119867000	-1.447801000	0.322660000
	O	0.550674000	-1.524231000	1.381118000
	C	2.466129000	-0.815027000	0.054752000
	H	3.161132000	-1.240971000	0.776944000
	H	2.786951000	-1.096897000	-0.950227000
	C	2.380190000	0.667710000	0.200945000

	C	1.716163000	1.528113000	-0.813196000
	H	2.476697000	2.075906000	-1.385327000
	H	1.190465000	0.907884000	-1.543566000
	C	0.755170000	2.552585000	-0.194043000
	H	0.360080000	3.190926000	-0.988369000
	H	1.328095000	3.204235000	0.470629000
	O	0.570439000	-1.913182000	-0.811555000
	H	2.708891000	1.110840000	1.131553000
	C	-0.399796000	1.930893000	0.587889000
	H	0.000457000	1.268779000	1.361787000
	H	-0.935831000	2.724854000	1.114220000
	C	-1.393035000	1.154423000	-0.268857000
	H	-1.821862000	1.824195000	-1.021738000
	H	-0.882829000	0.363812000	-0.825526000
	C	-2.514901000	0.525005000	0.547844000
	H	-3.022984000	1.304058000	1.123125000
	H	-2.075995000	-0.156596000	1.282123000
	C	-3.526311000	-0.221625000	-0.311570000
	H	-4.310570000	-0.673521000	0.296169000
	H	-4.004612000	0.452492000	-1.024584000
	H	-3.039857000	-1.014957000	-0.880977000
	O	-0.712462000	-2.460359000	-0.579317000
	H	-0.808292000	-2.308525000	0.383244000
**Nonanal-1,6-TS**	C	2.474621000	-0.803265000	-0.148198000
	O	3.245669000	-1.320788000	-0.896621000
	C	2.664132000	0.530780000	0.526486000
	H	2.495906000	0.444864000	1.598824000
	H	3.699052000	0.809675000	0.350179000
	C	1.717690000	1.585598000	-0.082253000
	H	1.776787000	1.524898000	-1.170711000
	H	2.090727000	2.574221000	0.195149000
	C	0.275163000	1.459617000	0.360472000
	C	-0.781746000	1.767541000	-0.664605000
	H	-0.650187000	1.102224000	-1.522058000
	H	-0.587879000	2.777370000	-1.048073000
	C	-2.216471000	1.700708000	-0.146045000
	H	-2.347358000	2.462602000	0.627112000
	H	-2.889190000	1.973704000	-0.962706000
	O	1.259838000	-1.425221000	0.018270000
	O	0.506650000	-0.914466000	1.065612000
	H	0.085745000	1.900050000	1.341568000
	H	0.179320000	0.219034000	0.689414000
	C	-2.635985000	0.342772000	0.415287000
	H	-3.668153000	0.419730000	0.766851000
	H	-2.037876000	0.102124000	1.298840000
	C	-2.534952000	-0.809115000	-0.578153000

	H	-3.124706000	-0.569354000	-1.467966000
	H	-1.501514000	-0.924176000	-0.910987000
	C	-3.004235000	-2.129503000	0.016703000
	H	-2.391719000	-2.399092000	0.878225000
	H	-4.042230000	-2.065196000	0.348674000
	H	-2.933281000	-2.939681000	-0.708586000
**Nonanal-1,6-rad**	C	1.163550000	1.328559000	0.299031000
	O	0.585650000	1.220754000	1.351641000
	C	2.499781000	0.745798000	-0.052814000
	H	2.748050000	0.998745000	-1.082258000
	H	3.234096000	1.218826000	0.601097000
	C	2.507405000	-0.776299000	0.154711000
	H	2.250154000	-0.993999000	1.192870000
	H	3.541342000	-1.115126000	0.014591000
	C	1.589252000	-1.497600000	-0.768443000
	C	0.649967000	-2.543097000	-0.281033000
	H	1.189161000	-3.247513000	0.363263000
	H	0.264378000	-3.119692000	-1.124098000
	C	-0.534981000	-1.975175000	0.522318000
	H	-1.096328000	-2.806303000	0.957104000
	H	-0.153100000	-1.385736000	1.359989000
	O	0.637914000	2.017837000	-0.725466000
	H	1.609005000	-1.231182000	-1.817989000
	C	-1.470607000	-1.110783000	-0.312109000
	H	-1.907051000	-1.716606000	-1.113227000
	H	-0.898634000	-0.322029000	-0.810132000
	C	-2.584889000	-0.473112000	0.507255000
	H	-3.149458000	-1.256149000	1.021361000
	H	-2.135682000	0.141830000	1.292405000
	C	-3.529808000	0.372928000	-0.335835000
	H	-4.017125000	-0.233228000	-1.101758000
	H	-4.310242000	0.828533000	0.274058000
	H	-2.986686000	1.172275000	-0.842401000
	O	-0.621975000	2.563002000	-0.389557000
	H	-0.732607000	2.227292000	0.523696000
**Nonanal-1,7-TS**	C	1.592546000	1.614252000	0.513054000
	H	0.897147000	2.167906000	1.144925000
	H	2.503791000	2.209475000	0.454077000
	C	1.025678000	1.475982000	-0.902537000
	H	1.802967000	1.101951000	-1.573140000
	C	-0.199689000	0.607590000	-1.043742000
	H	-0.487367000	0.452728000	-2.084164000
	C	1.921255000	0.286245000	1.222662000
	H	2.527907000	0.490746000	2.101412000
	H	1.014652000	-0.224927000	1.531741000
	C	2.747222000	-0.599064000	0.329453000

	O	3.935009000	-0.559424000	0.222034000
	C	-1.373180000	0.863297000	-0.135889000
	H	-1.057489000	0.816862000	0.909886000
	H	-1.714625000	1.894312000	-0.298589000
	O	2.088514000	-1.468451000	-0.512512000
	H	0.778647000	2.478136000	-1.271536000
	O	0.729925000	-1.601263000	-0.269903000
	H	0.214680000	-0.581077000	-0.731671000
	C	-2.534256000	-0.098460000	-0.358360000
	H	-2.185910000	-1.124093000	-0.207479000
	H	-2.860205000	-0.035982000	-1.400991000
	C	-3.719210000	0.173346000	0.559547000
	H	-4.061940000	1.201073000	0.410460000
	H	-3.388903000	0.109122000	1.599892000
	C	-4.875887000	-0.790130000	0.330883000
	H	-5.243782000	-0.722713000	-0.694323000
	H	-4.564283000	-1.821623000	0.502775000
	H	-5.709746000	-0.577136000	0.999351000
**Nonanal-1,7-rad**	C	2.471112000	-1.185592000	0.346903000
	H	3.464620000	-1.635138000	0.364805000
	H	2.059207000	-1.282286000	1.352241000
	C	1.594725000	-1.949650000	-0.661771000
	H	1.634919000	-3.010750000	-0.387665000
	C	0.175778000	-1.503368000	-0.710150000
	H	-0.182602000	-0.960575000	-1.575222000
	C	2.622147000	0.296194000	0.008066000
	H	3.407334000	0.738960000	0.624310000
	H	2.904270000	0.435061000	-1.035115000
	C	1.384255000	1.092392000	0.300940000
	O	0.758426000	1.101298000	1.331752000
	C	-0.766313000	-1.828133000	0.392518000
	H	-0.730623000	-2.909695000	0.581756000
	H	-0.420345000	-1.364905000	1.327518000
	O	1.054269000	1.884738000	-0.732454000
	H	2.049053000	-1.863610000	-1.651233000
	O	-0.068481000	2.688788000	-0.430916000
	C	-2.209961000	-1.402265000	0.136910000
	H	-2.850094000	-1.829131000	0.913271000
	H	-2.549886000	-1.829746000	-0.811504000
	C	-2.406545000	0.109100000	0.110628000
	H	-1.768948000	0.551818000	-0.657865000
	H	-2.066001000	0.523839000	1.063438000
	C	-3.853297000	0.510853000	-0.140875000
	H	-4.204396000	0.126017000	-1.100094000
	H	-4.511266000	0.114034000	0.634592000
	H	-3.968994000	1.594614000	-0.155377000

	H	-0.291370000	2.365712000	0.466073000
**Nonanal-1,8-TS**	C	1.734188000	-1.285512000	-0.233067000
	O	2.053799000	-1.794640000	-1.265143000
	C	2.603624000	-0.352776000	0.568808000
	H	2.268110000	-0.285570000	1.598977000
	H	3.588393000	-0.817906000	0.548279000
	C	2.719902000	1.046204000	-0.055591000
	H	2.950176000	0.940343000	-1.118005000
	H	3.585425000	1.527439000	0.401666000
	C	1.512164000	1.966018000	0.124246000
	C	0.246613000	1.553784000	-0.649958000
	H	0.480850000	0.783269000	-1.389740000
	H	-0.117180000	2.405062000	-1.236122000
	C	-0.900672000	1.088395000	0.208225000
	H	-0.435365000	0.109619000	0.897609000
	H	-1.158356000	1.789950000	1.005283000
	O	0.476888000	-1.603799000	0.229125000
	O	0.089688000	-0.914421000	1.365772000
	H	1.815393000	2.965069000	-0.190299000
	H	1.278589000	2.043727000	1.189349000
	C	-2.091066000	0.506794000	-0.503371000
	H	-1.757943000	-0.322503000	-1.134648000
	H	-2.488215000	1.266377000	-1.189905000
	C	-3.199637000	0.033075000	0.429129000
	H	-2.791511000	-0.716489000	1.110212000
	H	-3.530517000	0.870629000	1.049200000
	C	-4.387426000	-0.548923000	-0.324501000
	H	-4.083687000	-1.406927000	-0.926127000
	H	-5.166931000	-0.881256000	0.360484000
	H	-4.826362000	0.189700000	-0.997763000
**Nonanal-1,8-rad**	C	2.032562000	-0.219898000	0.322302000
	O	1.397576000	-1.231182000	0.151978000
	C	1.642930000	0.958296000	1.167404000
	H	1.857527000	0.676167000	2.200507000
	H	2.285169000	1.803424000	0.921226000
	C	0.165390000	1.302955000	1.007140000
	H	-0.099219000	2.055550000	1.752275000
	H	-0.431127000	0.417262000	1.229079000
	C	-0.178530000	1.818037000	-0.385392000
	C	-1.680442000	2.060166000	-0.571806000
	H	-1.833619000	2.454625000	-1.587484000
	H	-2.001668000	2.855904000	0.104773000
	C	-2.533350000	0.856405000	-0.357827000
	H	-3.507239000	0.993173000	0.098272000
	O	3.233036000	-0.039756000	-0.248739000
	H	0.169841000	1.104802000	-1.136659000

	H	0.360717000	2.748901000	-0.576858000
	C	-2.244476000	-0.461615000	-0.984613000
	H	-1.165594000	-0.642355000	-1.018813000
	H	-2.571898000	-0.449218000	-2.035678000
	C	-2.913559000	-1.636341000	-0.269897000
	H	-3.992874000	-1.465452000	-0.228689000
	H	-2.766041000	-2.542306000	-0.860747000
	C	-2.369784000	-1.852736000	1.136990000
	H	-1.293103000	-2.030282000	1.109172000
	H	-2.546259000	-0.975625000	1.762414000
	H	-2.842772000	-2.708052000	1.619659000
	O	3.620591000	-1.166395000	-1.009691000
	H	2.844103000	-1.749479000	-0.886632000
**Nonanal-1,9-TS**	C	-1.870594000	-1.053754000	-0.074349000
	O	-2.643232000	-1.383216000	-0.922497000
	C	-2.014454000	0.172835000	0.788056000
	H	-3.002136000	0.114435000	1.244313000
	H	-1.276616000	0.188583000	1.580240000
	C	-1.942732000	1.423204000	-0.102179000
	H	-2.063785000	2.305410000	0.530199000
	H	-2.802858000	1.394610000	-0.770731000
	C	-0.670946000	1.557731000	-0.947837000
	C	0.517715000	2.257514000	-0.287789000
	H	0.198871000	3.255461000	0.023995000
	H	1.283040000	2.416307000	-1.051085000
	C	1.167363000	1.579526000	0.917394000
	H	1.972505000	2.235566000	1.269846000
	H	0.465321000	1.524754000	1.752718000
	O	-0.738828000	-1.830896000	0.040132000
	O	0.049057000	-1.560881000	1.145750000
	H	-0.922548000	2.134580000	-1.839068000
	H	-0.370598000	0.574303000	-1.319812000
	C	1.780261000	0.215059000	0.692971000
	H	0.851920000	-0.677936000	0.814202000
	H	2.362995000	-0.107996000	1.558629000
	C	2.484534000	-0.075692000	-0.606507000
	H	3.277603000	0.672504000	-0.733696000
	H	1.803863000	0.069968000	-1.446910000
	C	3.080561000	-1.476080000	-0.662333000
	H	3.596011000	-1.643658000	-1.607044000
	H	3.798901000	-1.627688000	0.144628000
	H	2.301284000	-2.231317000	-0.562069000
**Nonanal-1,9-rad**	C	-1.875234000	-0.450329000	0.392331000
	O	-1.724722000	0.405595000	1.228403000
	C	-1.373689000	-1.862196000	0.460744000
	H	-1.800000000	-2.301104000	1.363143000

H	-1.737791000	-2.422519000	-0.399365000
C	0.155930000	-1.891028000	0.528551000
H	0.471823000	-2.913149000	0.745535000
H	0.474862000	-1.276521000	1.371316000
C	0.810352000	-1.416192000	-0.764353000
C	2.334425000	-1.369385000	-0.707119000
H	2.716114000	-1.180484000	-1.712674000
H	2.723167000	-2.345777000	-0.403332000
C	2.895513000	-0.285757000	0.236385000
H	2.610559000	-0.513782000	1.266548000
H	3.987328000	-0.350312000	0.198756000
O	-2.524706000	-0.205797000	-0.755979000
O	-2.971445000	1.133778000	-0.823196000
H	0.438860000	-0.419756000	-1.022530000
H	0.499171000	-2.076273000	-1.577911000
C	2.445766000	1.087113000	-0.118389000
H	-2.631363000	1.485697000	0.024929000
H	2.865918000	1.551972000	-1.004328000
C	1.418465000	1.838869000	0.651060000
H	1.906046000	2.521836000	1.362049000
H	0.835157000	1.148645000	1.265548000
C	0.473522000	2.654560000	-0.229760000
H	-0.087072000	2.008650000	-0.907303000
H	-0.243235000	3.208492000	0.376686000
H	1.027135000	3.371839000	-0.837589000

11. MATLAB code for calculating ECKART tunneling coefficient

```
function [G,xa,Va]=eckart(e1,e2,w,mu,T,plotting)

%This program calculates the Eckart correction factor numerically.
%The following input is required:
%e1 is the energy difference between reactants and TS in kcal/mol
%e2 is the energy difference between TS and products in kcal/mol
%w is the numerical value of the imaginary frequency at the TS in cm-1
%mu is the reduced mass of the reaction coordinate (only required for
%plotting, set to 1 if no plotting) in amu
%T is the temperature and can be a vector or a scalar in K
%plotting is set to 1 if plotting of the potential is needed, otherwise it
%is set to 0
%e1 = left barrier = energy difference between transition state and
%product, positive (Sum of electronic and zero-point energies/kcal/mol)
%e2 = right barrier
%w = imaginary frequency of transition state
%mu = reduced mass of transition state
%T = temperature(K)
%plotting (1=on, 0=off)

%Constants
c=299792458 ; %SI
h=2*pi ; %atomic units
k=3.1668152*(10^(-6)); %atomic units

%Converting input to au
v1=e1*(1.5936*(10^(-3)));
v2=e2*(1.5936*(10^(-3)));
wau=w*c*100*(1/(4.134137*(10^16)));
m=mu*1822.888479;

%Calculate force constant, A, B and L
F=-4*(pi^2)*(wau^2)*m;
%F=-4*(pi^2)*(wau^2)*1822.888479;
A=v1-v2;
B=(sqrt(v2)+sqrt(v1))^2;
L=-pi*(A-B)*(B+A)/(sqrt(-2*F*B)*B);

%Defining reaction coordinate
x=-3:0.01:3;

%Removing reduced mass from reaction coordinate in order to get a well
%defined potential
```

```
x(:)=x(:)/sqrt(mu);

%Calculating potential
for i=1:numel(x)
   y(i)=-exp((2*pi*x(i))/L);
   V(i)=((-(y(i)*A)/(1-y(i)))-((y(i)*B)/((1-y(i))^2)));
   xa(i)=0.529177*x(i)*sqrt(mu); %reduced mass re-inserted for plotting
   Va(i)=V(i)*627.51; %potential converted to kcal/mol for plotting
end

%Calculating the correction factors for all T's
VB(1)=V(1); %value of potential at reactants
VB(2)=V(numel(x)); %value of potential at products
Emin=max(VB(:)); %minimum energy at which tunnelling can occur
Gdiff=1;    %initial convergence control set to 1
Gsize=max(V)/50;   %initial integration stepsize
[Gold,Eka,K,GK]=Gcalc(Emin,Gsize,V,A,B,L,h,k,m,T,v1); %calculate G
Gsize=Gsize/10; %reduce integration stepsize
runs=0; %initial number of runs
while Gdiff>=0.001  %convergence criteria
   [Gnew,Eka,K,GK]=Gcalc(Emin,Gsize,V,A,B,L,h,k,m,T,v1); %new G
   display(Gnew); %display new correction factor
   Gsize=Gsize/10; %reduce integration stepsize
   for j=1:numel(T)
      Gdiffcalc(j)=abs(Gnew(j)-Gold(j))/Gold(j); %calculate convergence
   end
   Gdiff=max(Gdiffcalc); %max convergence control value
   display(Gdiff); %display convergence control value
   Gold=Gnew; %replace old correction factor with new
   runs=runs+1;    %a run completed
   display(runs);  %display run number
end
[G,Eka,K,GK]=Gcalc(Emin,Gsize,V,A,B,L,h,k,m,T,v1); %final G


%If plotting needed run plot function
if plotting==1
   plotpotential;
end

%End of main program

function plotpotential
%Plotting function for numparabol program
%Plot potential, transmission and value of integrand in correction factor
%expression
```

```
subplot(2,2,[1 2])
plot(xa,Va) ;
xlabel('Reaction Coordinate in Å*amu^{1/2}'); ylabel('V in kcal/mol');title('Potential');
subplot(2,2,3)
plot(Eka,K);
xlabel('E in kcal/mol'); ylabel('KI');title('Transmission');
subplot(2,2,4)
plot(Eka,GK);
xlabel('E in kcal/mol'); ylabel('GKI');title('Integrand');

%End of plotting program

end
end
function [G,Eka,K,GK]=Gcalc(Emin,Gsize,V,A,B,L,h,k,m,T,v1)
%Sub-function to numparabol
%This program do the actual calculation of the correction factor

%Calculating correction factor
count=1; %index control
for E=Emin:Gsize:(2*max(V)) %interval where transmission may be less than 1
   C=(h^2)/(8*m*(L^2)); %calculation of C factor
   a=0.5*sqrt(E/C);    %calculation of a
   b=0.5*sqrt((E-A)/C); %calculation of b
   d=0.5*sqrt((B-C)/C); %calculation of d
   K(count)=1-((cosh(2*pi*(a-b))+cosh(2*pi*d))/(cosh(2*pi*(a+b))+cosh(2*pi*d))); %calc K
   EK(count)=E;    %save energy for later
   Eka(count)=E*627.51;    %convert energy to kcal/mol for plotting
   count=count+1;  %index control
end
for q=1:numel(T) %temperature XXXperat XXXperations begin
   GK=0; %clean variable
   for j=1:numel(K)
      GK(j)=K(j)*exp(-EK(j)/(k*T(q)));   %calculate integrand
   end
   GI=0; %sum control of integral
   for i=1:(numel(EK)-1) %numerical integration by using the areal of squares
      GI=GI+(0.5*(GK(i)+GK(i+1))*abs(EK(i)-EK(i+1)));
   end
   GI=GI*(exp(v1/(k*T(q)))/(k*T(q))); %multiplying integral with prefactor

   %For energies above the interval where transmission is less than 1, we use
   %analytical integration to obtain the final result
   G(q)=GI+exp(v1/(k*T(q)))*exp(-EK(numel(EK))/(k*T(q)));
end %temperature dependent operations end
```

```
%End of sub-function
End
```

12. MESMER Input files for all the possible H-shifts in pristine acyl peroxy radicals

Ethanal–AcOO ------> Ethanal–AcOOH


```
<?xml version="1.0" encoding="utf-8" ?>
<?xml-stylesheet type='text/xsl' href='../../mesmer2.xsl' media='other'?>
<?xml-stylesheet type='text/xsl' href='../../mesmer1.xsl' media='screen'?>
<me:mesmer xmlns="http://www.xml-cml.org/schema"
xmlns:me="http://www.chem.leeds.ac.uk/mesmer"
xmlns:xsi="http://www.w3.org/2001/XMLSchema-instance">
  <me:title>Project name</me:title>
  <moleculeList convention="">
    <molecule id="N2">
    <atomArray>
      <atom id="a1" elementType="N" />
      <atom id="a2" elementType="N" />
    </atomArray>
    <bondArray>
      <bond atomRefs2="a2 a1" order="3"/>
    </bondArray>
    <propertyList>
      <property dictRef="me:epsilon">
        <scalar>48.0</scalar>
      </property>
      <property dictRef="me:sigma">
        <scalar>3.90</scalar>
      </property>
      <property dictRef="me:MW">
        <scalar units="amu">28.0</scalar>
      </property>
    </propertyList>
  </molecule>
    <molecule id="Ethanal-14-rad" spinMultiplicity="2">
      <atomArray>
        <atom id="a1" elementType="C" x3="-0.473923" y3="0.132072" z3="-0.000057" />
        <atom id="a2" elementType="O" x3="-0.209785" y3="1.320234" z3="-0.000002" />
        <atom id="a3" elementType="C" spinMultiplicity="2" x3="-1.777775" y3="-0.467027"
z3="0.000033" />
        <atom id="a4" elementType="O" x3="0.504537" y3="-0.801697" z3="-0.000047" />
        <atom id="a5" elementType="H" x3="-1.884497" y3="-1.537757" z3="0.000145" />
        <atom id="a6" elementType="H" x3="-2.635330" y3="0.181296" z3="0.000004" />
        <atom id="a7" elementType="O" x3="1.769181" y3="-0.192974" z3="0.000029" />
        <atom id="a8" elementType="H" x3="1.518548" y3="0.761695" z3="0.000158" />
      </atomArray>
```

```
<bondArray>
  <bond atomRefs2="a1 a4" order="1" />
  <bond atomRefs2="a1 a2" order="2" />
  <bond atomRefs2="a1 a3" order="1" />
  <bond atomRefs2="a4 a7" order="1" />
  <bond atomRefs2="a6 a3" order="1" />
  <bond atomRefs2="a7 a8" order="1" />
  <bond atomRefs2="a3 a5" order="1" />
</bondArray>
<propertyList>
  <property title="program">
    <scalar>Gaussian 16, Revision C.02</scalar>
  </property>
  <property title="basis">
    <scalar>Aug-CC-pVTZ (5D, 7F)</scalar>
  </property>
  <property title="File Format">
    <scalar>g03</scalar>
  </property>
  <property title="Energy" dictRef="me:ZPE">
    <scalar units="kcal/mol" >2.6</scalar>
  </property>
  <property title="SpinMultiplicity" dictRef="me:spinMultiplicity">
    <scalar>2.00</scalar>
  </property>
  <property title="Vibrational Frequencies" dictRef="me:vibFreqs">
    <array units="cm-1">209.27 325.89 340.52 429.76 501.99 642.97 687.18 778.64 900.73 1025.38
1084.74 1308.29 1444.45 1491.46 1757.93 3225.87 3340.13 3507.38</array>
  </property>
  <property title="Rotational Constants" dictRef="me:rotConsts">
    <array units="cm-1">0.380 0.155 0.110</array>
  </property>
  <property title="Symmetry Number" dictRef="me:symmetryNumber">
    <scalar>1</scalar>
  </property>
  <property dictRef="me:sigma" >
    <scalar>5.40</scalar>
  </property>
  <property dictRef="me:epsilon" >
    <scalar>307.0</scalar>
  </property>
  <property dictRef="me:frequenciesScaleFactor" >
    <scalar>1</scalar>
  </property>
</propertyList>
<me:DOSCMethod  name="ClassicalRotors" />
```

<me:DistributionCalcMethod name="Boltzmann" />
<me:energyTransferModel name="ExponentialDown" />
<me:deltaEDown >225.0</me:deltaEDown>
</molecule>
<molecule id="14Hshift-TS" spinMultiplicity="2">
<atomArray>
<atom id="a1" elementType="C" x3="0.000000" y3="0.583442" z3="0.000000" />
<atom id="a2" elementType="O" x3="0.244666" y3="1.747616" z3="0.000000" />
<atom id="a3" elementType="C" spinMultiplicity="2" x3="-1.350373" y3="-0.066108" z3="0.000000" />
<atom id="a4" elementType="O" x3="0.973883" y3="-0.366911" z3="0.000000" />
<atom id="a5" elementType="O" x3="0.374846" y3="-1.632600" z3="0.000000" />
<atom id="a6" elementType="H" x3="-1.922334" y3="0.076256" z3="0.909061" />
<atom id="a7" elementType="H" x3="-0.800250" y3="-1.241358" z3="0.000000" />
<atom id="a8" elementType="H" x3="-1.922334" y3="0.076256" z3="-0.909061" />
</atomArray>
<bondArray>
<bond atomRefs2="a8 a3" order="1" />
<bond atomRefs2="a1 a2" order="2" />
<bond atomRefs2="a1 a3" order="1" />
<bond atomRefs2="a1 a4" order="1" />
<bond atomRefs2="a3 a6" order="1" />
<bond atomRefs2="a4 a5" order="1" />
<bond atomRefs2="a5 a7" order="1" />
</bondArray>
<propertyList>
<property title="program">
<scalar>Gaussian 16, Revision C.02</scalar>
</property>
<property title="basis">
<scalar>Aug-CC-pVTZ (5D, 7F)</scalar>
</property>
<property title="File Format">
<scalar>g03</scalar>
</property>
<property title="Energy" dictRef="me:ZPE">
<scalar units="kcal/mol" >29.6</scalar>
</property>
<property title="SpinMultiplicity" dictRef="me:spinMultiplicity">
<scalar>2.00</scalar>
</property>
<property title="Vibrational Frequencies" dictRef="me:vibFreqs">
<array units="cm-1">130.40 269.53 489.20 539.65 612.68 686.25 865.66 1024.10 1026.56 1033.63 1095.48 1199.02 1404.19 1741.14 1920.21 3156.26 3240.52</array>
</property>
<property title="ImaginaryFrequency" dictRef="me:imFreqs">

<scalar units="cm-1">1903.81</scalar>
</property>
<property title="Rotational Constants" dictRef="me:rotConsts">
<array units="cm-1">0.345 0.165 0.114</array>
</property>
<property title="Symmetry Number" dictRef="me:symmetryNumber">
<scalar>1</scalar>
</property>
<property dictRef="me:frequenciesScaleFactor" >
<scalar>1</scalar>
</property>
</propertyList>
<me:DOSCMethod name="ClassicalRotors" />
</molecule>
<molecule id="Ethanal-AcylOO-rad" spinMultiplicity="2">
<atomArray>
<atom id="a1" elementType="C" x3="-1.441707" y3="0.186549" z3="0.000000" />
<atom id="a2" elementType="H" x3="-1.672779" y3="-0.417565" z3="0.874873" />
<atom id="a3" elementType="H" x3="-2.020645" y3="1.103483" z3="0.000000" />
<atom id="a4" elementType="H" x3="-1.672779" y3="-0.417565" z3="-0.874873" />
<atom id="a5" elementType="C" x3="-0.000000" y3="0.558925" z3="0.000000" />
<atom id="a6" elementType="O" x3="0.491666" y3="1.638754" z3="0.000000" />
<atom id="a7" elementType="O" x3="0.916874" y3="-0.528970" z3="0.000000" />
<atom id="a8" elementType="O" spinMultiplicity="2" x3="0.343516" y3="-1.702434" z3="0.000000" />
</atomArray>
<bondArray>
<bond atomRefs2="a4 a1" order="1" />
<bond atomRefs2="a1 a3" order="1" />
<bond atomRefs2="a1 a5" order="1" />
<bond atomRefs2="a1 a2" order="1" />
<bond atomRefs2="a5 a6" order="2" />
<bond atomRefs2="a5 a7" order="1" />
<bond atomRefs2="a7 a8" order="1" />
</bondArray>
<propertyList>
<property title="program">
<scalar>Gaussian 16, Revision C.02</scalar>
</property>
<property title="basis">
<scalar>Aug-CC-pVTZ (5D, 7F)</scalar>
</property>
<property title="File Format">
<scalar>g03</scalar>
</property>
<property title="Energy" dictRef="me:ZPE">

<scalar units="kcal/mol" >0.0</scalar>
</property>
<property title="SpinMultiplicity" dictRef="me:spinMultiplicity">
<scalar>2.00</scalar>
</property>
<property title="Vibrational Frequencies" dictRef="me:vibFreqs">
<array units="cm-1">143.92 211.58 339.42 500.44 543.44 574.84 786.97 993.82 1038.80 1178.35 1255.80 1386.05 1442.38 1450.85 1925.95 3099.79 3168.56 3208.93</array>
</property>
<property title="Rotational Constants" dictRef="me:rotConsts">
<array units="cm-1">0.314 0.164 0.110</array>
</property>
<property title="Symmetry Number" dictRef="me:symmetryNumber">
<scalar>1</scalar>
</property>
<property dictRef="me:sigma" >
<scalar>5.40</scalar>
</property>
<property dictRef="me:epsilon" >
<scalar>307.0</scalar>
</property>
<property dictRef="me:frequenciesScaleFactor" >
<scalar>1</scalar>
</property>
</propertyList>
<me:DOSCMethod name="ClassicalRotors" />
<me:DistributionCalcMethod name="Boltzmann" />
<me:energyTransferModel name="ExponentialDown" />
<me:deltaEDown >225.0</me:deltaEDown>
</molecule>
</moleculeList>
<reactionList>
<reaction id="R1">
<reactantList>
<reactant>
<molecule ref="Ethanal-AcylOO-rad" role="modelled" />
</reactant>
</reactantList>
<productList>
<product>
<molecule ref="Ethanal-14-rad" role="sink" />
</product>
</productList>
<me:transitionState>
<molecule ref="14Hshift-TS" role="transitionState" />
</me:transitionState>

```
    <me:MCRCMethod name="RRKM" />
            <me:tunneling name="Eckart" />
  </reaction>
</reactionList>
<me:conditions>
  <me:bathGas>N2</me:bathGas>
  <me:PTs>
    <me:PTpair units="Torr" P="760." T="298." precision="qd"  bathGas="N2" />
    <!--<me:PTpair units="Torr" P="201.60" T="298." />-->
    <!--<me:PTpair units="Torr" P="10.06" T="298." />-->
    <!--<me:PTpair units="Torr" P="15.01" T="298." />-->
  </me:PTs>
        <me:InitialPopulation>
    <me:molecule ref="Ethanal-AcylOO-rad" population="1.0" />
  </me:InitialPopulation>
</me:conditions>
<me:modelParameters>
  <!--Specify grain size directly...-->
  <me:grainSize units="cm-1">100</me:grainSize>
  <!--...or by the total number of grains
     <me:numberOfGrains> 500 </me:numberOfGrains>-->
  <!--Specify increased energy range
     <me:maxTemperature>6000</me:maxTemperature>-->
  <me:energyAboveTheTopHill>25.0</me:energyAboveTheTopHill>
</me:modelParameters>
<me:control>
  <me:printSpeciesProfile />
  <!--<me:testMicroRates />
        <me:testDOS />
  <me:testRateConstant />
  <me:printGrainDOS />-->
  <!--<me:printCellDOS />-->
  <!--<me:printReactionOperatorColumnSums />-->
  <!--<me:printTunnellingCoefficients />
  <me:printGrainkfE />-->
  <!--<me:printGrainBoltzmann />
  <me:printGrainkbE />-->
  <me:eigenvalues>3</me:eigenvalues>
  <!-- <me:hideInactive/>  Molecules and reactions with attribute active="false" are not shown
  <me:diagramEnergyOffset>0</me:diagramEnergyOffset>-->
  <!--Adjusts displayed energies to this values for the lowest species. -->
  <me:calcMethod  name="simpleCalc" />
  <me:ForceMacroDetailedBalance >true</me:ForceMacroDetailedBalance>
</me:control>
<metadataList xmlns:dc="http://purl.org/dc/elements/1.1/">
  <dc:title>Project name</dc:title>
```

```
    <dc:source>Acylperoxyrad-Ethanal.xml</dc:source>
    <dc:creator>Mesmer v6.0</dc:creator>
    <dc:date>20230618_144216</dc:date>
    <dc:contributor>ndprse</dc:contributor>
  </metadataList>
</me:mesmer>
```


Propanal–AcOO ------> Propanal–AcOOH


```
<?xml version="1.0" encoding="utf-8" ?>
<?xml-stylesheet type='text/xsl' href='../../mesmer2.xsl' media='other'?>
<?xml-stylesheet type='text/xsl' href='../../mesmer1.xsl' media='screen'?>
<me:mesmer                                        xmlns="http://www.xml-cml.org/schema"
xmlns:me="http://www.chem.leeds.ac.uk/mesmer"
xmlns:xsi="http://www.w3.org/2001/XMLSchema-instance">
  <me:title>Project name</me:title>
  <moleculeList convention="">
    <molecule id="N2">
    <atomArray>
      <atom id="a1" elementType="N" />
      <atom id="a2" elementType="N" />
    </atomArray>
    <bondArray>
      <bond atomRefs2="a2 a1" order="3"/>
    </bondArray>
    <propertyList>
      <property dictRef="me:epsilon">
        <scalar>48.0</scalar>
      </property>
      <property dictRef="me:sigma">
        <scalar>3.90</scalar>
      </property>
      <property dictRef="me:MW">
        <scalar units="amu">28.0</scalar>
      </property>
    </propertyList>
  </molecule>
    <molecule id="Propanal-14-rad" spinMultiplicity="2">
      <atomArray>
        <atom id="a1" elementType="C" x3="-0.058002" y3="0.066469" z3="-0.000144" />
        <atom id="a2" elementType="O" x3="-0.219161" y3="1.271229" z3="-0.000251" />
        <atom  id="a3"  elementType="C"  spinMultiplicity="2"  x3="1.187978"  y3="-0.647388"  z3="-
0.000004" />
        <atom id="a4" elementType="O" x3="-1.113721" y3="-0.783641" z3="0.000086" />
        <atom id="a5" elementType="H" x3="1.146638" y3="-1.727506" z3="0.000194" />
```

```
<atom id="a6" elementType="C" x3="2.482763" y3="0.060148" z3="0.000080" />
<atom id="a7" elementType="H" x3="3.074318" y3="-0.221806" z3="0.875729" />
<atom id="a8" elementType="H" x3="3.075582" y3="-0.223822" z3="-0.874031" />
<atom id="a9" elementType="H" x3="2.343236" y3="1.138285" z3="-0.001166" />
<atom id="a10" elementType="O" x3="-2.330746" y3="-0.074036" z3="0.000113" />
<atom id="a11" elementType="H" x3="-2.007196" y3="0.851050" z3="0.000088" />
</atomArray>
<bondArray>
<bond atomRefs2="a8 a6" order="1" />
<bond atomRefs2="a9 a6" order="1" />
<bond atomRefs2="a2 a1" order="2" />
<bond atomRefs2="a1 a3" order="1" />
<bond atomRefs2="a1 a4" order="1" />
<bond atomRefs2="a3 a6" order="1" />
<bond atomRefs2="a3 a5" order="1" />
<bond atomRefs2="a6 a7" order="1" />
<bond atomRefs2="a4 a10" order="1" />
<bond atomRefs2="a11 a10" order="1" />
</bondArray>
<propertyList>
<property title="program">
<scalar>Gaussian 16, Revision C.01</scalar>
</property>
<property title="basis">
<scalar>Aug-CC-pVTZ (5D, 7F)</scalar>
</property>
<property title="File Format">
<scalar>g03</scalar>
</property>
<property title="Energy" dictRef="me:ZPE">
<scalar units="kcal/mol">-2.75</scalar>
</property>
<property title="SpinMultiplicity" dictRef="me:spinMultiplicity">
<scalar>2.00</scalar>
</property>
<property title="Vibrational Frequencies" dictRef="me:vibFreqs">
<array units="cm-1">85.91 118.81 215.18 218.44 390.74 467.83 499.33 605.19 702.84 744.87
927.99 993.18 1004.26 1090.90 1156.55 1211.62 1404.21 1438.17 1476.12 1505.10 1533.05 1752.28
3028.46 3075.98 3155.81 3216.65 3539.89</array>
</property>
<property title="Rotational Constants" dictRef="me:rotConsts">
<array units="cm-1">0.354 0.073 0.062</array>
</property>
<property title="Symmetry Number" dictRef="me:symmetryNumber">
<scalar>1</scalar>
</property>
```

</propertyList>
</molecule>
<molecule id="14Hshift-TS" spinMultiplicity="2">
<atomArray>
<atom id="a1" elementType="C" x3="0.273710" y3="0.650626" z3="0.083502" />
<atom id="a2" elementType="O" x3="0.145549" y3="1.827929" z3="-0.012879" />
<atom id="a3" elementType="C" x3="-0.761926" y3="-0.352274" z3="0.524879" />
<atom id="a4" elementType="O" x3="1.438266" y3="0.022386" z3="-0.216026" />
<atom id="a5" elementType="O" spinMultiplicity="2" x3="1.268616" y3="-1.362571" z3="-0.040690" />
<atom id="a6" elementType="H" x3="-0.930195" y3="-0.315651" z3="1.599266" />
<atom id="a7" elementType="H" x3="0.050674" y3="-1.310465" z3="0.329910" />
<atom id="a8" elementType="C" x3="-2.005140" y3="-0.447497" z3="-0.311085" />
<atom id="a9" elementType="H" x3="-2.563130" y3="0.489297" z3="-0.242028" />
<atom id="a10" elementType="H" x3="-1.768301" y3="-0.620723" z3="-1.359509" />
<atom id="a11" elementType="H" x3="-2.648368" y3="-1.249536" z3="0.045347" />
</atomArray>
<bondArray>
<bond atomRefs2="a10 a8" order="1" />
<bond atomRefs2="a8 a9" order="1" />
<bond atomRefs2="a8 a11" order="1" />
<bond atomRefs2="a8 a3" order="1" />
<bond atomRefs2="a4 a5" order="1" />
<bond atomRefs2="a4 a1" order="1" />
<bond atomRefs2="a2 a1" order="2" />
<bond atomRefs2="a1 a3" order="1" />
<bond atomRefs2="a7 a3" order="1" />
<bond atomRefs2="a3 a6" order="1" />
</bondArray>
<propertyList>
<property title="program">
<scalar>Gaussian 16, Revision C.01</scalar>
</property>
<property title="basis">
<scalar>Aug-CC-pVTZ (5D, 7F)</scalar>
</property>
<property title="File Format">
<scalar>g03</scalar>
</property>
<property title="Energy" dictRef="me:ZPE">
<scalar units="kcal/mol">26.79</scalar>
</property>
<property title="SpinMultiplicity" dictRef="me:spinMultiplicity">
<scalar>2.00</scalar>
</property>
<property title="Vibrational Frequencies" dictRef="me:vibFreqs">

<array units="cm-1">79.65 180.88 196.44 247.34 502.32 562.36 673.81 690.16 800.16 819.13 1016.54 1043.57 1081.24 1129.30 1179.07 1188.00 1339.19 1411.38 1487.14 1495.06 1770.18 1917.31 3047.98 3119.88 3132.89 3152.86</array>
</property>
<property title="ImaginaryFrequency" dictRef="me:imFreqs">
<scalar units="cm-1">1629.08</scalar>
</property>
<property title="Rotational Constants" dictRef="me:rotConsts">
<array units="cm-1">0.159 0.118 0.074</array>
</property>
<property title="Symmetry Number" dictRef="me:symmetryNumber">
<scalar>1</scalar>
</property>
<property dictRef="me:frequenciesScaleFactor" >
<scalar>1</scalar>
</property>
</propertyList>
<me:DOSCMethod name="ClassicalRotors" />
</molecule>
<molecule id="Propanal-15-rad" spinMultiplicity="2">
<atomArray>
<atom id="a1" elementType="C" x3="0.031465" y3="0.083762" z3="0.065888" />
<atom id="a2" elementType="O" x3="0.190152" y3="1.275561" z3="0.113709" />
<atom id="a3" elementType="C" x3="-1.267891" y3="-0.668025" z3="0.169634" />
<atom id="a4" elementType="H" x3="-1.197615" y3="-1.583896" z3="-0.421097" />
<atom id="a5" elementType="H" x3="-1.333026" y3="-1.007878" z3="1.213990" />
<atom id="a6" elementType="C" spinMultiplicity="2" x3="-2.445253" y3="0.150905" z3="-0.200434" />
<atom id="a7" elementType="O" x3="1.061810" y3="-0.765450" z3="-0.064251" />
<atom id="a8" elementType="O" x3="2.297616" y3="-0.079784" z3="-0.098100" />
<atom id="a9" elementType="H" x3="-2.369692" y3="1.225591" z3="-0.249448" />
<atom id="a10" elementType="H" x3="1.998096" y3="0.849842" z3="-0.038098" />
<atom id="a11" elementType="H" x3="-3.404310" y3="-0.326136" z3="-0.326733" />
</atomArray>
<bondArray>
<bond atomRefs2="a4 a3" order="1" />
<bond atomRefs2="a11 a6" order="1" />
<bond atomRefs2="a9 a6" order="1" />
<bond atomRefs2="a6 a3" order="1" />
<bond atomRefs2="a8 a7" order="1" />
<bond atomRefs2="a8 a10" order="1" />
<bond atomRefs2="a7 a1" order="1" />
<bond atomRefs2="a1 a2" order="2" />
<bond atomRefs2="a1 a3" order="1" />
<bond atomRefs2="a3 a5" order="1" />
</bondArray>

<propertyList>
<property title="program">
<scalar>Gaussian 16, Revision C.01</scalar>
</property>
<property title="basis">
<scalar>Aug-CC-pVTZ (5D, 7F)</scalar>
</property>
<property title="File Format">
<scalar>g03</scalar>
</property>
<property title="Energy" dictRef="me:ZPE">
<scalar units="kcal/mol">5.22</scalar>
</property>
<property title="SpinMultiplicity" dictRef="me:spinMultiplicity">
<scalar>2.00</scalar>
</property>
<property title="Vibrational Frequencies" dictRef="me:vibFreqs">
<array units="cm-1">35.59 160.62 192.34 244.89 376.31 443.91 471.61 474.43 611.30 686.74 902.50 935.52 1004.06 1045.79 1122.72 1194.38 1239.11 1403.37 1444.93 1456.77 1524.80 1829.28 2981.39 3078.37 3181.75 3296.63 3567.84</array>
</property>
<property title="Rotational Constants" dictRef="me:rotConsts">
<array units="cm-1">0.336 0.076 0.063</array>
</property>
<property title="Symmetry Number" dictRef="me:symmetryNumber">
<scalar>1</scalar>
</property>
</propertyList>
</molecule>
<molecule id="15Hshift-TS" spinMultiplicity="2">
<atomArray>
<atom id="a1" elementType="C" x3="-0.822449" y3="-0.142568" z3="0.030371" />
<atom id="a2" elementType="O" x3="-1.975823" y3="-0.391386" z3="-0.120103" />
<atom id="a3" elementType="C" x3="0.274903" y3="-1.137140" z3="0.364581" />
<atom id="a4" elementType="H" x3="0.420663" y3="-1.110569" z3="1.445302" />
<atom id="a5" elementType="H" x3="-0.121657" y3="-2.116471" z3="0.099894" />
<atom id="a6" elementType="C" spinMultiplicity="2" x3="1.560440" y3="-0.816210" z3="-0.342249" />
<atom id="a7" elementType="O" x3="-0.405446" y3="1.146366" z3="-0.182735" />
<atom id="a8" elementType="O" x3="0.889257" y3="1.419991" z3="0.251084" />
<atom id="a9" elementType="H" x3="2.476307" y3="-1.126164" z3="0.148068" />
<atom id="a10" elementType="H" x3="1.519795" y3="0.482030" z3="-0.175904" />
<atom id="a11" elementType="H" x3="1.563629" y3="-0.953083" z3="-1.419544" />
</atomArray>
<bondArray>
<bond atomRefs2="a11 a6" order="1" />

```
      <bond atomRefs2="a6 a9" order="1" />
      <bond atomRefs2="a6 a3" order="1" />
      <bond atomRefs2="a7 a1" order="1" />
      <bond atomRefs2="a7 a8" order="1" />
      <bond atomRefs2="a10 a8" order="1" />
      <bond atomRefs2="a2 a1" order="2" />
      <bond atomRefs2="a1 a3" order="1" />
      <bond atomRefs2="a5 a3" order="1" />
      <bond atomRefs2="a3 a4" order="1" />
    </bondArray>
    <propertyList>
      <property title="program">
        <scalar>Gaussian 16, Revision C.01</scalar>
      </property>
      <property title="basis">
        <scalar>Aug-CC-pVTZ (5D, 7F)</scalar>
      </property>
      <property title="File Format">
        <scalar>g03</scalar>
      </property>
      <property title="Energy" dictRef="me:ZPE">
        <scalar units="kcal/mol">24.54</scalar>
      </property>
      <property title="SpinMultiplicity" dictRef="me:spinMultiplicity">
        <scalar>2.00</scalar>
      </property>
      <property title="Vibrational Frequencies" dictRef="me:vibFreqs">
        <array units="cm-1">132.40 206.27 360.09 479.66 526.26 551.99 620.37 700.15 780.52 847.85
986.73 1056.44 1080.15 1112.84 1193.85 1212.10 1262.19 1330.06 1453.02 1473.83 1565.52 1877.05
3078.66 3117.34 3132.42 3212.65</array>
      </property>
      <property title="ImaginaryFrequency" dictRef="me:imFreqs">
        <scalar units="cm-1">1798.87</scalar>
      </property>
      <property title="Rotational Constants" dictRef="me:rotConsts">
        <array units="cm-1">0.176 0.124 0.079</array>
      </property>
      <property title="Symmetry Number" dictRef="me:symmetryNumber">
        <scalar>1</scalar>
      </property>
      <property dictRef="me:frequenciesScaleFactor" >
        <scalar>1</scalar>
      </property>
    </propertyList>
    <me:DOSCMethod  name="ClassicalRotors" />
  </molecule>
```

<molecule id="Propanal-AcylOO-rad" spinMultiplicity="2">
<atomArray>
<atom id="a1" elementType="C" x3="-2.065742" y3="-0.896490" z3="0.000000" />
<atom id="a2" elementType="H" x3="-2.429023" y3="-1.922372" z3="0.000000" />
<atom id="a3" elementType="H" x3="-2.460857" y3="-0.390792" z3="0.878971" />
<atom id="a4" elementType="H" x3="-2.460857" y3="-0.390792" z3="-0.878971" />
<atom id="a5" elementType="C" x3="-0.545728" y3="-0.888667" z3="0.000000" />
<atom id="a6" elementType="H" x3="-0.135805" y3="-1.410024" z3="0.865943" />
<atom id="a7" elementType="H" x3="-0.135805" y3="-1.410024" z3="-0.865943" />
<atom id="a8" elementType="C" x3="0.000000" y3="0.504177" z3="0.000000" />
<atom id="a9" elementType="O" x3="-0.586155" y3="1.530979" z3="0.000000" />
<atom id="a10" elementType="O" x3="1.427433" y3="0.628193" z3="0.000000" />
<atom id="a11" elementType="O" spinMultiplicity="2" x3="2.070117" y3="-0.507937" z3="0.000000" />
</atomArray>
<bondArray>
<bond atomRefs2="a4 a1" order="1" />
<bond atomRefs2="a7 a5" order="1" />
<bond atomRefs2="a1 a2" order="1" />
<bond atomRefs2="a1 a5" order="1" />
<bond atomRefs2="a1 a3" order="1" />
<bond atomRefs2="a5 a8" order="1" />
<bond atomRefs2="a5 a6" order="1" />
<bond atomRefs2="a8 a9" order="2" />
<bond atomRefs2="a8 a10" order="1" />
<bond atomRefs2="a10 a11" order="1" />
</bondArray>
<propertyList>
<property title="program">
<scalar>Gaussian 16, Revision C.01</scalar>
</property>
<property title="basis">
<scalar>Aug-CC-pVTZ (5D, 7F)</scalar>
</property>
<property title="File Format">
<scalar>g03</scalar>
</property>
<property title="Energy" dictRef="me:ZPE">
<scalar units="kcal/mol">0.0</scalar>
</property>
<property title="SpinMultiplicity" dictRef="me:spinMultiplicity">
<scalar>2.00</scalar>
</property>
<property title="Vibrational Frequencies" dictRef="me:vibFreqs">

```
        <array units="cm-1">95.05 137.99 219.09 234.67 332.22 526.80 553.24 602.71 766.85 813.39
1012.89 1057.92 1108.82 1120.59 1251.13 1285.07 1383.71 1432.36 1454.37 1503.45 1507.84 1923.28
3070.82 3083.19 3115.45 3149.46 3151.42</array>
      </property>
      <property title="Rotational Constants" dictRef="me:rotConsts">
        <array units="cm-1">0.216 0.091 0.066</array>
      </property>
      <property title="Symmetry Number" dictRef="me:symmetryNumber">
        <scalar>1</scalar>
      </property>
      <property dictRef="me:sigma" >
        <scalar>5.85</scalar>
      </property>
      <property dictRef="me:epsilon" >
        <scalar>327.0</scalar>
      </property>
      <property dictRef="me:frequenciesScaleFactor" >
        <scalar>1</scalar>
      </property>
    </propertyList>
    <me:DOSCMethod  name="ClassicalRotors" />
    <me:DistributionCalcMethod  name="Boltzmann" />
    <me:energyTransferModel name="ExponentialDown"  />
    <me:deltaEDown >225.0</me:deltaEDown>
  </molecule>
</moleculeList>
<reactionList>
  <reaction id="R1" >
        <!--reversible="true"-->
    <reactantList>
      <reactant>
        <molecule ref="Propanal-AcylOO-rad" role="modelled" />
      </reactant>
    </reactantList>
    <productList>
      <product>
        <molecule ref="Propanal-14-rad" role="sink" />
      </product>
    </productList>
    <me:transitionState>
      <molecule ref="14Hshift-TS" role="transitionState" />
    </me:transitionState>
    <me:MCRCMethod name="RRKM" />
          <me:tunneling name="Eckart" />
  </reaction>
  <reaction id="R2" >
```

```
        <!--reversible="true"-->
    <reactantList>
      <reactant>
        <molecule ref="Propanal-AcylOO-rad" role="modelled" />
      </reactant>
    </reactantList>
    <productList>
      <product>
        <molecule ref="Propanal-15-rad" role="sink" />
      </product>
    </productList>
    <me:transitionState>
      <molecule ref="15Hshift-TS" role="transitionState" />
    </me:transitionState>
    <me:MCRCMethod name="RRKM" />
          <me:tunneling name="Eckart" />
  </reaction>
</reactionList>
<me:conditions>
  <me:bathGas>N2</me:bathGas>
  <me:PTs>
    <me:PTpair units="Torr" P="760." T="298." precision="qd"  bathGas="N2" />
  </me:PTs>
</me:conditions>
<me:modelParameters>
  <!--Specify grain size directly...-->
  <me:grainSize units="cm-1">100</me:grainSize>
  <!--...or by the total number of grains
      <me:numberOfGrains> 500 </me:numberOfGrains>-->
  <!--Specify increased energy range
      <me:maxTemperature>6000</me:maxTemperature>-->
  <me:energyAboveTheTopHill>25.0</me:energyAboveTheTopHill>
</me:modelParameters>
<me:control>
  <me:printSpeciesProfile />
  <!--<me:testMicroRates />
        <me:testDOS />
  <me:testRateConstant />
  <me:printGrainDOS />-->
  <!--<me:printCellDOS />-->
  <!--<me:printReactionOperatorColumnSums />-->
  <!--<me:printTunnellingCoefficients />
  <me:printGrainkfE />-->
  <!--<me:printGrainBoltzmann />
  <me:printGrainkbE />-->
  <me:eigenvalues>3</me:eigenvalues>
```

```
    <!-- <me:hideInactive/>  Molecules and reactions with attribute active="false" are not shown
    <me:diagramEnergyOffset>0</me:diagramEnergyOffset>-->
    <!--Adjusts displayed energies to this values for the lowest species. -->
    <me:calcMethod  name="simpleCalc" />
    <me:ForceMacroDetailedBalance >true</me:ForceMacroDetailedBalance>
  </me:control>
  <metadataList xmlns:dc="http://purl.org/dc/elements/1.1/">
    <dc:title>Project name</dc:title>
    <dc:source>Acylperoxyrad-Propanal.xml</dc:source>
    <dc:creator>Mesmer v6.0</dc:creator>
    <dc:date>20220122_135250</dc:date>
    <dc:contributor>ndprse</dc:contributor>
  </metadataList>
</me:mesmer>
```


Butanal–AcOO ------> Butanal–AcOOH


```
<?xml version="1.0" encoding="utf-8" ?>
<?xml-stylesheet type='text/xsl' href='../../mesmer2.xsl' media='other'?>
<?xml-stylesheet type='text/xsl' href='../../mesmer1.xsl' media='screen'?>
<me:mesmer                                                   xmlns="http://www.xml-cml.org/schema"
xmlns:me="http://www.chem.leeds.ac.uk/mesmer"
xmlns:xsi="http://www.w3.org/2001/XMLSchema-instance">
  <me:title>Project name</me:title>
  <moleculeList convention="">
    <molecule id="N2">
    <atomArray>
      <atom id="a1" elementType="N" />
      <atom id="a2" elementType="N" />
    </atomArray>
    <bondArray>
      <bond atomRefs2="a2 a1" order="3"/>
    </bondArray>
    <propertyList>
      <property dictRef="me:epsilon">
        <scalar>48.0</scalar>
      </property>
      <property dictRef="me:sigma">
        <scalar>3.90</scalar>
      </property>
      <property dictRef="me:MW">
        <scalar units="amu">28.0</scalar>
      </property>
    </propertyList>
  </molecule>
```

<molecule id="Butanal-15-rad" spinMultiplicity="2">
<atomArray>
<atom id="a1" elementType="C" x3="0.488277" y3="0.042843" z3="0.364443" />
<atom id="a2" elementType="O" x3="0.580958" y3="1.239245" z3="0.476282" />
<atom id="a3" elementType="C" x3="-0.714871" y3="-0.805696" z3="0.702382" />
<atom id="a4" elementType="H" x3="-0.404351" y3="-1.844390" z3="0.800000" />
<atom id="a5" elementType="H" x3="-1.085391" y3="-0.448332" z3="1.664992" />
<atom id="a6" elementType="C" spinMultiplicity="2" x3="-1.752923" y3="-0.644086" z3="-
0.359795" />
<atom id="a7" elementType="C" x3="-2.626159" y3="0.552480" z3="-0.391028" />
<atom id="a8" elementType="H" x3="-3.406497" y3="0.460031" z3="-1.144021" />
<atom id="a9" elementType="H" x3="-3.098649" y3="0.723913" z3="0.580507" />
<atom id="a10" elementType="O" x3="1.479795" y3="-0.701455" z3="-0.147908" />
<atom id="a11" elementType="H" x3="-1.700432" y3="-1.300402" z3="-1.217178" />
<atom id="a12" elementType="H" x3="-2.051674" y3="1.460936" z3="-0.613227" />
<atom id="a13" elementType="O" x3="2.579163" y3="0.098064" z3="-0.538770" />
<atom id="a14" elementType="H" x3="2.261723" y3="0.988158" z3="-0.283910" />
</atomArray>
<bondArray>
<bond atomRefs2="a11 a6" order="1" />
<bond atomRefs2="a8 a7" order="1" />
<bond atomRefs2="a12 a7" order="1" />
<bond atomRefs2="a13 a14" order="1" />
<bond atomRefs2="a13 a10" order="1" />
<bond atomRefs2="a7 a6" order="1" />
<bond atomRefs2="a7 a9" order="1" />
<bond atomRefs2="a6 a3" order="1" />
<bond atomRefs2="a10 a1" order="1" />
<bond atomRefs2="a1 a2" order="2" />
<bond atomRefs2="a1 a3" order="1" />
<bond atomRefs2="a3 a4" order="1" />
<bond atomRefs2="a3 a5" order="1" />
</bondArray>
<propertyList>
<property title="program">
<scalar>Gaussian 16, Revision C.01</scalar>
</property>
<property title="basis">
<scalar>Aug-CC-pVTZ (5D, 7F)</scalar>
</property>
<property title="File Format">
<scalar>g03</scalar>
</property>
<property title="Energy" dictRef="me:ZPE">
<scalar units="kcal/mol">2.35</scalar>
</property>

<property title="SpinMultiplicity" dictRef="me:spinMultiplicity">
<scalar>2.00</scalar>
</property>
<property title="Vibrational Frequencies" dictRef="me:vibFreqs">
<array units="cm-1">40.29 72.53 115.97 167.11 265.33 301.77 387.22 442.75 444.76 488.82 650.72 790.77 882.21 917.15 959.95 998.30 1024.33 1093.19 1155.33 1215.42 1283.08 1328.53 1389.87 1425.68 1470.57 1478.29 1487.49 1517.81 1813.55 2997.91 3055.07 3064.69 3131.11 3133.99 3205.11 3557.11</array>
</property>
<property title="Rotational Constants" dictRef="me:rotConsts">
<array units="cm-1">0.199 0.050 0.046</array>
</property>
<property title="Symmetry Number" dictRef="me:symmetryNumber">
<scalar>1</scalar>
</property>
</propertyList>
</molecule>
<molecule id="15Hshift-TS" spinMultiplicity="2">
<atomArray>
<atom id="a1" elementType="C" x3="-1.026384" y3="-0.389335" z3="0.029845" />
<atom id="a2" elementType="O" x3="-1.865319" y3="-1.153816" z3="-0.328541" />
<atom id="a3" elementType="C" x3="0.143233" y3="-0.697869" z3="0.944327" />
<atom id="a4" elementType="H" x3="-0.128525" y3="-0.370473" z3="1.948551" />
<atom id="a5" elementType="H" x3="0.243025" y3="-1.783099" z3="0.952230" />
<atom id="a6" elementType="C" spinMultiplicity="2" x3="1.402474" y3="-0.001358" z3="0.490475" />
<atom id="a7" elementType="C" x3="2.030001" y3="-0.483853" z3="-0.785841" />
<atom id="a8" elementType="H" x3="2.417269" y3="-1.498751" z3="-0.653071" />
<atom id="a9" elementType="H" x3="1.304719" y3="-0.515976" z3="-1.600144" />
<atom id="a10" elementType="O" x3="-1.049265" y3="0.882803" z3="-0.481374" />
<atom id="a11" elementType="O" x3="-0.203569" y3="1.781717" z3="0.162338" />
<atom id="a12" elementType="H" x3="2.100553" y3="0.224756" z3="1.292481" />
<atom id="a13" elementType="H" x3="0.853552" y3="1.139040" z3="0.255751" />
<atom id="a14" elementType="H" x3="2.858687" y3="0.153369" z3="-1.088015" />
</atomArray>
<bondArray>
<bond atomRefs2="a9 a7" order="1" />
<bond atomRefs2="a14 a7" order="1" />
<bond atomRefs2="a7 a8" order="1" />
<bond atomRefs2="a7 a6" order="1" />
<bond atomRefs2="a10 a1" order="1" />
<bond atomRefs2="a10 a11" order="1" />
<bond atomRefs2="a2 a1" order="2" />
<bond atomRefs2="a1 a3" order="1" />
<bond atomRefs2="a11 a13" order="1" />
<bond atomRefs2="a6 a3" order="1" />

```
    <bond atomRefs2="a6 a12" order="1" />
    <bond atomRefs2="a3 a5" order="1" />
    <bond atomRefs2="a3 a4" order="1" />
   </bondArray>
   <propertyList>
    <property title="program">
     <scalar>Gaussian 16, Revision C.01</scalar>
    </property>
    <property title="basis">
     <scalar>Aug-CC-pVTZ (5D, 7F)</scalar>
    </property>
    <property title="File Format">
     <scalar>g03</scalar>
    </property>
    <property title="Energy" dictRef="me:ZPE">
     <scalar units="kcal/mol">20.95</scalar>
    </property>
    <property title="SpinMultiplicity" dictRef="me:spinMultiplicity">
     <scalar>2.00</scalar>
    </property>
    <property title="Vibrational Frequencies" dictRef="me:vibFreqs">
     <array units="cm-1">100.40 131.71 193.57 243.69 340.65 480.23 494.21 544.27 591.40 687.78
778.03 859.22 899.82 932.65 1042.04 1072.43 1106.57 1143.83 1195.13 1224.41 1251.10 1318.52
1372.02 1423.51 1457.16 1487.66 1497.24 1570.30 1874.76 3030.88 3076.03 3099.48 3128.02 3133.63
3146.41</array>
    </property>
    <property title="ImaginaryFrequency" dictRef="me:imFreqs">
     <scalar units="cm-1">1716.03</scalar>
    </property>
    <property title="Rotational Constants" dictRef="me:rotConsts">
     <array units="cm-1">0.121 0.077 0.060</array>
    </property>
    <property title="Symmetry Number" dictRef="me:symmetryNumber">
     <scalar>1</scalar>
    </property>
    <property dictRef="me:frequenciesScaleFactor" >
     <scalar>1</scalar>
    </property>
   </propertyList>
   <me:DOSCMethod name="ClassicalRotors" />
  </molecule>
  <molecule id="Butanal-16-rad" spinMultiplicity="2">
   <atomArray>
    <atom id="a1" elementType="C" x3="0.589404" y3="0.109965" z3="0.116190" />
    <atom id="a2" elementType="O" x3="0.843454" y3="1.284318" z3="0.206404" />
    <atom id="a3" elementType="C" x3="-0.758280" y3="-0.537267" z3="0.242670" />
```

<atom id="a4" elementType="H" x3="-0.750140" y3="-1.492080" z3="-0.280963" />
<atom id="a5" elementType="H" x3="-0.898807" y3="-0.758997" z3="1.304104" />
<atom id="a6" elementType="C" x3="-1.885216" y3="0.365544" z3="-0.253764" />
<atom id="a7" elementType="H" x3="-1.735256" y3="0.593417" z3="-1.312747" />
<atom id="a8" elementType="H" x3="-1.806276" y3="1.332582" z3="0.258855" />
<atom id="a9" elementType="C" spinMultiplicity="2" x3="-3.226133" y3="-0.229683" z3="-0.041973" />
<atom id="a10" elementType="O" x3="1.547130" y3="-0.805230" z3="-0.099651" />
<atom id="a11" elementType="O" x3="2.827902" y3="-0.210406" z3="-0.164565" />
<atom id="a12" elementType="H" x3="-3.392544" y3="-0.965897" z3="0.732231" />
<atom id="a13" elementType="H" x3="2.601412" y3="0.734427" z3="-0.046996" />
<atom id="a14" elementType="H" x3="-4.084929" y3="0.155741" z3="-0.570715" />
</atomArray>
<bondArray>
<bond atomRefs2="a7 a6" order="1" />
<bond atomRefs2="a14 a9" order="1" />
<bond atomRefs2="a4 a3" order="1" />
<bond atomRefs2="a6 a9" order="1" />
<bond atomRefs2="a6 a3" order="1" />
<bond atomRefs2="a6 a8" order="1" />
<bond atomRefs2="a11 a10" order="1" />
<bond atomRefs2="a11 a13" order="1" />
<bond atomRefs2="a10 a1" order="1" />
<bond atomRefs2="a9 a12" order="1" />
<bond atomRefs2="a1 a2" order="2" />
<bond atomRefs2="a1 a3" order="1" />
<bond atomRefs2="a3 a5" order="1" />
</bondArray>
<propertyList>
<property title="program">
<scalar>Gaussian 16, Revision C.01</scalar>
</property>
<property title="basis">
<scalar>Aug-CC-pVTZ (5D, 7F)</scalar>
</property>
<property title="File Format">
<scalar>g03</scalar>
</property>
<property title="Energy" dictRef="me:ZPE">
<scalar units="kcal/mol">4.6</scalar>
</property>
<property title="SpinMultiplicity" dictRef="me:spinMultiplicity">
<scalar>2.00</scalar>
</property>
<property title="Vibrational Frequencies" dictRef="me:vibFreqs">

<array units="cm-1">26.11 91.04 122.38 149.29 241.53 344.63 350.91 442.62 478.61 482.89 632.24 739.92 784.75 902.80 960.00 1005.19 1035.51 1104.34 1121.66 1172.68 1241.00 1301.10 1356.60 1416.79 1471.42 1475.06 1480.38 1512.01 1822.02 3001.65 3052.53 3064.73 3126.79 3161.59 3267.78 3566.02</array>
</property>
<property title="Rotational Constants" dictRef="me:rotConsts">
<array units="cm-1">0.295 0.042 0.038</array>
</property>
<property title="Symmetry Number" dictRef="me:symmetryNumber">
<scalar>1</scalar>
</property>
</propertyList>
</molecule>
<molecule id="16Hshift-TS" spinMultiplicity="2">
<atomArray>
<atom id="a1" elementType="C" x3="-1.107266" y3="-0.186294" z3="0.036439" />
<atom id="a2" elementType="O" x3="-2.151752" y3="-0.622031" z3="-0.338675" />
<atom id="a3" elementType="C" x3="-0.013537" y3="-0.979569" z3="0.703821" />
<atom id="a4" elementType="H" x3="0.284656" y3="-0.507797" z3="1.639048" />
<atom id="a5" elementType="H" x3="-0.442201" y3="-1.954033" z3="0.919128" />
<atom id="a6" elementType="C" x3="1.206256" y3="-1.129776" z3="-0.232358" />
<atom id="a7" elementType="H" x3="0.857923" y3="-1.421923" z3="-1.222808" />
<atom id="a8" elementType="H" x3="1.813382" y3="-1.956549" z3="0.143780" />
<atom id="a9" elementType="C" spinMultiplicity="2" x3="2.057515" y3="0.111355" z3="-0.316200" />
<atom id="a10" elementType="O" x3="-0.836757" y3="1.125599" z3="-0.275854" />
<atom id="a11" elementType="O" x3="0.261393" y3="1.652072" z3="0.391975" />
<atom id="a12" elementType="H" x3="2.744682" y3="0.262416" z3="0.512668" />
<atom id="a13" elementType="H" x3="1.209612" y3="1.090876" z3="-0.078954" />
<atom id="a14" elementType="H" x3="2.491063" y3="0.347596" z3="-1.282640" />
</atomArray>
<bondArray>
<bond atomRefs2="a14 a9" order="1" />
<bond atomRefs2="a7 a6" order="1" />
<bond atomRefs2="a2 a1" order="2" />
<bond atomRefs2="a9 a6" order="1" />
<bond atomRefs2="a9 a12" order="1" />
<bond atomRefs2="a10 a1" order="1" />
<bond atomRefs2="a10 a11" order="1" />
<bond atomRefs2="a6 a8" order="1" />
<bond atomRefs2="a6 a3" order="1" />
<bond atomRefs2="a13 a11" order="1" />
<bond atomRefs2="a1 a3" order="1" />
<bond atomRefs2="a3 a5" order="1" />
<bond atomRefs2="a3 a4" order="1" />
</bondArray>

<propertyList>
<property title="program">
<scalar>Gaussian 16, Revision C.01</scalar>
</property>
<property title="basis">
<scalar>Aug-CC-pVTZ (5D, 7F)</scalar>
</property>
<property title="File Format">
<scalar>g03</scalar>
</property>
<property title="Energy" dictRef="me:ZPE">
<scalar units="kcal/mol">22.76</scalar>
</property>
<property title="SpinMultiplicity" dictRef="me:spinMultiplicity">
<scalar>2.00</scalar>
</property>
<property title="Vibrational Frequencies" dictRef="me:vibFreqs">
<array units="cm-1">84.50 188.28 227.76 331.03 393.55 459.04 513.47 581.24 641.73 721.01 767.95 886.14 898.79 933.53 1052.80 1061.61 1098.27 1117.25 1163.68 1220.28 1251.38 1297.27 1362.81 1371.95 1444.69 1468.58 1489.48 1498.88 1874.32 3050.47 3094.37 3104.76 3110.55 3162.39 3196.82</array>
</property>
<property title="ImaginaryFrequency" dictRef="me:imFreqs">
<scalar units="cm-1">1878.23</scalar>
</property>
<property title="Rotational Constants" dictRef="me:rotConsts">
<array units="cm-1">0.131 0.081 0.057</array>
</property>
<property title="Symmetry Number" dictRef="me:symmetryNumber">
<scalar>1</scalar>
</property>
<property dictRef="me:frequenciesScaleFactor" >
<scalar>1</scalar>
</property>
</propertyList>
<me:DOSCMethod name="ClassicalRotors" />
</molecule>
<molecule id="Butanal-AcylOO-rad" spinMultiplicity="2">
<atomArray>
<atom id="a1" elementType="C" x3="-1.561744" y3="2.519026" z3="0.000000" />
<atom id="a2" elementType="H" x3="-2.604241" y3="2.833907" z3="0.000000" />
<atom id="a3" elementType="H" x3="-1.083206" y3="2.949383" z3="0.881054" />
<atom id="a4" elementType="H" x3="-1.083206" y3="2.949383" z3="-0.881054" />
<atom id="a5" elementType="C" x3="-1.452454" y3="1.000379" z3="0.000000" />
<atom id="a6" elementType="H" x3="-1.960128" y3="0.586731" z3="0.871799" />
<atom id="a7" elementType="H" x3="-1.960128" y3="0.586731" z3="-0.871799" />

<atom id="a8" elementType="C" x3="0.000000" y3="0.543723" z3="0.000000" />
<atom id="a9" elementType="H" x3="0.541305" y3="0.929281" z3="-0.866332" />
<atom id="a10" elementType="H" x3="0.541305" y3="0.929281" z3="0.866332" />
<atom id="a11" elementType="C" x3="0.122519" y3="-0.946049" z3="0.000000" />
<atom id="a12" elementType="O" x3="-0.732495" y3="-1.762655" z3="0.000000" />
<atom id="a13" elementType="O" x3="1.455380" y3="-1.474761" z3="0.000000" />
<atom id="a14" elementType="O" spinMultiplicity="2" x3="2.396912" y3="-0.570979" z3="0.000000" />
</atomArray>
<bondArray>
<bond atomRefs2="a4 a1" order="1" />
<bond atomRefs2="a7 a5" order="1" />
<bond atomRefs2="a9 a8" order="1" />
<bond atomRefs2="a1 a2" order="1" />
<bond atomRefs2="a1 a5" order="1" />
<bond atomRefs2="a1 a3" order="1" />
<bond atomRefs2="a5 a8" order="1" />
<bond atomRefs2="a5 a6" order="1" />
<bond atomRefs2="a8 a11" order="1" />
<bond atomRefs2="a8 a10" order="1" />
<bond atomRefs2="a11 a12" order="2" />
<bond atomRefs2="a11 a13" order="1" />
<bond atomRefs2="a13 a14" order="1" />
</bondArray>
<propertyList>
<property title="program">
<scalar>Gaussian 16, Revision C.01</scalar>
</property>
<property title="basis">
<scalar>Aug-CC-pVTZ (5D, 7F)</scalar>
</property>
<property title="File Format">
<scalar>g03</scalar>
</property>
<property title="Energy" dictRef="me:ZPE">
<scalar units="kcal/mol">0.0</scalar>
</property>
<property title="SpinMultiplicity" dictRef="me:spinMultiplicity">
<scalar>2.00</scalar>
</property>
<property title="Vibrational Frequencies" dictRef="me:vibFreqs">
<array units="cm-1">78.03 103.47 139.47 156.21 251.57 304.63 366.30 530.20 562.11 591.26 752.98 826.26 889.29 931.07 1052.37 1074.42 1126.34 1143.83 1251.00 1257.41 1331.10 1335.83 1414.63 1427.83 1450.98 1499.31 1507.83 1514.25 1922.87 3047.03 3067.79 3075.72 3094.98 3109.16 3126.99 3131.93</array>
</property>

<property title="Rotational Constants" dictRef="me:rotConsts">
<array units="cm-1">0.167 0.051 0.040</array>
</property>
<property title="Symmetry Number" dictRef="me:symmetryNumber">
<scalar>1</scalar>
</property>
<property dictRef="me:sigma" >
<scalar>6.25</scalar>
</property>
<property dictRef="me:epsilon" >
<scalar>343.0</scalar>
</property>
<property dictRef="me:frequenciesScaleFactor" >
<scalar>1</scalar>
</property>
</propertyList>
<me:DOSCMethod name="ClassicalRotors" />
<me:DistributionCalcMethod name="Boltzmann" />
<me:energyTransferModel name="ExponentialDown" />
<me:deltaEDown >225.0</me:deltaEDown>
</molecule>
<molecule id="Butanal-14-rad" spinMultiplicity="2">
<atomArray>
<atom id="a1" elementType="C" x3="0.604381" y3="0.113777" z3="-0.078897" />
<atom id="a2" elementType="O" x3="0.847616" y3="1.293984" z3="0.082231" />
<atom id="a3" elementType="C" spinMultiplicity="2" x3="-0.676224" y3="-0.489746" z3="-
0.317447" />
<atom id="a4" elementType="O" x3="1.590168" y3="-0.816504" z3="-0.045280" />
<atom id="a5" elementType="H" x3="-0.711374" y3="-1.565372" z3="-0.430154" />
<atom id="a6" elementType="C" x3="-1.914498" y3="0.313001" z3="-0.414158" />
<atom id="a7" elementType="H" x3="-1.695141" y3="1.349544" z3="-0.161866" />
<atom id="a8" elementType="H" x3="-2.248374" y3="0.307348" z3="-1.458968" />
<atom id="a9" elementType="C" x3="-3.043008" y3="-0.237359" z3="0.462878" />
<atom id="a10" elementType="H" x3="-3.275265" y3="-1.270765" z3="0.203987" />
<atom id="a11" elementType="H" x3="-2.767269" y3="-0.206386" z3="1.516316" />
<atom id="a12" elementType="H" x3="-3.947293" y3="0.354864" z3="0.330931" />
<atom id="a13" elementType="O" x3="2.841756" y3="-0.214501" z3="0.191512" />
<atom id="a14" elementType="H" x3="2.584494" y3="0.728903" z3="0.257786" />
</atomArray>
<bondArray>
<bond atomRefs2="a8 a6" order="1" />
<bond atomRefs2="a5 a3" order="1" />
<bond atomRefs2="a6 a3" order="1" />
<bond atomRefs2="a6 a7" order="1" />
<bond atomRefs2="a6 a9" order="1" />
<bond atomRefs2="a3 a1" order="1" />

<bond atomRefs2="a1 a4" order="1" />
<bond atomRefs2="a1 a2" order="2" />
<bond atomRefs2="a4 a13" order="1" />
<bond atomRefs2="a13 a14" order="1" />
<bond atomRefs2="a10 a9" order="1" />
<bond atomRefs2="a12 a9" order="1" />
<bond atomRefs2="a9 a11" order="1" />
</bondArray>
<propertyList>
<property title="program">
<scalar>Gaussian 16, Revision C.01</scalar>
</property>
<property title="basis">
<scalar>Aug-CC-pVTZ (5D, 7F)</scalar>
</property>
<property title="File Format">
<scalar>g03</scalar>
</property>
<property title="Energy" dictRef="me:ZPE">
<scalar units="kcal/mol">-2.23</scalar>
</property>
<property title="SpinMultiplicity" dictRef="me:spinMultiplicity">
<scalar>2.00</scalar>
</property>
<property title="Vibrational Frequencies" dictRef="me:vibFreqs">
<array units="cm-1">24.46 90.12 172.42 200.53 255.35 349.19 380.97 460.99 501.03 609.59 707.72 764.72 807.61 925.29 970.79 1031.01 1058.09 1094.87 1169.18 1209.35 1279.93 1341.65 1418.29 1468.02 1484.26 1504.93 1511.11 1521.95 1749.60 3010.16 3054.16 3110.90 3132.37 3137.38 3204.15 3539.82</array>
</property>
<property title="Rotational Constants" dictRef="me:rotConsts">
<array units="cm-1">0.275 0.042 0.038</array>
</property>
<property title="Symmetry Number" dictRef="me:symmetryNumber">
<scalar>1</scalar>
</property>
</propertyList>
</molecule>
<molecule id="14Hshift-TS" spinMultiplicity="2">
<atomArray>
<atom id="a1" elementType="C" x3="1.016152" y3="0.546451" z3="0.090614" />
<atom id="a2" elementType="O" x3="1.382656" y3="1.674176" z3="0.012302" />
<atom id="a3" elementType="C" x3="-0.346013" y3="0.049582" z3="0.497816" />
<atom id="a4" elementType="O" x3="1.828092" y3="-0.499341" z3="-0.207259" />
<atom id="a5" elementType="O" spinMultiplicity="2" x3="1.111623" y3="-1.697599" z3="-0.035735" />

<atom id="a6" elementType="H" x3="-0.534665" y3="0.176973" z3="1.562771" />
<atom id="a7" elementType="H" x3="0.018092" y3="-1.158430" z3="0.335265" />
<atom id="a8" elementType="C" x3="-1.497772" y3="0.422292" z3="-0.395656" />
<atom id="a9" elementType="H" x3="-1.597566" y3="1.511851" z3="-0.366405" />
<atom id="a10" elementType="H" x3="-1.256445" y3="0.163892" z3="-1.427988" />
<atom id="a11" elementType="C" x3="-2.804302" y3="-0.235583" z3="0.029243" />
<atom id="a12" elementType="H" x3="-2.728817" y3="-1.322217" z3="-0.015768" />
<atom id="a13" elementType="H" x3="-3.069130" y3="0.042099" z3="1.050097" />
<atom id="a14" elementType="H" x3="-3.618821" y3="0.071494" z3="-0.624540" />
</atomArray>
<bondArray>
<bond atomRefs2="a10 a8" order="1" />
<bond atomRefs2="a14 a11" order="1" />
<bond atomRefs2="a8 a9" order="1" />
<bond atomRefs2="a8 a11" order="1" />
<bond atomRefs2="a8 a3" order="1" />
<bond atomRefs2="a4 a5" order="1" />
<bond atomRefs2="a4 a1" order="1" />
<bond atomRefs2="a12 a11" order="1" />
<bond atomRefs2="a2 a1" order="2" />
<bond atomRefs2="a11 a13" order="1" />
<bond atomRefs2="a1 a3" order="1" />
<bond atomRefs2="a7 a3" order="1" />
<bond atomRefs2="a3 a6" order="1" />
</bondArray>
<propertyList>
<property title="program">
<scalar>Gaussian 16, Revision C.01</scalar>
</property>
<property title="basis">
<scalar>Aug-CC-pVTZ (5D, 7F)</scalar>
</property>
<property title="File Format">
<scalar>g03</scalar>
</property>
<property title="Energy" dictRef="me:ZPE">
<scalar units="kcal/mol" >26.54</scalar>
</property>
<property title="SpinMultiplicity" dictRef="me:spinMultiplicity">
<scalar>2.00</scalar>
</property>
<property title="Vibrational Frequencies" dictRef="me:vibFreqs">
<array units="cm-1">68.67 82.36 163.26 210.84 248.97 324.18 522.36 575.61 671.33 691.23 745.08 864.76 891.31 952.52 1017.23 1068.93 1113.29 1146.37 1178.26 1188.67 1284.36 1300.45 1398.83 1427.05 1480.90 1506.28 1509.21 1763.78 1914.31 3038.94 3053.38 3086.87 3124.56 3128.02 3138.72</array>

```
      </property>
      <property title="ImaginaryFrequency" dictRef="me:imFreqs">
        <scalar units="cm-1">1623.87</scalar>
      </property>
      <property title="Rotational Constants" dictRef="me:rotConsts">
        <array units="cm-1">0.142 0.059 0.044</array>
      </property>
      <property title="Symmetry Number" dictRef="me:symmetryNumber">
        <scalar>1</scalar>
      </property>
      <property dictRef="me:frequenciesScaleFactor" >
        <scalar>1</scalar>
      </property>
    </propertyList>
    <me:DOSCMethod name="ClassicalRotors" />
  </molecule>
</moleculeList>
<reactionList>
  <reaction id="R1" >
        <!--reversible="true"-->
    <reactantList>
      <reactant>
        <molecule ref="Butanal-AcylOO-rad" role="modelled" />
      </reactant>
    </reactantList>
    <productList>
      <product>
        <molecule ref="Butanal-14-rad" role="sink" />
      </product>
    </productList>
    <me:transitionState>
      <molecule ref="14Hshift-TS" role="transitionState" />
    </me:transitionState>
    <me:MCRCMethod name="RRKM" />
          <me:tunneling name="Eckart" />
  </reaction>
  <reaction id="R2" >
        <!--reversible="true"-->
    <reactantList>
      <reactant>
        <molecule ref="Butanal-AcylOO-rad" role="modelled" />
      </reactant>
    </reactantList>
    <productList>
      <product>
        <molecule ref="Butanal-15-rad" role="sink" />
```

```
      </product>
    </productList>
    <me:transitionState>
      <molecule ref="15Hshift-TS" role="transitionState" />
    </me:transitionState>
    <me:MCRCMethod name="RRKM" />
          <me:tunneling name="Eckart" />
  </reaction>
  <reaction id="R3" >
        <!--reversible="true"-->
    <reactantList>
      <reactant>
        <molecule ref="Butanal-AcylOO-rad" role="modelled" />
      </reactant>
    </reactantList>
    <productList>
      <product>
        <molecule ref="Butanal-16-rad" role="sink" />
      </product>
    </productList>
    <me:transitionState>
      <molecule ref="16Hshift-TS" role="transitionState" />
    </me:transitionState>
    <me:MCRCMethod name="RRKM" />
          <me:tunneling name="Eckart" />
  </reaction>
</reactionList>
<me:conditions>
  <me:bathGas>N2</me:bathGas>
  <me:PTs>
    <me:PTpair units="Torr" P="760." T="298." precision="qd" bathGas="N2" />
  </me:PTs>
        <me:InitialPopulation>
    <me:molecule ref="Butanal-AcylOO-rad" population="1.0" />
  </me:InitialPopulation>
</me:conditions>
<me:modelParameters>
  <!--Specify grain size directly...-->
  <me:grainSize units="cm-1">100</me:grainSize>
  <!--...or by the total number of grains
      <me:numberOfGrains> 500 </me:numberOfGrains>-->
  <!--Specify increased energy range
      <me:maxTemperature>6000</me:maxTemperature>-->
  <me:energyAboveTheTopHill>25.0</me:energyAboveTheTopHill>
</me:modelParameters>
<me:control>
```

```
    <me:printSpeciesProfile />
    <!--<me:testMicroRates />
          <me:testDOS />
    <me:testRateConstant />
    <me:printGrainDOS />-->
    <!--<me:printCellDOS />-->
    <!--<me:printReactionOperatorColumnSums />-->
    <!--<me:printTunnellingCoefficients />
    <me:printGrainkfE />-->
    <!--<me:printGrainBoltzmann />
    <me:printGrainkbE />-->
    <me:eigenvalues>3</me:eigenvalues>
    <!-- <me:hideInactive/>  Molecules and reactions with attribute active="false" are not shown
    <me:diagramEnergyOffset>0</me:diagramEnergyOffset>-->
    <!--Adjusts displayed energies to this values for the lowest species. -->
    <me:calcMethod name="simpleCalc" />
    <me:ForceMacroDetailedBalance >true</me:ForceMacroDetailedBalance>
  </me:control>
  <metadataList xmlns:dc="http://purl.org/dc/elements/1.1/">
    <dc:title>Project name</dc:title>
    <dc:source>Acylperoxyrad-Butanal.xml</dc:source>
    <dc:creator>Mesmer v6.0</dc:creator>
    <dc:date>20220117_153338</dc:date>
    <dc:contributor>ndprse</dc:contributor>
  </metadataList>
</me:mesmer>
```


Pentanal–AcOO ------> Pentanal–AcOOH


```
<?xml version="1.0" encoding="utf-8" ?>
<?xml-stylesheet type='text/xsl' href='../../mesmer2.xsl' media='other'?>
<?xml-stylesheet type='text/xsl' href='../../mesmer1.xsl' media='screen'?>
<me:mesmer                                                xmlns="http://www.xml-cml.org/schema"
xmlns:me="http://www.chem.leeds.ac.uk/mesmer"
xmlns:xsi="http://www.w3.org/2001/XMLSchema-instance">
  <me:title>Project name</me:title>
  <moleculeList convention="">
    <molecule id="N2">
    <atomArray>
      <atom id="a1" elementType="N" />
      <atom id="a2" elementType="N" />
    </atomArray>
    <bondArray>
      <bond atomRefs2="a2 a1" order="3"/>
```

```
    </bondArray>
    <propertyList>
      <property dictRef="me:epsilon">
        <scalar>48.0</scalar>
      </property>
      <property dictRef="me:sigma">
        <scalar>3.90</scalar>
      </property>
      <property dictRef="me:MW">
        <scalar units="amu">28.0</scalar>
      </property>
    </propertyList>
  </molecule>
    <molecule id="Pentanal-15-rad" spinMultiplicity="2">
      <atomArray>
        <atom id="a1" elementType="C" x3="1.059070" y3="0.112695" z3="0.383158" />
        <atom id="a2" elementType="O" x3="1.107986" y3="-0.959505" z3="0.931413" />
        <atom id="a3" elementType="C" x3="-0.020386" y3="1.157880" z3="0.536148" />
        <atom id="a4" elementType="H" x3="-0.242017" y3="1.221830" z3="1.602956" />
        <atom id="a5" elementType="H" x3="0.359336" y3="2.117698" z3="0.190158" />
        <atom id="a6" elementType="C" spinMultiplicity="2" x3="-1.229612" y3="0.739460" z3="-
0.234037" />
        <atom id="a7" elementType="C" x3="-2.189812" y3="-0.243715" z3="0.325979" />
        <atom id="a8" elementType="H" x3="-1.662951" y3="-1.184004" z3="0.539873" />
        <atom id="a9" elementType="H" x3="-2.535629" y3="0.107842" z3="1.305884" />
        <atom id="a10" elementType="C" x3="-3.381553" y3="-0.516152" z3="-0.582879" />
        <atom id="a11" elementType="H" x3="-3.945704" y3="0.397503" z3="-0.772772" />
        <atom id="a12" elementType="H" x3="-4.057782" y3="-1.242899" z3="-0.134703" />
        <atom id="a13" elementType="O" x3="1.982910" y3="0.495596" z3="-0.511043" />
        <atom id="a14" elementType="H" x3="-1.281743" y3="1.007410" z3="-1.281524" />
        <atom id="a15" elementType="H" x3="-3.053277" y3="-0.912906" z3="-1.544298" />
        <atom id="a16" elementType="O" x3="2.953324" y3="-0.512498" z3="-0.717598" />
        <atom id="a17" elementType="H" x3="2.639773" y3="-1.202231" z3="-0.097966" />
      </atomArray>
      <bondArray>
        <bond atomRefs2="a15 a10" order="1" />
        <bond atomRefs2="a14 a6" order="1" />
        <bond atomRefs2="a11 a10" order="1" />
        <bond atomRefs2="a16 a13" order="1" />
        <bond atomRefs2="a16 a17" order="1" />
        <bond atomRefs2="a10 a12" order="1" />
        <bond atomRefs2="a10 a7" order="1" />
        <bond atomRefs2="a13 a1" order="1" />
        <bond atomRefs2="a6 a7" order="1" />
        <bond atomRefs2="a6 a3" order="1" />
        <bond atomRefs2="a5 a3" order="1" />
```

```
<bond atomRefs2="a7 a8" order="1" />
<bond atomRefs2="a7 a9" order="1" />
<bond atomRefs2="a1 a3" order="1" />
<bond atomRefs2="a1 a2" order="2" />
<bond atomRefs2="a3 a4" order="1" />
</bondArray>
<propertyList>
<property title="program">
<scalar>Gaussian 16, Revision C.01</scalar>
</property>
<property title="basis">
<scalar>Aug-CC-pVTZ (5D, 7F)</scalar>
</property>
<property title="File Format">
<scalar>g03</scalar>
</property>
<property title="Energy" dictRef="me:ZPE">
<scalar units="kcal/mol" >2.68</scalar>
</property>
<property title="SpinMultiplicity" dictRef="me:spinMultiplicity">
<scalar>2.00</scalar>
</property>
<property title="Vibrational Frequencies" dictRef="me:vibFreqs">
<array units="cm-1">33.03 47.83 74.40 160.12 238.35 246.16 261.37 321.96 407.61 431.88
463.40 498.94 653.91 769.00 801.29 848.77 930.41 978.37 1026.98 1042.02 1069.11 1102.55 1148.81
1207.86 1271.88 1285.60 1320.40 1348.15 1422.52 1430.28 1470.68 1475.25 1505.86 1509.90 1525.96
1813.58 2987.17 3012.57 3050.65 3066.10 3120.65 3130.58 3132.70 3190.34 3565.16</array>
</property>
<property title="Rotational Constants" dictRef="me:rotConsts">
<array units="cm-1">0.155 0.030 0.030</array>
</property>
<property title="Symmetry Number" dictRef="me:symmetryNumber">
<scalar>1</scalar>
</property>
</propertyList>
</molecule>
<molecule id="15Hshift-TS" spinMultiplicity="2">
<atomArray>
<atom id="a1" elementType="C" x3="-1.496941" y3="-0.299314" z3="-0.128694" />
<atom id="a2" elementType="O" x3="-2.322489" y3="-0.929553" z3="-0.710282" />
<atom id="a3" elementType="C" x3="-0.572494" y3="-0.813085" z3="0.958231" />
<atom id="a4" elementType="H" x3="-1.007834" y3="-0.536743" z3="1.919271" />
<atom id="a5" elementType="H" x3="-0.585588" y3="-1.899799" z3="0.876549" />
<atom id="a6" elementType="C" spinMultiplicity="2" x3="0.814443" y3="-0.236157"
z3="0.826538" />
<atom id="a7" elementType="C" x3="1.644941" y3="-0.681881" z3="-0.345219" />
```

```
    <atom id="a8" elementType="H" x3="1.876304" y3="-1.745372" z3="-0.210584" />
    <atom id="a9" elementType="H" x3="1.050664" y3="-0.619570" z3="-1.260516" />
    <atom id="a10" elementType="C" x3="2.933427" y3="0.113964" z3="-0.503117" />
    <atom id="a11" elementType="H" x3="3.550558" y3="0.043170" z3="0.393669" />
    <atom id="a12" elementType="H" x3="2.717156" y3="1.167785" z3="-0.680274" />
    <atom id="a13" elementType="O" x3="-1.285671" y3="0.999543" z3="-0.513901" />
    <atom id="a14" elementType="O" x3="-0.499851" y3="1.745044" z3="0.360883" />
    <atom id="a15" elementType="H" x3="1.358738" y3="-0.151530" z3="1.765310" />
    <atom id="a16" elementType="H" x3="0.444342" y3="0.975469" z3="0.599916" />
    <atom id="a17" elementType="H" x3="3.519493" y3="-0.254841" z3="-1.343374" />
  </atomArray>
  <bondArray>
    <bond atomRefs2="a17 a10" order="1" />
    <bond atomRefs2="a9 a7" order="1" />
    <bond atomRefs2="a2 a1" order="2" />
    <bond atomRefs2="a12 a10" order="1" />
    <bond atomRefs2="a13 a1" order="1" />
    <bond atomRefs2="a13 a14" order="1" />
    <bond atomRefs2="a10 a7" order="1" />
    <bond atomRefs2="a10 a11" order="1" />
    <bond atomRefs2="a7 a8" order="1" />
    <bond atomRefs2="a7 a6" order="1" />
    <bond atomRefs2="a1 a3" order="1" />
    <bond atomRefs2="a14 a16" order="1" />
    <bond atomRefs2="a6 a3" order="1" />
    <bond atomRefs2="a6 a15" order="1" />
    <bond atomRefs2="a5 a3" order="1" />
    <bond atomRefs2="a3 a4" order="1" />
  </bondArray>
  <propertyList>
    <property title="program">
      <scalar>Gaussian 16, Revision C.01</scalar>
    </property>
    <property title="basis">
      <scalar>Aug-CC-pVTZ (5D, 7F)</scalar>
    </property>
    <property title="File Format">
      <scalar>g03</scalar>
    </property>
    <property title="Energy" dictRef="me:ZPE">
      <scalar units="kcal/mol" >20.64</scalar>
    </property>
    <property title="SpinMultiplicity" dictRef="me:spinMultiplicity">
      <scalar>2.00</scalar>
    </property>
    <property title="Vibrational Frequencies" dictRef="me:vibFreqs">
```

<array units="cm-1">70.23 83.04 119.55 213.81 238.90 284.02 380.94 479.28 505.23 552.45 590.36 701.91 755.32 790.28 857.15 918.13 971.47 1035.16 1054.80 1076.28 1110.25 1155.22 1191.20 1221.72 1246.45 1289.94 1315.05 1336.86 1418.03 1426.75 1457.78 1477.91 1506.60 1509.35 1577.06 1873.91 3013.31 3050.84 3066.59 3076.36 3119.57 3122.99 3130.97 3134.84</array>
</property>
<property title="ImaginaryFrequency" dictRef="me:imFreqs">
<scalar units="cm-1">1699.90</scalar>
</property>
<property title="Rotational Constants" dictRef="me:rotConsts">
<array units="cm-1">0.110 0.044 0.039</array>
</property>
<property title="Symmetry Number" dictRef="me:symmetryNumber">
<scalar>1</scalar>
</property>
<property dictRef="me:frequenciesScaleFactor" >
<scalar>1</scalar>
</property>
</propertyList>
<me:DOSCMethod name="ClassicalRotors" />
</molecule>
<molecule id="Pentanal-16-rad" spinMultiplicity="2">
<atomArray>
<atom id="a1" elementType="C" x3="-0.984876" y3="0.369388" z3="0.203177" />
<atom id="a2" elementType="O" x3="-1.114036" y3="0.036715" z3="1.354101" />
<atom id="a3" elementType="C" x3="0.028410" y3="1.332064" z3="-0.344394" />
<atom id="a4" elementType="H" x3="-0.050499" y3="1.365055" z3="-1.430011" />
<atom id="a5" elementType="H" x3="-0.234648" y3="2.318024" z3="0.041596" />
<atom id="a6" elementType="C" x3="1.442668" y3="0.950124" z3="0.094251" />
<atom id="a7" elementType="H" x3="1.484406" y3="0.921754" z3="1.186843" />
<atom id="a8" elementType="H" x3="2.120312" y3="1.761100" z3="-0.207723" />
<atom id="a9" elementType="C" spinMultiplicity="2" x3="1.898414" y3="-0.348860" z3="-0.466437" />
<atom id="a10" elementType="C" x3="3.074164" y3="-1.050683" z3="0.101923" />
<atom id="a11" elementType="H" x3="2.990317" y3="-1.146760" z3="1.188503" />
<atom id="a12" elementType="H" x3="4.006137" y3="-0.500143" z3="-0.086210" />
<atom id="a13" elementType="O" x3="-1.786193" y3="-0.105375" z3="-0.763155" />
<atom id="a14" elementType="O" x3="-2.753455" y3="-0.994370" z3="-0.241613" />
<atom id="a15" elementType="H" x3="1.476909" y3="-0.685408" z3="-1.405762" />
<atom id="a16" elementType="H" x3="3.195281" y3="-2.046764" z3="-0.320159" />
<atom id="a17" elementType="H" x3="-2.511425" y3="-0.994822" z3="0.707143" />
</atomArray>
<bondArray>
<bond atomRefs2="a4 a3" order="1" />
<bond atomRefs2="a15 a9" order="1" />
<bond atomRefs2="a13 a14" order="1" />
<bond atomRefs2="a13 a1" order="1" />

<bond atomRefs2="a9 a6" order="1" />
<bond atomRefs2="a9 a10" order="1" />
<bond atomRefs2="a3 a5" order="1" />
<bond atomRefs2="a3 a6" order="1" />
<bond atomRefs2="a3 a1" order="1" />
<bond atomRefs2="a16 a10" order="1" />
<bond atomRefs2="a14 a17" order="1" />
<bond atomRefs2="a8 a6" order="1" />
<bond atomRefs2="a12 a10" order="1" />
<bond atomRefs2="a6 a7" order="1" />
<bond atomRefs2="a10 a11" order="1" />
<bond atomRefs2="a1 a2" order="2" />
</bondArray>
<propertyList>
<property title="program">
<scalar>Gaussian 16, Revision C.01</scalar>
</property>
<property title="basis">
<scalar>Aug-CC-pVTZ (5D, 7F)</scalar>
</property>
<property title="File Format">
<scalar>g03</scalar>
</property>
<property title="Energy" dictRef="me:ZPE">
<scalar units="kcal/mol" >1.67</scalar>
</property>
<property title="SpinMultiplicity" dictRef="me:spinMultiplicity">
<scalar>2.00</scalar>
</property>
<property title="Vibrational Frequencies" dictRef="me:vibFreqs">
<array units="cm-1">26.19 41.88 83.08 116.08 194.26 219.94 296.95 339.20 384.37 429.27 469.65 504.66 631.20 718.69 855.77 897.95 949.58 980.52 1000.23 1021.16 1057.93 1120.75 1150.32 1195.85 1231.80 1284.22 1331.70 1397.57 1411.06 1438.05 1471.34 1480.21 1482.95 1489.55 1530.93 1823.17 2977.15 2983.51 3049.67 3056.43 3079.66 3127.64 3133.55 3180.74 3556.69</array>
</property>
<property title="Rotational Constants" dictRef="me:rotConsts">
<array units="cm-1">0.125 0.035 0.033</array>
</property>
<property title="Symmetry Number" dictRef="me:symmetryNumber">
<scalar>1</scalar>
</property>
</propertyList>
</molecule>
<molecule id="16Hshift-TS" spinMultiplicity="2">
<atomArray>
<atom id="a1" elementType="C" x3="-1.495809" y3="-0.099302" z3="-0.128717" />

<atom id="a2" elementType="O" x3="-2.437841" y3="-0.402227" z3="-0.794551" />
<atom id="a3" elementType="C" x3="-0.687425" y3="-1.040211" z3="0.727407" />
<atom id="a4" elementType="H" x3="-0.597769" y3="-0.653997" z3="1.741728" />
<atom id="a5" elementType="H" x3="-1.243623" y3="-1.972882" z3="0.751369" />
<atom id="a6" elementType="C" x3="0.710068" y3="-1.266175" z3="0.114912" />
<atom id="a7" elementType="H" x3="0.602024" y3="-1.456452" z3="-0.954591" />
<atom id="a8" elementType="H" x3="1.128878" y3="-2.175841" z3="0.552279" />
<atom id="a9" elementType="C" spinMultiplicity="2" x3="1.672311" y3="-0.120177" z3="0.341143" />
<atom id="a10" elementType="C" x3="2.657565" y3="0.182307" z3="-0.750662" />
<atom id="a11" elementType="H" x3="3.328625" y3="-0.670012" z3="-0.899535" />
<atom id="a12" elementType="H" x3="3.269907" y3="1.049835" z3="-0.511640" />
<atom id="a13" elementType="O" x3="-1.039819" y3="1.191711" z3="-0.251056" />
<atom id="a14" elementType="O" x3="-0.117849" y3="1.559959" z3="0.718544" />
<atom id="a15" elementType="H" x3="2.099488" y3="-0.115997" z3="1.344351" />
<atom id="a16" elementType="H" x3="0.887946" y3="0.895484" z3="0.465393" />
<atom id="a17" elementType="H" x3="2.148346" y3="0.365660" z3="-1.697356" />
</atomArray>
<bondArray>
<bond atomRefs2="a17 a10" order="1" />
<bond atomRefs2="a7 a6" order="1" />
<bond atomRefs2="a11 a10" order="1" />
<bond atomRefs2="a2 a1" order="2" />
<bond atomRefs2="a10 a12" order="1" />
<bond atomRefs2="a10 a9" order="1" />
<bond atomRefs2="a13 a1" order="1" />
<bond atomRefs2="a13 a14" order="1" />
<bond atomRefs2="a1 a3" order="1" />
<bond atomRefs2="a6 a9" order="1" />
<bond atomRefs2="a6 a8" order="1" />
<bond atomRefs2="a6 a3" order="1" />
<bond atomRefs2="a9 a15" order="1" />
<bond atomRefs2="a16 a14" order="1" />
<bond atomRefs2="a3 a5" order="1" />
<bond atomRefs2="a3 a4" order="1" />
</bondArray>
<propertyList>
<property title="program">
<scalar>Gaussian 16, Revision C.01</scalar>
</property>
<property title="basis">
<scalar>Aug-CC-pVTZ (5D, 7F)</scalar>
</property>
<property title="File Format">
<scalar>g03</scalar>
</property>

<property title="Energy" dictRef="me:ZPE">
<scalar units="kcal/mol" >19.51</scalar>
</property>
<property title="SpinMultiplicity" dictRef="me:spinMultiplicity">
<scalar>2.00</scalar>
</property>
<property title="Vibrational Frequencies" dictRef="me:vibFreqs">
<array units="cm-1">47.78 123.39 185.05 207.61 260.28 330.84 389.82 397.90 512.38 528.29 581.85 735.52 768.57 844.50 871.14 907.98 972.82 1017.39 1074.87 1105.17 1113.39 1134.64 1178.22 1218.14 1244.19 1298.72 1335.24 1365.91 1397.70 1417.15 1444.74 1477.51 1487.04 1495.39 1497.45 1872.18 3025.91 3045.88 3083.29 3095.08 3100.43 3105.67 3140.74 3161.88</array>
</property>
<property title="ImaginaryFrequency" dictRef="me:imFreqs">
<scalar units="cm-1">1778.60</scalar>
</property>
<property title="Rotational Constants" dictRef="me:rotConsts">
<array units="cm-1">0.108 0.048 0.041</array>
</property>
<property title="Symmetry Number" dictRef="me:symmetryNumber">
<scalar>1</scalar>
</property>
<property dictRef="me:frequenciesScaleFactor" >
<scalar>1</scalar>
</property>
</propertyList>
<me:DOSCMethod name="ClassicalRotors" />
</molecule>
<molecule id="Pentanal-17-rad" spinMultiplicity="2">
<atomArray>
<atom id="a1" elementType="C" x3="1.556315" y3="-0.989503" z3="0.196225" />
<atom id="a2" elementType="H" x3="2.350273" y3="-1.258388" z3="0.895471" />
<atom id="a3" elementType="H" x3="1.320832" y3="-1.882058" z3="-0.382773" />
<atom id="a4" elementType="C" x3="2.055687" y3="0.113940" z3="-0.738634" />
<atom id="a5" elementType="H" x3="1.273366" y3="0.374939" z3="-1.456829" />
<atom id="a6" elementType="C" spinMultiplicity="2" x3="2.530153" y3="1.329315" z3="-0.031632" />
<atom id="a7" elementType="H" x3="3.021525" y3="1.246525" z3="0.929248" />
<atom id="a8" elementType="C" x3="0.327765" y3="-0.581218" z3="1.005902" />
<atom id="a9" elementType="H" x3="0.064134" y3="-1.368847" z3="1.715097" />
<atom id="a10" elementType="H" x3="0.511249" y3="0.326416" z3="1.579760" />
<atom id="a11" elementType="C" x3="-0.879032" y3="-0.378660" z3="0.136097" />
<atom id="a12" elementType="O" x3="-1.204857" y3="-1.041887" z3="-0.816831" />
<atom id="a13" elementType="O" x3="-1.616901" y3="0.660345" z3="0.555333" />
<atom id="a14" elementType="H" x3="2.870462" y3="-0.303720" z3="-1.347055" />
<atom id="a15" elementType="O" x3="-2.780046" y3="0.820429" z3="-0.231834" />
<atom id="a16" elementType="H" x3="-2.666401" y3="0.095435" z3="-0.879990" />

<atom id="a17" elementType="H" x3="2.523664" y3="2.295359" z3="-0.514018" />
</atomArray>
<bondArray>
<bond atomRefs2="a5 a4" order="1" />
<bond atomRefs2="a14 a4" order="1" />
<bond atomRefs2="a16 a15" order="1" />
<bond atomRefs2="a12 a11" order="2" />
<bond atomRefs2="a4 a6" order="1" />
<bond atomRefs2="a4 a1" order="1" />
<bond atomRefs2="a17 a6" order="1" />
<bond atomRefs2="a3 a1" order="1" />
<bond atomRefs2="a15 a13" order="1" />
<bond atomRefs2="a6 a7" order="1" />
<bond atomRefs2="a11 a13" order="1" />
<bond atomRefs2="a11 a8" order="1" />
<bond atomRefs2="a1 a2" order="1" />
<bond atomRefs2="a1 a8" order="1" />
<bond atomRefs2="a8 a10" order="1" />
<bond atomRefs2="a8 a9" order="1" />
</bondArray>
<propertyList>
<property title="program">
<scalar>Gaussian 16, Revision C.01</scalar>
</property>
<property title="basis">
<scalar>Aug-CC-pVTZ (5D, 7F)</scalar>
</property>
<property title="File Format">
<scalar>g03</scalar>
</property>
<property title="Energy" dictRef="me:ZPE">
<scalar units="kcal/mol" >4.31</scalar>
</property>
<property title="SpinMultiplicity" dictRef="me:spinMultiplicity">
<scalar>2.00</scalar>
</property>
<property title="Vibrational Frequencies" dictRef="me:vibFreqs">
<array units="cm-1">20.87 54.06 129.33 144.00 199.25 225.86 309.05 379.64 426.33 464.44 490.91 502.26 625.35 705.95 807.00 854.77 901.52 952.77 990.60 1003.02 1051.45 1105.78 1130.01 1181.47 1212.10 1273.81 1296.75 1372.34 1397.19 1410.52 1470.38 1471.32 1475.87 1496.20 1516.37 1817.50 2973.47 3053.69 3066.13 3073.35 3113.75 3128.69 3152.78 3259.78 3556.86</array>
</property>
<property title="Rotational Constants" dictRef="me:rotConsts">
<array units="cm-1">0.124 0.038 0.035</array>
</property>
<property title="Symmetry Number" dictRef="me:symmetryNumber">

<scalar>1</scalar>
</property>
</propertyList>
</molecule>
<molecule id="17Hshift-TS" spinMultiplicity="2">
<atomArray>
<atom id="a1" elementType="C" x3="0.771040" y3="-1.507056" z3="0.264176" />
<atom id="a2" elementType="H" x3="1.455106" y3="-1.876485" z3="1.030030" />
<atom id="a3" elementType="H" x3="0.400548" y3="-2.376839" z3="-0.278273" />
<atom id="a4" elementType="C" x3="1.531453" y3="-0.614505" z3="-0.718671" />
<atom id="a5" elementType="H" x3="0.897717" y3="-0.380871" z3="-1.578445" />
<atom id="a6" elementType="C" spinMultiplicity="2" x3="2.063385" y3="0.660214" z3="-0.132447" />
<atom id="a7" elementType="H" x3="2.578673" y3="1.320733" z3="-0.823772" />
<atom id="a8" elementType="C" x3="-0.416464" y3="-0.831379" z3="0.979094" />
<atom id="a9" elementType="H" x3="-1.031920" y3="-1.593052" z3="1.451172" />
<atom id="a10" elementType="H" x3="-0.068651" y3="-0.139535" z3="1.739937" />
<atom id="a11" elementType="C" x3="-1.295020" y3="-0.126275" z3="-0.018053" />
<atom id="a12" elementType="O" x3="-2.183144" y3="-0.626700" z3="-0.637228" />
<atom id="a13" elementType="O" x3="-0.996552" y3="1.183332" z3="-0.333646" />
<atom id="a14" elementType="H" x3="2.371880" y3="-1.187094" z3="-1.126771" />
<atom id="a15" elementType="O" x3="-0.080959" y3="1.794530" z3="0.511437" />
<atom id="a16" elementType="H" x3="0.989632" y3="1.366711" z3="0.188205" />
<atom id="a17" elementType="H" x3="2.565884" y3="0.571135" z3="0.828829" />
</atomArray>
<bondArray>
<bond atomRefs2="a5 a4" order="1" />
<bond atomRefs2="a14 a4" order="1" />
<bond atomRefs2="a7 a6" order="1" />
<bond atomRefs2="a4 a6" order="1" />
<bond atomRefs2="a4 a1" order="1" />
<bond atomRefs2="a12 a11" order="2" />
<bond atomRefs2="a13 a11" order="1" />
<bond atomRefs2="a13 a15" order="1" />
<bond atomRefs2="a3 a1" order="1" />
<bond atomRefs2="a6 a17" order="1" />
<bond atomRefs2="a11 a8" order="1" />
<bond atomRefs2="a16 a15" order="1" />
<bond atomRefs2="a1 a8" order="1" />
<bond atomRefs2="a1 a2" order="1" />
<bond atomRefs2="a8 a9" order="1" />
<bond atomRefs2="a8 a10" order="1" />
</bondArray>
<propertyList>
<property title="program">
<scalar>Gaussian 16, Revision C.01</scalar>

</property>
<property title="basis">
<scalar>Aug-CC-pVTZ (5D, 7F)</scalar>
</property>
<property title="File Format">
<scalar>g03</scalar>
</property>
<property title="Energy" dictRef="me:ZPE">
<scalar units="kcal/mol" >20.72</scalar>
</property>
<property title="SpinMultiplicity" dictRef="me:spinMultiplicity">
<scalar>2.00</scalar>
</property>
<property title="Vibrational Frequencies" dictRef="me:vibFreqs">
<array units="cm-1">89.88 151.68 199.17 281.15 307.68 363.74 460.51 480.33 504.51 581.80
589.02 730.13 772.75 810.75 831.35 918.14 967.17 984.75 1068.00 1081.39 1100.13 1136.22 1150.70
1190.06 1240.35 1264.24 1305.74 1366.38 1383.92 1404.12 1448.80 1472.06 1482.28 1489.03 1502.99
1872.09 3021.63 3058.93 3070.08 3095.05 3109.41 3115.58 3169.18 3184.72</array>
</property>
<property title="ImaginaryFrequency" dictRef="me:imFreqs">
<scalar units="cm-1">1889.29</scalar>
</property>
<property title="Rotational Constants" dictRef="me:rotConsts">
<array units="cm-1">0.091 0.063 0.046</array>
</property>
<property title="Symmetry Number" dictRef="me:symmetryNumber">
<scalar>1</scalar>
</property>
<property dictRef="me:frequenciesScaleFactor" >
<scalar>1</scalar>
</property>
</propertyList>
<me:DOSCMethod name="ClassicalRotors" />
</molecule>
<molecule id="Pentanal-AcylOO-rad" spinMultiplicity="2">
<atomArray>
<atom id="a1" elementType="C" x3="-3.366360" y3="-1.502871" z3="0.000000" />
<atom id="a2" elementType="H" x3="-4.404385" y3="-1.171800" z3="0.000000" />
<atom id="a3" elementType="H" x3="-3.214448" y3="-2.128789" z3="0.880707" />
<atom id="a4" elementType="H" x3="-3.214448" y3="-2.128789" z3="-0.880707" />
<atom id="a5" elementType="C" x3="-2.407713" y3="-0.320001" z3="0.000000" />
<atom id="a6" elementType="H" x3="-2.599186" y3="0.307646" z3="-0.874578" />
<atom id="a7" elementType="H" x3="-2.599186" y3="0.307646" z3="0.874578" />
<atom id="a8" elementType="C" x3="-0.947000" y3="-0.754115" z3="0.000000" />
<atom id="a9" elementType="H" x3="-0.746266" y3="-1.378855" z3="-0.872491" />
<atom id="a10" elementType="H" x3="-0.746266" y3="-1.378855" z3="0.872491" />

<atom id="a11" elementType="C" x3="0.000000" y3="0.436707" z3="0.000000" />
<atom id="a12" elementType="H" x3="-0.164439" y3="1.080664" z3="0.866330" />
<atom id="a13" elementType="H" x3="-0.164439" y3="1.080664" z3="-0.866330" />
<atom id="a14" elementType="C" x3="1.434237" y3="0.016059" z3="0.000000" />
<atom id="a15" elementType="O" x3="1.890196" y3="-1.075025" z3="0.000000" />
<atom id="a16" elementType="O" x3="2.406140" y3="1.070605" z3="0.000000" />
<atom id="a17" elementType="O" spinMultiplicity="2" x3="1.900425" y3="2.273894" z3="0.000000" />
</atomArray>
<bondArray>
<bond atomRefs2="a4 a1" order="1" />
<bond atomRefs2="a6 a5" order="1" />
<bond atomRefs2="a9 a8" order="1" />
<bond atomRefs2="a13 a11" order="1" />
<bond atomRefs2="a1 a2" order="1" />
<bond atomRefs2="a1 a5" order="1" />
<bond atomRefs2="a1 a3" order="1" />
<bond atomRefs2="a5 a8" order="1" />
<bond atomRefs2="a5 a7" order="1" />
<bond atomRefs2="a8 a11" order="1" />
<bond atomRefs2="a8 a10" order="1" />
<bond atomRefs2="a11 a14" order="1" />
<bond atomRefs2="a11 a12" order="1" />
<bond atomRefs2="a14 a15" order="2" />
<bond atomRefs2="a14 a16" order="1" />
<bond atomRefs2="a16 a17" order="1" />
</bondArray>
<propertyList>
<property title="program">
<scalar>Gaussian 16, Revision C.01</scalar>
</property>
<property title="basis">
<scalar>Aug-CC-pVTZ (5D, 7F)</scalar>
</property>
<property title="File Format">
<scalar>g03</scalar>
</property>
<property title="Energy" dictRef="me:ZPE">
<scalar units="kcal/mol" >0.0</scalar>
</property>
<property title="SpinMultiplicity" dictRef="me:spinMultiplicity">
<scalar>2.00</scalar>
</property>
<property title="Vibrational Frequencies" dictRef="me:vibFreqs">
<array units="cm-1">53.55 86.94 115.11 132.54 145.49 246.44 253.57 309.88 429.15 529.33 560.28 606.17 738.68 808.87 820.34 928.02 946.26 1041.25 1062.72 1088.34 1135.63 1146.75 1242.83

```
1250.30 1301.30 1321.05 1344.37 1382.75 1424.57 1426.61 1450.95 1496.41 1502.71 1506.13 1516.02
1921.44 3034.54 3045.83 3057.30 3064.55 3071.17 3093.72 3111.96 3117.58 3126.25</array>
        </property>
        <property title="Rotational Constants" dictRef="me:rotConsts">
          <array units="cm-1">0.162 0.029 0.025</array>
        </property>
        <property title="Symmetry Number" dictRef="me:symmetryNumber">
          <scalar>1</scalar>
        </property>
        <property dictRef="me:sigma" >
          <scalar>6.65</scalar>
        </property>
        <property dictRef="me:epsilon" >
          <scalar>351.0</scalar>
        </property>
        <property dictRef="me:frequenciesScaleFactor" >
          <scalar>1</scalar>
        </property>
      </propertyList>
      <me:DOSCMethod  name="ClassicalRotors" />
      <me:DistributionCalcMethod  name="Boltzmann" />
      <me:energyTransferModel name="ExponentialDown"  />
      <me:deltaEDown >225.0</me:deltaEDown>
    </molecule>
          <molecule id="Pentanal-14-rad" spinMultiplicity="2">
      <atomArray>
        <atom id="a1" elementType="C" x3="0.924169" y3="-0.195883" z3="0.164158" />
        <atom id="a2" elementType="O" x3="1.049269" y3="0.438813" z3="1.193579" />
        <atom  id="a3"  elementType="C"  spinMultiplicity="2"  x3="-0.235420"  y3="-0.914005"  z3="-
0.283376" />
        <atom id="a4" elementType="O" x3="1.940609" y3="-0.292553" z3="-0.728069" />
        <atom id="a5" elementType="O" x3="3.072145" y3="0.417716" z3="-0.280613" />
        <atom id="a6" elementType="H" x3="-0.179490" y3="-1.406213" z3="-1.245305" />
        <atom id="a7" elementType="H" x3="2.744922" y3="0.768872" z3="0.573830" />
        <atom id="a8" elementType="C" x3="-1.490167" y3="-0.938780" z3="0.498272" />
        <atom id="a9" elementType="H" x3="-1.797996" y3="-1.979878" z3="0.634370" />
        <atom id="a10" elementType="H" x3="-1.312800" y3="-0.510953" z3="1.484964" />
        <atom id="a11" elementType="C" x3="-2.635104" y3="-0.179507" z3="-0.198848" />
        <atom id="a12" elementType="H" x3="-2.817778" y3="-0.623945" z3="-1.179882" />
        <atom id="a13" elementType="H" x3="-3.543351" y3="-0.331511" z3="0.386212" />
        <atom id="a14" elementType="C" x3="-2.354683" y3="1.309961" z3="-0.344923" />
        <atom id="a15" elementType="H" x3="-1.480352" y3="1.489976" z3="-0.972357" />
        <atom id="a16" elementType="H" x3="-3.200983" y3="1.824245" z3="-0.799021" />
        <atom id="a17" elementType="H" x3="-2.161133" y3="1.766890" z3="0.626315" />
      </atomArray>
      <bondArray>
```

<bond atomRefs2="a6 a3" order="1" />
<bond atomRefs2="a12 a11" order="1" />
<bond atomRefs2="a15 a14" order="1" />
<bond atomRefs2="a16 a14" order="1" />
<bond atomRefs2="a4 a5" order="1" />
<bond atomRefs2="a4 a1" order="1" />
<bond atomRefs2="a14 a11" order="1" />
<bond atomRefs2="a14 a17" order="1" />
<bond atomRefs2="a3 a1" order="1" />
<bond atomRefs2="a3 a8" order="1" />
<bond atomRefs2="a5 a7" order="1" />
<bond atomRefs2="a11 a13" order="1" />
<bond atomRefs2="a11 a8" order="1" />
<bond atomRefs2="a1 a2" order="2" />
<bond atomRefs2="a8 a9" order="1" />
<bond atomRefs2="a8 a10" order="1" />
</bondArray>
<propertyList>
<property title="program">
<scalar>Gaussian 16, Revision C.01</scalar>
</property>
<property title="basis">
<scalar>Aug-CC-pVTZ (5D, 7F)</scalar>
</property>
<property title="File Format">
<scalar>g03</scalar>
</property>
<property title="Energy" dictRef="me:ZPE">
<scalar units="kcal/mol">-2.27</scalar>
</property>
<property title="SpinMultiplicity" dictRef="me:spinMultiplicity">
<scalar>2.00</scalar>
</property>
<property title="Vibrational Frequencies" dictRef="me:vibFreqs">
<array units="cm-1">27.79 61.67 109.85 183.83 213.84 247.92 317.12 375.15 411.27 473.61 499.91 619.44 712.29 763.01 794.78 857.50 899.57 976.73 994.14 1059.29 1084.72 1103.69 1168.12 1204.07 1254.22 1289.11 1354.65 1379.27 1427.41 1461.50 1488.78 1499.29 1506.25 1509.97 1526.96 1749.92 3035.27 3047.47 3051.41 3088.98 3112.63 3120.80 3128.54 3204.73 3538.70</array>
</property>
<property title="Rotational Constants" dictRef="me:rotConsts">
<array units="cm-1">0.150 0.033 0.032</array>
</property>
<property title="Symmetry Number" dictRef="me:symmetryNumber">
<scalar>1</scalar>
</property>
</propertyList>

</molecule>
<molecule id="14Hshift-TS" spinMultiplicity="2">
<atomArray>
<atom id="a1" elementType="C" x3="1.538458" y3="0.561188" z3="0.058368" />
<atom id="a2" elementType="O" x3="1.864186" y3="1.699620" z3="-0.042328" />
<atom id="a3" elementType="C" x3="0.242545" y3="0.021243" z3="0.603174" />
<atom id="a4" elementType="O" x3="2.341105" y3="-0.457612" z3="-0.341309" />
<atom id="a5" elementType="O" spinMultiplicity="2" x3="1.680825" y3="-1.677691" z3="-
0.109140" />
<atom id="a6" elementType="H" x3="0.165438" y3="0.134854" z3="1.683451" />
<atom id="a7" elementType="H" x3="0.620242" y3="-1.173657" z3="0.388225" />
<atom id="a8" elementType="C" x3="-1.007301" y3="0.366019" z3="-0.158254" />
<atom id="a9" elementType="H" x3="-1.138201" y3="1.452787" z3="-0.110087" />
<atom id="a10" elementType="H" x3="-0.874201" y3="0.120276" z3="-1.214320" />
<atom id="a11" elementType="C" x3="-2.248789" y3="-0.328271" z3="0.392604" />
<atom id="a12" elementType="H" x3="-2.101701" y3="-1.410099" z3="0.355608" />
<atom id="a13" elementType="H" x3="-2.367164" y3="-0.067111" z3="1.447164" />
<atom id="a14" elementType="C" x3="-3.508101" y3="0.046196" z3="-0.376209" />
<atom id="a15" elementType="H" x3="-4.382527" y3="-0.459143" z3="0.031755" />
<atom id="a16" elementType="H" x3="-3.689991" y3="1.120920" z3="-0.330081" />
<atom id="a17" elementType="H" x3="-3.421692" y3="-0.231609" z3="-1.427601" />
</atomArray>
<bondArray>
<bond atomRefs2="a17 a14" order="1" />
<bond atomRefs2="a10 a8" order="1" />
<bond atomRefs2="a14 a16" order="1" />
<bond atomRefs2="a14 a15" order="1" />
<bond atomRefs2="a14 a11" order="1" />
<bond atomRefs2="a4 a5" order="1" />
<bond atomRefs2="a4 a1" order="1" />
<bond atomRefs2="a8 a9" order="1" />
<bond atomRefs2="a8 a11" order="1" />
<bond atomRefs2="a8 a3" order="1" />
<bond atomRefs2="a2 a1" order="2" />
<bond atomRefs2="a1 a3" order="1" />
<bond atomRefs2="a12 a11" order="1" />
<bond atomRefs2="a7 a3" order="1" />
<bond atomRefs2="a11 a13" order="1" />
<bond atomRefs2="a3 a6" order="1" />
</bondArray>
<propertyList>
<property title="program">
<scalar>Gaussian 16, Revision C.01</scalar>
</property>
<property title="basis">
<scalar>Aug-CC-pVTZ (5D, 7F)</scalar>

</property>
<property title="File Format">
<scalar>g03</scalar>
</property>
<property title="Energy" dictRef="me:ZPE">
<scalar units="kcal/mol" >26.46</scalar>
</property>
<property title="SpinMultiplicity" dictRef="me:spinMultiplicity">
<scalar>2.00</scalar>
</property>
<property title="Vibrational Frequencies" dictRef="me:vibFreqs">
<array units="cm-1">50.45 68.40 123.16 126.13 205.43 243.88 283.81 371.60 520.21 592.19
675.86 690.98 732.98 806.61 883.16 921.62 941.50 1017.35 1050.38 1082.67 1129.50 1151.43 1175.84
1189.17 1263.74 1276.19 1335.25 1364.77 1414.03 1426.08 1477.46 1499.68 1506.55 1513.07 1765.74
1914.69 3026.74 3044.65 3048.10 3069.92 3089.27 3119.46 3126.69 3130.33</array>
</property>
<property title="ImaginaryFrequency" dictRef="me:imFreqs">
<scalar units="cm-1">1628.40</scalar>
</property>
<property title="Rotational Constants" dictRef="me:rotConsts">
<array units="cm-1">0.132 0.033 0.028</array>
</property>
<property title="Symmetry Number" dictRef="me:symmetryNumber">
<scalar>1</scalar>
</property>
<property dictRef="me:frequenciesScaleFactor">
<scalar>1</scalar>
</property>
</propertyList>
<me:DOSCMethod name="ClassicalRotors" />
</molecule>
</moleculeList>
<reactionList>
<reaction id="R1" >
<!--reversible="true"-->
<reactantList>
<reactant>
<molecule ref="Pentanal-AcylOO-rad" role="modelled" />
</reactant>
</reactantList>
<productList>
<product>
<molecule ref="Pentanal-14-rad" role="sink" />
</product>
</productList>
<me:transitionState>

```
    <molecule ref="14Hshift-TS" role="transitionState" />
  </me:transitionState>
  <me:MCRCMethod name="RRKM" />
        <me:tunneling name="Eckart" />
</reaction>
<reaction id="R2" >
      <!--reversible="true"-->
  <reactantList>
    <reactant>
      <molecule ref="Pentanal-AcylOO-rad" role="modelled" />
    </reactant>
  </reactantList>
  <productList>
    <product>
      <molecule ref="Pentanal-15-rad" role="sink" />
    </product>
  </productList>
  <me:transitionState>
    <molecule ref="15Hshift-TS" role="transitionState" />
  </me:transitionState>
  <me:MCRCMethod name="RRKM" />
        <me:tunneling name="Eckart" />
</reaction>
<reaction id="R3" >
      <!--reversible="true"-->
  <reactantList>
    <reactant>
      <molecule ref="Pentanal-AcylOO-rad" role="modelled" />
    </reactant>
  </reactantList>
  <productList>
    <product>
      <molecule ref="Pentanal-16-rad" role="sink" />
    </product>
  </productList>
  <me:transitionState>
    <molecule ref="16Hshift-TS" role="transitionState" />
  </me:transitionState>
  <me:MCRCMethod name="RRKM" />
        <me:tunneling name="Eckart" />
</reaction>
<reaction id="R4" >
      <!--reversible="true"-->
  <reactantList>
    <reactant>
      <molecule ref="Pentanal-AcylOO-rad" role="modelled" />
```

```
      </reactant>
    </reactantList>
    <productList>
      <product>
        <molecule ref="Pentanal-17-rad" role="sink" />
      </product>
    </productList>
    <me:transitionState>
      <molecule ref="17Hshift-TS" role="transitionState" />
    </me:transitionState>
    <me:MCRCMethod name="RRKM" />
          <me:tunneling name="Eckart" />
  </reaction>
</reactionList>
<me:conditions>
  <me:bathGas>N2</me:bathGas>
  <me:PTs>
    <me:PTpair units="Torr" P="760.0" T="298.0" precision="qd"  bathGas="N2" />
  </me:PTs>
</me:conditions>
<me:modelParameters>
  <!--Specify grain size directly...-->
  <me:grainSize units="cm-1">100</me:grainSize>
  <!--...or by the total number of grains
      <me:numberOfGrains> 500 </me:numberOfGrains>-->
  <!--Specify increased energy range
      <me:maxTemperature>6000</me:maxTemperature>-->
  <me:energyAboveTheTopHill>25.0</me:energyAboveTheTopHill>
</me:modelParameters>
<me:control>

  <me:printSpeciesProfile />
  <!--<me:testMicroRates />
        <me:testDOS />
  <me:testRateConstant />
  <me:printGrainDOS />-->
  <!--<me:printCellDOS />-->
  <!--<me:printReactionOperatorColumnSums />-->
  <!--<me:printTunnellingCoefficients />
  <me:printGrainkfE />-->
  <!--<me:printGrainBoltzmann />
  <me:printGrainkbE />-->
  <me:eigenvalues>3</me:eigenvalues>
  <!-- <me:hideInactive/>  Molecules and reactions with attribute active="false" are not shown
  <me:diagramEnergyOffset>0</me:diagramEnergyOffset>-->
  <!--Adjusts displayed energies to this values for the lowest species. -->
```

```
    <me:calcMethod  name="simpleCalc" />
    <me:ForceMacroDetailedBalance >true</me:ForceMacroDetailedBalance>
  </me:control>
  <metadataList xmlns:dc="http://purl.org/dc/elements/1.1/">
    <dc:title>Project name</dc:title>
    <dc:source>Acylperoxyrad-Pentanal.xml</dc:source>
    <dc:creator>Mesmer v6.0</dc:creator>
    <dc:date>20220117_101627</dc:date>
    <dc:contributor>ndprse</dc:contributor>
  </metadataList>
</me:mesmer>
```


Hexanal–AcOO ------> Hexanal–AcOOH


```
<?xml version="1.0" encoding="utf-8" ?>
<?xml-stylesheet type='text/xsl' href='../../mesmer2.xsl' media='other'?>
<?xml-stylesheet type='text/xsl' href='../../mesmer1.xsl' media='screen'?>
<me:mesmer                                                     xmlns="http://www.xml-cml.org/schema"
xmlns:me="http://www.chem.leeds.ac.uk/mesmer"
xmlns:xsi="http://www.w3.org/2001/XMLSchema-instance">
  <me:title>Project name</me:title>
  <moleculeList convention="">
    <molecule id="N2">
    <atomArray>
      <atom id="a1" elementType="N" />
      <atom id="a2" elementType="N" />
    </atomArray>
    <bondArray>
      <bond atomRefs2="a2 a1" order="3"/>
    </bondArray>
    <propertyList>
      <property dictRef="me:epsilon">
        <scalar>48.0</scalar>
      </property>
      <property dictRef="me:sigma">
        <scalar>3.90</scalar>
      </property>
      <property dictRef="me:MW">
        <scalar units="amu">28.0</scalar>
      </property>
    </propertyList>
  </molecule>
    <molecule id="Hexanal-15-rad" spinMultiplicity="2">
      <atomArray>
        <atom id="a1" elementType="C" x3="1.182166" y3="-0.510110" z3="0.292476" />
```

<atom id="a2" elementType="O" x3="1.199883" y3="-0.008924" z3="1.388278" />
<atom id="a3" elementType="C" x3="0.363879" y3="-1.701687" z3="-0.145222" />
<atom id="a4" elementType="H" x3="0.893939" y3="-2.234296" z3="-0.932176" />
<atom id="a5" elementType="H" x3="0.273943" y3="-2.348290" z3="0.729775" />
<atom id="a6" elementType="C" spinMultiplicity="2" x3="-0.974650" y3="-1.233315" z3="-
0.614648" />
<atom id="a7" elementType="C" x3="-2.006163" y3="-0.709201" z3="0.318036" />
<atom id="a8" elementType="H" x3="-2.841277" y3="-1.419556" z3="0.375230" />
<atom id="a9" elementType="H" x3="-1.592264" y3="-0.640726" z3="1.327469" />
<atom id="a10" elementType="C" x3="-2.562223" y3="0.659128" z3="-0.098426" />
<atom id="a11" elementType="H" x3="-3.377115" y3="0.925073" z3="0.577473" />
<atom id="a12" elementType="H" x3="-3.001930" y3="0.575853" z3="-1.095703" />
<atom id="a13" elementType="O" x3="1.880445" y3="0.001474" z3="-0.732973" />
<atom id="a14" elementType="H" x3="-1.142844" y3="-1.157243" z3="-1.681169" />
<atom id="a15" elementType="C" x3="-1.506345" y3="1.757132" z3="-0.090476" />
<atom id="a16" elementType="H" x3="-1.051735" y3="1.852533" z3="0.896641" />
<atom id="a17" elementType="H" x3="-0.706663" y3="1.542919" z3="-0.802124" />
<atom id="a18" elementType="H" x3="-1.937625" y3="2.720964" z3="-0.359482" />
<atom id="a19" elementType="O" x3="2.567833" y3="1.178395" z3="-0.354781" />
<atom id="a20" elementType="H" x3="2.318303" y3="1.243524" z3="0.589424" />
</atomArray>
<bondArray>
<bond atomRefs2="a14 a6" order="1" />
<bond atomRefs2="a12 a10" order="1" />
<bond atomRefs2="a4 a3" order="1" />
<bond atomRefs2="a17 a15" order="1" />
<bond atomRefs2="a13 a19" order="1" />
<bond atomRefs2="a13 a1" order="1" />
<bond atomRefs2="a6 a3" order="1" />
<bond atomRefs2="a6 a7" order="1" />
<bond atomRefs2="a18 a15" order="1" />
<bond atomRefs2="a19 a20" order="1" />
<bond atomRefs2="a3 a1" order="1" />
<bond atomRefs2="a3 a5" order="1" />
<bond atomRefs2="a10 a15" order="1" />
<bond atomRefs2="a10 a7" order="1" />
<bond atomRefs2="a10 a11" order="1" />
<bond atomRefs2="a15 a16" order="1" />
<bond atomRefs2="a1 a2" order="2" />
<bond atomRefs2="a7 a8" order="1" />
<bond atomRefs2="a7 a9" order="1" />
</bondArray>
<propertyList>
<property title="program">
<scalar>Gaussian 16, Revision C.01</scalar>
</property>

<property title="basis">
<scalar>Aug-CC-pVTZ (5D, 7F)</scalar>
</property>
<property title="File Format">
<scalar>g03</scalar>
</property>
<property title="Energy" dictRef="me:ZPE">
<scalar units="kcal/mol">2.92</scalar>
</property>
<property title="SpinMultiplicity" dictRef="me:spinMultiplicity">
<scalar>2.00</scalar>
</property>
<property title="Vibrational Frequencies" dictRef="me:vibFreqs">
<array units="cm-1">29.20 45.90 75.83 125.82 157.00 230.62 251.30 299.65 316.72 372.13 421.89 436.88 485.16 497.78 653.63 778.50 784.72 840.87 872.57 913.00 939.12 997.06 1030.46 1063.82 1080.45 1114.49 1151.85 1207.51 1248.00 1283.30 1296.40 1324.87 1354.04 1382.12 1421.37 1430.00 1473.27 1478.25 1498.62 1505.06 1512.35 1517.77 1813.82 2990.23 3044.64 3045.33 3063.37 3068.56 3085.60 3116.44 3126.15 3136.57 3189.86 3561.26</array>
</property>
<property title="Rotational Constants" dictRef="me:rotConsts">
<array units="cm-1">0.078 0.036 0.030</array>
</property>
<property title="Symmetry Number" dictRef="me:symmetryNumber">
<scalar>1</scalar>
</property>
</propertyList>
</molecule>
<molecule id="15Hshift-TS" spinMultiplicity="2">
<atomArray>
<atom id="a1" elementType="C" x3="-1.893790" y3="-0.404622" z3="-0.227985" />
<atom id="a2" elementType="O" x3="-2.552360" y3="-1.209049" z3="-0.807831" />
<atom id="a3" elementType="C" x3="-1.181726" y3="-0.609024" z3="1.095231" />
<atom id="a4" elementType="H" x3="-1.814279" y3="-0.189617" z3="1.878496" />
<atom id="a5" elementType="H" x3="-1.123408" y3="-1.687208" z3="1.243015" />
<atom id="a6" elementType="C" spinMultiplicity="2" x3="0.172754" y3="0.053711" z3="1.104679" />
<atom id="a7" elementType="C" x3="1.240232" y3="-0.534212" z3="0.225181" />
<atom id="a8" elementType="H" x3="1.502057" y3="-1.524740" z3="0.619758" />
<atom id="a9" elementType="H" x3="0.838002" y3="-0.710402" z3="-0.777122" />
<atom id="a10" elementType="C" x3="2.495193" y3="0.326234" z3="0.132035" />
<atom id="a11" elementType="H" x3="2.888241" y3="0.498666" z3="1.137375" />
<atom id="a12" elementType="H" x3="2.223236" y3="1.306224" z3="-0.266643" />
<atom id="a13" elementType="O" x3="-1.675959" y3="0.807432" z3="-0.830949" />
<atom id="a14" elementType="O" x3="-1.120671" y3="1.778127" z3="-0.001530" />
<atom id="a15" elementType="H" x3="0.513847" y3="0.368054" z3="2.089249" />
<atom id="a16" elementType="H" x3="-0.205564" y3="1.158842" z3="0.566050" />

<atom id="a17" elementType="C" x3="3.571726" y3="-0.303655" z3="-0.740595" />
<atom id="a18" elementType="H" x3="3.208514" y3="-0.458344" z3="-1.757617" />
<atom id="a19" elementType="H" x3="4.456328" y3="0.329749" z3="-0.794939" />
<atom id="a20" elementType="H" x3="3.878607" y3="-1.273888" z3="-0.346421" />
</atomArray>
<bondArray>
<bond atomRefs2="a18 a17" order="1" />
<bond atomRefs2="a13 a1" order="1" />
<bond atomRefs2="a13 a14" order="1" />
<bond atomRefs2="a2 a1" order="2" />
<bond atomRefs2="a19 a17" order="1" />
<bond atomRefs2="a9 a7" order="1" />
<bond atomRefs2="a17 a20" order="1" />
<bond atomRefs2="a17 a10" order="1" />
<bond atomRefs2="a12 a10" order="1" />
<bond atomRefs2="a1 a3" order="1" />
<bond atomRefs2="a14 a16" order="1" />
<bond atomRefs2="a10 a7" order="1" />
<bond atomRefs2="a10 a11" order="1" />
<bond atomRefs2="a7 a8" order="1" />
<bond atomRefs2="a7 a6" order="1" />
<bond atomRefs2="a3 a6" order="1" />
<bond atomRefs2="a3 a5" order="1" />
<bond atomRefs2="a3 a4" order="1" />
<bond atomRefs2="a6 a15" order="1" />
</bondArray>
<propertyList>
<property title="program">
<scalar>Gaussian 16, Revision C.01</scalar>
</property>
<property title="basis">
<scalar>Aug-CC-pVTZ (5D, 7F)</scalar>
</property>
<property title="File Format">
<scalar>g03</scalar>
</property>
<property title="Energy" dictRef="me:ZPE">
<scalar units="kcal/mol">20.49</scalar>
</property>
<property title="SpinMultiplicity" dictRef="me:spinMultiplicity">
<scalar>2.00</scalar>
</property>
<property title="Vibrational Frequencies" dictRef="me:vibFreqs">
<array units="cm-1">59.27 71.05 73.63 153.05 181.31 243.45 246.27 354.04 400.29 479.95 503.27 553.82 590.44 703.45 739.02 780.38 837.03 879.38 939.04 954.20 1029.87 1055.39 1069.36 1081.49 1127.64 1154.57 1190.28 1219.26 1239.85 1271.52 1294.14 1323.44 1343.54 1381.22 1425.26

1426.71 1457.98 1474.88 1499.50 1506.78 1512.86 1574.94 1873.74 3001.29 3041.58 3046.11 3054.03 3075.72 3082.65 3116.94 3121.45 3127.91 3130.63</array>
</property>
<property title="ImaginaryFrequency" dictRef="me:imFreqs">
<scalar units="cm-1">1704.50</scalar>
</property>
<property title="Rotational Constants" dictRef="me:rotConsts">
<array units="cm-1">0.093 0.027 0.026</array>
</property>
<property title="Symmetry Number" dictRef="me:symmetryNumber">
<scalar>1</scalar>
</property>
<property dictRef="me:frequenciesScaleFactor" >
<scalar>1</scalar>
</property>
</propertyList>
<me:DOSCMethod name="ClassicalRotors" />
</molecule>
<molecule id="Hexanal-16-rad" spinMultiplicity="2">
<atomArray>
<atom id="a1" elementType="C" x3="-1.478951" y3="0.318188" z3="0.159405" />
<atom id="a2" elementType="O" x3="-1.549901" y3="0.077280" z3="1.338001" />
<atom id="a3" elementType="C" x3="-0.732586" y3="1.451922" z3="-0.481746" />
<atom id="a4" elementType="H" x3="-0.771023" y3="1.345715" z3="-1.564878" />
<atom id="a5" elementType="H" x3="-1.262237" y3="2.368041" z3="-0.215647" />
<atom id="a6" elementType="C" x3="0.712534" y3="1.508179" z3="0.016293" />
<atom id="a7" elementType="H" x3="0.713177" y3="1.614563" z3="1.104333" />
<atom id="a8" elementType="H" x3="1.167408" y3="2.428844" z3="-0.374847" />
<atom id="a9" elementType="C" spinMultiplicity="2" x3="1.509637" y3="0.317908" z3="-0.379440" />
<atom id="a10" elementType="C" x3="2.756388" y3="-0.049363" z3="0.337785" />
<atom id="a11" elementType="H" x3="2.554119" y3="-0.086773" z3="1.414755" />
<atom id="a12" elementType="H" x3="3.501816" y3="0.751468" z3="0.220410" />
<atom id="a13" elementType="O" x3="-2.104919" y3="-0.438927" z3="-0.754941" />
<atom id="a14" elementType="H" x3="1.266476" y3="-0.186914" z3="-1.308377" />
<atom id="a15" elementType="C" x3="3.359983" y3="-1.370584" z3="-0.123067" />
<atom id="a16" elementType="H" x3="4.272789" y3="-1.600023" z3="0.425600" />
<atom id="a17" elementType="H" x3="2.658317" y3="-2.190972" z3="0.029792" />
<atom id="a18" elementType="H" x3="3.606850" y3="-1.335729" z3="-1.185064" />
<atom id="a19" elementType="O" x3="-2.826492" y3="-1.491848" z3="-0.147810" />
<atom id="a20" elementType="H" x3="-2.619227" y3="-1.337754" z3="0.796545" />
</atomArray>
<bondArray>
<bond atomRefs2="a4 a3" order="1" />
<bond atomRefs2="a14 a9" order="1" />
<bond atomRefs2="a18 a15" order="1" />

<bond atomRefs2="a13 a19" order="1" />
<bond atomRefs2="a13 a1" order="1" />
<bond atomRefs2="a3 a5" order="1" />
<bond atomRefs2="a3 a6" order="1" />
<bond atomRefs2="a3 a1" order="1" />
<bond atomRefs2="a9 a6" order="1" />
<bond atomRefs2="a9 a10" order="1" />
<bond atomRefs2="a8 a6" order="1" />
<bond atomRefs2="a19 a20" order="1" />
<bond atomRefs2="a15 a17" order="1" />
<bond atomRefs2="a15 a10" order="1" />
<bond atomRefs2="a15 a16" order="1" />
<bond atomRefs2="a6 a7" order="1" />
<bond atomRefs2="a1 a2" order="2" />
<bond atomRefs2="a12 a10" order="1" />
<bond atomRefs2="a10 a11" order="1" />
</bondArray>
<propertyList>
<property title="program">
<scalar>Gaussian 16, Revision C.01</scalar>
</property>
<property title="basis">
<scalar>Aug-CC-pVTZ (5D, 7F)</scalar>
</property>
<property title="File Format">
<scalar>g03</scalar>
</property>
<property title="Energy" dictRef="me:ZPE">
<scalar units="kcal/mol">1.75</scalar>
</property>
<property title="SpinMultiplicity" dictRef="me:spinMultiplicity">
<scalar>2.00</scalar>
</property>
<property title="Vibrational Frequencies" dictRef="me:vibFreqs">
<array units="cm-1">21.39 27.54 41.76 101.07 163.98 196.55 234.63 260.38 328.62 366.85 408.01 417.24 460.38 496.84 631.62 714.82 774.50 873.31 902.98 949.23 973.44 1008.97 1044.20 1066.17 1072.33 1126.38 1155.25 1191.84 1227.69 1273.50 1281.67 1305.02 1378.99 1400.15 1422.01 1444.25 1468.91 1478.68 1483.01 1504.71 1509.58 1511.51 1822.04 2962.70 2979.87 3017.16 3049.36 3062.24 3077.37 3119.36 3128.68 3132.20 3162.66 3551.15</array>
</property>
<property title="Rotational Constants" dictRef="me:rotConsts">
<array units="cm-1">0.085 0.026 0.023</array>
</property>
<property title="Symmetry Number" dictRef="me:symmetryNumber">
<scalar>1</scalar>
</property>

</propertyList>
</molecule>
<molecule id="16Hshift-TS" spinMultiplicity="2">
<atomArray>
<atom id="a1" elementType="C" x3="1.882031" y3="-0.147797" z3="-0.184359" />
<atom id="a2" elementType="O" x3="2.800408" y3="-0.113215" z3="-0.944786" />
<atom id="a3" elementType="C" x3="1.377249" y3="1.011404" z3="0.635748" />
<atom id="a4" elementType="H" x3="1.283075" y3="0.727924" z3="1.682758" />
<atom id="a5" elementType="H" x3="2.128321" y3="1.790843" z3="0.544615" />
<atom id="a6" elementType="C" x3="0.022924" y3="1.513223" z3="0.092244" />
<atom id="a7" elementType="H" x3="0.103432" y3="1.636609" z3="-0.989408" />
<atom id="a8" elementType="H" x3="-0.158332" y3="2.506557" z3="0.508266" />
<atom id="a9" elementType="C" spinMultiplicity="2" x3="-1.147657" y3="0.610896" z3="0.421747" />
<atom id="a10" elementType="C" x3="-2.179500" y3="0.383831" z3="-0.648434" />
<atom id="a11" elementType="H" x3="-1.679912" y3="0.031325" z3="-1.554444" />
<atom id="a12" elementType="H" x3="-2.620422" y3="1.354400" z3="-0.908431" />
<atom id="a13" elementType="O" x3="1.153747" y3="-1.312983" z3="-0.145461" />
<atom id="a14" elementType="O" x3="0.256554" y3="-1.383829" z3="0.911159" />
<atom id="a15" elementType="H" x3="-1.569116" y3="0.792833" z3="1.412021" />
<atom id="a16" elementType="H" x3="-0.593814" y3="-0.527192" z3="0.650583" />
<atom id="a17" elementType="C" x3="-3.274601" y3="-0.590214" z3="-0.237137" />
<atom id="a18" elementType="H" x3="-3.802875" y3="-0.234687" z3="0.648684" />
<atom id="a19" elementType="H" x3="-4.005357" y3="-0.717396" z3="-1.034467" />
<atom id="a20" elementType="H" x3="-2.853349" y3="-1.569060" z3="-0.006314" />
</atomArray>
<bondArray>
<bond atomRefs2="a11 a10" order="1" />
<bond atomRefs2="a19 a17" order="1" />
<bond atomRefs2="a7 a6" order="1" />
<bond atomRefs2="a2 a1" order="2" />
<bond atomRefs2="a12 a10" order="1" />
<bond atomRefs2="a10 a17" order="1" />
<bond atomRefs2="a10 a9" order="1" />
<bond atomRefs2="a17 a20" order="1" />
<bond atomRefs2="a17 a18" order="1" />
<bond atomRefs2="a1 a13" order="1" />
<bond atomRefs2="a1 a3" order="1" />
<bond atomRefs2="a13 a14" order="1" />
<bond atomRefs2="a6 a9" order="1" />
<bond atomRefs2="a6 a8" order="1" />
<bond atomRefs2="a6 a3" order="1" />
<bond atomRefs2="a9 a15" order="1" />
<bond atomRefs2="a5 a3" order="1" />
<bond atomRefs2="a3 a4" order="1" />
<bond atomRefs2="a16 a14" order="1" />

</bondArray>
<propertyList>
<property title="program">
<scalar>Gaussian 16, Revision C.01</scalar>
</property>
<property title="basis">
<scalar>Aug-CC-pVTZ (5D, 7F)</scalar>
</property>
<property title="File Format">
<scalar>g03</scalar>
</property>
<property title="Energy" dictRef="me:ZPE">
<scalar units="kcal/mol">19.09</scalar>
</property>
<property title="SpinMultiplicity" dictRef="me:spinMultiplicity">
<scalar>2.00</scalar>
</property>
<property title="Vibrational Frequencies" dictRef="me:vibFreqs">
<array units="cm-1">28.09 81.42 106.27 167.70 230.92 249.65 304.48 358.81 397.48 440.62 517.05 542.16 583.08 735.20 761.95 769.44 869.45 900.09 947.92 952.73 1021.62 1056.54 1075.45 1102.92 1125.52 1143.03 1174.72 1206.24 1231.96 1284.19 1307.01 1311.27 1367.47 1373.59 1412.27 1426.27 1447.72 1477.50 1488.58 1497.65 1506.64 1509.88 1871.60 3006.72 3049.45 3050.43 3063.66 3081.44 3095.04 3105.43 3122.01 3133.27 3161.17</array>
</property>
<property title="ImaginaryFrequency" dictRef="me:imFreqs">
<scalar units="cm-1">1743.82</scalar>
</property>
<property title="Rotational Constants" dictRef="me:rotConsts">
<array units="cm-1">0.094 0.032 0.028</array>
</property>
<property title="Symmetry Number" dictRef="me:symmetryNumber">
<scalar>1</scalar>
</property>
<property dictRef="me:frequenciesScaleFactor" >
<scalar>1</scalar>
</property>
</propertyList>
<me:DOSCMethod name="ClassicalRotors" />
</molecule>
<molecule id="Hexanal-17-rad" spinMultiplicity="2">
<atomArray>
<atom id="a1" elementType="C" x3="1.358202" y3="-1.371831" z3="0.350536" />
<atom id="a2" elementType="H" x3="1.437529" y3="-0.951559" z3="1.354147" />
<atom id="a3" elementType="H" x3="1.898608" y3="-2.319209" z3="0.357149" />
<atom id="a4" elementType="C" x3="2.016334" y3="-0.425473" z3="-0.671563" />
<atom id="a5" elementType="H" x3="1.939690" y3="-0.877033" z3="-1.663202" />

<atom id="a6" elementType="C" spinMultiplicity="2" x3="1.449342" y3="0.950632" z3="-0.689490" />
<atom id="a7" elementType="H" x3="0.766285" y3="1.232407" z3="-1.479128" />
<atom id="a8" elementType="C" x3="-0.111290" y3="-1.663331" z3="0.044348" />
<atom id="a9" elementType="H" x3="-0.239244" y3="-1.988929" z3="-0.987409" />
<atom id="a10" elementType="H" x3="-0.472348" y3="-2.462206" z3="0.694481" />
<atom id="a11" elementType="C" x3="-0.999442" y3="-0.483981" z3="0.310669" />
<atom id="a12" elementType="O" x3="-1.082889" y3="0.137416" z3="1.341432" />
<atom id="a13" elementType="C" x3="1.688535" y3="1.890687" z3="0.433737" />
<atom id="a14" elementType="H" x3="1.464761" y3="2.919302" z3="0.154121" />
<atom id="a15" elementType="H" x3="1.061651" y3="1.649979" z3="1.302433" />
<atom id="a16" elementType="H" x3="2.726955" y3="1.844696" z3="0.775403" />
<atom id="a17" elementType="O" x3="-1.767399" y3="-0.192039" z3="-0.751458" />
<atom id="a18" elementType="H" x3="3.083449" y3="-0.376415" z3="-0.426956" />
<atom id="a19" elementType="O" x3="-2.613665" y3="0.908854" z3="-0.488322" />
<atom id="a20" elementType="H" x3="-2.365802" y3="1.114901" z3="0.436313" />
</atomArray>
<bondArray>
<bond atomRefs2="a5 a4" order="1" />
<bond atomRefs2="a7 a6" order="1" />
<bond atomRefs2="a9 a8" order="1" />
<bond atomRefs2="a17 a19" order="1" />
<bond atomRefs2="a17 a11" order="1" />
<bond atomRefs2="a6 a4" order="1" />
<bond atomRefs2="a6 a13" order="1" />
<bond atomRefs2="a4 a18" order="1" />
<bond atomRefs2="a4 a1" order="1" />
<bond atomRefs2="a19 a20" order="1" />
<bond atomRefs2="a8 a11" order="1" />
<bond atomRefs2="a8 a1" order="1" />
<bond atomRefs2="a8 a10" order="1" />
<bond atomRefs2="a14 a13" order="1" />
<bond atomRefs2="a11 a12" order="2" />
<bond atomRefs2="a1 a3" order="1" />
<bond atomRefs2="a1 a2" order="1" />
<bond atomRefs2="a13 a16" order="1" />
<bond atomRefs2="a13 a15" order="1" />
</bondArray>
<propertyList>
<property title="program">
<scalar>Gaussian 16, Revision C.01</scalar>
</property>
<property title="basis">
<scalar>Aug-CC-pVTZ (5D, 7F)</scalar>
</property>
<property title="File Format">

<scalar>g03</scalar>
</property>
<property title="Energy" dictRef="me:ZPE">
<scalar units="kcal/mol">1.44</scalar>
</property>
<property title="SpinMultiplicity" dictRef="me:spinMultiplicity">
<scalar>2.00</scalar>
</property>
<property title="Vibrational Frequencies" dictRef="me:vibFreqs">
<array units="cm-1">46.71 56.33 90.43 118.02 128.08 201.64 255.65 265.19 339.86 378.04 410.01 441.70 488.79 518.68 624.76 728.62 821.51 863.02 906.56 938.19 965.89 1000.04 1016.70 1020.56 1064.03 1120.32 1160.65 1195.56 1229.23 1257.78 1303.55 1359.88 1371.99 1401.04 1401.86 1426.29 1476.40 1479.06 1489.31 1490.89 1501.14 1523.95 1814.74 2994.33 3012.72 3050.60 3064.36 3072.08 3077.43 3111.45 3119.93 3128.52 3198.56 3559.79</array>
</property>
<property title="Rotational Constants" dictRef="me:rotConsts">
<array units="cm-1">0.073 0.040 0.032</array>
</property>
<property title="Symmetry Number" dictRef="me:symmetryNumber">
<scalar>1</scalar>
</property>
</propertyList>
</molecule>
<molecule id="17Hshift-TS" spinMultiplicity="2">
<atomArray>
<atom id="a1" elementType="C" x3="0.419071" y3="1.675348" z3="0.177771" />
<atom id="a2" elementType="H" x3="0.528040" y3="2.747147" z3="0.012357" />
<atom id="a3" elementType="H" x3="0.345610" y3="1.545815" z3="1.259017" />
<atom id="a4" elementType="C" x3="1.679286" y3="0.998405" z3="-0.364365" />
<atom id="a5" elementType="H" x3="2.547202" y3="1.466717" z3="0.114895" />
<atom id="a6" elementType="C" spinMultiplicity="2" x3="1.789212" y3="-0.494129" z3="-0.161957" />
<atom id="a7" elementType="H" x3="2.624383" y3="-0.927690" z3="-0.711395" />
<atom id="a8" elementType="C" x3="-0.908542" y3="1.245080" z3="-0.477318" />
<atom id="a9" elementType="H" x3="-1.646655" y3="2.026737" z3="-0.312613" />
<atom id="a10" elementType="H" x3="-0.776538" y3="1.101942" z3="-1.547081" />
<atom id="a11" elementType="C" x3="-1.513684" y3="0.020527" z3="0.161182" />
<atom id="a12" elementType="O" x3="-2.254782" y3="0.039228" z3="1.097668" />
<atom id="a13" elementType="C" x3="1.649079" y3="-1.038035" z3="1.232909" />
<atom id="a14" elementType="H" x3="1.716246" y3="-2.124141" z3="1.241012" />
<atom id="a15" elementType="H" x3="2.451728" y3="-0.645791" z3="1.866151" />
<atom id="a16" elementType="H" x3="0.703958" y3="-0.758058" z3="1.697702" />
<atom id="a17" elementType="O" x3="-1.178303" y3="-1.222774" z3="-0.317882" />
<atom id="a18" elementType="H" x3="1.768748" y3="1.216261" z3="-1.430884" />
<atom id="a19" elementType="O" x3="-0.285410" y3="-1.206213" z3="-1.375608" />
<atom id="a20" elementType="H" x3="0.798700" y3="-0.974043" z3="-0.831928" />

</atomArray>
<bondArray>
<bond atomRefs2="a10 a8" order="1" />
<bond atomRefs2="a18 a4" order="1" />
<bond atomRefs2="a19 a20" order="1" />
<bond atomRefs2="a19 a17" order="1" />
<bond atomRefs2="a7 a6" order="1" />
<bond atomRefs2="a8 a9" order="1" />
<bond atomRefs2="a8 a11" order="1" />
<bond atomRefs2="a8 a1" order="1" />
<bond atomRefs2="a4 a6" order="1" />
<bond atomRefs2="a4 a5" order="1" />
<bond atomRefs2="a4 a1" order="1" />
<bond atomRefs2="a17 a11" order="1" />
<bond atomRefs2="a6 a13" order="1" />
<bond atomRefs2="a2 a1" order="1" />
<bond atomRefs2="a11 a12" order="2" />
<bond atomRefs2="a1 a3" order="1" />
<bond atomRefs2="a13 a14" order="1" />
<bond atomRefs2="a13 a16" order="1" />
<bond atomRefs2="a13 a15" order="1" />
</bondArray>
<propertyList>
<property title="program">
<scalar>Gaussian 16, Revision C.01</scalar>
</property>
<property title="basis">
<scalar>Aug-CC-pVTZ (5D, 7F)</scalar>
</property>
<property title="File Format">
<scalar>g03</scalar>
</property>
<property title="Energy" dictRef="me:ZPE">
<scalar units="kcal/mol">18.07</scalar>
</property>
<property title="SpinMultiplicity" dictRef="me:spinMultiplicity">
<scalar>2.00</scalar>
</property>
<property title="Vibrational Frequencies" dictRef="me:vibFreqs">
<array units="cm-1">70.32 118.03 167.80 175.57 214.42 284.12 326.82 336.22 411.22 505.55 518.79 569.28 603.29 711.75 760.77 805.75 846.64 886.77 916.62 960.47 1004.16 1034.29 1082.75 1097.69 1120.71 1134.00 1170.81 1210.45 1231.63 1272.14 1322.82 1361.80 1373.11 1393.38 1404.49 1423.83 1452.15 1475.24 1487.91 1491.51 1493.40 1514.02 1861.37 3014.20 3026.47 3064.70 3076.76 3097.83 3102.05 3108.01 3112.53 3143.95 3157.97</array>
</property>
<property title="ImaginaryFrequency" dictRef="me:imFreqs">

<scalar units="cm-1">1733.34</scalar>
</property>
<property title="Rotational Constants" dictRef="me:rotConsts">
<array units="cm-1">0.069 0.046 0.039</array>
</property>
<property title="Symmetry Number" dictRef="me:symmetryNumber">
<scalar>1</scalar>
</property>
<property dictRef="me:frequenciesScaleFactor" >
<scalar>1</scalar>
</property>
</propertyList>
<me:DOSCMethod name="ClassicalRotors" />
</molecule>
<molecule id="Hexanal-18-rad" spinMultiplicity="2">
<atomArray>
<atom id="a1" elementType="C" x3="1.315647" y3="0.407440" z3="0.244453" />
<atom id="a2" elementType="O" x3="1.977463" y3="1.082023" z3="-0.503863" />
<atom id="a3" elementType="C" x3="0.082936" y3="0.831533" z3="0.985263" />
<atom id="a4" elementType="H" x3="-0.283269" y3="0.003818" z3="1.589596" />
<atom id="a5" elementType="H" x3="0.384350" y3="1.630971" z3="1.663144" />
<atom id="a6" elementType="C" x3="-0.995279" y3="1.346895" z3="0.027294" />
<atom id="a7" elementType="H" x3="-0.611178" y3="2.225291" z3="-0.491674" />
<atom id="a8" elementType="H" x3="-1.842712" y3="1.674694" z3="0.631227" />
<atom id="a9" elementType="C" x3="-1.460840" y3="0.317892" z3="-1.001576" />
<atom id="a10" elementType="C" x3="-2.040589" y3="-0.965776" z3="-0.402095" />
<atom id="a11" elementType="H" x3="-1.267829" y3="-1.515102" z3="0.142991" />
<atom id="a12" elementType="H" x3="-2.315384" y3="-1.630737" z3="-1.233628" />
<atom id="a13" elementType="C" spinMultiplicity="2" x3="-3.220462" y3="-0.744892" z3="0.469371" />
<atom id="a14" elementType="H" x3="3.005555" y3="-0.448252" z3="-0.684917" />
<atom id="a15" elementType="H" x3="-3.894921" y3="0.080426" z3="0.281787" />
<atom id="a16" elementType="O" x3="1.626332" y3="-0.874955" z3="0.496014" />
<atom id="a17" elementType="O" x3="2.784042" y3="-1.275089" z3="-0.209435" />
<atom id="a18" elementType="H" x3="-0.629995" y3="0.059708" z3="-1.662809" />
<atom id="a19" elementType="H" x3="-2.217637" y3="0.786729" z3="-1.634640" />
<atom id="a20" elementType="H" x3="-3.518150" y3="-1.481927" z3="1.200935" />
</atomArray>
<bondArray>
<bond atomRefs2="a18 a9" order="1" />
<bond atomRefs2="a19 a9" order="1" />
<bond atomRefs2="a12 a10" order="1" />
<bond atomRefs2="a9 a10" order="1" />
<bond atomRefs2="a9 a6" order="1" />
<bond atomRefs2="a14 a17" order="1" />
<bond atomRefs2="a2 a1" order="2" />

```
    <bond atomRefs2="a7 a6" order="1" />
    <bond atomRefs2="a10 a11" order="1" />
    <bond atomRefs2="a10 a13" order="1" />
    <bond atomRefs2="a17 a16" order="1" />
    <bond atomRefs2="a6 a8" order="1" />
    <bond atomRefs2="a6 a3" order="1" />
    <bond atomRefs2="a1 a16" order="1" />
    <bond atomRefs2="a1 a3" order="1" />
    <bond atomRefs2="a15 a13" order="1" />
    <bond atomRefs2="a13 a20" order="1" />
    <bond atomRefs2="a3 a4" order="1" />
    <bond atomRefs2="a3 a5" order="1" />
   </bondArray>
   <propertyList>
    <property title="program">
     <scalar>Gaussian 16, Revision C.01</scalar>
    </property>
    <property title="basis">
     <scalar>Aug-CC-pVTZ (5D, 7F)</scalar>
    </property>
    <property title="File Format">
     <scalar>g03</scalar>
    </property>
    <property title="Energy" dictRef="me:ZPE">
     <scalar units="kcal/mol">4.66</scalar>
    </property>
    <property title="SpinMultiplicity" dictRef="me:spinMultiplicity">
     <scalar>2.00</scalar>
    </property>
    <property title="Vibrational Frequencies" dictRef="me:vibFreqs">
     <array units="cm-1">27.85 40.59 75.71 144.09 168.81 214.05 236.98 308.91 339.92 403.11
438.53 481.49 501.20 515.75 636.03 714.97 791.71 818.68 870.96 928.96 946.37 954.84 1022.24
1030.86 1080.92 1112.90 1133.72 1157.78 1218.43 1235.78 1286.33 1321.45 1372.81 1387.92 1402.87
1407.96 1470.41 1473.94 1489.44 1494.85 1503.42 1508.39 1820.31 2971.57 3046.55 3053.20 3066.13
3083.99 3089.36 3113.17 3139.10 3152.04 3256.25 3553.57</array>
    </property>
    <property title="Rotational Constants" dictRef="me:rotConsts">
     <array units="cm-1">0.094 0.030 0.026</array>
    </property>
    <property title="Symmetry Number" dictRef="me:symmetryNumber">
     <scalar>1</scalar>
    </property>
   </propertyList>
  </molecule>
  <molecule id="18Hshift-TS" spinMultiplicity="2">
   <atomArray>
```

<atom id="a1" elementType="C" x3="-1.516506" y3="-0.031659" z3="-0.072905" />
<atom id="a2" elementType="O" x3="-2.328011" y3="-0.522146" z3="-0.797981" />
<atom id="a3" elementType="C" x3="-0.754875" y3="-0.774349" z3="0.992426" />
<atom id="a4" elementType="H" x3="-0.227260" y3="-0.093538" z3="1.651245" />
<atom id="a5" elementType="H" x3="-1.500988" y3="-1.308487" z3="1.579241" />
<atom id="a6" elementType="C" x3="0.198814" y3="-1.793445" z3="0.354811" />
<atom id="a7" elementType="H" x3="-0.394938" y3="-2.529964" z3="-0.187274" />
<atom id="a8" elementType="H" x3="0.704964" y3="-2.325350" z3="1.163002" />
<atom id="a9" elementType="C" x3="1.243332" y3="-1.211723" z3="-0.598007" />
<atom id="a10" elementType="C" x3="2.201873" y3="-0.184738" z3="0.028783" />
<atom id="a11" elementType="H" x3="3.220438" y3="-0.385139" z3="-0.323128" />
<atom id="a12" elementType="H" x3="2.242689" y3="-0.314842" z3="1.113275" />
<atom id="a13" elementType="C" spinMultiplicity="2" x3="1.911937" y3="1.250975" z3="-
0.305390" />
<atom id="a14" elementType="H" x3="0.737293" y3="1.598505" z3="0.172772" />
<atom id="a15" elementType="H" x3="1.758316" y3="1.460309" z3="-1.362436" />
<atom id="a16" elementType="O" x3="-1.241036" y3="1.299197" z3="-0.294258" />
<atom id="a17" elementType="O" x3="-0.351500" y3="1.872470" z3="0.601007" />
<atom id="a18" elementType="H" x3="0.742318" y3="-0.764773" z3="-1.461121" />
<atom id="a19" elementType="H" x3="1.821418" y3="-2.047560" z3="-0.992688" />
<atom id="a20" elementType="H" x3="2.552681" y3="1.984301" z3="0.178654" />
</atomArray>
<bondArray>
<bond atomRefs2="a18 a9" order="1" />
<bond atomRefs2="a15 a13" order="1" />
<bond atomRefs2="a19 a9" order="1" />
<bond atomRefs2="a2 a1" order="2" />
<bond atomRefs2="a9 a10" order="1" />
<bond atomRefs2="a9 a6" order="1" />
<bond atomRefs2="a11 a10" order="1" />
<bond atomRefs2="a13 a10" order="1" />
<bond atomRefs2="a13 a20" order="1" />
<bond atomRefs2="a16 a1" order="1" />
<bond atomRefs2="a16 a17" order="1" />
<bond atomRefs2="a7 a6" order="1" />
<bond atomRefs2="a1 a3" order="1" />
<bond atomRefs2="a10 a12" order="1" />
<bond atomRefs2="a14 a17" order="1" />
<bond atomRefs2="a6 a3" order="1" />
<bond atomRefs2="a6 a8" order="1" />
<bond atomRefs2="a3 a5" order="1" />
<bond atomRefs2="a3 a4" order="1" />
</bondArray>
<propertyList>
<property title="program">
<scalar>Gaussian 16, Revision C.01</scalar>

```
      </property>
      <property title="basis">
        <scalar>Aug-CC-pVTZ (5D, 7F)</scalar>
      </property>
      <property title="File Format">
        <scalar>g03</scalar>
      </property>
      <property title="Energy" dictRef="me:ZPE">
        <scalar units="kcal/mol">22.21</scalar>
      </property>
      <property title="SpinMultiplicity" dictRef="me:spinMultiplicity">
        <scalar>2.00</scalar>
      </property>
      <property title="Vibrational Frequencies" dictRef="me:vibFreqs">
        <array units="cm-1">70.53 126.16 142.58 174.66 209.19 318.89 375.72 385.85 448.24 516.20
522.82 582.87 639.78 703.22 745.39 779.75 837.02 849.15 917.68 982.60 1006.61 1038.72 1076.43
1084.51 1112.95 1132.75 1149.19 1203.89 1239.79 1258.59 1282.14 1319.45 1363.64 1384.01 1398.65
1408.04 1430.71 1463.59 1476.72 1494.39 1503.77 1507.50 1866.45 3013.88 3045.87 3057.03 3066.00
3090.00 3097.05 3101.06 3107.50 3175.79 3176.29</array>
      </property>
      <property title="ImaginaryFrequency" dictRef="me:imFreqs">
        <scalar units="cm-1">1858.46</scalar>
      </property>
      <property title="Rotational Constants" dictRef="me:rotConsts">
        <array units="cm-1">0.068 0.048 0.034</array>
      </property>
      <property title="Symmetry Number" dictRef="me:symmetryNumber">
        <scalar>1</scalar>
      </property>
      <property dictRef="me:frequenciesScaleFactor" >
        <scalar>1</scalar>
      </property>
    </propertyList>
    <me:DOSCMethod  name="ClassicalRotors" />
  </molecule>
  <molecule id="Hexanal-AcylOO-rad" spinMultiplicity="2">
    <atomArray>
      <atom id="a1" elementType="C" x3="-3.193143" y3="2.987216" z3="0.000000" />
      <atom id="a2" elementType="H" x3="-3.336055" y3="4.067432" z3="0.000000" />
      <atom id="a3" elementType="H" x3="-3.695744" y3="2.583990" z3="0.880737" />
      <atom id="a4" elementType="H" x3="-3.695744" y3="2.583990" z3="-0.880737" />
      <atom id="a5" elementType="C" x3="-1.714936" y3="2.621449" z3="0.000000" />
      <atom id="a6" elementType="H" x3="-1.227489" y3="3.060911" z3="0.874111" />
      <atom id="a7" elementType="H" x3="-1.227489" y3="3.060911" z3="-0.874111" />
      <atom id="a8" elementType="C" x3="-1.477911" y3="1.116145" z3="0.000000" />
      <atom id="a9" elementType="H" x3="-1.964980" y3="0.674237" z3="0.874975" />
```

```
<atom id="a10" elementType="H" x3="-1.964980" y3="0.674237" z3="-0.874975" />
<atom id="a11" elementType="C" x3="0.000000" y3="0.747098" z3="0.000000" />
<atom id="a12" elementType="H" x3="0.490993" y3="1.182042" z3="-0.872396" />
<atom id="a13" elementType="H" x3="0.490993" y3="1.182042" z3="0.872396" />
<atom id="a14" elementType="C" x3="0.209251" y3="-0.760173" z3="0.000000" />
<atom id="a15" elementType="H" x3="-0.260360" y3="-1.230390" z3="0.866221" />
<atom id="a16" elementType="H" x3="-0.260360" y3="-1.230390" z3="-0.866221" />
<atom id="a17" elementType="C" x3="1.657628" y3="-1.128832" z3="0.000000" />
<atom id="a18" elementType="O" x3="2.605809" y3="-0.422566" z3="0.000000" />
<atom id="a19" elementType="O" x3="1.956557" y3="-2.531626" z3="0.000000" />
<atom id="a20" elementType="O" spinMultiplicity="2" x3="0.908367" y3="-3.309111" z3="0.000000" />
</atomArray>
<bondArray>
<bond atomRefs2="a4 a1" order="1" />
<bond atomRefs2="a10 a8" order="1" />
<bond atomRefs2="a7 a5" order="1" />
<bond atomRefs2="a12 a11" order="1" />
<bond atomRefs2="a16 a14" order="1" />
<bond atomRefs2="a1 a2" order="1" />
<bond atomRefs2="a1 a5" order="1" />
<bond atomRefs2="a1 a3" order="1" />
<bond atomRefs2="a5 a8" order="1" />
<bond atomRefs2="a5 a6" order="1" />
<bond atomRefs2="a8 a11" order="1" />
<bond atomRefs2="a8 a9" order="1" />
<bond atomRefs2="a11 a14" order="1" />
<bond atomRefs2="a11 a13" order="1" />
<bond atomRefs2="a14 a17" order="1" />
<bond atomRefs2="a14 a15" order="1" />
<bond atomRefs2="a17 a18" order="2" />
<bond atomRefs2="a17 a19" order="1" />
<bond atomRefs2="a19 a20" order="1" />
</bondArray>
<propertyList>
<property title="program">
<scalar>Gaussian 16, Revision C.01</scalar>
</property>
<property title="basis">
<scalar>Aug-CC-pVTZ (5D, 7F)</scalar>
</property>
<property title="File Format">
<scalar>g03</scalar>
</property>
<property title="Energy" dictRef="me:ZPE">
<scalar units="kcal/mol">0.0</scalar>
```

</property>
<property title="SpinMultiplicity" dictRef="me:spinMultiplicity">
<scalar>2.00</scalar>
</property>
<property title="Vibrational Frequencies" dictRef="me:vibFreqs">
<array units="cm-1">52.98 61.04 86.53 128.84 137.68 148.07 221.32 244.60 248.42 397.87 435.34 529.57 562.51 610.25 735.20 769.36 821.66 861.61 921.48 984.03 1031.03 1061.10 1075.88 1090.14 1140.20 1149.43 1234.55 1250.68 1282.63 1300.24 1344.26 1346.00 1353.73 1408.19 1422.62 1430.11 1451.59 1493.98 1496.94 1506.03 1506.95 1516.41 1922.97 3022.86 3039.56 3044.47 3051.73 3059.64 3071.94 3076.86 3095.70 3113.64 3115.47 3123.97</array>
</property>
<property title="Rotational Constants" dictRef="me:rotConsts">
<array units="cm-1">0.147 0.018 0.017</array>
</property>
<property title="Symmetry Number" dictRef="me:symmetryNumber">
<scalar>1</scalar>
</property>
<property dictRef="me:sigma" >
<scalar>7.02</scalar>
</property>
<property dictRef="me:epsilon" >
<scalar>359.0</scalar>
</property>
<property dictRef="me:frequenciesScaleFactor" >
<scalar>1</scalar>
</property>
</propertyList>
<me:DOSCMethod name="ClassicalRotors" />
<me:DistributionCalcMethod name="Boltzmann" />
<me:energyTransferModel name="ExponentialDown" />
<me:deltaEDown >225.0</me:deltaEDown>
</molecule>
<molecule id="Hexanal-14-rad" spinMultiplicity="2">
<atomArray>
<atom id="a1" elementType="C" x3="1.228654" y3="-0.205980" z3="0.203841" />
<atom id="a2" elementType="O" x3="1.225750" y3="0.686980" z3="1.029921" />
<atom id="a3" elementType="C" spinMultiplicity="2" x3="0.198553" y3="-1.175495" z3="-0.037787" />
<atom id="a4" elementType="O" x3="2.281668" y3="-0.383456" z3="-0.631061" />
<atom id="a5" elementType="O" x3="3.284702" y3="0.575417" z3="-0.387132" />
<atom id="a6" elementType="H" x3="0.360408" y3="-1.888943" z3="-0.835069" />
<atom id="a7" elementType="H" x3="2.879514" y3="1.085648" z3="0.345012" />
<atom id="a8" elementType="C" x3="-1.067945" y3="-1.180596" z3="0.724441" />
<atom id="a9" elementType="H" x3="-1.203361" y3="-2.170314" z3="1.171787" />
<atom id="a10" elementType="H" x3="-1.006846" y3="-0.460916" z3="1.539195" />
<atom id="a11" elementType="C" x3="-2.291644" y3="-0.889100" z3="-0.165382" />

<atom id="a12" elementType="H" x3="-2.371344" y3="-1.670568" z3="-0.924648" />
<atom id="a13" elementType="H" x3="-3.185359" y3="-0.966018" z3="0.458164" />
<atom id="a14" elementType="C" x3="-2.259545" y3="0.477889" z3="-0.845794" />
<atom id="a15" elementType="H" x3="-1.389044" y3="0.534702" z3="-1.505661" />
<atom id="a16" elementType="H" x3="-3.135194" y3="0.557363" z3="-1.492932" />
<atom id="a17" elementType="C" x3="-2.237946" y3="1.652439" z3="0.125586" />
<atom id="a18" elementType="H" x3="-1.320492" y3="1.672843" z3="0.713788" />
<atom id="a19" elementType="H" x3="-2.304505" y3="2.599042" z3="-0.409886" />
<atom id="a20" elementType="H" x3="-3.081503" y3="1.600692" z3="0.816992" />
</atomArray>
<bondArray>
<bond atomRefs2="a15 a14" order="1" />
<bond atomRefs2="a16 a14" order="1" />
<bond atomRefs2="a12 a11" order="1" />
<bond atomRefs2="a14 a11" order="1" />
<bond atomRefs2="a14 a17" order="1" />
<bond atomRefs2="a6 a3" order="1" />
<bond atomRefs2="a4 a5" order="1" />
<bond atomRefs2="a4 a1" order="1" />
<bond atomRefs2="a19 a17" order="1" />
<bond atomRefs2="a5 a7" order="1" />
<bond atomRefs2="a11 a13" order="1" />
<bond atomRefs2="a11 a8" order="1" />
<bond atomRefs2="a3 a1" order="1" />
<bond atomRefs2="a3 a8" order="1" />
<bond atomRefs2="a17 a18" order="1" />
<bond atomRefs2="a17 a20" order="1" />
<bond atomRefs2="a1 a2" order="2" />
<bond atomRefs2="a8 a9" order="1" />
<bond atomRefs2="a8 a10" order="1" />
</bondArray>
<propertyList>
<property title="program">
<scalar>Gaussian 16, Revision C.01</scalar>
</property>
<property title="basis">
<scalar>Aug-CC-pVTZ (5D, 7F)</scalar>
</property>
<property title="File Format">
<scalar>g03</scalar>
</property>
<property title="Energy" dictRef="me:ZPE">
<scalar units="kcal/mol" >-2.11</scalar>
</property>
<property title="SpinMultiplicity" dictRef="me:spinMultiplicity">
<scalar>2.00</scalar>

</property>
<property title="Vibrational Frequencies" dictRef="me:vibFreqs">
<array units="cm-1">37.66 51.74 93.05 149.55 174.51 216.81 273.37 284.16 366.06 392.20 444.90 485.76 501.25 625.26 711.52 764.45 769.74 819.03 878.89 917.97 965.06 999.91 1016.43 1082.48 1109.90 1112.38 1158.11 1193.80 1239.85 1261.67 1321.75 1349.24 1385.11 1395.29 1429.11 1462.71 1484.79 1497.72 1501.54 1509.26 1521.89 1529.47 1747.02 3032.42 3037.77 3045.67 3046.20 3079.28 3087.89 3116.84 3123.78 3131.37 3204.30 3543.60</array>
</property>
<property title="Rotational Constants" dictRef="me:rotConsts">
<array units="cm-1">0.097 0.028 0.025</array>
</property>
<property title="Symmetry Number" dictRef="me:symmetryNumber">
<scalar>1</scalar>
</property>
</propertyList>
</molecule>
<molecule id="14Hshift-TS" spinMultiplicity="2">
<atomArray>
<atom id="a1" elementType="C" x3="1.633960" y3="-0.262020" z3="-0.078668" />
<atom id="a2" elementType="O" x3="2.196212" y3="-1.169349" z3="-0.601553" />
<atom id="a3" elementType="C" x3="0.610453" y3="-0.323579" z3="1.024727" />
<atom id="a4" elementType="O" x3="1.862125" y3="1.028765" z3="-0.433857" />
<atom id="a5" elementType="O" spinMultiplicity="2" x3="1.006107" y3="1.864182" z3="0.304126" />
<atom id="a6" elementType="H" x3="1.091616" y3="-0.400921" z3="1.998690" />
<atom id="a7" elementType="H" x3="0.385368" y3="0.925720" z3="0.910566" />
<atom id="a8" elementType="C" x3="-0.572876" y3="-1.235511" z3="0.850279" />
<atom id="a9" elementType="H" x3="-0.212257" y3="-2.258645" z3="1.000317" />
<atom id="a10" elementType="H" x3="-1.270512" y3="-1.046014" z3="1.668020" />
<atom id="a11" elementType="C" x3="-1.281523" y3="-1.151393" z3="-0.500584" />
<atom id="a12" elementType="H" x3="-0.597079" y3="-1.486352" z3="-1.282627" />
<atom id="a13" elementType="H" x3="-2.104329" y3="-1.869870" z3="-0.485513" />
<atom id="a14" elementType="C" x3="-1.828276" y3="0.229071" z3="-0.865285" />
<atom id="a15" elementType="H" x3="-1.004931" y3="0.928051" z3="-1.025866" />
<atom id="a16" elementType="H" x3="-2.339522" y3="0.144577" z3="-1.825639" />
<atom id="a17" elementType="C" x3="-2.787201" y3="0.808556" z3="0.167533" />
<atom id="a18" elementType="H" x3="-3.605660" y3="0.116986" z3="0.377724" />
<atom id="a19" elementType="H" x3="-2.284467" y3="1.023691" z3="1.112130" />
<atom id="a20" elementType="H" x3="-3.221001" y3="1.743248" z3="-0.185536" />
</atomArray>
<bondArray>
<bond atomRefs2="a16 a14" order="1" />
<bond atomRefs2="a12 a11" order="1" />
<bond atomRefs2="a15 a14" order="1" />
<bond atomRefs2="a14 a11" order="1" />
<bond atomRefs2="a14 a17" order="1" />

<bond atomRefs2="a2 a1" order="2" />
<bond atomRefs2="a11 a13" order="1" />
<bond atomRefs2="a11 a8" order="1" />
<bond atomRefs2="a4 a1" order="1" />
<bond atomRefs2="a4 a5" order="1" />
<bond atomRefs2="a20 a17" order="1" />
<bond atomRefs2="a1 a3" order="1" />
<bond atomRefs2="a17 a18" order="1" />
<bond atomRefs2="a17 a19" order="1" />
<bond atomRefs2="a8 a9" order="1" />
<bond atomRefs2="a8 a3" order="1" />
<bond atomRefs2="a8 a10" order="1" />
<bond atomRefs2="a7 a3" order="1" />
<bond atomRefs2="a3 a6" order="1" />
</bondArray>
<propertyList>
<property title="program">
<scalar>Gaussian 16, Revision C.01</scalar>
</property>
<property title="basis">
<scalar>Aug-CC-pVTZ (5D, 7F)</scalar>
</property>
<property title="File Format">
<scalar>g03</scalar>
</property>
<property title="Energy" dictRef="me:ZPE">
<scalar units="kcal/mol">27.05</scalar>
</property>
<property title="SpinMultiplicity" dictRef="me:spinMultiplicity">
<scalar>2.00</scalar>
</property>
<property title="Vibrational Frequencies" dictRef="me:vibFreqs">
<array units="cm-1">34.78 63.86 102.14 153.56 182.85 210.20 287.08 300.13 395.09 481.84 513.09 546.30 651.31 692.96 728.31 787.82 825.15 878.87 905.79 975.94 983.31 1018.45 1054.59 1103.44 1122.98 1145.37 1174.10 1183.67 1236.42 1270.30 1327.71 1341.97 1374.79 1402.92 1407.05 1427.44 1465.71 1499.90 1501.65 1512.82 1517.69 1768.73 1913.28 3032.52 3041.92 3051.44 3059.58 3080.80 3087.84 3100.13 3113.53 3125.31 3126.15</array>
</property>
<property title="ImaginaryFrequency" dictRef="me:imFreqs">
<scalar units="cm-1">1637.38</scalar>
</property>
<property title="Rotational Constants" dictRef="me:rotConsts">
<array units="cm-1">0.077 0.038 0.031</array>
</property>
<property title="Symmetry Number" dictRef="me:symmetryNumber">
<scalar>1</scalar>

```
      </property>
      <property dictRef="me:frequenciesScaleFactor" >
        <scalar>1</scalar>
      </property>
    </propertyList>
    <me:DOSCMethod name="ClassicalRotors" />
  </molecule>
</moleculeList>
<reactionList>
<reaction id="R1" >
          <!--reversible="true"-->
    <reactantList>
      <reactant>
        <molecule ref="Hexanal-AcylOO-rad" role="modelled" />
      </reactant>
    </reactantList>
    <productList>
      <product>
        <molecule ref="Hexanal-14-rad" role="sink" />
      </product>
    </productList>
    <me:transitionState>
      <molecule ref="14Hshift-TS" role="transitionState" />
    </me:transitionState>
    <me:MCRCMethod name="RRKM" />
            <me:tunneling name="Eckart" />
  </reaction>
  <reaction id="R2" >
          <!--reversible="true"-->
    <reactantList>
      <reactant>
        <molecule ref="Hexanal-AcylOO-rad" role="modelled" />
      </reactant>
    </reactantList>
    <productList>
      <product>
        <molecule ref="Hexanal-15-rad" role="sink" />
      </product>
    </productList>
    <me:transitionState>
      <molecule ref="15Hshift-TS" role="transitionState" />
    </me:transitionState>
    <me:MCRCMethod name="RRKM" />
            <me:tunneling name="Eckart" />
  </reaction>
  <reaction id="R3" >
```

```
      <!--reversible="true"-->
  <reactantList>
    <reactant>
      <molecule ref="Hexanal-AcylOO-rad" role="modelled" />
    </reactant>
  </reactantList>
  <productList>
    <product>
      <molecule ref="Hexanal-16-rad" role="sink" />
    </product>
  </productList>
  <me:transitionState>
    <molecule ref="16Hshift-TS" role="transitionState" />
  </me:transitionState>
  <me:MCRCMethod name="RRKM" />
        <me:tunneling name="Eckart" />
</reaction>
<reaction id="R4" >
      <!--reversible="true"-->
  <reactantList>
    <reactant>
      <molecule ref="Hexanal-AcylOO-rad" role="modelled" />
    </reactant>
  </reactantList>
  <productList>
    <product>
      <molecule ref="Hexanal-17-rad" role="sink" />
    </product>
  </productList>
  <me:transitionState>
    <molecule ref="17Hshift-TS" role="transitionState" />
  </me:transitionState>
  <me:MCRCMethod name="RRKM" />
        <me:tunneling name="Eckart" />
</reaction>
<reaction id="R5" >
      <!--reversible="true"-->
  <reactantList>
    <reactant>
      <molecule ref="Hexanal-AcylOO-rad" role="modelled" />
    </reactant>
  </reactantList>
  <productList>
    <product>
      <molecule ref="Hexanal-18-rad" role="sink" />
    </product>
```

```
    </productList>
    <me:transitionState>
      <molecule ref="18Hshift-TS" role="transitionState" />
    </me:transitionState>
    <me:MCRCMethod name="RRKM" />
          <me:tunneling name="Eckart" />
  </reaction>
</reactionList>
<me:conditions>
  <me:bathGas>N2</me:bathGas>
  <me:PTs>
    <me:PTpair units="Torr" P="760." T="298." precision="qd"  bathGas="N2" />
  </me:PTs>
</me:conditions>
<me:modelParameters>
  <!--Specify grain size directly...-->
  <me:grainSize units="cm-1">100</me:grainSize>
  <!--...or by the total number of grains
      <me:numberOfGrains> 500 </me:numberOfGrains>-->
  <!--Specify increased energy range
      <me:maxTemperature>6000</me:maxTemperature>-->
  <me:energyAboveTheTopHill>25.0</me:energyAboveTheTopHill>
</me:modelParameters>
<me:control>
  <me:printSpeciesProfile />
  <!--<me:testMicroRates />
        <me:testDOS />
  <me:testRateConstant />
  <me:printGrainDOS />-->
  <!--<me:printCellDOS />-->
  <!--<me:printReactionOperatorColumnSums />-->
  <me:printTunnellingCoefficients />
  <!--<me:printGrainkfE />-->
  <!--<me:printGrainBoltzmann />
  <me:printGrainkbE />-->
  <me:eigenvalues>3</me:eigenvalues>
  <!-- <me:hideInactive/>  Molecules and reactions with attribute active="false" are not shown
  <me:diagramEnergyOffset>0</me:diagramEnergyOffset>-->
  <!--Adjusts displayed energies to this values for the lowest species. -->
  <me:calcMethod  name="simpleCalc" />
  <me:ForceMacroDetailedBalance >true</me:ForceMacroDetailedBalance>
</me:control>
<metadataList xmlns:dc="http://purl.org/dc/elements/1.1/">
  <dc:title>Project name</dc:title>
  <dc:source>Acylperoxyrad-Hexanal.xml</dc:source>
  <dc:creator>Mesmer v6.0</dc:creator>
```

```
    <dc:date>20220118_085722</dc:date>
    <dc:contributor>ndprse</dc:contributor>
  </metadataList>
</me:mesmer>
```


Heptanal–AcOO ------> Heptanal–AcOOH


```
<?xml version="1.0" encoding="utf-8" ?>
<?xml-stylesheet type='text/xsl' href='../../mesmer2.xsl' media='other'?>
<?xml-stylesheet type='text/xsl' href='../../mesmer1.xsl' media='screen'?>
<me:mesmer xmlns="http://www.xml-cml.org/schema"
xmlns:me="http://www.chem.leeds.ac.uk/mesmer"
xmlns:xsi="http://www.w3.org/2001/XMLSchema-instance">
  <me:title>Project name</me:title>
  <moleculeList convention="">
    <molecule id="N2">
    <atomArray>
      <atom id="a1" elementType="N" />
      <atom id="a2" elementType="N" />
    </atomArray>
    <bondArray>
      <bond atomRefs2="a2 a1" order="3"/>
    </bondArray>
    <propertyList>
      <property dictRef="me:epsilon">
        <scalar>48.0</scalar>
      </property>
      <property dictRef="me:sigma">
        <scalar>3.90</scalar>
      </property>
      <property dictRef="me:MW">
        <scalar units="amu">28.0</scalar>
      </property>
    </propertyList>
  </molecule>
    <molecule id="Heptanal-15-rad" spinMultiplicity="2">
      <atomArray>
        <atom id="a1" elementType="C" x3="1.588095" y3="-0.316037" z3="0.082686" />
        <atom id="a2" elementType="O" x3="2.103867" y3="-0.071660" z3="1.143228" />
        <atom id="a3" elementType="C" x3="1.021068" y3="-1.643842" z3="-0.360744" />
        <atom id="a4" elementType="H" x3="0.834896" y3="-1.617487" z3="-1.433902" />
        <atom id="a5" elementType="H" x3="1.794355" y3="-2.386888" z3="-0.154928" />
        <atom id="a6" elementType="C" spinMultiplicity="2" x3="-0.228449" y3="-1.951661"
z3="0.399878" />
        <atom id="a7" elementType="C" x3="-1.572468" y3="-1.743859" z3="-0.201633" />
```

```
  <atom id="a8" elementType="H" x3="-1.662540" y3="-2.350527" z3="-1.110782" />
  <atom id="a9" elementType="H" x3="-2.337374" y3="-2.099961" z3="0.490401" />
  <atom id="a10" elementType="C" x3="-1.871687" y3="-0.280953" z3="-0.578749" />
  <atom id="a11" elementType="H" x3="-2.850524" y3="-0.240189" z3="-1.063133" />
  <atom id="a12" elementType="H" x3="-1.147296" y3="0.057860" z3="-1.323743" />
  <atom id="a13" elementType="O" x3="1.419267" y3="0.628468" z3="-0.856285" />
  <atom id="a14" elementType="H" x3="-0.124508" y3="-2.163775" z3="1.454595" />
  <atom id="a15" elementType="C" x3="-1.851428" y3="0.672587" z3="0.609593" />
  <atom id="a16" elementType="H" x3="-2.609337" y3="0.360287" z3="1.333596" />
  <atom id="a17" elementType="H" x3="-0.891084" y3="0.594445" z3="1.126464" />
  <atom id="a18" elementType="C" x3="-2.086204" y3="2.119993" z3="0.200852" />
  <atom id="a19" elementType="H" x3="-2.085769" y3="2.782400" z3="1.066276" />
  <atom id="a20" elementType="H" x3="-1.304159" y3="2.459716" z3="-0.479672" />
  <atom id="a21" elementType="H" x3="-3.045694" y3="2.233816" z3="-0.307098" />
  <atom id="a22" elementType="O" x3="1.879602" y3="1.889765" z3="-0.410688" />
  <atom id="a23" elementType="H" x3="2.213572" y3="1.660351" z3="0.480583" />
</atomArray>
<bondArray>
  <bond atomRefs2="a4 a3" order="1" />
  <bond atomRefs2="a12 a10" order="1" />
  <bond atomRefs2="a8 a7" order="1" />
  <bond atomRefs2="a11 a10" order="1" />
  <bond atomRefs2="a13 a22" order="1" />
  <bond atomRefs2="a13 a1" order="1" />
  <bond atomRefs2="a10 a7" order="1" />
  <bond atomRefs2="a10 a15" order="1" />
  <bond atomRefs2="a20 a18" order="1" />
  <bond atomRefs2="a22 a23" order="1" />
  <bond atomRefs2="a3 a5" order="1" />
  <bond atomRefs2="a3 a1" order="1" />
  <bond atomRefs2="a3 a6" order="1" />
  <bond atomRefs2="a21 a18" order="1" />
  <bond atomRefs2="a7 a6" order="1" />
  <bond atomRefs2="a7 a9" order="1" />
  <bond atomRefs2="a1 a2" order="2" />
  <bond atomRefs2="a18 a15" order="1" />
  <bond atomRefs2="a18 a19" order="1" />
  <bond atomRefs2="a6 a14" order="1" />
  <bond atomRefs2="a15 a17" order="1" />
  <bond atomRefs2="a15 a16" order="1" />
</bondArray>
<propertyList>
  <property title="program">
    <scalar>Gaussian 16, Revision C.01</scalar>
  </property>
  <property title="basis">
```

<scalar>Aug-CC-pVTZ (5D, 7F)</scalar>
</property>
<property title="File Format">
<scalar>g03</scalar>
</property>
<property title="Energy" dictRef="me:ZPE">
<scalar units="kcal/mol">2.8</scalar>
</property>
<property title="SpinMultiplicity" dictRef="me:spinMultiplicity">
<scalar>2.00</scalar>
</property>
<property title="Vibrational Frequencies" dictRef="me:vibFreqs">
<array units="cm-1">28.15 40.23 61.23 91.69 136.57 179.15 226.58 253.02 262.91 294.63 330.03 398.13 424.62 439.52 487.33 561.27 644.72 747.16 783.50 822.81 846.91 901.07 924.04 952.63 986.50 1012.21 1040.14 1068.59 1115.12 1117.26 1157.97 1185.33 1248.53 1263.27 1282.45 1326.26 1337.08 1341.48 1364.51 1404.04 1421.07 1423.07 1471.07 1486.44 1496.67 1503.14 1507.21 1515.43 1526.40 1817.45 3004.54 3034.19 3042.33 3045.82 3059.82 3062.46 3080.45 3094.87 3113.98 3122.10 3125.40 3206.66 3555.56</array>
</property>
<property title="Rotational Constants" dictRef="me:rotConsts">
<array units="cm-1">0.049 0.035 0.024</array>
</property>
<property title="Symmetry Number" dictRef="me:symmetryNumber">
<scalar>1</scalar>
</property>
</propertyList>
</molecule>
<molecule id="15Hshift-TS" spinMultiplicity="2">
<atomArray>
<atom id="a1" elementType="C" x3="2.097102" y3="-0.103401" z3="-0.269627" />
<atom id="a2" elementType="O" x3="2.917083" y3="-0.133561" z3="-1.131743" />
<atom id="a3" elementType="C" x3="1.609131" y3="1.126078" z3="0.470941" />
<atom id="a4" elementType="H" x3="2.133213" y3="1.165617" z3="1.426575" />
<atom id="a5" elementType="H" x3="1.918989" y3="1.982907" z3="-0.126811" />
<atom id="a6" elementType="C" spinMultiplicity="2" x3="0.117428" y3="1.080569" z3="0.693222" />
<atom id="a7" elementType="C" x3="-0.760005" y3="1.234877" z3="-0.518285" />
<atom id="a8" elementType="H" x3="-0.569276" y3="2.228682" z3="-0.941302" />
<atom id="a9" elementType="H" x3="-0.448441" y3="0.525625" z3="-1.290088" />
<atom id="a10" elementType="C" x3="-2.253203" y3="1.091097" z3="-0.233700" />
<atom id="a11" elementType="H" x3="-2.799831" y3="1.300471" z3="-1.156190" />
<atom id="a12" elementType="H" x3="-2.551938" y3="1.861192" z3="0.482681" />
<atom id="a13" elementType="O" x3="1.455528" y3="-1.272291" z3="0.053624" />
<atom id="a14" elementType="O" x3="0.700972" y3="-1.211079" z3="1.221847" />
<atom id="a15" elementType="H" x3="-0.222454" y3="1.620182" z3="1.575641" />
<atom id="a16" elementType="H" x3="0.072896" y3="-0.145531" z3="1.076872" />

<atom id="a17" elementType="C" x3="-2.676966" y3="-0.279288" z3="0.294972" />
<atom id="a18" elementType="H" x3="-2.209040" y3="-0.464741" z3="1.265316" />
<atom id="a19" elementType="H" x3="-3.752074" y3="-0.252528" z3="0.482382" />
<atom id="a20" elementType="C" x3="-2.355694" y3="-1.431521" z3="-0.649222" />
<atom id="a21" elementType="H" x3="-2.767247" y3="-2.367234" z3="-0.272780" />
<atom id="a22" elementType="H" x3="-2.779018" y3="-1.253680" z3="-1.639870" />
<atom id="a23" elementType="H" x3="-1.281207" y3="-1.575983" z3="-0.762059" />
</atomArray>
<bondArray>
<bond atomRefs2="a22 a20" order="1" />
<bond atomRefs2="a9 a7" order="1" />
<bond atomRefs2="a11 a10" order="1" />
<bond atomRefs2="a2 a1" order="2" />
<bond atomRefs2="a8 a7" order="1" />
<bond atomRefs2="a23 a20" order="1" />
<bond atomRefs2="a20 a21" order="1" />
<bond atomRefs2="a20 a17" order="1" />
<bond atomRefs2="a7 a10" order="1" />
<bond atomRefs2="a7 a6" order="1" />
<bond atomRefs2="a1 a13" order="1" />
<bond atomRefs2="a1 a3" order="1" />
<bond atomRefs2="a10 a17" order="1" />
<bond atomRefs2="a10 a12" order="1" />
<bond atomRefs2="a5 a3" order="1" />
<bond atomRefs2="a13 a14" order="1" />
<bond atomRefs2="a17 a19" order="1" />
<bond atomRefs2="a17 a18" order="1" />
<bond atomRefs2="a3 a6" order="1" />
<bond atomRefs2="a3 a4" order="1" />
<bond atomRefs2="a6 a15" order="1" />
<bond atomRefs2="a16 a14" order="1" />
</bondArray>
<propertyList>
<property title="program">
<scalar>Gaussian 16, Revision C.01</scalar>
</property>
<property title="basis">
<scalar>Aug-CC-pVTZ (5D, 7F)</scalar>
</property>
<property title="File Format">
<scalar>g03</scalar>
</property>
<property title="Energy" dictRef="me:ZPE">
<scalar units="kcal/mol">20.9</scalar>
</property>
<property title="SpinMultiplicity" dictRef="me:spinMultiplicity">

<scalar>2.00</scalar>
</property>
<property title="Vibrational Frequencies" dictRef="me:vibFreqs">
<array units="cm-1">37.51 76.63 86.02 116.55 170.50 222.22 252.98 290.50 304.74 383.03 465.86 482.86 505.97 565.46 590.46 707.64 744.26 775.77 811.85 851.56 873.05 912.53 983.49 1003.58 1035.86 1059.92 1066.17 1110.82 1128.68 1147.55 1180.62 1222.45 1230.49 1252.85 1278.72 1314.97 1325.44 1342.05 1385.03 1398.34 1424.16 1429.23 1459.40 1475.18 1496.21 1499.42 1509.42 1518.76 1583.56 1875.09 3010.41 3038.04 3045.60 3047.31 3063.72 3076.64 3078.10 3088.19 3118.13 3118.48 3129.99 3131.64</array>
</property>
<property title="ImaginaryFrequency" dictRef="me:imFreqs">
<scalar units="cm-1">1706.73</scalar>
</property>
<property title="Rotational Constants" dictRef="me:rotConsts">
<array units="cm-1">0.069 0.027 0.024</array>
</property>
<property title="Symmetry Number" dictRef="me:symmetryNumber">
<scalar>1</scalar>
</property>
<property dictRef="me:frequenciesScaleFactor" >
<scalar>1</scalar>
</property>
</propertyList>
<me:DOSCMethod name="ClassicalRotors" />
</molecule>
<molecule id="Heptanal-16-rad" spinMultiplicity="2">
<atomArray>
<atom id="a1" elementType="C" x3="1.400142" y3="-0.426462" z3="0.283287" />
<atom id="a2" elementType="O" x3="1.279884" y3="0.110404" z3="1.356053" />
<atom id="a3" elementType="C" x3="0.915597" y3="-1.795318" z3="-0.091255" />
<atom id="a4" elementType="H" x3="1.163079" y3="-1.998385" z3="-1.131895" />
<atom id="a5" elementType="H" x3="1.456624" y3="-2.507146" z3="0.533891" />
<atom id="a6" elementType="C" x3="-0.596221" y3="-1.920481" z3="0.147158" />
<atom id="a7" elementType="H" x3="-0.808530" y3="-1.709580" z3="1.197049" />
<atom id="a8" elementType="H" x3="-0.864343" y3="-2.971785" z3="-0.016724" />
<atom id="a9" elementType="C" spinMultiplicity="2" x3="-1.401680" y3="-1.029273" z3="-0.731449" />
<atom id="a10" elementType="C" x3="-2.528407" y3="-0.217384" z3="-0.199984" />
<atom id="a11" elementType="H" x3="-3.144695" y3="-0.836526" z3="0.461840" />
<atom id="a12" elementType="H" x3="-3.172798" y3="0.108224" z3="-1.019335" />
<atom id="a13" elementType="C" x3="-2.071698" y3="1.021632" z3="0.595456" />
<atom id="a14" elementType="H" x3="-2.949627" y3="1.480624" z3="1.055026" />
<atom id="a15" elementType="H" x3="-1.421096" y3="0.702866" z3="1.412716" />
<atom id="a16" elementType="O" x3="2.008025" y3="0.183827" z3="-0.745735" />
<atom id="a17" elementType="H" x3="-1.133444" y3="-0.966401" z3="-1.778900" />
<atom id="a18" elementType="C" x3="-1.345133" y3="2.046728" z3="-0.264078" />

<atom id="a19" elementType="H" x3="-1.033531" y3="2.906793" z3="0.328206" />
<atom id="a20" elementType="H" x3="-0.452449" y3="1.620938" z3="-0.724266" />
<atom id="a21" elementType="H" x3="-1.988416" y3="2.408902" z3="-1.068492" />
<atom id="a22" elementType="O" x3="2.457310" y3="1.475477" z3="-0.388373" />
<atom id="a23" elementType="H" x3="2.151873" y3="1.527151" z3="0.540517" />
</atomArray>
<bondArray>
<bond atomRefs2="a17 a9" order="1" />
<bond atomRefs2="a4 a3" order="1" />
<bond atomRefs2="a21 a18" order="1" />
<bond atomRefs2="a12 a10" order="1" />
<bond atomRefs2="a16 a22" order="1" />
<bond atomRefs2="a16 a1" order="1" />
<bond atomRefs2="a9 a10" order="1" />
<bond atomRefs2="a9 a6" order="1" />
<bond atomRefs2="a20 a18" order="1" />
<bond atomRefs2="a22 a23" order="1" />
<bond atomRefs2="a18 a19" order="1" />
<bond atomRefs2="a18 a13" order="1" />
<bond atomRefs2="a10 a11" order="1" />
<bond atomRefs2="a10 a13" order="1" />
<bond atomRefs2="a3 a6" order="1" />
<bond atomRefs2="a3 a1" order="1" />
<bond atomRefs2="a3 a5" order="1" />
<bond atomRefs2="a8 a6" order="1" />
<bond atomRefs2="a6 a7" order="1" />
<bond atomRefs2="a1 a2" order="2" />
<bond atomRefs2="a13 a14" order="1" />
<bond atomRefs2="a13 a15" order="1" />
</bondArray>
<propertyList>
<property title="program">
<scalar>Gaussian 16, Revision C.01</scalar>
</property>
<property title="basis">
<scalar>Aug-CC-pVTZ (5D, 7F)</scalar>
</property>
<property title="File Format">
<scalar>g03</scalar>
</property>
<property title="Energy" dictRef="me:ZPE">
<scalar units="kcal/mol">1.92</scalar>
</property>
<property title="SpinMultiplicity" dictRef="me:spinMultiplicity">
<scalar>2.00</scalar>
</property>

<property title="Vibrational Frequencies" dictRef="me:vibFreqs">
<array units="cm-1">38.09 46.17 73.72 93.59 154.55 181.61 217.83 239.30 274.65 327.38 341.40 373.98 430.68 463.05 495.18 510.14 633.46 725.15 770.22 848.92 857.53 895.95 914.87 960.62 992.59 1024.87 1041.42 1055.47 1090.40 1128.29 1149.60 1196.96 1216.44 1257.61 1287.41 1293.09 1336.16 1359.90 1380.57 1393.51 1423.07 1433.83 1477.51 1485.19 1488.62 1501.23 1503.49 1512.42 1520.30 1820.03 2995.99 3010.05 3044.34 3049.93 3073.96 3077.43 3083.67 3089.89 3115.66 3125.45 3135.36 3179.95 3552.92</array>
</property>
<property title="Rotational Constants" dictRef="me:rotConsts">
<array units="cm-1">0.055 0.033 0.025</array>
</property>
<property title="Symmetry Number" dictRef="me:symmetryNumber">
<scalar>1</scalar>
</property>
</propertyList>
</molecule>
<molecule id="16Hshift-TS" spinMultiplicity="2">
<atomArray>
<atom id="a1" elementType="C" x3="1.967194" y3="-0.567126" z3="-0.311747" />
<atom id="a2" elementType="O" x3="2.459941" y3="-1.118706" z3="-1.247724" />
<atom id="a3" elementType="C" x3="2.103559" y3="0.892298" z3="0.029879" />
<atom id="a4" elementType="H" x3="2.336496" y3="1.005691" z3="1.086455" />
<atom id="a5" elementType="H" x3="2.932027" y3="1.269857" z3="-0.562937" />
<atom id="a6" elementType="C" x3="0.819486" y3="1.679725" z3="-0.315253" />
<atom id="a7" elementType="H" x3="0.668079" y3="1.623108" z3="-1.395332" />
<atom id="a8" elementType="H" x3="1.005652" y3="2.725515" z3="-0.072293" />
<atom id="a9" elementType="C" spinMultiplicity="2" x3="-0.436421" y3="1.192664" z3="0.389703" />
<atom id="a10" elementType="C" x3="-1.479547" y3="0.520503" z3="-0.461517" />
<atom id="a11" elementType="H" x3="-1.006672" y3="-0.267379" z3="-1.056652" />
<atom id="a12" elementType="H" x3="-1.853003" y3="1.256210" z3="-1.186393" />
<atom id="a13" elementType="C" x3="-2.645743" y3="-0.058866" z3="0.328935" />
<atom id="a14" elementType="H" x3="-2.259797" y3="-0.791473" z3="1.041024" />
<atom id="a15" elementType="H" x3="-3.109325" y3="0.735361" z3="0.920234" />
<atom id="a16" elementType="O" x3="1.125796" y3="-1.316950" z3="0.477116" />
<atom id="a17" elementType="O" x3="0.688051" y3="-0.658531" z3="1.617368" />
<atom id="a18" elementType="H" x3="-0.844636" y3="1.892063" z3="1.119067" />
<atom id="a19" elementType="H" x3="-0.014302" y3="0.257461" z3="1.162901" />
<atom id="a20" elementType="C" x3="-3.690306" y3="-0.711396" z3="-0.565884" />
<atom id="a21" elementType="H" x3="-4.108506" y3="0.008388" z3="-1.271755" />
<atom id="a22" elementType="H" x3="-3.253181" y3="-1.527379" z3="-1.143212" />
<atom id="a23" elementType="H" x3="-4.512466" y3="-1.120745" z3="0.020114" />
</atomArray>
<bondArray>
<bond atomRefs2="a7 a6" order="1" />
<bond atomRefs2="a21 a20" order="1" />

<bond atomRefs2="a2 a1" order="2" />
<bond atomRefs2="a12 a10" order="1" />
<bond atomRefs2="a22 a20" order="1" />
<bond atomRefs2="a11 a10" order="1" />
<bond atomRefs2="a20 a23" order="1" />
<bond atomRefs2="a20 a13" order="1" />
<bond atomRefs2="a5 a3" order="1" />
<bond atomRefs2="a10 a13" order="1" />
<bond atomRefs2="a10 a9" order="1" />
<bond atomRefs2="a6 a8" order="1" />
<bond atomRefs2="a6 a3" order="1" />
<bond atomRefs2="a6 a9" order="1" />
<bond atomRefs2="a1 a3" order="1" />
<bond atomRefs2="a1 a16" order="1" />
<bond atomRefs2="a3 a4" order="1" />
<bond atomRefs2="a13 a15" order="1" />
<bond atomRefs2="a13 a14" order="1" />
<bond atomRefs2="a9 a18" order="1" />
<bond atomRefs2="a16 a17" order="1" />
<bond atomRefs2="a19 a17" order="1" />
</bondArray>
<propertyList>
<property title="program">
<scalar>Gaussian 16, Revision C.01</scalar>
</property>
<property title="basis">
<scalar>Aug-CC-pVTZ (5D, 7F)</scalar>
</property>
<property title="File Format">
<scalar>g03</scalar>
</property>
<property title="Energy" dictRef="me:ZPE">
<scalar units="kcal/mol">19.02</scalar>
</property>
<property title="SpinMultiplicity" dictRef="me:spinMultiplicity">
<scalar>2.00</scalar>
</property>
<property title="Vibrational Frequencies" dictRef="me:vibFreqs">
<array units="cm-1">9.87 74.53 78.48 129.25 146.88 207.69 243.00 269.61 356.73 384.49 419.34 452.13 508.92 578.66 606.64 731.75 740.10 773.86 829.91 886.85 900.54 919.22 982.67 1001.10 1048.59 1074.64 1088.28 1106.21 1119.96 1138.40 1167.89 1182.20 1230.69 1270.09 1291.51 1297.01 1341.63 1349.18 1374.45 1386.87 1421.32 1424.36 1467.55 1478.08 1482.27 1499.78 1501.16 1506.71 1513.02 1872.31 2996.71 3040.96 3044.87 3050.80 3055.65 3084.15 3096.01 3103.50 3116.10 3117.02 3126.12 3163.28</array>
</property>
<property title="ImaginaryFrequency" dictRef="me:imFreqs">

```
<scalar units="cm-1">1736.78</scalar>
</property>
<property title="Rotational Constants" dictRef="me:rotConsts">
<array units="cm-1">0.068 0.025 0.023</array>
</property>
<property title="Symmetry Number" dictRef="me:symmetryNumber">
<scalar>1</scalar>
</property>
<property dictRef="me:frequenciesScaleFactor" >
<scalar>1</scalar>
</property>
</propertyList>
<me:DOSCMethod name="ClassicalRotors" />
</molecule>
<molecule id="Heptanal-17-rad" spinMultiplicity="2">
<atomArray>
<atom id="a1" elementType="C" x3="-0.505553" y3="2.092378" z3="0.404841" />
<atom id="a2" elementType="H" x3="-0.709270" y3="1.714766" z3="1.407591" />
<atom id="a3" elementType="H" x3="-0.608479" y3="3.177257" z3="0.445941" />
<atom id="a4" elementType="C" x3="-1.531649" y3="1.527171" z3="-0.594309" />
<atom id="a5" elementType="H" x3="-1.362962" y3="1.994132" z3="-1.566939" />
<atom id="a6" elementType="C" spinMultiplicity="2" x3="-1.497091" y3="0.045332" z3="-
0.729459" />
<atom id="a7" elementType="H" x3="-1.109688" y3="-0.398526" z3="-1.637729" />
<atom id="a8" elementType="C" x3="0.937919" y3="1.755676" z3="0.032101" />
<atom id="a9" elementType="H" x3="1.142298" y3="1.992437" z3="-1.011539" />
<atom id="a10" elementType="H" x3="1.625636" y3="2.339470" z3="0.646921" />
<atom id="a11" elementType="C" x3="1.275658" y3="0.317208" z3="0.295051" />
<atom id="a12" elementType="O" x3="1.104461" y3="-0.285763" z3="1.326171" />
<atom id="a13" elementType="C" x3="-2.025745" y3="-0.844751" z3="0.336181" />
<atom id="a14" elementType="H" x3="-1.629077" y3="-0.531509" z3="1.309071" />
<atom id="a15" elementType="H" x3="-3.114496" y3="-0.712048" z3="0.415591" />
<atom id="a16" elementType="O" x3="1.870781" y3="-0.255578" z3="-0.763819" />
<atom id="a17" elementType="H" x3="-2.525101" y3="1.847286" z3="-0.258549" />
<atom id="a18" elementType="O" x3="2.216179" y3="-1.599406" z3="-0.493969" />
<atom id="a19" elementType="C" x3="-1.703617" y3="-2.316270" z3="0.109461" />
<atom id="a20" elementType="H" x3="-2.106763" y3="-2.937642" z3="0.908381" />
<atom id="a21" elementType="H" x3="-2.123435" y3="-2.665972" z3="-0.834789" />
<atom id="a22" elementType="H" x3="-0.624956" y3="-2.471093" z3="0.071451" />
<atom id="a23" elementType="H" x3="1.915390" y3="-1.683038" z3="0.434331" />
</atomArray>
<bondArray>
<bond atomRefs2="a7 a6" order="1" />
<bond atomRefs2="a5 a4" order="1" />
<bond atomRefs2="a9 a8" order="1" />
<bond atomRefs2="a21 a19" order="1" />
```

<bond atomRefs2="a16 a18" order="1" />
<bond atomRefs2="a16 a11" order="1" />
<bond atomRefs2="a6 a4" order="1" />
<bond atomRefs2="a6 a13" order="1" />
<bond atomRefs2="a4 a17" order="1" />
<bond atomRefs2="a4 a1" order="1" />
<bond atomRefs2="a18 a23" order="1" />
<bond atomRefs2="a8 a11" order="1" />
<bond atomRefs2="a8 a1" order="1" />
<bond atomRefs2="a8 a10" order="1" />
<bond atomRefs2="a22 a19" order="1" />
<bond atomRefs2="a19 a13" order="1" />
<bond atomRefs2="a19 a20" order="1" />
<bond atomRefs2="a11 a12" order="2" />
<bond atomRefs2="a13 a15" order="1" />
<bond atomRefs2="a13 a14" order="1" />
<bond atomRefs2="a1 a3" order="1" />
<bond atomRefs2="a1 a2" order="1" />
</bondArray>
<propertyList>
<property title="program">
<scalar>Gaussian 16, Revision C.01</scalar>
</property>
<property title="basis">
<scalar>Aug-CC-pVTZ (5D, 7F)</scalar>
</property>
<property title="File Format">
<scalar>g03</scalar>
</property>
<property title="Energy" dictRef="me:ZPE">
<scalar units="kcal/mol">1.39</scalar>
</property>
<property title="SpinMultiplicity" dictRef="me:spinMultiplicity">
<scalar>2.00</scalar>
</property>
<property title="Vibrational Frequencies" dictRef="me:vibFreqs">
<array units="cm-1">43.98 57.44 82.77 104.19 116.92 191.92 233.72 237.86 255.14 295.73
341.50 366.91 427.40 445.09 494.03 533.19 621.37 724.59 778.32 813.33 867.93 907.73 937.46 964.05
1008.61 1034.93 1052.92 1062.97 1069.94 1121.97 1158.24 1195.99 1226.31 1253.48 1281.15 1297.27
1332.01 1364.24 1383.81 1403.08 1422.96 1434.99 1471.28 1477.33 1488.58 1497.54 1504.28 1508.83
1534.04 1812.87 2971.43 3007.28 3021.07 3049.62 3065.74 3071.25 3077.03 3112.37 3119.78 3127.27
3128.64 3186.04 3555.41</array>
</property>
<property title="Rotational Constants" dictRef="me:rotConsts">
<array units="cm-1">0.047 0.039 0.026</array>
</property>

<property title="Symmetry Number" dictRef="me:symmetryNumber">
<scalar>1</scalar>
</property>
</propertyList>
</molecule>
<molecule id="17Hshift-TS" spinMultiplicity="2">
<atomArray>
<atom id="a1" elementType="C" x3="-0.886238" y3="1.684260" z3="-0.541200" />
<atom id="a2" elementType="H" x3="-0.670244" y3="1.348605" z3="-1.556919" />
<atom id="a3" elementType="H" x3="-1.430574" y3="2.622786" z3="-0.645933" />
<atom id="a4" elementType="C" x3="0.408478" y3="1.987624" z3="0.215396" />
<atom id="a5" elementType="H" x3="0.159085" y3="2.412069" z3="1.190441" />
<atom id="a6" elementType="C" spinMultiplicity="2" x3="1.362578" y3="0.835266" z3="0.417379" />
<atom id="a7" elementType="H" x3="2.180502" y3="1.082279" z3="1.096080" />
<atom id="a8" elementType="C" x3="-1.838183" y3="0.677361" z3="0.134307" />
<atom id="a9" elementType="H" x3="-1.857965" y3="0.833943" z3="1.210061" />
<atom id="a10" elementType="H" x3="-2.839398" y3="0.825908" z3="-0.263862" />
<atom id="a11" elementType="C" x3="-1.514374" y3="-0.757302" z3="-0.198155" />
<atom id="a12" elementType="O" x3="-1.935581" y3="-1.345718" z3="-1.148369" />
<atom id="a13" elementType="C" x3="1.815716" y3="0.048938" z3="-0.784173" />
<atom id="a14" elementType="H" x3="0.956133" y3="-0.320159" z3="-1.347309" />
<atom id="a15" elementType="H" x3="2.336744" y3="0.740688" z3="-1.458358" />
<atom id="a16" elementType="O" x3="-0.646297" y3="-1.454990" z3="0.606697" />
<atom id="a17" elementType="H" x3="0.941790" y3="2.775268" z3="-0.329819" />
<atom id="a18" elementType="O" x3="-0.144486" y3="-0.728674" z3="1.673610" />
<atom id="a19" elementType="H" x3="0.704061" y3="0.001674" z3="1.146216" />
<atom id="a20" elementType="C" x3="2.727758" y3="-1.116726" z3="-0.426042" />
<atom id="a21" elementType="H" x3="3.614336" y3="-0.771201" z3="0.107824" />
<atom id="a22" elementType="H" x3="2.205190" y3="-1.829769" z3="0.211397" />
<atom id="a23" elementType="H" x3="3.056834" y3="-1.643573" z3="-1.320394" />
</atomArray>
<bondArray>
<bond atomRefs2="a2 a1" order="1" />
<bond atomRefs2="a15 a13" order="1" />
<bond atomRefs2="a14 a13" order="1" />
<bond atomRefs2="a23 a20" order="1" />
<bond atomRefs2="a12 a11" order="2" />
<bond atomRefs2="a13 a20" order="1" />
<bond atomRefs2="a13 a6" order="1" />
<bond atomRefs2="a3 a1" order="1" />
<bond atomRefs2="a1 a8" order="1" />
<bond atomRefs2="a1 a4" order="1" />
<bond atomRefs2="a20 a21" order="1" />
<bond atomRefs2="a20 a22" order="1" />
<bond atomRefs2="a17 a4" order="1" />

```
<bond atomRefs2="a10 a8" order="1" />
<bond atomRefs2="a11 a8" order="1" />
<bond atomRefs2="a11 a16" order="1" />
<bond atomRefs2="a8 a9" order="1" />
<bond atomRefs2="a4 a6" order="1" />
<bond atomRefs2="a4 a5" order="1" />
<bond atomRefs2="a6 a7" order="1" />
<bond atomRefs2="a16 a18" order="1" />
<bond atomRefs2="a19 a18" order="1" />
</bondArray>
<propertyList>
<property title="program">
<scalar>Gaussian 16, Revision C.01</scalar>
</property>
<property title="basis">
<scalar>Aug-CC-pVTZ (5D, 7F)</scalar>
</property>
<property title="File Format">
<scalar>g03</scalar>
</property>
<property title="Energy" dictRef="me:ZPE">
<scalar units="kcal/mol">17.39</scalar>
</property>
<property title="SpinMultiplicity" dictRef="me:spinMultiplicity">
<scalar>2.00</scalar>
</property>
<property title="Vibrational Frequencies" dictRef="me:vibFreqs">
<array units="cm-1">57.46 83.78 117.24 161.07 198.74 239.05 252.64 288.15 333.26 379.96
422.45 506.55 527.17 571.03 604.99 711.39 761.45 769.03 831.65 834.21 911.91 925.36 968.86
1010.05 1035.05 1063.20 1088.48 1098.01 1120.43 1137.92 1172.88 1206.98 1231.31 1263.60 1297.98
1319.00 1327.05 1371.87 1389.07 1402.87 1417.98 1427.17 1451.95 1475.85 1481.41 1490.09 1506.40
1508.14 1511.10 1860.87 3004.08 3015.60 3051.29 3064.64 3075.48 3078.26 3093.28 3097.77 3112.07
3124.38 3136.01 3157.47</array>
</property>
<property title="ImaginaryFrequency" dictRef="me:imFreqs">
<scalar units="cm-1">1710.89</scalar>
</property>
<property title="Rotational Constants" dictRef="me:rotConsts">
<array units="cm-1">0.052 0.037 0.029</array>
</property>
<property title="Symmetry Number" dictRef="me:symmetryNumber">
<scalar>1</scalar>
</property>
<property dictRef="me:frequenciesScaleFactor" >
<scalar>1</scalar>
</property>
```

</propertyList>
<me:DOSCMethod name="ClassicalRotors" />
</molecule>
<molecule id="Heptanal-18-rad" spinMultiplicity="2">
<atomArray>
<atom id="a1" elementType="C" x3="1.627806" y3="-0.112568" z3="-0.347978" />
<atom id="a2" elementType="O" x3="1.333762" y3="0.952418" z3="-0.831211" />
<atom id="a3" elementType="C" x3="0.938678" y3="-1.427084" z3="-0.575230" />
<atom id="a4" elementType="H" x3="1.300567" y3="-1.798707" z3="-1.536434" />
<atom id="a5" elementType="H" x3="1.259161" y3="-2.135742" z3="0.187935" />
<atom id="a6" elementType="C" x3="-0.579025" y3="-1.272984" z3="-0.602054" />
<atom id="a7" elementType="H" x3="-1.017990" y3="-2.221160" z3="-0.918225" />
<atom id="a8" elementType="H" x3="-0.847703" y3="-0.533545" z3="-1.357843" />
<atom id="a9" elementType="C" x3="-1.159471" y3="-0.864602" z3="0.746503" />
<atom id="a10" elementType="C" x3="-2.673099" y3="-0.619203" z3="0.690250" />
<atom id="a11" elementType="H" x3="-3.012476" y3="-0.406088" z3="1.714182" />
<atom id="a12" elementType="H" x3="-3.178321" y3="-1.539605" z3="0.389556" />
<atom id="a13" elementType="C" spinMultiplicity="2" x3="-3.071615" y3="0.498634" z3="-
0.210617" />
<atom id="a14" elementType="H" x3="-3.880334" y3="0.351398" z3="-0.912052" />
<atom id="a15" elementType="O" x3="2.675041" y3="-0.243642" z3="0.480432" />
<atom id="a16" elementType="H" x3="-0.663937" y3="0.044349" z3="1.098282" />
<atom id="a17" elementType="H" x3="-0.946381" y3="-1.638648" z3="1.487823" />
<atom id="a18" elementType="C" x3="-2.417436" y3="1.828938" z3="-0.115381" />
<atom id="a19" elementType="H" x3="-2.940797" y3="2.579400" z3="-0.705084" />
<atom id="a20" elementType="H" x3="-2.377567" y3="2.181201" z3="0.922305" />
<atom id="a21" elementType="H" x3="-1.377479" y3="1.803029" z3="-0.464801" />
<atom id="a22" elementType="O" x3="3.351511" y3="0.984661" z3="0.660094" />
<atom id="a23" elementType="H" x3="2.805727" y3="1.579836" z3="0.106868" />
</atomArray>
<bondArray>
<bond atomRefs2="a4 a3" order="1" />
<bond atomRefs2="a8 a6" order="1" />
<bond atomRefs2="a7 a6" order="1" />
<bond atomRefs2="a14 a13" order="1" />
<bond atomRefs2="a2 a1" order="2" />
<bond atomRefs2="a19 a18" order="1" />
<bond atomRefs2="a6 a3" order="1" />
<bond atomRefs2="a6 a9" order="1" />
<bond atomRefs2="a3 a1" order="1" />
<bond atomRefs2="a3 a5" order="1" />
<bond atomRefs2="a21 a18" order="1" />
<bond atomRefs2="a1 a15" order="1" />
<bond atomRefs2="a13 a18" order="1" />
<bond atomRefs2="a13 a10" order="1" />
<bond atomRefs2="a18 a20" order="1" />

<bond atomRefs2="a23 a22" order="1" />
<bond atomRefs2="a12 a10" order="1" />
<bond atomRefs2="a15 a22" order="1" />
<bond atomRefs2="a10 a9" order="1" />
<bond atomRefs2="a10 a11" order="1" />
<bond atomRefs2="a9 a16" order="1" />
<bond atomRefs2="a9 a17" order="1" />
</bondArray>
<propertyList>
<property title="program">
<scalar>Gaussian 16, Revision C.01</scalar>
</property>
<property title="basis">
<scalar>Aug-CC-pVTZ (5D, 7F)</scalar>
</property>
<property title="File Format">
<scalar>g03</scalar>
</property>
<property title="Energy" dictRef="me:ZPE">
<scalar units="kcal/mol">1.48</scalar>
</property>
<property title="SpinMultiplicity" dictRef="me:spinMultiplicity">
<scalar>2.00</scalar>
</property>
<property title="Vibrational Frequencies" dictRef="me:vibFreqs">
<array units="cm-1">22.50 34.05 53.02 79.81 125.46 137.71 210.97 217.99 251.99 289.37 322.41 344.03 408.21 459.99 489.46 567.48 625.33 700.89 791.60 830.12 864.06 906.65 953.32 987.72 991.86 1009.11 1029.01 1069.52 1083.75 1133.48 1161.48 1177.89 1219.11 1251.98 1281.08 1327.45 1343.43 1376.93 1388.17 1405.01 1417.39 1429.07 1473.48 1476.38 1482.54 1489.77 1496.31 1509.70 1516.11 1816.93 2970.47 2991.86 3028.92 3042.50 3060.35 3066.01 3069.35 3082.03 3107.74 3123.97 3124.84 3203.82 3559.94</array>
</property>
<property title="Rotational Constants" dictRef="me:rotConsts">
<array units="cm-1">0.075 0.022 0.019</array>
</property>
<property title="Symmetry Number" dictRef="me:symmetryNumber">
<scalar>1</scalar>
</property>
</propertyList>
</molecule>
<molecule id="18Hshift-TS" spinMultiplicity="2">
<atomArray>
<atom id="a1" elementType="C" x3="1.398882" y3="-0.872465" z3="-0.243383" />
<atom id="a2" elementType="O" x3="1.899178" y3="-1.189442" z3="-1.280546" />
<atom id="a3" elementType="C" x3="1.825737" y3="0.307921" z3="0.589062" />
<atom id="a4" elementType="H" x3="1.482556" y3="0.214879" z3="1.614719" />

<atom id="a5" elementType="H" x3="2.914162" y3="0.267870" z3="0.576109" />
<atom id="a6" elementType="C" x3="1.386371" y3="1.652786" z3="-0.010339" />
<atom id="a7" elementType="H" x3="1.640697" y3="1.665593" z3="-1.072432" />
<atom id="a8" elementType="H" x3="1.993965" y3="2.425666" z3="0.462133" />
<atom id="a9" elementType="C" x3="-0.084901" y3="2.023588" z3="0.176126" />
<atom id="a10" elementType="C" x3="-1.090974" y3="1.161392" z3="-0.607865" />
<atom id="a11" elementType="H" x3="-0.577991" y3="0.554200" z3="-1.359062" />
<atom id="a12" elementType="H" x3="-1.760993" y3="1.811027" z3="-1.182454" />
<atom id="a13" elementType="C" spinMultiplicity="2" x3="-1.962848" y3="0.272745" z3="0.240952" />
<atom id="a14" elementType="H" x3="-1.148290" y3="-0.473134" z3="0.896984" />
<atom id="a15" elementType="H" x3="-2.452092" y3="0.804435" z3="1.058771" />
<atom id="a16" elementType="O" x3="0.363873" y3="-1.669538" z3="0.191797" />
<atom id="a17" elementType="O" x3="-0.265281" y3="-1.221908" z3="1.340662" />
<atom id="a18" elementType="H" x3="-0.197255" y3="3.066185" z3="-0.122769" />
<atom id="a19" elementType="H" x3="-0.328387" y3="1.988286" z3="1.241342" />
<atom id="a20" elementType="C" x3="-2.854138" y3="-0.691714" z3="-0.489450" />
<atom id="a21" elementType="H" x3="-2.270132" y3="-1.334607" z3="-1.149083" />
<atom id="a22" elementType="H" x3="-3.414030" y3="-1.321706" z3="0.199281" />
<atom id="a23" elementType="H" x3="-3.573150" y3="-0.147108" z3="-1.109462" />
</atomArray>
<bondArray>
<bond atomRefs2="a11 a10" order="1" />
<bond atomRefs2="a2 a1" order="2" />
<bond atomRefs2="a12 a10" order="1" />
<bond atomRefs2="a21 a20" order="1" />
<bond atomRefs2="a23 a20" order="1" />
<bond atomRefs2="a7 a6" order="1" />
<bond atomRefs2="a10 a9" order="1" />
<bond atomRefs2="a10 a13" order="1" />
<bond atomRefs2="a20 a22" order="1" />
<bond atomRefs2="a20 a13" order="1" />
<bond atomRefs2="a1 a16" order="1" />
<bond atomRefs2="a1 a3" order="1" />
<bond atomRefs2="a18 a9" order="1" />
<bond atomRefs2="a6 a9" order="1" />
<bond atomRefs2="a6 a8" order="1" />
<bond atomRefs2="a6 a3" order="1" />
<bond atomRefs2="a9 a19" order="1" />
<bond atomRefs2="a16 a17" order="1" />
<bond atomRefs2="a13 a15" order="1" />
<bond atomRefs2="a5 a3" order="1" />
<bond atomRefs2="a3 a4" order="1" />
<bond atomRefs2="a14 a17" order="1" />
</bondArray>
<propertyList>

```
<property title="program">
  <scalar>Gaussian 16, Revision C.01</scalar>
</property>
<property title="basis">
  <scalar>Aug-CC-pVTZ (5D, 7F)</scalar>
</property>
<property title="File Format">
  <scalar>g03</scalar>
</property>
<property title="Energy" dictRef="me:ZPE">
  <scalar units="kcal/mol">18.73</scalar>
</property>
<property title="SpinMultiplicity" dictRef="me:spinMultiplicity">
  <scalar>2.00</scalar>
</property>
<property title="Vibrational Frequencies" dictRef="me:vibFreqs">
  <array units="cm-1">59.61 84.33 121.23 141.13 196.52 235.02 241.44 284.80 366.18 383.12
416.94 465.90 521.82 555.56 592.22 694.97 770.74 779.10 859.33 880.79 896.13 916.28 957.29
1021.70 1049.68 1083.30 1096.10 1108.44 1123.23 1133.26 1159.97 1173.07 1237.40 1265.28 1286.53
1304.37 1332.81 1382.58 1394.10 1398.04 1410.38 1419.06 1447.77 1482.75 1490.22 1493.52 1497.55
1500.59 1503.21 1862.01 3015.57 3029.54 3044.88 3055.63 3061.72 3087.63 3092.94 3096.20 3102.03
3104.55 3140.11 3168.22</array>
</property>
<property title="ImaginaryFrequency" dictRef="me:imFreqs">
  <scalar units="cm-1">1706.70</scalar>
</property>
<property title="Rotational Constants" dictRef="me:rotConsts">
  <array units="cm-1">0.053 0.038 0.028</array>
</property>
<property title="Symmetry Number" dictRef="me:symmetryNumber">
  <scalar>1</scalar>
</property>
<property dictRef="me:frequenciesScaleFactor" >
  <scalar>1</scalar>
</property>
</propertyList>
<me:DOSCMethod  name="ClassicalRotors" />
</molecule>
<molecule id="Heptanal-AcylOO-rad" spinMultiplicity="2">
 <atomArray>
  <atom id="a1" elementType="C" x3="-5.062371" y3="0.050865" z3="0.000000" />
  <atom id="a2" elementType="H" x3="-5.151208" y3="0.689354" z3="0.880655" />
  <atom id="a3" elementType="H" x3="-5.151208" y3="0.689354" z3="-0.880655" />
  <atom id="a4" elementType="H" x3="-5.907645" y3="-0.637094" z3="0.000000" />
  <atom id="a5" elementType="C" x3="-3.735382" y3="-0.696169" z3="0.000000" />
  <atom id="a6" elementType="H" x3="-3.682864" y3="-1.350598" z3="0.874416" />
```

<atom id="a7" elementType="H" x3="-3.682864" y3="-1.350598" z3="-0.874416" />
<atom id="a8" elementType="C" x3="-2.530562" y3="0.236638" z3="0.000000" />
<atom id="a9" elementType="H" x3="-2.581000" y3="0.892464" z3="0.874776" />
<atom id="a10" elementType="H" x3="-2.581000" y3="0.892464" z3="-0.874776" />
<atom id="a11" elementType="C" x3="-1.197997" y3="-0.500841" z3="0.000000" />
<atom id="a12" elementType="H" x3="-1.147391" y3="-1.156323" z3="0.875138" />
<atom id="a13" elementType="H" x3="-1.147391" y3="-1.156323" z3="-0.875138" />
<atom id="a14" elementType="C" x3="0.000000" y3="0.439943" z3="0.000000" />
<atom id="a15" elementType="H" x3="-0.042127" y3="1.094579" z3="0.872477" />
<atom id="a16" elementType="H" x3="-0.042127" y3="1.094579" z3="-0.872477" />
<atom id="a17" elementType="C" x3="1.318263" y3="-0.319829" z3="0.000000" />
<atom id="a18" elementType="H" x3="1.401511" y3="-0.979124" z3="-0.866354" />
<atom id="a19" elementType="H" x3="1.401511" y3="-0.979124" z3="0.866354" />
<atom id="a20" elementType="C" x3="2.497676" y3="0.598299" z3="0.000000" />
<atom id="a21" elementType="O" x3="2.520857" y3="1.780599" z3="0.000000" />
<atom id="a22" elementType="O" x3="3.789367" y3="-0.025147" z3="0.000000" />
<atom id="a23" elementType="O" spinMultiplicity="2" x3="3.761781" y3="-1.330084" z3="0.000000" />
</atomArray>
<bondArray>
<bond atomRefs2="a3 a1" order="1" />
<bond atomRefs2="a13 a11" order="1" />
<bond atomRefs2="a10 a8" order="1" />
<bond atomRefs2="a7 a5" order="1" />
<bond atomRefs2="a16 a14" order="1" />
<bond atomRefs2="a18 a17" order="1" />
<bond atomRefs2="a1 a4" order="1" />
<bond atomRefs2="a1 a5" order="1" />
<bond atomRefs2="a1 a2" order="1" />
<bond atomRefs2="a5 a8" order="1" />
<bond atomRefs2="a5 a6" order="1" />
<bond atomRefs2="a8 a11" order="1" />
<bond atomRefs2="a8 a9" order="1" />
<bond atomRefs2="a11 a14" order="1" />
<bond atomRefs2="a11 a12" order="1" />
<bond atomRefs2="a14 a17" order="1" />
<bond atomRefs2="a14 a15" order="1" />
<bond atomRefs2="a17 a20" order="1" />
<bond atomRefs2="a17 a19" order="1" />
<bond atomRefs2="a20 a21" order="2" />
<bond atomRefs2="a20 a22" order="1" />
<bond atomRefs2="a22 a23" order="1" />
</bondArray>
<propertyList>
<property title="program">
<scalar>Gaussian 16, Revision C.01</scalar>

</property>
<property title="basis">
<scalar>Aug-CC-pVTZ (5D, 7F)</scalar>
</property>
<property title="File Format">
<scalar>g03</scalar>
</property>
<property title="Energy" dictRef="me:ZPE">
<scalar units="kcal/mol">0.0</scalar>
</property>
<property title="SpinMultiplicity" dictRef="me:spinMultiplicity">
<scalar>2.00</scalar>
</property>
<property title="Vibrational Frequencies" dictRef="me:vibFreqs">
<array units="cm-1">34.81 52.45 69.38 102.55 130.14 139.34 160.94 176.41 226.40 245.18 318.97 416.94 465.33 529.95 563.74 609.24 733.32 749.85 809.39 826.79 901.65 913.07 1008.09 1033.20 1044.90 1077.91 1081.10 1091.06 1142.24 1152.73 1228.63 1249.61 1268.85 1284.78 1330.16 1330.38 1344.56 1350.82 1385.00 1419.33 1421.75 1430.53 1452.60 1493.85 1494.33 1501.19 1505.86 1510.29 1517.58 1921.65 3021.62 3026.30 3036.74 3043.01 3047.06 3058.68 3061.10 3073.10 3079.61 3095.97 3113.10 3115.12 3120.91</array>
</property>
<property title="Rotational Constants" dictRef="me:rotConsts">
<array units="cm-1">0.142 0.012 0.012</array>
</property>
<property title="Symmetry Number" dictRef="me:symmetryNumber">
<scalar>1</scalar>
</property>
<property dictRef="me:sigma" >
<scalar>7.34</scalar>
</property>
<property dictRef="me:epsilon" >
<scalar>362.0</scalar>
</property>
<property dictRef="me:frequenciesScaleFactor" >
<scalar>1</scalar>
</property>
</propertyList>
<me:DOSCMethod name="ClassicalRotors" />
<me:DistributionCalcMethod name="Boltzmann" />
<me:energyTransferModel name="ExponentialDown" />
<me:deltaEDown >225.0</me:deltaEDown>
</molecule>
<molecule id="Heptanal-14-rad" spinMultiplicity="2">
<atomArray>
<atom id="a1" elementType="C" x3="-1.545960" y3="0.182322" z3="0.184131" />
<atom id="a2" elementType="O" x3="-1.348860" y3="-0.579183" z3="1.112099" />

<atom id="a3" elementType="C" spinMultiplicity="2" x3="-0.789758" y3="1.351831" z3="-0.162022" />
<atom id="a4" elementType="O" x3="-2.573204" y3="-0.016510" z3="-0.677727" />
<atom id="a5" elementType="O" x3="-3.305471" y3="-1.169026" z3="-0.330374" />
<atom id="a6" elementType="H" x3="-1.095819" y3="1.902634" z3="-1.041640" />
<atom id="a7" elementType="H" x3="-2.814199" y3="-1.468661" z3="0.463073" />
<atom id="a8" elementType="C" x3="0.405335" y3="1.767306" z3="0.602301" />
<atom id="a9" elementType="H" x3="0.282731" y3="2.811658" z3="0.905110" />
<atom id="a10" elementType="H" x3="0.484206" y3="1.171060" z3="1.509820" />
<atom id="a11" elementType="C" x3="1.699447" y3="1.661054" z3="-0.228873" />
<atom id="a12" elementType="H" x3="1.623928" y3="2.333703" z3="-1.086307" />
<atom id="a13" elementType="H" x3="2.523900" y3="2.029476" z3="0.385774" />
<atom id="a14" elementType="C" x3="2.015423" y3="0.250171" z3="-0.718510" />
<atom id="a15" elementType="H" x3="1.203419" y3="-0.104596" z3="-1.361945" />
<atom id="a16" elementType="H" x3="2.903965" y3="0.296052" z3="-1.353642" />
<atom id="a17" elementType="C" x3="2.251710" y3="-0.766200" z3="0.393930" />
<atom id="a18" elementType="H" x3="3.036497" y3="-0.392365" z3="1.058167" />
<atom id="a19" elementType="H" x3="1.350628" y3="-0.867723" z3="1.001423" />
<atom id="a20" elementType="C" x3="2.645051" y3="-2.134862" z3="-0.145700" />
<atom id="a21" elementType="H" x3="1.862156" y3="-2.537274" z3="-0.790854" />
<atom id="a22" elementType="H" x3="3.562640" y3="-2.077801" z3="-0.734204" />
<atom id="a23" elementType="H" x3="2.808734" y3="-2.848146" z3="0.661707" />
</atomArray>
<bondArray>
<bond atomRefs2="a15 a14" order="1" />
<bond atomRefs2="a16 a14" order="1" />
<bond atomRefs2="a12 a11" order="1" />
<bond atomRefs2="a6 a3" order="1" />
<bond atomRefs2="a21 a20" order="1" />
<bond atomRefs2="a22 a20" order="1" />
<bond atomRefs2="a14 a11" order="1" />
<bond atomRefs2="a14 a17" order="1" />
<bond atomRefs2="a4 a5" order="1" />
<bond atomRefs2="a4 a1" order="1" />
<bond atomRefs2="a5 a7" order="1" />
<bond atomRefs2="a11 a13" order="1" />
<bond atomRefs2="a11 a8" order="1" />
<bond atomRefs2="a3 a1" order="1" />
<bond atomRefs2="a3 a8" order="1" />
<bond atomRefs2="a20 a17" order="1" />
<bond atomRefs2="a20 a23" order="1" />
<bond atomRefs2="a1 a2" order="2" />
<bond atomRefs2="a17 a19" order="1" />
<bond atomRefs2="a17 a18" order="1" />
<bond atomRefs2="a8 a9" order="1" />
<bond atomRefs2="a8 a10" order="1" />

</bondArray>
<propertyList>
<property title="program">
<scalar>Gaussian 16, Revision C.01</scalar>
</property>
<property title="basis">
<scalar>Aug-CC-pVTZ (5D, 7F)</scalar>
</property>
<property title="File Format">
<scalar>g03</scalar>
</property>
<property title="Energy" dictRef="me:ZPE">
<scalar units="kcal/mol" >-2.23</scalar>
</property>
<property title="SpinMultiplicity" dictRef="me:spinMultiplicity">
<scalar>2.00</scalar>
</property>
<property title="Vibrational Frequencies" dictRef="me:vibFreqs">
<array units="cm-1">29.04 36.53 79.44 90.44 144.08 181.22 236.42 244.17 261.93 307.12 371.11 394.98 456.95 498.75 503.02 624.94 711.69 745.50 769.04 805.45 850.20 890.95 930.75 956.75 1001.84 1038.05 1049.47 1088.29 1107.59 1122.65 1160.41 1192.65 1237.36 1250.23 1302.68 1331.23 1346.60 1364.70 1389.76 1418.56 1422.44 1463.79 1489.13 1495.39 1500.62 1503.98 1506.79 1515.29 1532.28 1747.53 3023.63 3034.33 3038.25 3041.78 3046.29 3065.31 3085.29 3090.45 3113.39 3122.44 3126.68 3204.03 3531.90</array>
</property>
<property title="Rotational Constants" dictRef="me:rotConsts">
<array units="cm-1">0.059 0.024 0.019</array>
</property>
<property title="Symmetry Number" dictRef="me:symmetryNumber">
<scalar>1</scalar>
</property>
</propertyList>
</molecule>
<molecule id="14Hshift-TS" spinMultiplicity="2">
<atomArray>
<atom id="a1" elementType="C" x3="-1.857010" y3="-0.194584" z3="0.506138" />
<atom id="a2" elementType="O" x3="-2.117997" y3="-0.054679" z3="1.657296" />
<atom id="a3" elementType="C" x3="-1.521076" y3="0.876387" z3="-0.499837" />
<atom id="a4" elementType="O" x3="-1.824996" y3="-1.417395" z3="-0.082151" />
<atom id="a5" elementType="O" spinMultiplicity="2" x3="-1.435004" y3="-1.269287" z3="-1.424347" />
<atom id="a6" elementType="H" x3="-2.422328" y3="1.350240" z3="-0.885742" />
<atom id="a7" elementType="H" x3="-1.246887" y3="-0.005594" z3="-1.376014" />
<atom id="a8" elementType="C" x3="-0.416613" y3="1.838030" z3="-0.153037" />
<atom id="a9" elementType="H" x3="-0.228089" y3="2.474355" z3="-1.020653" />
<atom id="a10" elementType="H" x3="-0.810462" y3="2.498276" z3="0.627237" />

```
  <atom id="a11" elementType="C" x3="0.881413" y3="1.198027" z3="0.331648" />
  <atom id="a12" elementType="H" x3="1.536692" y3="1.993213" z3="0.694960" />
  <atom id="a13" elementType="H" x3="0.665400" y3="0.565805" z3="1.195519" />
  <atom id="a14" elementType="C" x3="1.616810" y3="0.399712" z3="-0.741282" />
  <atom id="a15" elementType="H" x3="0.996573" y3="-0.436084" z3="-1.075583" />
  <atom id="a16" elementType="H" x3="1.769344" y3="1.041783" z3="-1.613795" />
  <atom id="a17" elementType="C" x3="2.966285" y3="-0.147143" z3="-0.280505" />
  <atom id="a18" elementType="H" x3="3.469835" y3="-0.596163" z3="-1.138812" />
  <atom id="a19" elementType="H" x3="3.599762" y3="0.682157" z3="0.047046" />
  <atom id="a20" elementType="C" x3="2.861571" y3="-1.183096" z3="0.833471" />
  <atom id="a21" elementType="H" x3="3.838663" y3="-1.604831" z3="1.068055" />
  <atom id="a22" elementType="H" x3="2.462817" y3="-0.752776" z3="1.752403" />
  <atom id="a23" elementType="H" x3="2.204369" y3="-2.003491" z3="0.539430" />
 </atomArray>
 <bondArray>
  <bond atomRefs2="a16 a14" order="1" />
  <bond atomRefs2="a5 a4" order="1" />
  <bond atomRefs2="a7 a3" order="1" />
  <bond atomRefs2="a18 a17" order="1" />
  <bond atomRefs2="a15 a14" order="1" />
  <bond atomRefs2="a9 a8" order="1" />
  <bond atomRefs2="a6 a3" order="1" />
  <bond atomRefs2="a14 a17" order="1" />
  <bond atomRefs2="a14 a11" order="1" />
  <bond atomRefs2="a3 a8" order="1" />
  <bond atomRefs2="a3 a1" order="1" />
  <bond atomRefs2="a17 a19" order="1" />
  <bond atomRefs2="a17 a20" order="1" />
  <bond atomRefs2="a8 a11" order="1" />
  <bond atomRefs2="a8 a10" order="1" />
  <bond atomRefs2="a4 a1" order="1" />
  <bond atomRefs2="a11 a12" order="1" />
  <bond atomRefs2="a11 a13" order="1" />
  <bond atomRefs2="a1 a2" order="2" />
  <bond atomRefs2="a23 a20" order="1" />
  <bond atomRefs2="a20 a21" order="1" />
  <bond atomRefs2="a20 a22" order="1" />
 </bondArray>
 <propertyList>
  <property title="program">
   <scalar>Gaussian 16, Revision C.01</scalar>
  </property>
  <property title="basis">
   <scalar>Aug-CC-pVTZ (5D, 7F)</scalar>
  </property>
  <property title="File Format">
```

```
<scalar>g03</scalar>
</property>
<property title="Energy" dictRef="me:ZPE">
<scalar units="kcal/mol" >27.03</scalar>
</property>
<property title="SpinMultiplicity" dictRef="me:spinMultiplicity">
<scalar>2.00</scalar>
</property>
<property title="Vibrational Frequencies" dictRef="me:vibFreqs">
<array units="cm-1">25.87 32.38 77.55 118.99 148.64 173.32 202.75 232.76 293.63 318.10
411.63 490.44 519.79 542.63 645.51 691.88 739.00 778.13 803.11 856.91 890.30 925.08 956.59
1003.80 1017.64 1024.16 1086.33 1111.04 1129.95 1145.18 1175.99 1182.54 1224.52 1263.24 1312.45
1323.83 1349.36 1360.65 1389.95 1392.36 1422.89 1428.23 1463.31 1494.46 1497.91 1509.67 1512.14
1515.52 1767.72 1912.86 3028.11 3031.72 3038.82 3047.05 3051.02 3070.87 3076.70 3081.73 3099.89
3118.78 3124.40 3127.89</array>
</property>
<property title="ImaginaryFrequency" dictRef="me:imFreqs">
<scalar units="cm-1">1645.41</scalar>
</property>
<property title="Rotational Constants" dictRef="me:rotConsts">
<array units="cm-1">0.059 0.026 0.024</array>
</property>
<property title="Symmetry Number" dictRef="me:symmetryNumber">
<scalar>1</scalar>
</property>
<property dictRef="me:frequenciesScaleFactor" >
<scalar>1</scalar>
</property>
</propertyList>
<me:DOSCMethod name="ClassicalRotors" />
</molecule>
<molecule id="Heptanal-19-rad" spinMultiplicity="2">
<atomArray>
<atom id="a1" elementType="C" x3="1.721753" y3="-0.200771" z3="-0.044727" />
<atom id="a2" elementType="O" x3="2.366226" y3="-0.174395" z3="0.973173" />
<atom id="a3" elementType="C" x3="1.089480" y3="-1.402927" z3="-0.679303" />
<atom id="a4" elementType="H" x3="0.676354" y3="-1.127035" z3="-1.647217" />
<atom id="a5" elementType="H" x3="1.883643" y3="-2.130818" z3="-0.844914" />
<atom id="a6" elementType="C" x3="0.017264" y3="-2.003379" z3="0.239851" />
<atom id="a7" elementType="H" x3="0.504183" y3="-2.367162" z3="1.145058" />
<atom id="a8" elementType="H" x3="-0.406549" y3="-2.872987" z3="-0.266457" />
<atom id="a9" elementType="C" x3="-1.094896" y3="-1.029643" z3="0.620398" />
<atom id="a10" elementType="C" x3="-1.916521" y3="-0.514139" z3="-0.556497" />
<atom id="a11" elementType="H" x3="-2.370859" y3="-1.360245" z3="-1.078869" />
<atom id="a12" elementType="H" x3="-1.267208" y3="-0.011828" z3="-1.278145" />
<atom id="a13" elementType="C" x3="-3.005922" y3="0.469004" z3="-0.128840" />
```

<atom id="a14" elementType="H" x3="-3.646404" y3="0.681229" z3="-0.997278" />
<atom id="a15" elementType="H" x3="-3.667857" y3="-0.006564" z3="0.602802" />
<atom id="a16" elementType="O" x3="1.462917" y3="0.917844" z3="-0.743160" />
<atom id="a17" elementType="O" x3="2.025620" y3="2.056894" z3="-0.123666" />
<atom id="a18" elementType="H" x3="-0.669300" y3="-0.180646" z3="1.163544" />
<atom id="a19" elementType="H" x3="-1.763212" y3="-1.527717" z3="1.327054" />
<atom id="a20" elementType="C" spinMultiplicity="2" x3="-2.473062" y3="1.741507" z3="0.419369" />
<atom id="a21" elementType="H" x3="2.451077" y3="1.650651" z3="0.659144" />
<atom id="a22" elementType="H" x3="-1.499938" y3="2.105395" z3="0.116418" />
<atom id="a23" elementType="H" x3="-3.090608" y3="2.387079" z3="1.026575" />
</atomArray>
<bondArray>
<bond atomRefs2="a4 a3" order="1" />
<bond atomRefs2="a12 a10" order="1" />
<bond atomRefs2="a11 a10" order="1" />
<bond atomRefs2="a14 a13" order="1" />
<bond atomRefs2="a5 a3" order="1" />
<bond atomRefs2="a16 a17" order="1" />
<bond atomRefs2="a16 a1" order="1" />
<bond atomRefs2="a3 a1" order="1" />
<bond atomRefs2="a3 a6" order="1" />
<bond atomRefs2="a10 a13" order="1" />
<bond atomRefs2="a10 a9" order="1" />
<bond atomRefs2="a8 a6" order="1" />
<bond atomRefs2="a13 a20" order="1" />
<bond atomRefs2="a13 a15" order="1" />
<bond atomRefs2="a17 a21" order="1" />
<bond atomRefs2="a1 a2" order="2" />
<bond atomRefs2="a22 a20" order="1" />
<bond atomRefs2="a6 a9" order="1" />
<bond atomRefs2="a6 a7" order="1" />
<bond atomRefs2="a20 a23" order="1" />
<bond atomRefs2="a9 a18" order="1" />
<bond atomRefs2="a9 a19" order="1" />
</bondArray>
<propertyList>
<property title="program">
<scalar>Gaussian 16, Revision C.01</scalar>
</property>
<property title="basis">
<scalar>Aug-CC-pVTZ (5D, 7F)</scalar>
</property>
<property title="File Format">
<scalar>g03</scalar>
</property>

```
      <property title="Energy" dictRef="me:ZPE">
        <scalar units="kcal/mol" >4.71</scalar>
      </property>
      <property title="SpinMultiplicity" dictRef="me:spinMultiplicity">
        <scalar>2.00</scalar>
      </property>
      <property title="Vibrational Frequencies" dictRef="me:vibFreqs">
        <array units="cm-1">30.35 38.39 56.71 122.32 162.56 173.66 178.73 237.89 269.54 326.96
347.81 412.29 441.60 485.41 501.52 514.40 643.26 727.11 765.95 820.31 858.22 894.65 908.98 940.59
981.39 993.90 1034.18 1079.55 1091.31 1112.67 1135.29 1160.84 1211.79 1225.56 1283.96 1303.25
1337.14 1347.90 1380.00 1393.05 1394.90 1420.30 1470.52 1472.62 1488.69 1496.35 1498.90 1508.58
1514.10 1822.26 2965.90 3030.46 3036.19 3044.13 3056.77 3070.49 3088.82 3091.67 3108.00 3145.03
3153.16 3258.90 3555.44</array>
      </property>
      <property title="Rotational Constants" dictRef="me:rotConsts">
        <array units="cm-1">0.056 0.028 0.021</array>
      </property>
      <property title="Symmetry Number" dictRef="me:symmetryNumber">
        <scalar>1</scalar>
      </property>
    </propertyList>
  </molecule>
  <molecule id="19Hshift-TS" spinMultiplicity="2">
    <atomArray>
      <atom id="a1" elementType="C" x3="0.817524" y3="-1.240710" z3="0.221320" />
      <atom id="a2" elementType="O" x3="0.656480" y3="-1.786720" z3="1.266642" />
      <atom id="a3" elementType="C" x3="2.004163" y3="-0.404602" z3="-0.192202" />
      <atom id="a4" elementType="H" x3="2.640715" y3="-0.325233" z3="0.687642" />
      <atom id="a5" elementType="H" x3="2.555003" y3="-0.968717" z3="-0.947663" />
      <atom id="a6" elementType="C" x3="1.659426" y3="0.980153" z3="-0.755148" />
      <atom id="a7" elementType="H" x3="1.049317" y3="0.859247" z3="-1.650076" />
      <atom id="a8" elementType="H" x3="2.593664" y3="1.434944" z3="-1.087992" />
      <atom id="a9" elementType="C" x3="0.987265" y3="1.945182" z3="0.227001" />
      <atom id="a10" elementType="C" x3="-0.375069" y3="1.516966" z3="0.778036" />
      <atom id="a11" elementType="H" x3="-0.694286" y3="2.253411" z3="1.517973" />
      <atom id="a12" elementType="H" x3="-0.270024" y3="0.586289" z3="1.336009" />
      <atom id="a13" elementType="C" x3="-1.477991" y3="1.360168" z3="-0.276042" />
      <atom id="a14" elementType="H" x3="-1.972655" y3="2.324345" z3="-0.440546" />
      <atom id="a15" elementType="H" x3="-1.052678" y3="1.089029" z3="-1.244604" />
      <atom id="a16" elementType="O" x3="-0.086357" y3="-1.271957" z3="-0.813109" />
      <atom id="a17" elementType="O" x3="-1.305829" y3="-1.812996" z3="-0.449534" />
      <atom id="a18" elementType="H" x3="0.882373" y3="2.912093" z3="-0.272411" />
      <atom id="a19" elementType="H" x3="1.661610" y3="2.110466" z3="1.071168" />
      <atom id="a20" elementType="C" spinMultiplicity="2" x3="-2.515503" y3="0.337809"
z3="0.089331" />
      <atom id="a21" elementType="H" x3="-1.945986" y3="-0.831396" z3="-0.123053" />
```

<atom id="a22" elementType="H" x3="-3.374471" y3="0.276257" z3="-0.573859" />
<atom id="a23" elementType="H" x3="-2.785818" y3="0.282842" z3="1.141643" />
</atomArray>
<bondArray>
<bond atomRefs2="a7 a6" order="1" />
<bond atomRefs2="a15 a13" order="1" />
<bond atomRefs2="a8 a6" order="1" />
<bond atomRefs2="a5 a3" order="1" />
<bond atomRefs2="a16 a17" order="1" />
<bond atomRefs2="a16 a1" order="1" />
<bond atomRefs2="a6 a3" order="1" />
<bond atomRefs2="a6 a9" order="1" />
<bond atomRefs2="a22 a20" order="1" />
<bond atomRefs2="a17 a21" order="1" />
<bond atomRefs2="a14 a13" order="1" />
<bond atomRefs2="a13 a20" order="1" />
<bond atomRefs2="a13 a10" order="1" />
<bond atomRefs2="a18 a9" order="1" />
<bond atomRefs2="a3 a1" order="1" />
<bond atomRefs2="a3 a4" order="1" />
<bond atomRefs2="a20 a23" order="1" />
<bond atomRefs2="a1 a2" order="2" />
<bond atomRefs2="a9 a10" order="1" />
<bond atomRefs2="a9 a19" order="1" />
<bond atomRefs2="a10 a12" order="1" />
<bond atomRefs2="a10 a11" order="1" />
</bondArray>
<propertyList>
<property title="program">
<scalar>Gaussian 16, Revision C.01</scalar>
</property>
<property title="basis">
<scalar>Aug-CC-pVTZ (5D, 7F)</scalar>
</property>
<property title="File Format">
<scalar>g03</scalar>
</property>
<property title="Energy" dictRef="me:ZPE">
<scalar units="kcal/mol" >20.85</scalar>
</property>
<property title="SpinMultiplicity" dictRef="me:spinMultiplicity">
<scalar>2.00</scalar>
</property>
<property title="Vibrational Frequencies" dictRef="me:vibFreqs">
<array units="cm-1">61.14 90.08 171.93 183.32 235.10 275.36 293.34 327.49 330.97 397.20 445.08 479.17 515.67 560.36 618.60 668.74 738.65 796.98 840.44 875.57 887.55 931.26 965.10

```
1002.82 1045.50 1063.94 1074.31 1105.24 1122.19 1134.69 1140.72 1183.90 1216.79 1244.82 1266.04
1302.91 1326.48 1348.60 1374.14 1395.86 1406.79 1419.43 1453.79 1471.31 1472.33 1480.84 1493.91
1503.31 1521.34 1874.72 3017.37 3036.31 3060.12 3065.72 3072.01 3075.77 3081.26 3095.36 3107.28
3109.82 3132.44 3180.78</array>
        </property>
        <property title="ImaginaryFrequency" dictRef="me:imFreqs">
          <scalar units="cm-1">1867.51</scalar>
        </property>
        <property title="Rotational Constants" dictRef="me:rotConsts">
          <array units="cm-1">0.047 0.046 0.029</array>
        </property>
        <property title="Symmetry Number" dictRef="me:symmetryNumber">
          <scalar>1</scalar>
        </property>
        <property dictRef="me:frequenciesScaleFactor" >
          <scalar>1</scalar>
        </property>
      </propertyList>
      <me:DOSCMethod name="ClassicalRotors" />
    </molecule>
  </moleculeList>
  <reactionList>
  <reaction id="R1" >
          <!--reversible="true"-->
      <reactantList>
        <reactant>
          <molecule ref="Heptanal-AcylOO-rad" role="modelled" />
        </reactant>
      </reactantList>
      <productList>
        <product>
          <molecule ref="Heptanal-14-rad" role="sink" />
        </product>
      </productList>
      <me:transitionState>
        <molecule ref="14Hshift-TS" role="transitionState" />
      </me:transitionState>
      <me:MCRCMethod name="RRKM" />
            <me:tunneling name="Eckart" />
    </reaction>
    <reaction id="R2" >
          <!--reversible="true"-->
      <reactantList>
        <reactant>
          <molecule ref="Heptanal-AcylOO-rad" role="modelled" />
        </reactant>
```

```
  </reactantList>
  <productList>
    <product>
      <molecule ref="Heptanal-15-rad" role="sink" />
    </product>
  </productList>
  <me:transitionState>
    <molecule ref="15Hshift-TS" role="transitionState" />
  </me:transitionState>
  <me:MCRCMethod name="RRKM" />
        <me:tunneling name="Eckart" />
</reaction>
<reaction id="R3" >
      <!--reversible="true"-->
  <reactantList>
    <reactant>
      <molecule ref="Heptanal-AcylOO-rad" role="modelled" />
    </reactant>
  </reactantList>
  <productList>
    <product>
      <molecule ref="Heptanal-16-rad" role="sink" />
    </product>
  </productList>
  <me:transitionState>
    <molecule ref="16Hshift-TS" role="transitionState" />
  </me:transitionState>
  <me:MCRCMethod name="RRKM" />
        <me:tunneling name="Eckart" />
</reaction>
<reaction id="R4" >
      <!--reversible="true"-->
  <reactantList>
    <reactant>
      <molecule ref="Heptanal-AcylOO-rad" role="modelled" />
    </reactant>
  </reactantList>
  <productList>
    <product>
      <molecule ref="Heptanal-17-rad" role="sink" />
    </product>
  </productList>
  <me:transitionState>
    <molecule ref="17Hshift-TS" role="transitionState" />
  </me:transitionState>
  <me:MCRCMethod name="RRKM" />
```

<me:tunneling name="Eckart" />
</reaction>
<reaction id="R5" >
<!--reversible="true"-->
<reactantList>
<reactant>
<molecule ref="Heptanal-AcylOO-rad" role="modelled" />
</reactant>
</reactantList>
<productList>
<product>
<molecule ref="Heptanal-18-rad" role="sink" />
</product>
</productList>
<me:transitionState>
<molecule ref="18Hshift-TS" role="transitionState" />
</me:transitionState>
<me:MCRCMethod name="RRKM" />
<me:tunneling name="Eckart" />
</reaction>
<reaction id="R6" >
<!--reversible="true"-->
<reactantList>
<reactant>
<molecule ref="Heptanal-AcylOO-rad" role="modelled" />
</reactant>
</reactantList>
<productList>
<product>
<molecule ref="Heptanal-19-rad" role="sink" />
</product>
</productList>
<me:transitionState>
<molecule ref="19Hshift-TS" role="transitionState" />
</me:transitionState>
<me:MCRCMethod name="RRKM" />
<me:tunneling name="Eckart" />
</reaction>
</reactionList>
<me:conditions>
<me:bathGas>N2</me:bathGas>
<me:PTs>
<me:PTpair units="Torr" P="760." T="298." precision="qd" bathGas="N2" />
</me:PTs>
</me:conditions>
<me:modelParameters>

```
    <!--Specify grain size directly...-->
    <me:grainSize units="cm-1">100</me:grainSize>
    <!--...or by the total number of grains
        <me:numberOfGrains> 500 </me:numberOfGrains>-->
    <!--Specify increased energy range
        <me:maxTemperature>6000</me:maxTemperature>-->
    <me:energyAboveTheTopHill>25.0</me:energyAboveTheTopHill>
  </me:modelParameters>
  <me:control>
    <me:printSpeciesProfile />
    <!--<me:testMicroRates />
          <me:testDOS />
    <me:testRateConstant />
    <me:printGrainDOS />-->
    <!--<me:printCellDOS />-->
    <!--<me:printReactionOperatorColumnSums />-->
    <!--<me:printTunnellingCoefficients />
    <me:printGrainkfE />-->
    <!--<me:printGrainBoltzmann />
    <me:printGrainkbE />-->
    <me:eigenvalues>3</me:eigenvalues>
    <!-- <me:hideInactive/>  Molecules and reactions with attribute active="false" are not shown
    <me:diagramEnergyOffset>0</me:diagramEnergyOffset>-->
    <!--Adjusts displayed energies to this values for the lowest species. -->
    <me:calcMethod  name="simpleCalc" />
    <me:ForceMacroDetailedBalance >true</me:ForceMacroDetailedBalance>
  </me:control>
  <metadataList xmlns:dc="http://purl.org/dc/elements/1.1/">
    <dc:title>Project name</dc:title>
    <dc:source>Acylperoxyrad-Heptanal.xml</dc:source>
    <dc:creator>Mesmer v6.0</dc:creator>
    <dc:date>20220118_122459</dc:date>
    <dc:contributor>ndprse</dc:contributor>
  </metadataList>
</me:mesmer>
```


Octanal–AcOO ------> Octanal–AcOOH


```
<?xml version="1.0" encoding="utf-8" ?>
<?xml-stylesheet type='text/xsl' href='../../mesmer2.xsl' media='other'?>
<?xml-stylesheet type='text/xsl' href='../../mesmer1.xsl' media='screen'?>
```

```
<me:mesmer xmlns="http://www.xml-cml.org/schema"
xmlns:me="http://www.chem.leeds.ac.uk/mesmer"
xmlns:xsi="http://www.w3.org/2001/XMLSchema-instance">
  <me:title>Project name</me:title>
  <moleculeList convention="">
    <molecule id="N2">
    <atomArray>
      <atom id="a1" elementType="N" />
      <atom id="a2" elementType="N" />
    </atomArray>
    <bondArray>
      <bond atomRefs2="a2 a1" order="3"/>
    </bondArray>
    <propertyList>
      <property dictRef="me:epsilon">
        <scalar>48.0</scalar>
      </property>
      <property dictRef="me:sigma">
        <scalar>3.90</scalar>
      </property>
      <property dictRef="me:MW">
        <scalar units="amu">28.0</scalar>
      </property>
    </propertyList>
  </molecule>
    <molecule id="Octanal-15-rad" spinMultiplicity="2">
      <atomArray>
        <atom id="a1" elementType="C" x3="1.606257" y3="-0.393545" z3="0.337872" />
        <atom id="a2" elementType="O" x3="1.534757" y3="0.272705" z3="1.338519" />
        <atom id="a3" elementType="C" x3="1.309749" y3="-1.869429" z3="0.202437" />
        <atom id="a4" elementType="H" x3="1.680788" y3="-2.209500" z3="-0.766707" />
        <atom id="a5" elementType="H" x3="1.867369" y3="-2.375448" z3="0.988991" />
        <atom id="a6" elementType="C" spinMultiplicity="2" x3="-0.156269" y3="-2.115503"
z3="0.338751" />
        <atom id="a7" elementType="C" x3="-1.124807" y3="-1.752926" z3="-0.729183" />
        <atom id="a8" elementType="H" x3="-0.625375" y3="-1.164557" z3="-1.502926" />
        <atom id="a9" elementType="H" x3="-1.475014" y3="-2.663847" z3="-1.232528" />
        <atom id="a10" elementType="C" x3="-2.353299" y3="-1.000884" z3="-0.197305" />
        <atom id="a11" elementType="H" x3="-2.877478" y3="-1.651845" z3="0.507091" />
        <atom id="a12" elementType="H" x3="-3.043254" y3="-0.822684" z3="-1.026049" />
        <atom id="a13" elementType="O" x3="1.938033" y3="0.143119" z3="-0.848062" />
        <atom id="a14" elementType="H" x3="-0.531397" y3="-2.460315" z3="1.293110" />
        <atom id="a15" elementType="C" x3="-2.025993" y3="0.321171" z3="0.493105" />
        <atom id="a16" elementType="H" x3="-2.934454" y3="0.708300" z3="0.962042" />
        <atom id="a17" elementType="H" x3="-1.316870" y3="0.140130" z3="1.306828" />
        <atom id="a18" elementType="C" x3="-1.457083" y3="1.392035" z3="-0.431566" />
```

<atom id="a19" elementType="H" x3="-0.558478" y3="1.024344" z3="-0.930502" />
<atom id="a20" elementType="H" x3="-2.178832" y3="1.601551" z3="-1.226252" />
<atom id="a21" elementType="C" x3="-1.116543" y3="2.677752" z3="0.309585" />
<atom id="a22" elementType="H" x3="-0.693414" y3="3.424120" z3="-0.362419" />
<atom id="a23" elementType="H" x3="-2.003464" y3="3.108967" z3="0.777496" />
<atom id="a24" elementType="H" x3="-0.389427" y3="2.483191" z3="1.100363" />
<atom id="a25" elementType="O" x3="2.153976" y3="1.536962" z3="-0.745043" />
<atom id="a26" elementType="H" x3="1.973098" y3="1.683280" z3="0.205977" />
</atomArray>
<bondArray>
<bond atomRefs2="a8 a7" order="1" />
<bond atomRefs2="a9 a7" order="1" />
<bond atomRefs2="a20 a18" order="1" />
<bond atomRefs2="a12 a10" order="1" />
<bond atomRefs2="a19 a18" order="1" />
<bond atomRefs2="a13 a25" order="1" />
<bond atomRefs2="a13 a1" order="1" />
<bond atomRefs2="a4 a3" order="1" />
<bond atomRefs2="a25 a26" order="1" />
<bond atomRefs2="a7 a10" order="1" />
<bond atomRefs2="a7 a6" order="1" />
<bond atomRefs2="a18 a21" order="1" />
<bond atomRefs2="a18 a15" order="1" />
<bond atomRefs2="a22 a21" order="1" />
<bond atomRefs2="a10 a15" order="1" />
<bond atomRefs2="a10 a11" order="1" />
<bond atomRefs2="a3 a1" order="1" />
<bond atomRefs2="a3 a6" order="1" />
<bond atomRefs2="a3 a5" order="1" />
<bond atomRefs2="a21 a23" order="1" />
<bond atomRefs2="a21 a24" order="1" />
<bond atomRefs2="a1 a2" order="2" />
<bond atomRefs2="a6 a14" order="1" />
<bond atomRefs2="a15 a16" order="1" />
<bond atomRefs2="a15 a17" order="1" />
</bondArray>
<propertyList>
<property title="program">
<scalar>Gaussian 16, Revision C.01</scalar>
</property>
<property title="basis">
<scalar>Aug-CC-pVTZ (5D, 7F)</scalar>
</property>
<property title="File Format">
<scalar>g03</scalar>
</property>

<property title="Energy" dictRef="me:ZPE">
<scalar units="kcal/mol" >2.91</scalar>
</property>
<property title="SpinMultiplicity" dictRef="me:spinMultiplicity">
<scalar>2.00</scalar>
</property>
<property title="Vibrational Frequencies" dictRef="me:vibFreqs">
<array units="cm-1">41.29 56.56 68.58 76.27 145.85 162.58 186.60 227.71 261.14 267.00 294.56 324.54 356.21 418.57 437.39 458.22 482.86 515.70 642.47 738.37 783.55 794.73 840.95 873.93 895.85 930.81 966.34 972.99 1012.44 1048.59 1052.18 1092.73 1116.73 1122.67 1152.78 1182.93 1224.12 1251.29 1299.47 1304.62 1323.07 1330.09 1341.82 1356.90 1392.26 1416.63 1419.47 1426.94 1470.80 1486.15 1492.40 1497.19 1502.89 1506.73 1517.89 1523.99 1817.58 2988.05 3030.69 3039.21 3041.49 3043.07 3058.04 3065.34 3068.84 3084.94 3092.71 3114.24 3122.27 3129.48 3194.81 3561.71</array>
</property>
<property title="Rotational Constants" dictRef="me:rotConsts">
<array units="cm-1">0.038 0.032 0.021</array>
</property>
<property title="Symmetry Number" dictRef="me:symmetryNumber">
<scalar>1</scalar>
</property>
</propertyList>
</molecule>
<molecule id="15Hshift-TS" spinMultiplicity="2">
<atomArray>
<atom id="a1" elementType="C" x3="-2.884537" y3="-0.424980" z3="-0.387313" />
<atom id="a2" elementType="O" x3="-3.465563" y3="-1.279136" z3="-0.978906" />
<atom id="a3" elementType="C" x3="-2.393678" y3="-0.496820" z3="1.045729" />
<atom id="a4" elementType="H" x3="-3.129530" y3="0.008586" z3="1.672331" />
<atom id="a5" elementType="H" x3="-2.387640" y3="-1.554159" z3="1.309815" />
<atom id="a6" elementType="C" spinMultiplicity="2" x3="-1.040297" y3="0.149606" z3="1.201507" />
<atom id="a7" elementType="C" x3="0.135395" y3="-0.537332" z3="0.565487" />
<atom id="a8" elementType="H" x3="0.312017" y3="-1.479269" z3="1.100900" />
<atom id="a9" elementType="H" x3="-0.113965" y3="-0.821005" z3="-0.461390" />
<atom id="a10" elementType="C" x3="1.407308" y3="0.301857" z3="0.573307" />
<atom id="a11" elementType="H" x3="1.649398" y3="0.583799" z3="1.602570" />
<atom id="a12" elementType="H" x3="1.221875" y3="1.234324" z3="0.033009" />
<atom id="a13" elementType="O" x3="-2.543244" y3="0.714806" z3="-1.068724" />
<atom id="a14" elementType="O" x3="-2.098715" y3="1.761652" z3="-0.265501" />
<atom id="a15" elementType="H" x3="-0.848644" y3="0.562108" z3="2.190231" />
<atom id="a16" elementType="H" x3="-1.300677" y3="1.195638" z3="0.500475" />
<atom id="a17" elementType="C" x3="2.596958" y3="-0.416690" z3="-0.048989" />
<atom id="a18" elementType="H" x3="2.781600" y3="-1.350228" z3="0.492186" />
<atom id="a19" elementType="H" x3="2.348972" y3="-0.703362" z3="-1.075597" />
<atom id="a20" elementType="C" x3="3.869087" y3="0.421905" z3="-0.054714" />

<atom id="a21" elementType="H" x3="3.681687" y3="1.352967" z3="-0.596119" />
<atom id="a22" elementType="H" x3="4.114689" y3="0.709241" z3="0.971328" />
<atom id="a23" elementType="C" x3="5.053067" y3="-0.303546" z3="-0.679800" />
<atom id="a24" elementType="H" x3="4.842325" y3="-0.574470" z3="-1.715709" />
<atom id="a25" elementType="H" x3="5.949253" y3="0.316499" z3="-0.672936" />
<atom id="a26" elementType="H" x3="5.278989" y3="-1.223252" z3="-0.137324" />
</atomArray>
<bondArray>
<bond atomRefs2="a24 a23" order="1" />
<bond atomRefs2="a19 a17" order="1" />
<bond atomRefs2="a13 a1" order="1" />
<bond atomRefs2="a13 a14" order="1" />
<bond atomRefs2="a2 a1" order="2" />
<bond atomRefs2="a23 a25" order="1" />
<bond atomRefs2="a23 a26" order="1" />
<bond atomRefs2="a23 a20" order="1" />
<bond atomRefs2="a21 a20" order="1" />
<bond atomRefs2="a9 a7" order="1" />
<bond atomRefs2="a1 a3" order="1" />
<bond atomRefs2="a14 a16" order="1" />
<bond atomRefs2="a20 a17" order="1" />
<bond atomRefs2="a20 a22" order="1" />
<bond atomRefs2="a17 a18" order="1" />
<bond atomRefs2="a17 a10" order="1" />
<bond atomRefs2="a12 a10" order="1" />
<bond atomRefs2="a7 a10" order="1" />
<bond atomRefs2="a7 a8" order="1" />
<bond atomRefs2="a7 a6" order="1" />
<bond atomRefs2="a10 a11" order="1" />
<bond atomRefs2="a3 a6" order="1" />
<bond atomRefs2="a3 a5" order="1" />
<bond atomRefs2="a3 a4" order="1" />
<bond atomRefs2="a6 a15" order="1" />
</bondArray>
<propertyList>
<property title="program">
<scalar>Gaussian 16, Revision C.01</scalar>
</property>
<property title="basis">
<scalar>Aug-CC-pVTZ (5D, 7F)</scalar>
</property>
<property title="File Format">
<scalar>g03</scalar>
</property>
<property title="Energy" dictRef="me:ZPE">
<scalar units="kcal/mol" >20.51</scalar>

</property>
<property title="SpinMultiplicity" dictRef="me:spinMultiplicity">
<scalar>2.00</scalar>
</property>
<property title="Vibrational Frequencies" dictRef="me:vibFreqs">
<array units="cm-1">37.02 44.12 58.33 97.74 117.62 129.88 163.50 217.60 246.25
264.23 302.85 399.31 456.69 480.79 519.70 555.19 590.91 704.14 733.58 751.16 782.40 829.33
889.85 917.42 922.80 997.89 1020.86 1056.82 1059.82 1067.09 1081.92 1089.35 1139.06
1159.69 1192.35 1221.02 1232.26 1245.29 1273.31 1299.42 1318.37 1332.27 1344.98 1347.91
1381.86 1418.84 1422.54 1429.74 1458.10 1475.11 1494.03 1497.05 1506.36 1506.79 1516.37
1580.40 1873.66 3002.40 3023.22 3030.88 3038.16 3043.10 3050.16 3055.65 3072.08 3076.06
3086.40 3113.29 3121.41 3121.81 3130.94</array>
</property>
<property title="ImaginaryFrequency" dictRef="me:imFreqs">
<scalar units="cm-1">1688.96</scalar>
</property>
<property title="Rotational Constants" dictRef="me:rotConsts">
<array units="cm-1">0.079 0.012 0.012</array>
</property>
<property title="Symmetry Number" dictRef="me:symmetryNumber">
<scalar>1</scalar>
</property>
<property dictRef="me:frequenciesScaleFactor" >
<scalar>1</scalar>
</property>
</propertyList>
<me:DOSCMethod name="ClassicalRotors" />
</molecule>
<molecule id="Octanal-16-rad" spinMultiplicity="2">
<atomArray>
<atom id="a1" elementType="C" x3="-1.595243" y3="0.027557" z3="0.307284" />
<atom id="a2" elementType="O" x3="-1.181592" y3="0.549499" z3="1.312373" />
<atom id="a3" elementType="C" x3="-1.805395" y3="-1.440191" z3="0.082788" />
<atom id="a4" elementType="H" x3="-2.560048" y3="-1.767858" z3="0.799190" />
<atom id="a5" elementType="H" x3="-2.193364" y3="-1.603858" z3="-0.921223" />
<atom id="a6" elementType="C" x3="-0.497447" y3="-2.217989" z3="0.297480" />
<atom id="a7" elementType="H" x3="-0.745702" y3="-3.285362" z3="0.246895" />
<atom id="a8" elementType="H" x3="-0.137884" y3="-2.033442" z3="1.311413" />
<atom id="a9" elementType="C" spinMultiplicity="2" x3="0.556997" y3="-1.876745"
z3="-0.696240" />
<atom id="a10" elementType="C" x3="1.956587" y3="-1.583582" z3="-0.285284" />
<atom id="a11" elementType="H" x3="2.612862" y3="-1.604829" z3="-1.157458" />
<atom id="a12" elementType="H" x3="2.310182" y3="-2.367679" z3="0.394771" />
<atom id="a13" elementType="C" x3="2.114155" y3="-0.228603" z3="0.429233" />
<atom id="a14" elementType="H" x3="1.447530" y3="-0.197923" z3="1.295210" />
<atom id="a15" elementType="H" x3="3.132669" y3="-0.150702" z3="0.818803" />

<atom id="a16" elementType="O" x3="-1.918183" y3="0.746546" z3="-0.778655" />
<atom id="a17" elementType="H" x3="0.277491" y3="-1.835231" z3="-1.741704" />
<atom id="a18" elementType="C" x3="1.819920" y3="0.966464" z3="-0.468739" />
<atom id="a19" elementType="H" x3="0.832081" y3="0.849731" z3="-0.920642" />
<atom id="a20" elementType="H" x3="2.531129" y3="0.977216" z3="-1.299641" />
<atom id="a21" elementType="C" x3="1.879002" y3="2.290230" z3="0.280025" />
<atom id="a22" elementType="H" x3="2.870156" y3="2.458791" z3="0.705315" />
<atom id="a23" elementType="H" x3="1.647582" y3="3.129381" z3="-0.375811" />
<atom id="a24" elementType="H" x3="1.162909" y3="2.296472" z3="1.104109" />
<atom id="a25" elementType="O" x3="-1.702428" y3="2.127575" z3="-0.568710" />
<atom id="a26" elementType="H" x3="-1.341428" y3="2.123484" z3="0.341427" />
</atomArray>
<bondArray>
<bond atomRefs2="a17 a9" order="1" />
<bond atomRefs2="a20 a18" order="1" />
<bond atomRefs2="a11 a10" order="1" />
<bond atomRefs2="a5 a3" order="1" />
<bond atomRefs2="a19 a18" order="1" />
<bond atomRefs2="a16 a25" order="1" />
<bond atomRefs2="a16 a1" order="1" />
<bond atomRefs2="a9 a10" order="1" />
<bond atomRefs2="a9 a6" order="1" />
<bond atomRefs2="a25 a26" order="1" />
<bond atomRefs2="a18 a21" order="1" />
<bond atomRefs2="a18 a13" order="1" />
<bond atomRefs2="a23 a21" order="1" />
<bond atomRefs2="a10 a12" order="1" />
<bond atomRefs2="a10 a13" order="1" />
<bond atomRefs2="a3 a6" order="1" />
<bond atomRefs2="a3 a1" order="1" />
<bond atomRefs2="a3 a4" order="1" />
<bond atomRefs2="a7 a6" order="1" />
<bond atomRefs2="a21 a22" order="1" />
<bond atomRefs2="a21 a24" order="1" />
<bond atomRefs2="a6 a8" order="1" />
<bond atomRefs2="a1 a2" order="2" />
<bond atomRefs2="a13 a15" order="1" />
<bond atomRefs2="a13 a14" order="1" />
</bondArray>
<propertyList>
<property title="program">
<scalar>Gaussian 16, Revision C.01</scalar>
</property>
<property title="basis">
<scalar>Aug-CC-pVTZ (5D, 7F)</scalar>
</property>

<property title="File Format">
<scalar>g03</scalar>
</property>
<property title="Energy" dictRef="me:ZPE">
<scalar units="kcal/mol" >1.53</scalar>
</property>
<property title="SpinMultiplicity" dictRef="me:spinMultiplicity">
<scalar>2.00</scalar>
</property>
<property title="Vibrational Frequencies" dictRef="me:vibFreqs">
<array units="cm-1">41.99 51.51 69.73 91.30 136.26 159.79 173.79 221.56 254.11 259.29 294.93 333.57 349.26 403.67 430.76 457.55 480.30 535.93 633.52 726.15 747.70 815.81 843.15 887.45 904.07 939.79 964.90 977.25 1006.83 1046.65 1051.95 1070.06 1117.23 1130.61 1146.93 1196.59 1215.69 1249.28 1263.49 1289.74 1327.15 1333.19 1348.14 1362.48 1391.83 1408.65 1419.81 1432.54 1477.97 1485.86 1489.28 1498.41 1503.16 1506.10 1517.49 1537.67 1819.19 2997.99 3005.20 3036.73 3040.02 3043.26 3068.25 3077.34 3077.88 3086.40 3091.36 3110.78 3120.62 3135.50 3180.67 3565.15</array>
</property>
<property title="Rotational Constants" dictRef="me:rotConsts">
<array units="cm-1">0.038 0.033 0.021</array>
</property>
<property title="Symmetry Number" dictRef="me:symmetryNumber">
<scalar>1</scalar>
</property>
</propertyList>
</molecule>
<molecule id="16Hshift-TS" spinMultiplicity="2">
<atomArray>
<atom id="a1" elementType="C" x3="2.385301" y3="-0.476895" z3="-0.140175" />
<atom id="a2" elementType="O" x3="3.254682" y3="-0.819295" z3="-0.881466" />
<atom id="a3" elementType="C" x3="2.253928" y3="0.884965" z3="0.491375" />
<atom id="a4" elementType="H" x3="2.121179" y3="0.796788" z3="1.568512" />
<atom id="a5" elementType="H" x3="3.189934" y3="1.398110" z3="0.290065" />
<atom id="a6" elementType="C" x3="1.076644" y3="1.661135" z3="-0.133008" />
<atom id="a7" elementType="H" x3="1.136817" y3="1.578057" z3="-1.219952" />
<atom id="a8" elementType="H" x3="1.205239" y3="2.719537" z3="0.105552" />
<atom id="a9" elementType="C" spinMultiplicity="2" x3="-0.289253" y3="1.211303" z3="0.340565" />
<atom id="a10" elementType="C" x3="-1.402877" y3="1.236853" z3="-0.670364" />
<atom id="a11" elementType="H" x3="-1.137895" y3="0.587884" z3="-1.509537" />
<atom id="a12" elementType="H" x3="-1.446387" y3="2.250146" z3="-1.089564" />
<atom id="a13" elementType="C" x3="-2.778078" y3="0.869807" z3="-0.118933" />
<atom id="a14" elementType="H" x3="-3.053582" y3="1.594487" z3="0.652115" />
<atom id="a15" elementType="H" x3="-3.510400" y3="0.982627" z3="-0.922241" />
<atom id="a16" elementType="O" x3="1.354513" y3="-1.364441" z3="0.058394" />
<atom id="a17" elementType="O" x3="0.507055" y3="-1.017355" z3="1.101080" />

```
<atom id="a18" elementType="H" x3="-0.563008" y3="1.621075" z3="1.315021" />
<atom id="a19" elementType="H" x3="-0.084746" y3="-0.008250" z3="0.698849" />
<atom id="a20" elementType="C" x3="-2.887884" y3="-0.541720" z3="0.458052" />
<atom id="a21" elementType="H" x3="-3.905974" y3="-0.677901" z3="0.828007" />
<atom id="a22" elementType="H" x3="-2.236182" y3="-0.640051" z3="1.329409" />
<atom id="a23" elementType="C" x3="-2.560215" y3="-1.644500" z3="-0.540953" />
<atom id="a24" elementType="H" x3="-1.511302" y3="-1.623104" z3="-0.836953" />
<atom id="a25" elementType="H" x3="-2.750059" y3="-2.626797" z3="-0.110129" />
<atom id="a26" elementType="H" x3="-3.169021" y3="-1.549566" z3="-1.442567" />
</atomArray>
<bondArray>
<bond atomRefs2="a11 a10" order="1" />
<bond atomRefs2="a26 a23" order="1" />
<bond atomRefs2="a7 a6" order="1" />
<bond atomRefs2="a12 a10" order="1" />
<bond atomRefs2="a15 a13" order="1" />
<bond atomRefs2="a2 a1" order="2" />
<bond atomRefs2="a24 a23" order="1" />
<bond atomRefs2="a10 a13" order="1" />
<bond atomRefs2="a10 a9" order="1" />
<bond atomRefs2="a23 a25" order="1" />
<bond atomRefs2="a23 a20" order="1" />
<bond atomRefs2="a1 a16" order="1" />
<bond atomRefs2="a1 a3" order="1" />
<bond atomRefs2="a6 a8" order="1" />
<bond atomRefs2="a6 a9" order="1" />
<bond atomRefs2="a6 a3" order="1" />
<bond atomRefs2="a13 a20" order="1" />
<bond atomRefs2="a13 a14" order="1" />
<bond atomRefs2="a16 a17" order="1" />
<bond atomRefs2="a5 a3" order="1" />
<bond atomRefs2="a9 a18" order="1" />
<bond atomRefs2="a20 a21" order="1" />
<bond atomRefs2="a20 a22" order="1" />
<bond atomRefs2="a3 a4" order="1" />
<bond atomRefs2="a19 a17" order="1" />
</bondArray>
<propertyList>
<property title="program">
<scalar>Gaussian 16, Revision C.01</scalar>
</property>
<property title="basis">
<scalar>Aug-CC-pVTZ (5D, 7F)</scalar>
</property>
<property title="File Format">
<scalar>g03</scalar>
```

</property>
<property title="Energy" dictRef="me:ZPE">
<scalar units="kcal/mol" >19.51</scalar>
</property>
<property title="SpinMultiplicity" dictRef="me:spinMultiplicity">
<scalar>2.00</scalar>
</property>
<property title="Vibrational Frequencies" dictRef="me:vibFreqs">
<array units="cm-1">32.34 45.38 78.95 114.80 151.72 183.86 237.48 254.98 289.40 306.95 358.23 396.75 414.87 492.90 513.70 549.90 583.49 733.50 745.13 769.56 807.64 857.64 871.94 906.41 915.97 978.16 1011.07 1039.72 1047.97 1073.51 1090.01 1122.41 1134.01 1140.79 1170.87 1196.93 1249.00 1259.48 1271.96 1299.06 1312.63 1332.45 1366.18 1373.81 1390.98 1398.48 1417.35 1428.39 1457.47 1472.64 1483.71 1494.97 1496.19 1500.13 1512.16 1518.43 1872.16 3004.28 3036.62 3045.25 3047.24 3050.76 3059.94 3075.27 3079.24 3088.10 3093.48 3104.15 3117.91 3129.43 3161.67</array>
</property>
<property title="ImaginaryFrequency" dictRef="me:imFreqs">
<scalar units="cm-1">1753.64</scalar>
</property>
<property title="Rotational Constants" dictRef="me:rotConsts">
<array units="cm-1">0.059 0.021 0.018</array>
</property>
<property title="Symmetry Number" dictRef="me:symmetryNumber">
<scalar>1</scalar>
</property>
<property dictRef="me:frequenciesScaleFactor" >
<scalar>1</scalar>
</property>
</propertyList>
<me:DOSCMethod name="ClassicalRotors" />
</molecule>
<molecule id="Octanal-17-rad" spinMultiplicity="2">
<atomArray>
<atom id="a1" elementType="C" x3="-1.385975" y3="-1.928389" z3="0.398237" />
<atom id="a2" elementType="H" x3="-0.937943" y3="-1.829386" z3="1.387798" />
<atom id="a3" elementType="H" x3="-2.077343" y3="-2.770663" z3="0.443932" />
<atom id="a4" elementType="C" x3="-0.298202" y3="-2.229470" z3="-0.648699" />
<atom id="a5" elementType="H" x3="-0.776735" y3="-2.344494" z3="-1.623724" />
<atom id="a6" elementType="C" spinMultiplicity="2" x3="0.783575" y3="-1.210242" z3="-0.725812" />
<atom id="a7" elementType="H" x3="0.832725" y3="-0.548773" z3="-1.581014" />
<atom id="a8" elementType="C" x3="-2.189655" y3="-0.667756" z3="0.083647" />
<atom id="a9" elementType="H" x3="-2.558137" y3="-0.682704" z3="-0.941559" />
<atom id="a10" elementType="H" x3="-3.055172" y3="-0.605647" z3="0.746198" />
<atom id="a11" elementType="C" x3="-1.410741" y3="0.593824" z3="0.315540" />
<atom id="a12" elementType="O" x3="-0.815178" y3="0.901364" z3="1.318054" />

<atom id="a13" elementType="C" x3="1.804585" y3="-1.098529" z3="0.348176" />
<atom id="a14" elementType="H" x3="1.320942" y3="-0.816787" z3="1.294705" />
<atom id="a15" elementType="H" x3="2.233972" y3="-2.092489" z3="0.533115" />
<atom id="a16" elementType="O" x3="-1.489808" y3="1.419861" z3="-0.740741" />
<atom id="a17" elementType="H" x3="0.136023" y3="-3.203533" z3="-0.393615" />
<atom id="a18" elementType="O" x3="-0.807544" y3="2.634292" z3="-0.501319" />
<atom id="a19" elementType="C" x3="2.926669" y3="-0.104036" z3="0.056065" />
<atom id="a20" elementType="H" x3="3.391305" y3="-0.359661" z3="-0.899733" />
<atom id="a21" elementType="H" x3="3.702137" y3="-0.216722" z3="0.816116" />
<atom id="a22" elementType="C" x3="2.457126" y3="1.345146" z3="0.029567" />
<atom id="a23" elementType="H" x3="1.706318" y3="1.511725" z3="-0.743690" />
<atom id="a24" elementType="H" x3="2.010240" y3="1.619789" z3="0.986825" />
<atom id="a25" elementType="H" x3="3.287134" y3="2.024593" z3="-0.164056" />
<atom id="a26" elementType="H" x3="-0.439507" y3="2.467327" z3="0.390417" />
</atomArray>
<bondArray>
<bond atomRefs2="a5 a4" order="1" />
<bond atomRefs2="a7 a6" order="1" />
<bond atomRefs2="a9 a8" order="1" />
<bond atomRefs2="a20 a19" order="1" />
<bond atomRefs2="a23 a22" order="1" />
<bond atomRefs2="a16 a18" order="1" />
<bond atomRefs2="a16 a11" order="1" />
<bond atomRefs2="a6 a4" order="1" />
<bond atomRefs2="a6 a13" order="1" />
<bond atomRefs2="a4 a17" order="1" />
<bond atomRefs2="a4 a1" order="1" />
<bond atomRefs2="a18 a26" order="1" />
<bond atomRefs2="a25 a22" order="1" />
<bond atomRefs2="a22 a19" order="1" />
<bond atomRefs2="a22 a24" order="1" />
<bond atomRefs2="a19 a13" order="1" />
<bond atomRefs2="a19 a21" order="1" />
<bond atomRefs2="a8 a11" order="1" />
<bond atomRefs2="a8 a1" order="1" />
<bond atomRefs2="a8 a10" order="1" />
<bond atomRefs2="a11 a12" order="2" />
<bond atomRefs2="a13 a15" order="1" />
<bond atomRefs2="a13 a14" order="1" />
<bond atomRefs2="a1 a3" order="1" />
<bond atomRefs2="a1 a2" order="1" />
</bondArray>
<propertyList>
<property title="program">
<scalar>Gaussian 16, Revision C.01</scalar>
</property>

<property title="basis">
<scalar>Aug-CC-pVTZ (5D, 7F)</scalar>
</property>
<property title="File Format">
<scalar>g03</scalar>
</property>
<property title="Energy" dictRef="me:ZPE">
<scalar units="kcal/mol" >1.59</scalar>
</property>
<property title="SpinMultiplicity" dictRef="me:spinMultiplicity">
<scalar>2.00</scalar>
</property>
<property title="Vibrational Frequencies" dictRef="me:vibFreqs">
<array units="cm-1">39.54 66.47 76.46 87.35 112.73 161.94 194.52 212.79 247.04 260.97 301.52 320.49 353.38 366.35 432.27 457.06 478.21 543.56 620.73 726.77 773.69 827.76 851.57 873.35 905.75 934.25 943.24 987.98 1013.64 1055.91 1066.92 1077.86 1096.30 1128.85 1158.37 1188.78 1228.55 1244.98 1257.41 1293.81 1314.23 1342.54 1364.77 1383.80 1386.71 1403.78 1426.38 1436.98 1468.52 1476.97 1488.06 1496.46 1499.38 1507.38 1515.77 1522.51 1816.53 2975.68 2995.01 3005.96 3044.91 3045.19 3064.65 3070.82 3076.31 3080.65 3111.69 3117.81 3123.82 3127.30 3189.69 3564.11</array>
</property>
<property title="Rotational Constants" dictRef="me:rotConsts">
<array units="cm-1">0.038 0.032 0.020</array>
</property>
<property title="Symmetry Number" dictRef="me:symmetryNumber">
<scalar>1</scalar>
</property>
</propertyList>
</molecule>
<molecule id="17Hshift-TS" spinMultiplicity="2">
<atomArray>
<atom id="a1" elementType="C" x3="0.969050" y3="1.683483" z3="0.366542" />
<atom id="a2" elementType="H" x3="0.236368" y3="2.209758" z3="0.979383" />
<atom id="a3" elementType="H" x3="1.804369" y3="2.368225" z3="0.219901" />
<atom id="a4" elementType="C" x3="0.375754" y3="1.360940" z3="-1.007678" />
<atom id="a5" elementType="H" x3="1.168282" y3="1.015949" z3="-1.676063" />
<atom id="a6" elementType="C" spinMultiplicity="2" x3="-0.748635" y3="0.355304" z3="-1.025251" />
<atom id="a7" elementType="H" x3="-1.049923" y3="0.080108" z3="-2.036580" />
<atom id="a8" elementType="C" x3="1.469859" y3="0.466410" z3="1.168579" />
<atom id="a9" elementType="H" x3="2.078845" y3="0.811607" z3="2.000443" />
<atom id="a10" elementType="H" x3="0.637898" y3="-0.112716" z3="1.557420" />
<atom id="a11" elementType="C" x3="2.359722" y3="-0.396826" z3="0.316148" />
<atom id="a12" elementType="O" x3="3.531057" y3="-0.236802" z3="0.154224" />
<atom id="a13" elementType="C" x3="-1.914513" y3="0.560277" z3="-0.095056" />
<atom id="a14" elementType="H" x3="-1.564504" y3="0.636470" z3="0.938056" />

<atom id="a15" elementType="H" x3="-2.372452" y3="1.531334" z3="-0.326642" />
<atom id="a16" elementType="O" x3="1.774977" y3="-1.402602" z3="-0.422439" />
<atom id="a17" elementType="H" x3="0.006624" y3="2.294011" z3="-1.448784" />
<atom id="a18" elementType="O" x3="0.449299" y3="-1.662423" z3="-0.110901" />
<atom id="a19" elementType="H" x3="-0.193581" y3="-0.749255" z3="-0.632784" />
<atom id="a20" elementType="C" x3="-2.970446" y3="-0.535340" z3="-0.192384" />
<atom id="a21" elementType="H" x3="-2.505786" y3="-1.499022" z3="0.028242" />
<atom id="a22" elementType="H" x3="-3.329172" y3="-0.595707" z3="-1.222953" />
<atom id="a23" elementType="C" x3="-4.144289" y3="-0.302989" z3="0.748394" />
<atom id="a24" elementType="H" x3="-4.640368" y3="0.644452" z3="0.530470" />
<atom id="a25" elementType="H" x3="-4.885851" y3="-1.096125" z3="0.659530" />
<atom id="a26" elementType="H" x3="-3.812426" y3="-0.272025" z3="1.787527" />
</atomArray>
<bondArray>
<bond atomRefs2="a7 a6" order="1" />
<bond atomRefs2="a5 a4" order="1" />
<bond atomRefs2="a17 a4" order="1" />
<bond atomRefs2="a22 a20" order="1" />
<bond atomRefs2="a6 a4" order="1" />
<bond atomRefs2="a6 a13" order="1" />
<bond atomRefs2="a4 a1" order="1" />
<bond atomRefs2="a19 a18" order="1" />
<bond atomRefs2="a16 a18" order="1" />
<bond atomRefs2="a16 a11" order="1" />
<bond atomRefs2="a15 a13" order="1" />
<bond atomRefs2="a20 a13" order="1" />
<bond atomRefs2="a20 a21" order="1" />
<bond atomRefs2="a20 a23" order="1" />
<bond atomRefs2="a13 a14" order="1" />
<bond atomRefs2="a12 a11" order="2" />
<bond atomRefs2="a3 a1" order="1" />
<bond atomRefs2="a11 a8" order="1" />
<bond atomRefs2="a1 a2" order="1" />
<bond atomRefs2="a1 a8" order="1" />
<bond atomRefs2="a24 a23" order="1" />
<bond atomRefs2="a25 a23" order="1" />
<bond atomRefs2="a23 a26" order="1" />
<bond atomRefs2="a8 a10" order="1" />
<bond atomRefs2="a8 a9" order="1" />
</bondArray>
<propertyList>
<property title="program">
<scalar>Gaussian 16, Revision C.01</scalar>
</property>
<property title="basis">
<scalar>Aug-CC-pVTZ (5D, 7F)</scalar>

</property>
<property title="File Format">
<scalar>g03</scalar>
</property>
<property title="Energy" dictRef="me:ZPE">
<scalar units="kcal/mol" >17.65</scalar>
</property>
<property title="SpinMultiplicity" dictRef="me:spinMultiplicity">
<scalar>2.00</scalar>
</property>
<property title="Vibrational Frequencies" dictRef="me:vibFreqs">
<array units="cm-1">51.96 71.89 75.28 113.49 153.78 200.27 217.63 245.14 274.86 288.40 351.33 412.34 423.41 510.07 551.66 580.99 588.79 728.98 738.62 758.51 808.67 818.50 852.87 908.50 936.93 953.22 1004.93 1028.66 1065.62 1081.86 1087.35 1105.81 1137.49 1145.23 1173.87 1205.34 1234.73 1248.65 1276.56 1295.08 1315.04 1342.48 1362.66 1376.99 1388.75 1408.35 1419.32 1424.91 1462.18 1475.79 1485.95 1499.51 1501.67 1506.49 1510.39 1512.95 1869.45 2996.64 3020.43 3043.68 3044.55 3059.12 3068.06 3076.29 3083.46 3097.96 3110.62 3114.66 3115.75 3125.62 3171.86</array>
</property>
<property title="ImaginaryFrequency" dictRef="me:imFreqs">
<scalar units="cm-1">1766.33</scalar>
</property>
<property title="Rotational Constants" dictRef="me:rotConsts">
<array units="cm-1">0.067 0.019 0.017</array>
</property>
<property title="Symmetry Number" dictRef="me:symmetryNumber">
<scalar>1</scalar>
</property>
<property dictRef="me:frequenciesScaleFactor" >
<scalar>1</scalar>
</property>
</propertyList>
<me:DOSCMethod name="ClassicalRotors" />
</molecule>
<molecule id="Octanal-18-rad" spinMultiplicity="2">
<atomArray>
<atom id="a1" elementType="C" x3="-1.934413" y3="-0.102501" z3="0.368613" />
<atom id="a2" elementType="O" x3="-1.385639" y3="-1.142518" z3="0.635931" />
<atom id="a3" elementType="C" x3="-1.526919" y3="1.272490" z3="0.813260" />
<atom id="a4" elementType="H" x3="-2.048414" y3="2.013483" z3="0.208026" />
<atom id="a5" elementType="H" x3="-1.885751" y3="1.379773" z3="1.839277" />
<atom id="a6" elementType="C" x3="-0.013851" y3="1.460379" z3="0.756948" />
<atom id="a7" elementType="H" x3="0.463942" y3="0.680830" z3="1.352022" />
<atom id="a8" elementType="H" x3="0.234628" y3="2.413249" z3="1.228089" />
<atom id="a9" elementType="C" x3="0.536719" y3="1.433579" z3="-0.663837" />
<atom id="a10" elementType="C" x3="2.066350" y3="1.531455" z3="-0.707627" />

<atom id="a11" elementType="H" x3="2.380928" y3="2.479229" z3="-0.264764" />
<atom id="a12" elementType="H" x3="2.367018" y3="1.578248" z3="-1.764710" />
<atom id="a13" elementType="C" spinMultiplicity="2" x3="2.769793" y3="0.402061" z3="-0.037325" />
<atom id="a14" elementType="H" x3="3.646013" y3="0.609580" z3="0.563747" />
<atom id="a15" elementType="O" x3="-3.042847" y3="-0.066249" z3="-0.386500" />
<atom id="a16" elementType="H" x3="0.106294" y3="2.256728" z3="-1.239341" />
<atom id="a17" elementType="H" x3="0.222408" y3="0.512488" z3="-1.161898" />
<atom id="a18" elementType="C" x3="2.406822" y3="-1.014547" z3="-0.309133" />
<atom id="a19" elementType="H" x3="2.400850" y3="-1.189075" z3="-1.394889" />
<atom id="a20" elementType="H" x3="1.373736" y3="-1.208287" z3="0.007356" />
<atom id="a21" elementType="C" x3="3.333550" y3="-2.018528" z3="0.365093" />
<atom id="a22" elementType="H" x3="4.362788" y3="-1.888242" z3="0.027806" />
<atom id="a23" elementType="H" x3="3.318642" y3="-1.890157" z3="1.448251" />
<atom id="a24" elementType="H" x3="3.033779" y3="-3.042725" z3="0.145545" />
<atom id="a25" elementType="O" x3="-3.452926" y3="-1.364005" z3="-0.768984" />
<atom id="a26" elementType="H" x3="-2.753868" y3="-1.909276" z3="-0.354053" />
</atomArray>
<bondArray>
<bond atomRefs2="a12 a10" order="1" />
<bond atomRefs2="a19 a18" order="1" />
<bond atomRefs2="a16 a9" order="1" />
<bond atomRefs2="a17 a9" order="1" />
<bond atomRefs2="a25 a15" order="1" />
<bond atomRefs2="a25 a26" order="1" />
<bond atomRefs2="a10 a9" order="1" />
<bond atomRefs2="a10 a11" order="1" />
<bond atomRefs2="a10 a13" order="1" />
<bond atomRefs2="a9 a6" order="1" />
<bond atomRefs2="a15 a1" order="1" />
<bond atomRefs2="a18 a13" order="1" />
<bond atomRefs2="a18 a20" order="1" />
<bond atomRefs2="a18 a21" order="1" />
<bond atomRefs2="a13 a14" order="1" />
<bond atomRefs2="a22 a21" order="1" />
<bond atomRefs2="a24 a21" order="1" />
<bond atomRefs2="a4 a3" order="1" />
<bond atomRefs2="a21 a23" order="1" />
<bond atomRefs2="a1 a2" order="2" />
<bond atomRefs2="a1 a3" order="1" />
<bond atomRefs2="a6 a3" order="1" />
<bond atomRefs2="a6 a8" order="1" />
<bond atomRefs2="a6 a7" order="1" />
<bond atomRefs2="a3 a5" order="1" />
</bondArray>
<propertyList>

<property title="program">
<scalar>Gaussian 16, Revision C.01</scalar>
</property>
<property title="basis">
<scalar>Aug-CC-pVTZ (5D, 7F)</scalar>
</property>
<property title="File Format">
<scalar>g03</scalar>
</property>
<property title="Energy" dictRef="me:ZPE">
<scalar units="kcal/mol" >1.71</scalar>
</property>
<property title="SpinMultiplicity" dictRef="me:spinMultiplicity">
<scalar>2.00</scalar>
</property>
<property title="Vibrational Frequencies" dictRef="me:vibFreqs">
<array units="cm-1">19.23 25.64 31.38 73.84 81.70 131.27 203.84 211.76 230.83 232.11
272.58 302.07 321.48 362.84 419.89 469.26 494.01 581.69 625.05 700.17 773.73 801.40 839.65
851.02 911.96 947.52 979.66 1000.91 1040.99 1052.49 1063.33 1075.02 1087.04 1129.29
1163.01 1179.57 1216.24 1251.33 1280.17 1287.26 1317.99 1334.51 1349.00 1382.21 1404.29
1415.89 1420.19 1432.36 1475.62 1476.21 1479.89 1494.39 1504.31 1508.17 1508.88 1520.40
1817.18 2964.41 2971.71 3004.81 3042.33 3046.48 3060.33 3064.22 3070.04 3081.47 3108.30
3115.14 3124.73 3125.88 3182.62 3562.37</array>
</property>
<property title="Rotational Constants" dictRef="me:rotConsts">
<array units="cm-1">0.051 0.018 0.015</array>
</property>
<property title="Symmetry Number" dictRef="me:symmetryNumber">
<scalar>1</scalar>
</property>
</propertyList>
</molecule>
<molecule id="18Hshift-TS" spinMultiplicity="2">
<atomArray>
<atom id="a1" elementType="C" x3="-1.501773" y3="-1.144074" z3="0.238543" />
<atom id="a2" elementType="O" x3="-1.951481" y3="-1.556080" z3="1.265445" />
<atom id="a3" elementType="C" x3="-2.183913" y3="-0.142472" z3="-0.656029" />
<atom id="a4" elementType="H" x3="-1.776592" y3="-0.167731" z3="-1.661931" />
<atom id="a5" elementType="H" x3="-3.221761" y3="-0.471745" z3="-0.687267" />
<atom id="a6" elementType="C" x3="-2.149454" y3="1.286590" z3="-0.092802" />
<atom id="a7" elementType="H" x3="-2.456037" y3="1.260333" z3="0.955165" />
<atom id="a8" elementType="H" x3="-2.913105" y3="1.856277" z3="-0.623897" />
<atom id="a9" elementType="C" x3="-0.820687" y3="2.030627" z3="-0.226331" />
<atom id="a10" elementType="C" x3="0.330405" y3="1.490501" z3="0.642184" />
<atom id="a11" elementType="H" x3="-0.048319" y3="0.792716" z3="1.394152" />
<atom id="a12" elementType="H" x3="0.770953" y3="2.312402" z3="1.217544" />

<atom id="a13" elementType="C" spinMultiplicity="2" x3="1.452196" y3="0.838740" z3="-0.123200" />
<atom id="a14" elementType="H" x3="0.901581" y3="-0.105723" z3="-0.798687" />
<atom id="a15" elementType="H" x3="1.844180" y3="1.460617" z3="-0.931703" />
<atom id="a16" elementType="O" x3="-0.273266" y3="-1.649075" z3="-0.124155" />
<atom id="a17" elementType="O" x3="0.273958" y3="-1.073644" z3="-1.258532" />
<atom id="a18" elementType="H" x3="-1.005523" y3="3.073716" z3="0.032074" />
<atom id="a19" elementType="H" x3="-0.515963" y3="2.030125" z3="-1.276195" />
<atom id="a20" elementType="C" x3="2.516477" y3="0.145682" z3="0.683921" />
<atom id="a21" elementType="H" x3="2.041822" y3="-0.595576" z3="1.331729" />
<atom id="a22" elementType="H" x3="2.971385" y3="0.885327" z3="1.354145" />
<atom id="a23" elementType="C" x3="3.592448" y3="-0.514091" z3="-0.167699" />
<atom id="a24" elementType="H" x3="4.346491" y3="-0.992152" z3="0.456099" />
<atom id="a25" elementType="H" x3="3.157021" y3="-1.275856" z3="-0.814423" />
<atom id="a26" elementType="H" x3="4.095979" y3="0.218643" z3="-0.800392" />
</atomArray>
<bondArray>
<bond atomRefs2="a4 a3" order="1" />
<bond atomRefs2="a19 a9" order="1" />
<bond atomRefs2="a17 a14" order="1" />
<bond atomRefs2="a17 a16" order="1" />
<bond atomRefs2="a15 a13" order="1" />
<bond atomRefs2="a25 a23" order="1" />
<bond atomRefs2="a26 a23" order="1" />
<bond atomRefs2="a5 a3" order="1" />
<bond atomRefs2="a3 a6" order="1" />
<bond atomRefs2="a3 a1" order="1" />
<bond atomRefs2="a8 a6" order="1" />
<bond atomRefs2="a9 a6" order="1" />
<bond atomRefs2="a9 a18" order="1" />
<bond atomRefs2="a9 a10" order="1" />
<bond atomRefs2="a23 a24" order="1" />
<bond atomRefs2="a23 a20" order="1" />
<bond atomRefs2="a16 a1" order="1" />
<bond atomRefs2="a13 a10" order="1" />
<bond atomRefs2="a13 a20" order="1" />
<bond atomRefs2="a6 a7" order="1" />
<bond atomRefs2="a1 a2" order="2" />
<bond atomRefs2="a10 a12" order="1" />
<bond atomRefs2="a10 a11" order="1" />
<bond atomRefs2="a20 a21" order="1" />
<bond atomRefs2="a20 a22" order="1" />
</bondArray>
<propertyList>
<property title="program">
<scalar>Gaussian 16, Revision C.01</scalar>

</property>
<property title="basis">
<scalar>Aug-CC-pVTZ (5D, 7F)</scalar>
</property>
<property title="File Format">
<scalar>g03</scalar>
</property>
<property title="Energy" dictRef="me:ZPE">
<scalar units="kcal/mol" >18.36</scalar>
</property>
<property title="SpinMultiplicity" dictRef="me:spinMultiplicity">
<scalar>2.00</scalar>
</property>
<property title="Vibrational Frequencies" dictRef="me:vibFreqs">
<array units="cm-1">49.82 74.77 88.63 126.60 132.53 191.54 229.31 249.79 265.71
357.23 366.94 400.19 420.27 470.78 520.56 554.16 592.32 694.54 767.81 775.45 785.43 872.93
881.19 930.10 937.11 966.00 1004.37 1046.93 1064.93 1095.13 1097.51 1108.84 1131.01
1135.05 1168.24 1170.85 1228.93 1253.31 1280.78 1287.47 1310.39 1326.34 1367.84 1388.44
1397.99 1404.97 1419.89 1424.76 1447.50 1476.97 1488.85 1497.56 1502.14 1504.25 1506.08
1511.49 1861.86 3008.47 3018.62 3046.24 3049.57 3055.27 3061.56 3067.32 3080.37 3092.15
3096.32 3103.67 3121.45 3133.32 3164.42</array>
</property>
<property title="ImaginaryFrequency" dictRef="me:imFreqs">
<scalar units="cm-1">1677.33</scalar>
</property>
<property title="Rotational Constants" dictRef="me:rotConsts">
<array units="cm-1">0.050 0.026 0.021</array>
</property>
<property title="Symmetry Number" dictRef="me:symmetryNumber">
<scalar>1</scalar>
</property>
<property dictRef="me:frequenciesScaleFactor" >
<scalar>1</scalar>
</property>
</propertyList>
<me:DOSCMethod name="ClassicalRotors" />
</molecule>
<molecule id="Octanal-AcylOO-rad" spinMultiplicity="2">
<atomArray>
<atom id="a1" elementType="C" x3="-2.475650" y3="5.169719" z3="0.000000" />
<atom id="a2" elementType="H" x3="-1.933521" y3="5.518670" z3="0.880699" />
<atom id="a3" elementType="H" x3="-3.455004" y3="5.648035" z3="0.000000" />
<atom id="a4" elementType="H" x3="-1.933521" y3="5.518670" z3="-0.880699" />
<atom id="a5" elementType="C" x3="-2.595983" y3="3.651623" z3="0.000000" />
<atom id="a6" elementType="H" x3="-3.167903" y3="3.328950" z3="0.874281" />
<atom id="a7" elementType="H" x3="-3.167903" y3="3.328950" z3="-0.874281" />

<atom id="a8" elementType="C" x3="-1.243848" y3="2.949091" z3="0.000000" />
<atom id="a9" elementType="H" x3="-0.670048" y3="3.271198" z3="-0.874955" />
<atom id="a10" elementType="H" x3="-0.670048" y3="3.271198" z3="0.874955" />
<atom id="a11" elementType="C" x3="-1.352856" y3="1.429942" z3="0.000000" />
<atom id="a12" elementType="H" x3="-1.926035" y3="1.107756" z3="0.874813" />
<atom id="a13" elementType="H" x3="-1.926035" y3="1.107756" z3="-0.874813" />
<atom id="a14" elementType="C" x3="0.000000" y3="0.730191" z3="0.000000" />
<atom id="a15" elementType="H" x3="0.573516" y3="1.051618" z3="-0.875105" />
<atom id="a16" elementType="H" x3="0.573516" y3="1.051618" z3="0.875105" />
<atom id="a17" elementType="C" x3="-0.118274" y3="-0.788453" z3="0.000000" />
<atom id="a18" elementType="H" x3="-0.686379" y3="-1.116430" z3="0.872499" />
<atom id="a19" elementType="H" x3="-0.686379" y3="-1.116430" z3="-0.872499" />
<atom id="a20" elementType="C" x3="1.245205" y3="-1.463672" z3="0.000000" />
<atom id="a21" elementType="H" x3="1.839990" y3="-1.167216" z3="-0.866327" />
<atom id="a22" elementType="H" x3="1.839990" y3="-1.167216" z3="0.866327" />
<atom id="a23" elementType="C" x3="1.135961" y3="-2.954294" z3="0.000000" />
<atom id="a24" elementType="O" x3="0.165391" y3="-3.629849" z3="0.000000" />
<atom id="a25" elementType="O" x3="2.371549" y3="-3.682639" z3="0.000000" />
<atom id="a26" elementType="O" spinMultiplicity="2" x3="3.441614" y3="-2.935265"
z3="0.000000" />
</atomArray>
<bondArray>
<bond atomRefs2="a4 a1" order="1" />
<bond atomRefs2="a15 a14" order="1" />
<bond atomRefs2="a9 a8" order="1" />
<bond atomRefs2="a13 a11" order="1" />
<bond atomRefs2="a7 a5" order="1" />
<bond atomRefs2="a19 a17" order="1" />
<bond atomRefs2="a21 a20" order="1" />
<bond atomRefs2="a1 a3" order="1" />
<bond atomRefs2="a1 a5" order="1" />
<bond atomRefs2="a1 a2" order="1" />
<bond atomRefs2="a5 a8" order="1" />
<bond atomRefs2="a5 a6" order="1" />
<bond atomRefs2="a8 a11" order="1" />
<bond atomRefs2="a8 a10" order="1" />
<bond atomRefs2="a11 a14" order="1" />
<bond atomRefs2="a11 a12" order="1" />
<bond atomRefs2="a14 a17" order="1" />
<bond atomRefs2="a14 a16" order="1" />
<bond atomRefs2="a17 a20" order="1" />
<bond atomRefs2="a17 a18" order="1" />
<bond atomRefs2="a20 a23" order="1" />
<bond atomRefs2="a20 a22" order="1" />
<bond atomRefs2="a23 a24" order="2" />
<bond atomRefs2="a23 a25" order="1" />

<bond atomRefs2="a25 a26" order="1" />
</bondArray>
<propertyList>
<property title="program">
<scalar>Gaussian 16, Revision C.01</scalar>
</property>
<property title="basis">
<scalar>Aug-CC-pVTZ (5D, 7F)</scalar>
</property>
<property title="File Format">
<scalar>g03</scalar>
</property>
<property title="Energy" dictRef="me:ZPE">
<scalar units="kcal/mol" >0.0</scalar>
</property>
<property title="SpinMultiplicity" dictRef="me:spinMultiplicity">
<scalar>2.00</scalar>
</property>
<property title="Vibrational Frequencies" dictRef="me:vibFreqs">
<array units="cm-1">35.74 41.52 56.36 93.40 101.65 136.50 145.72 154.26 158.02
202.96 245.57 272.62 358.57 435.13 481.75 529.94 563.25 611.25 733.64 740.98 777.19 826.73
846.25 912.60 933.65 1018.98 1025.86 1049.17 1060.61 1078.70 1090.42 1093.76 1142.21
1154.48 1223.57 1248.71 1259.69 1269.92 1311.76 1315.88 1336.52 1348.62 1351.19 1361.80
1401.87 1420.99 1423.63 1429.73 1451.81 1492.46 1493.32 1497.63 1503.82 1505.31 1512.18
1517.91 1921.74 3020.46 3022.46 3029.65 3037.26 3041.93 3044.75 3053.36 3059.15 3069.23
3072.80 3082.09 3096.11 3111.73 3115.02 3120.43</array>
</property>
<property title="Rotational Constants" dictRef="me:rotConsts">
<array units="cm-1">0.133 0.009 0.008</array>
</property>
<property title="Symmetry Number" dictRef="me:symmetryNumber">
<scalar>1</scalar>
</property>
<property dictRef="me:sigma" >
<scalar>7.72</scalar>
</property>
<property dictRef="me:epsilon" >
<scalar>363.0</scalar>
</property>
<property dictRef="me:frequenciesScaleFactor" >
<scalar>1</scalar>
</property>
</propertyList>
<me:DOSCMethod name="ClassicalRotors" />
<me:DistributionCalcMethod name="Boltzmann" />
<me:energyTransferModel name="ExponentialDown" />

<me:deltaEDown >225.0</me:deltaEDown>
</molecule>
<molecule id="Octanal-19-rad" spinMultiplicity="2">
<atomArray>
<atom id="a1" elementType="C" x3="-1.931078" y3="-0.066476" z3="-0.017323" />
<atom id="a2" elementType="O" x3="-2.486443" y3="0.057568" z3="1.044868" />
<atom id="a3" elementType="C" x3="-1.534764" y3="-1.349941" z3="-0.682946" />
<atom id="a4" elementType="H" x3="-2.452999" y3="-1.911413" z3="-0.858219" />
<atom id="a5" elementType="H" x3="-1.085080" y3="-1.132663" z3="-1.649543" />
<atom id="a6" elementType="C" x3="-0.590212" y3="-2.164542" z3="0.208652" />
<atom id="a7" elementType="H" x3="-0.351637" y3="-3.090181" z3="-0.318951" />
<atom id="a8" elementType="H" x3="-1.126444" y3="-2.443426" z3="1.116101" />
<atom id="a9" elementType="C" x3="0.697592" y3="-1.436203" z3="0.584458" />
<atom id="a10" elementType="C" x3="1.572431" y3="-1.044182" z3="-0.600444" />
<atom id="a11" elementType="H" x3="1.037399" y3="-0.345231" z3="-1.247881" />
<atom id="a12" elementType="H" x3="1.788263" y3="-1.928994" z3="-1.206050" />
<atom id="a13" elementType="C" x3="2.898526" y3="-0.393531" z3="-0.168878" />
<atom id="a14" elementType="H" x3="3.468362" y3="-1.107820" z3="0.428927" />
<atom id="a15" elementType="H" x3="3.485651" y3="-0.204778" z3="-1.076735" />
<atom id="a16" elementType="O" x3="-1.571167" y3="1.000662" z3="-0.751202" />
<atom id="a17" elementType="O" x3="-1.924493" y3="2.210837" z3="-0.111500" />
<atom id="a18" elementType="H" x3="1.273547" y3="-2.079783" z3="1.254036" />
<atom id="a19" elementType="H" x3="0.456888" y3="-0.542158" z3="1.167583" />
<atom id="a20" elementType="C" spinMultiplicity="2" x3="2.725183" y3="0.880634" z3="0.582090" />
<atom id="a21" elementType="H" x3="-2.342305" y3="1.871721" z3="0.706846" />
<atom id="a22" elementType="H" x3="2.991174" y3="0.922919" z3="1.629270" />
<atom id="a23" elementType="C" x3="2.030481" y3="2.038918" z3="-0.035188" />
<atom id="a24" elementType="H" x3="0.938240" y3="1.924920" z3="-0.023119" />
<atom id="a25" elementType="H" x3="2.255400" y3="2.969940" z3="0.484066" />
<atom id="a26" elementType="H" x3="2.311413" y3="2.156350" z3="-1.086197" />
</atomArray>
<bondArray>
<bond atomRefs2="a5 a3" order="1" />
<bond atomRefs2="a11 a10" order="1" />
<bond atomRefs2="a12 a10" order="1" />
<bond atomRefs2="a26 a23" order="1" />
<bond atomRefs2="a15 a13" order="1" />
<bond atomRefs2="a4 a3" order="1" />
<bond atomRefs2="a16 a17" order="1" />
<bond atomRefs2="a16 a1" order="1" />
<bond atomRefs2="a3 a1" order="1" />
<bond atomRefs2="a3 a6" order="1" />
<bond atomRefs2="a10 a13" order="1" />
<bond atomRefs2="a10 a9" order="1" />
<bond atomRefs2="a7 a6" order="1" />

<bond atomRefs2="a13 a14" order="1" />
<bond atomRefs2="a13 a20" order="1" />
<bond atomRefs2="a17 a21" order="1" />
<bond atomRefs2="a23 a24" order="1" />
<bond atomRefs2="a23 a25" order="1" />
<bond atomRefs2="a23 a20" order="1" />
<bond atomRefs2="a1 a2" order="2" />
<bond atomRefs2="a6 a9" order="1" />
<bond atomRefs2="a6 a8" order="1" />
<bond atomRefs2="a20 a22" order="1" />
<bond atomRefs2="a9 a19" order="1" />
<bond atomRefs2="a9 a18" order="1" />
</bondArray>
<propertyList>
<property title="program">
<scalar>Gaussian 16, Revision C.01</scalar>
</property>
<property title="basis">
<scalar>Aug-CC-pVTZ (5D, 7F)</scalar>
</property>
<property title="File Format">
<scalar>g03</scalar>
</property>
<property title="Energy" dictRef="me:ZPE">
<scalar units="kcal/mol" >1.87</scalar>
</property>
<property title="SpinMultiplicity" dictRef="me:spinMultiplicity">
<scalar>2.00</scalar>
</property>
<property title="Vibrational Frequencies" dictRef="me:vibFreqs">
<array units="cm-1">27.81 39.12 43.33 61.80 114.07 127.65 150.77 173.02 227.20
239.57 281.32 338.05 359.00 391.09 446.16 455.19 503.22 546.39 641.20 723.47 763.48 834.13
838.19 889.50 912.97 941.32 962.51 993.22 1009.54 1017.82 1046.97 1090.39 1101.14 1129.05
1159.22 1171.90 1199.17 1238.94 1281.80 1294.58 1331.48 1342.92 1366.41 1388.23 1394.69
1397.89 1416.69 1424.43 1476.87 1485.12 1488.03 1489.90 1496.35 1499.52 1508.47 1540.25
1823.56 2990.58 2994.45 3034.17 3041.36 3047.78 3056.13 3066.23 3076.41 3085.64 3092.64
3106.81 3116.23 3142.23 3196.71 3558.70</array>
</property>
<property title="Rotational Constants" dictRef="me:rotConsts">
<array units="cm-1">0.044 0.025 0.018</array>
</property>
<property title="Symmetry Number" dictRef="me:symmetryNumber">
<scalar>1</scalar>
</property>
</propertyList>
</molecule>

<molecule id="19Hshift-TS" spinMultiplicity="2">
<atomArray>
<atom id="a1" elementType="C" x3="-1.353688" y3="-1.166166" z3="0.302773" />
<atom id="a2" elementType="O" x3="-1.661325" y3="-1.559966" z3="1.387722" />
<atom id="a3" elementType="C" x3="-2.089789" y3="-0.099842" z3="-0.462901" />
<atom id="a4" elementType="H" x3="-1.912878" y3="-0.192837" z3="-1.530460" />
<atom id="a5" elementType="H" x3="-3.138783" y3="-0.313701" z3="-0.261350" />
<atom id="a6" elementType="C" x3="-1.791219" y3="1.334391" z3="0.009071" />
<atom id="a7" elementType="H" x3="-1.760106" y3="1.347972" z3="1.101039" />
<atom id="a8" elementType="H" x3="-2.659353" y3="1.930958" z3="-0.269477" />
<atom id="a9" elementType="C" x3="-0.532138" y3="2.003389" z3="-0.574019" />
<atom id="a10" elementType="C" x3="0.635049" y3="2.166293" z3="0.400108" />
<atom id="a11" elementType="H" x3="0.277362" y3="2.694353" z3="1.287404" />
<atom id="a12" elementType="H" x3="1.382784" y3="2.818066" z3="-0.060039" />
<atom id="a13" elementType="C" x3="1.327524" y3="0.881350" z3="0.842563" />
<atom id="a14" elementType="H" x3="2.075211" y3="1.132723" z3="1.604505" />
<atom id="a15" elementType="H" x3="0.628714" y3="0.210589" z3="1.352068" />
<atom id="a16" elementType="O" x3="-0.268424" y3="-1.785109" z3="-0.276000" />
<atom id="a17" elementType="O" x3="0.207468" y3="-1.150702" z3="-1.410935" />
<atom id="a18" elementType="H" x3="-0.804588" y3="3.002109" z3="-0.917821" />
<atom id="a19" elementType="H" x3="-0.204775" y3="1.463371" z3="-1.464088" />
<atom id="a20" elementType="C" spinMultiplicity="2" x3="2.038751" y3="0.134115" z3="-0.256428" />
<atom id="a21" elementType="H" x3="1.122692" y3="-0.441209" z3="-0.949171" />
<atom id="a22" elementType="H" x3="2.496206" y3="0.784583" z3="-1.004313" />
<atom id="a23" elementType="C" x3="2.909542" y3="-1.013340" z3="0.175404" />
<atom id="a24" elementType="H" x3="3.311476" y3="-1.556037" z3="-0.678381" />
<atom id="a25" elementType="H" x3="3.751544" y3="-0.643238" z3="0.768273" />
<atom id="a26" elementType="H" x3="2.348557" y3="-1.712624" z3="0.796088" />
</atomArray>
<bondArray>
<bond atomRefs2="a4 a3" order="1" />
<bond atomRefs2="a19 a9" order="1" />
<bond atomRefs2="a17 a21" order="1" />
<bond atomRefs2="a17 a16" order="1" />
<bond atomRefs2="a22 a20" order="1" />
<bond atomRefs2="a18 a9" order="1" />
<bond atomRefs2="a24 a23" order="1" />
<bond atomRefs2="a9 a6" order="1" />
<bond atomRefs2="a9 a10" order="1" />
<bond atomRefs2="a3 a5" order="1" />
<bond atomRefs2="a3 a6" order="1" />
<bond atomRefs2="a3 a1" order="1" />
<bond atomRefs2="a16 a1" order="1" />
<bond atomRefs2="a8 a6" order="1" />
<bond atomRefs2="a20 a23" order="1" />

<bond atomRefs2="a20 a13" order="1" />
<bond atomRefs2="a12 a10" order="1" />
<bond atomRefs2="a6 a7" order="1" />
<bond atomRefs2="a23 a25" order="1" />
<bond atomRefs2="a23 a26" order="1" />
<bond atomRefs2="a1 a2" order="2" />
<bond atomRefs2="a10 a13" order="1" />
<bond atomRefs2="a10 a11" order="1" />
<bond atomRefs2="a13 a15" order="1" />
<bond atomRefs2="a13 a14" order="1" />
</bondArray>
<propertyList>
<property title="program">
<scalar>Gaussian 16, Revision C.01</scalar>
</property>
<property title="basis">
<scalar>Aug-CC-pVTZ (5D, 7F)</scalar>
</property>
<property title="File Format">
<scalar>g03</scalar>
</property>
<property title="Energy" dictRef="me:ZPE">
<scalar units="kcal/mol" >19.85</scalar>
</property>
<property title="SpinMultiplicity" dictRef="me:spinMultiplicity">
<scalar>2.00</scalar>
</property>
<property title="Vibrational Frequencies" dictRef="me:vibFreqs">
<array units="cm-1">51.06 74.07 128.55 149.87 180.55 192.20 207.19 248.68 292.58 316.00 365.67 414.79 424.11 490.11 515.01 555.67 600.03 699.07 742.23 772.15 825.33 845.54 888.58 892.71 950.37 970.28 1000.52 1045.09 1060.79 1082.76 1094.65 1119.15 1129.87 1132.41 1166.34 1174.31 1234.44 1247.47 1268.64 1307.90 1318.65 1341.96 1376.79 1390.34 1403.67 1405.03 1413.47 1421.12 1457.08 1483.66 1486.45 1489.21 1496.58 1498.84 1504.79 1510.97 1860.85 3011.44 3030.43 3037.46 3055.80 3057.22 3060.27 3077.76 3083.95 3089.03 3099.12 3104.28 3108.02 3140.17 3157.49</array>
</property>
<property title="ImaginaryFrequency" dictRef="me:imFreqs">
<scalar units="cm-1">1709.37</scalar>
</property>
<property title="Rotational Constants" dictRef="me:rotConsts">
<array units="cm-1">0.041 0.033 0.023</array>
</property>
<property title="Symmetry Number" dictRef="me:symmetryNumber">
<scalar>1</scalar>
</property>
<property dictRef="me:frequenciesScaleFactor" >

```
<scalar>1</scalar>
</property>
</propertyList>
<me:DOSCMethod name="ClassicalRotors" />
</molecule>
<molecule id="Octanal-14-rad" spinMultiplicity="2">
<atomArray>
<atom id="a1" elementType="C" x3="1.850074" y3="-0.116100" z3="0.214268" />
<atom id="a2" elementType="O" x3="1.349135" y3="-0.879862" z3="1.017856" />
<atom id="a3" elementType="C" spinMultiplicity="2" x3="1.526817" y3="1.266073"
z3="0.003102" />
<atom id="a4" elementType="O" x3="2.832891" y3="-0.528415" z3="-0.623344" />
<atom id="a5" elementType="O" x3="3.138749" y3="-1.887103" z3="-0.409883" />
<atom id="a6" elementType="H" x3="2.061672" y3="1.790213" z3="-0.777907" />
<atom id="a7" elementType="H" x3="2.514259" y3="-2.106447" z3="0.313000" />
<atom id="a8" elementType="C" x3="0.458558" y3="1.947118" z3="0.764771" />
<atom id="a9" elementType="H" x3="0.128738" y3="1.308713" z3="1.582665" />
<atom id="a10" elementType="H" x3="0.868866" y3="2.860752" z3="1.205260" />
<atom id="a11" elementType="C" x3="-0.735850" y3="2.342585" z3="-0.126759" />
<atom id="a12" elementType="H" x3="-1.456433" y3="2.877141" z3="0.496302" />
<atom id="a13" elementType="H" x3="-0.391164" y3="3.050423" z3="-0.884046" />
<atom id="a14" elementType="C" x3="-1.423255" y3="1.163640" z3="-0.810816" />
<atom id="a15" elementType="H" x3="-2.202913" y3="1.553890" z3="-1.470047" />
<atom id="a16" elementType="H" x3="-0.707225" y3="0.651009" z3="-1.461574" />
<atom id="a17" elementType="C" x3="-2.040659" y3="0.152053" z3="0.147943" />
<atom id="a18" elementType="H" x3="-1.264198" y3="-0.286065" z3="0.779568" />
<atom id="a19" elementType="H" x3="-2.734628" y3="0.668585" z3="0.819724" />
<atom id="a20" elementType="C" x3="-2.776700" y3="-0.971961" z3="-0.571018" />
<atom id="a21" elementType="H" x3="-3.563198" y3="-0.546221" z3="-1.200492" />
<atom id="a22" elementType="H" x3="-2.081967" y3="-1.476170" z3="-1.248301" />
<atom id="a23" elementType="C" x3="-3.380969" y3="-1.989115" z3="0.387598" />
<atom id="a24" elementType="H" x3="-4.099162" y3="-1.514796" z3="1.058946" />
<atom id="a25" elementType="H" x3="-3.899875" y3="-2.783888" z3="-0.148258" />
<atom id="a26" elementType="H" x3="-2.607081" y3="-2.449848" z3="1.003597" />
</atomArray>
<bondArray>
<bond atomRefs2="a15 a14" order="1" />
<bond atomRefs2="a16 a14" order="1" />
<bond atomRefs2="a22 a20" order="1" />
<bond atomRefs2="a21 a20" order="1" />
<bond atomRefs2="a13 a11" order="1" />
<bond atomRefs2="a14 a11" order="1" />
<bond atomRefs2="a14 a17" order="1" />
<bond atomRefs2="a6 a3" order="1" />
<bond atomRefs2="a4 a5" order="1" />
<bond atomRefs2="a4 a1" order="1" />
```

<bond atomRefs2="a20 a17" order="1" />
<bond atomRefs2="a20 a23" order="1" />
<bond atomRefs2="a5 a7" order="1" />
<bond atomRefs2="a25 a23" order="1" />
<bond atomRefs2="a11 a12" order="1" />
<bond atomRefs2="a11 a8" order="1" />
<bond atomRefs2="a3 a1" order="1" />
<bond atomRefs2="a3 a8" order="1" />
<bond atomRefs2="a17 a18" order="1" />
<bond atomRefs2="a17 a19" order="1" />
<bond atomRefs2="a1 a2" order="2" />
<bond atomRefs2="a23 a26" order="1" />
<bond atomRefs2="a23 a24" order="1" />
<bond atomRefs2="a8 a10" order="1" />
<bond atomRefs2="a8 a9" order="1" />
</bondArray>
<propertyList>
<property title="program">
<scalar>Gaussian 16, Revision C.01</scalar>
</property>
<property title="basis">
<scalar>Aug-CC-pVTZ (5D, 7F)</scalar>
</property>
<property title="File Format">
<scalar>g03</scalar>
</property>
<property title="Energy" dictRef="me:ZPE">
<scalar units="kcal/mol" >-2.33</scalar>
</property>
<property title="SpinMultiplicity" dictRef="me:spinMultiplicity">
<scalar>2.00</scalar>
</property>
<property title="Vibrational Frequencies" dictRef="me:vibFreqs">
<array units="cm-1">25.02 28.86 60.70 79.13 113.73 134.65 183.80 201.17 237.74
254.50 287.47 364.57 384.37 405.86 450.68 494.44 500.54 621.72 711.71 739.31 769.12 776.13
841.35 868.93 905.83 933.28 970.17 996.48 1019.22 1059.68 1075.93 1089.99 1116.54 1127.67
1160.32 1191.65 1232.53 1245.06 1283.60 1308.19 1334.44 1348.88 1357.41 1387.14 1392.05
1421.68 1426.51 1464.49 1489.41 1494.89 1496.34 1503.86 1505.48 1506.34 1516.21 1522.78
1746.96 3021.03 3028.09 3035.04 3036.01 3042.02 3047.10 3060.58 3067.46 3086.49 3092.38
3112.37 3120.18 3126.96 3203.86 3526.83</array>
</property>
<property title="Rotational Constants" dictRef="me:rotConsts">
<array units="cm-1">0.042 0.020 0.015</array>
</property>
<property title="Symmetry Number" dictRef="me:symmetryNumber">
<scalar>1</scalar>

</property>
</propertyList>
</molecule>
<molecule id="14Hshift-TS" spinMultiplicity="2">
<atomArray>
<atom id="a1" elementType="C" x3="1.992776" y3="0.933592" z3="0.152956" />
<atom id="a2" elementType="O" x3="1.453943" y3="1.992868" z3="0.189768" />
<atom id="a3" elementType="C" x3="1.735979" y3="-0.257976" z3="1.040675" />
<atom id="a4" elementType="O" x3="2.956494" y3="0.645556" z3="-0.756197" />
<atom id="a5" elementType="O" spinMultiplicity="2" x3="3.369520" y3="-0.687238" z3="-0.578638" />
<atom id="a6" elementType="H" x3="2.167999" y3="-0.126887" z3="2.030737" />
<atom id="a7" elementType="H" x3="2.575650" y3="-0.954198" z3="0.384086" />
<atom id="a8" elementType="C" x3="0.341615" y3="-0.817833" z3="1.020066" />
<atom id="a9" elementType="H" x3="0.294041" y3="-1.685282" z3="1.681335" />
<atom id="a10" elementType="H" x3="-0.311122" y3="-0.058142" z3="1.464338" />
<atom id="a11" elementType="C" x3="-0.153691" y3="-1.181064" z3="-0.376784" />
<atom id="a12" elementType="H" x3="-0.087306" y3="-0.302296" z3="-1.022734" />
<atom id="a13" elementType="H" x3="0.517204" y3="-1.926379" z3="-0.808936" />
<atom id="a14" elementType="C" x3="-1.581892" y3="-1.722209" z3="-0.393172" />
<atom id="a15" elementType="H" x3="-1.841515" y3="-1.965610" z3="-1.426223" />
<atom id="a16" elementType="H" x3="-1.613457" y3="-2.664733" z3="0.160284" />
<atom id="a17" elementType="C" x3="-2.641165" y3="-0.776698" z3="0.173667" />
<atom id="a18" elementType="H" x3="-3.619032" y3="-1.253765" z3="0.068407" />
<atom id="a19" elementType="H" x3="-2.492659" y3="-0.646744" z3="1.250073" />
<atom id="a20" elementType="C" x3="-2.683320" y3="0.594267" z3="-0.493343" />
<atom id="a21" elementType="H" x3="-1.740990" y3="1.121244" z3="-0.324848" />
<atom id="a22" elementType="H" x3="-2.770418" y3="0.464112" z3="-1.575873" />
<atom id="a23" elementType="C" x3="-3.832133" y3="1.455125" z3="0.014717" />
<atom id="a24" elementType="H" x3="-4.794776" y3="0.978417" z3="-0.178401" />
<atom id="a25" elementType="H" x3="-3.752686" y3="1.614572" z3="1.091653" />
<atom id="a26" elementType="H" x3="-3.839603" y3="2.432970" z3="-0.466049" />
</atomArray>
<bondArray>
<bond atomRefs2="a22 a20" order="1" />
<bond atomRefs2="a15 a14" order="1" />
<bond atomRefs2="a12 a11" order="1" />
<bond atomRefs2="a13 a11" order="1" />
<bond atomRefs2="a4 a5" order="1" />
<bond atomRefs2="a4 a1" order="1" />
<bond atomRefs2="a20 a21" order="1" />
<bond atomRefs2="a20 a23" order="1" />
<bond atomRefs2="a20 a17" order="1" />
<bond atomRefs2="a26 a23" order="1" />
<bond atomRefs2="a14 a11" order="1" />
<bond atomRefs2="a14 a16" order="1" />

<bond atomRefs2="a14 a17" order="1" />
<bond atomRefs2="a11 a8" order="1" />
<bond atomRefs2="a24 a23" order="1" />
<bond atomRefs2="a23 a25" order="1" />
<bond atomRefs2="a18 a17" order="1" />
<bond atomRefs2="a1 a2" order="2" />
<bond atomRefs2="a1 a3" order="1" />
<bond atomRefs2="a17 a19" order="1" />
<bond atomRefs2="a7 a3" order="1" />
<bond atomRefs2="a8 a3" order="1" />
<bond atomRefs2="a8 a10" order="1" />
<bond atomRefs2="a8 a9" order="1" />
<bond atomRefs2="a3 a6" order="1" />
</bondArray>
<propertyList>
<property title="program">
<scalar>Gaussian 16, Revision C.01</scalar>
</property>
<property title="basis">
<scalar>Aug-CC-pVTZ (5D, 7F)</scalar>
</property>
<property title="File Format">
<scalar>g03</scalar>
</property>
<property title="Energy" dictRef="me:ZPE">
<scalar units="kcal/mol" >26.94</scalar>
</property>
<property title="SpinMultiplicity" dictRef="me:spinMultiplicity">
<scalar>2.00</scalar>
</property>
<property title="Vibrational Frequencies" dictRef="me:vibFreqs">
<array units="cm-1">22.63 40.57 59.93 83.31 106.19 181.89 197.82 205.69 222.49
249.53 294.04 338.07 442.45 506.83 526.31 568.75 661.47 689.23 726.82 763.94 793.88 837.81
858.46 882.74 930.68 958.46 1016.90 1021.13 1042.55 1056.37 1080.25 1119.30 1134.89
1149.69 1170.79 1184.52 1218.46 1254.64 1298.97 1314.59 1335.95 1338.55 1349.38 1360.65
1401.53 1413.71 1421.57 1424.25 1467.78 1492.43 1498.86 1503.19 1505.49 1506.62 1516.80
1766.20 1912.45 3027.16 3030.47 3037.18 3039.79 3042.30 3050.98 3060.31 3074.78 3079.32
3085.18 3098.96 3111.45 3122.79 3135.06</array>
</property>
<property title="ImaginaryFrequency" dictRef="me:imFreqs">
<scalar units="cm-1">1618.87</scalar>
</property>
<property title="Rotational Constants" dictRef="me:rotConsts">
<array units="cm-1">0.059 0.017 0.015</array>
</property>
<property title="Symmetry Number" dictRef="me:symmetryNumber">

```
        <scalar>1</scalar>
      </property>
      <property dictRef="me:frequenciesScaleFactor" >
        <scalar>1</scalar>
      </property>
    </propertyList>
    <me:DOSCMethod name="ClassicalRotors" />
  </molecule>
</moleculeList>
<reactionList>
 <reaction id="R1">
    <reactantList>
      <reactant>
        <molecule ref="Octanal-AcylOO-rad" role="modelled" />
      </reactant>
    </reactantList>
    <productList>
      <product>
        <molecule ref="Octanal-14-rad" role="sink" />
      </product>
    </productList>
    <me:transitionState>
      <molecule ref="14Hshift-TS" role="transitionState" />
    </me:transitionState>
    <me:MCRCMethod name="RRKM" />
          <me:tunneling name="Eckart" />
  </reaction>
  <reaction id="R2">
    <reactantList>
      <reactant>
        <molecule ref="Octanal-AcylOO-rad" role="modelled" />
      </reactant>
    </reactantList>
    <productList>
      <product>
        <molecule ref="Octanal-15-rad" role="sink" />
      </product>
    </productList>
    <me:transitionState>
      <molecule ref="15Hshift-TS" role="transitionState" />
    </me:transitionState>
    <me:MCRCMethod name="RRKM" />
          <me:tunneling name="Eckart" />
  </reaction>
  <reaction id="R3">
    <reactantList>
```

```
    <reactant>
      <molecule ref="Octanal-AcylOO-rad" role="modelled" />
    </reactant>
  </reactantList>
  <productList>
    <product>
      <molecule ref="Octanal-16-rad" role="sink" />
    </product>
  </productList>
  <me:transitionState>
    <molecule ref="16Hshift-TS" role="transitionState" />
  </me:transitionState>
  <me:MCRCMethod name="RRKM" />
        <me:tunneling name="Eckart" />
</reaction>
<reaction id="R4">
  <reactantList>
    <reactant>
      <molecule ref="Octanal-AcylOO-rad" role="modelled" />
    </reactant>
  </reactantList>
  <productList>
    <product>
      <molecule ref="Octanal-17-rad" role="sink" />
    </product>
  </productList>
  <me:transitionState>
    <molecule ref="17Hshift-TS" role="transitionState" />
  </me:transitionState>
  <me:MCRCMethod name="RRKM" />
        <me:tunneling name="Eckart" />
</reaction>
<reaction id="R5">
  <reactantList>
    <reactant>
      <molecule ref="Octanal-AcylOO-rad" role="modelled" />
    </reactant>
  </reactantList>
  <productList>
    <product>
      <molecule ref="Octanal-18-rad" role="sink" />
    </product>
  </productList>
  <me:transitionState>
    <molecule ref="18Hshift-TS" role="transitionState" />
  </me:transitionState>
```

```
    <me:MCRCMethod name="RRKM" />
        <me:tunneling name="Eckart" />
  </reaction>
        <reaction id="R6">
    <reactantList>
      <reactant>
        <molecule ref="Octanal-AcylOO-rad" role="modelled" />
      </reactant>
    </reactantList>
    <productList>
      <product>
        <molecule ref="Octanal-19-rad" role="sink" />
      </product>
    </productList>
    <me:transitionState>
      <molecule ref="19Hshift-TS" role="transitionState" />
    </me:transitionState>
    <me:MCRCMethod name="RRKM" />
        <me:tunneling name="Eckart" />
  </reaction>
</reactionList>
<me:conditions>
  <me:bathGas>N2</me:bathGas>
  <me:PTs>
    <me:PTpair units="Torr" P="760." T="298." precision="qd"  bathGas="N2" />
  </me:PTs>
</me:conditions>
<me:modelParameters>
  <!--Specify grain size directly...-->
  <me:grainSize units="cm-1">100</me:grainSize>
  <!--...or by the total number of grains
      <me:numberOfGrains> 500 </me:numberOfGrains>-->
  <!--Specify increased energy range
      <me:maxTemperature>6000</me:maxTemperature>-->
  <me:energyAboveTheTopHill>25.0</me:energyAboveTheTopHill>
</me:modelParameters>
<me:control>
  <me:printSpeciesProfile />
  <!--<me:testMicroRates />
        <me:testDOS />
  <me:testRateConstant />
  <me:printGrainDOS />-->
  <!--<me:printCellDOS />-->
  <!--<me:printReactionOperatorColumnSums />-->
  <!--<me:printTunnellingCoefficients />
  <me:printGrainkfE />-->
```

```xml
    <!--<me:printGrainBoltzmann />
    <me:printGrainkbE />-->
    <me:eigenvalues>3</me:eigenvalues>
    <!-- <me:hideInactive/>  Molecules and reactions with attribute active="false" are not shown
    <me:diagramEnergyOffset>0</me:diagramEnergyOffset>-->
    <!--Adjusts displayed energies to this values for the lowest species. -->
    <me:calcMethod  name="simpleCalc" />
    <me:ForceMacroDetailedBalance >true</me:ForceMacroDetailedBalance>
  </me:control>
  <metadataList xmlns:dc="http://purl.org/dc/elements/1.1/">
    <dc:title>Project name</dc:title>
    <dc:source>Acylperoxyrad-Octanal.xml</dc:source>
    <dc:creator>Mesmer v6.0</dc:creator>
    <dc:date>20220513_153203</dc:date>
    <dc:contributor>ndprse</dc:contributor>
  </metadataList>
</me:mesmer>
```

Nonanal–AcOO ------> Nonanal–AcOOH


```
<?xml version="1.0" encoding="utf-8" ?>
<?xml-stylesheet type='text/xsl' href='../../mesmer2.xsl' media='other'?>
<?xml-stylesheet type='text/xsl' href='../../mesmer1.xsl' media='screen'?>
<me:mesmer xmlns="http://www.xml-cml.org/schema"
xmlns:me="http://www.chem.leeds.ac.uk/mesmer"
xmlns:xsi="http://www.w3.org/2001/XMLSchema-instance">
  <me:title>Project name</me:title>
  <moleculeList convention="">
    <molecule id="N2">
    <atomArray>
      <atom id="a1" elementType="N" />
      <atom id="a2" elementType="N" />
    </atomArray>
    <bondArray>
      <bond atomRefs2="a2 a1" order="3"/>
    </bondArray>
    <propertyList>
      <property dictRef="me:epsilon">
        <scalar>48.0</scalar>
      </property>
      <property dictRef="me:sigma">
        <scalar>3.90</scalar>
      </property>
      <property dictRef="me:MW">
        <scalar units="amu">28.0</scalar>
      </property>
    </propertyList>
  </molecule>
    <molecule id="Nonanal-15-rad" spinMultiplicity="2">
      <atomArray>
        <atom id="a1" elementType="C" x3="1.119867" y3="-1.447801" z3="0.322660" />
        <atom id="a2" elementType="O" x3="0.550674" y3="-1.524231" z3="1.381118" />
        <atom id="a3" elementType="C" x3="2.466129" y3="-0.815027" z3="0.054752" />
        <atom id="a4" elementType="H" x3="3.161132" y3="-1.240971" z3="0.776944" />
        <atom id="a5" elementType="H" x3="2.786951" y3="-1.096897" z3="-0.950227" />
        <atom id="a6" elementType="C" spinMultiplicity="2" x3="2.380190" y3="0.667710"
z3="0.200945" />
        <atom id="a7" elementType="C" x3="1.716163" y3="1.528113" z3="-0.813196" />
        <atom id="a8" elementType="H" x3="2.476697" y3="2.075906" z3="-1.385327" />
        <atom id="a9" elementType="H" x3="1.190465" y3="0.907884" z3="-1.543566" />
        <atom id="a10" elementType="C" x3="0.755170" y3="2.552585" z3="-0.194043" />
        <atom id="a11" elementType="H" x3="0.360080" y3="3.190926" z3="-0.988369" />
        <atom id="a12" elementType="H" x3="1.328095" y3="3.204235" z3="0.470629" />
        <atom id="a13" elementType="O" x3="0.570439" y3="-1.913182" z3="-0.811555" />
```

<atom id="a14" elementType="H" x3="2.708891" y3="1.110840" z3="1.131553" />
<atom id="a15" elementType="C" x3="-0.399796" y3="1.930893" z3="0.587889" />
<atom id="a16" elementType="H" x3="0.000457" y3="1.268779" z3="1.361787" />
<atom id="a17" elementType="H" x3="-0.935831" y3="2.724854" z3="1.114220" />
<atom id="a18" elementType="C" x3="-1.393035" y3="1.154423" z3="-0.268857" />
<atom id="a19" elementType="H" x3="-1.821862" y3="1.824195" z3="-1.021738" />
<atom id="a20" elementType="H" x3="-0.882829" y3="0.363812" z3="-0.825526" />
<atom id="a21" elementType="C" x3="-2.514901" y3="0.525005" z3="0.547844" />
<atom id="a22" elementType="H" x3="-3.022984" y3="1.304058" z3="1.123125" />
<atom id="a23" elementType="H" x3="-2.075995" y3="-0.156596" z3="1.282123" />
<atom id="a24" elementType="C" x3="-3.526311" y3="-0.221625" z3="-0.311570" />
<atom id="a25" elementType="H" x3="-4.310570" y3="-0.673521" z3="0.296169" />
<atom id="a26" elementType="H" x3="-4.004612" y3="0.452492" z3="-1.024584" />
<atom id="a27" elementType="H" x3="-3.039857" y3="-1.014957" z3="-0.880977" />
<atom id="a28" elementType="O" x3="-0.712462" y3="-2.460359" z3="-0.579317" />
<atom id="a29" elementType="H" x3="-0.808292" y3="-2.308525" z3="0.383244" />
</atomArray>
<bondArray>
<bond atomRefs2="a9 a7" order="1" />
<bond atomRefs2="a8 a7" order="1" />
<bond atomRefs2="a26 a24" order="1" />
<bond atomRefs2="a19 a18" order="1" />
<bond atomRefs2="a11 a10" order="1" />
<bond atomRefs2="a5 a3" order="1" />
<bond atomRefs2="a27 a24" order="1" />
<bond atomRefs2="a20 a18" order="1" />
<bond atomRefs2="a7 a10" order="1" />
<bond atomRefs2="a7 a6" order="1" />
<bond atomRefs2="a13 a28" order="1" />
<bond atomRefs2="a13 a1" order="1" />
<bond atomRefs2="a28 a29" order="1" />
<bond atomRefs2="a24 a25" order="1" />
<bond atomRefs2="a24 a21" order="1" />
<bond atomRefs2="a18 a21" order="1" />
<bond atomRefs2="a18 a15" order="1" />
<bond atomRefs2="a10 a12" order="1" />
<bond atomRefs2="a10 a15" order="1" />
<bond atomRefs2="a3 a6" order="1" />
<bond atomRefs2="a3 a1" order="1" />
<bond atomRefs2="a3 a4" order="1" />
<bond atomRefs2="a6 a14" order="1" />
<bond atomRefs2="a1 a2" order="2" />
<bond atomRefs2="a21 a22" order="1" />
<bond atomRefs2="a21 a23" order="1" />
<bond atomRefs2="a15 a17" order="1" />
<bond atomRefs2="a15 a16" order="1" />

</bondArray>
<propertyList>
<property title="program">
<scalar>Gaussian 16, Revision C.01</scalar>
</property>
<property title="basis">
<scalar>Aug-CC-pVTZ (5D, 7F)</scalar>
</property>
<property title="File Format">
<scalar>g03</scalar>
</property>
<property title="Energy" dictRef="me:ZPE">
<scalar units="kcal/mol" >2.45</scalar>
</property>
<property title="SpinMultiplicity" dictRef="me:spinMultiplicity">
<scalar>2.00</scalar>
</property>
<property title="Vibrational Frequencies" dictRef="me:vibFreqs">
<array units="cm-1">27.14 36.37 65.37 77.82 123.13 132.01 148.38 169.49 225.51 235.23 254.28 274.56 317.36 348.76 404.54 413.92 432.61 449.64 481.51 512.03 641.61 736.41 771.32 786.74 829.79 848.92 893.09 915.06 926.50 962.84 983.09 1003.37 1030.57 1064.33 1079.15 1096.56 1118.86 1129.09 1152.81 1179.99 1221.73 1240.57 1280.96 1298.45 1309.16 1325.01 1335.30 1341.45 1346.49 1387.96 1394.05 1419.84 1422.08 1426.16 1470.39 1485.22 1492.84 1497.56 1500.66 1504.19 1505.33 1511.97 1520.81 1817.70 2985.94 3024.12 3032.89 3036.83 3040.70 3041.93 3057.86 3059.27 3065.53 3070.39 3084.64 3088.24 3112.43 3120.74 3128.59 3195.10 3554.66</array>
</property>
<property title="Rotational Constants" dictRef="me:rotConsts">
<array units="cm-1">0.031 0.026 0.017</array>
</property>
<property title="Symmetry Number" dictRef="me:symmetryNumber">
<scalar>1</scalar>
</property>
</propertyList>
</molecule>
<molecule id="15Hshift-TS" spinMultiplicity="2">
<atomArray>
<atom id="a1" elementType="C" x3="-3.435362" y3="-0.192371" z3="-0.543637" />
<atom id="a2" elementType="O" x3="-4.024696" y3="-0.760036" z3="-1.408268" />
<atom id="a3" elementType="C" x3="-2.989195" y3="-0.783240" z3="0.779758" />
<atom id="a4" elementType="H" x3="-3.727218" y3="-0.502376" z3="1.532070" />
<atom id="a5" elementType="H" x3="-3.023384" y3="-1.865234" z3="0.653856" />
<atom id="a6" elementType="C" spinMultiplicity="2" x3="-1.621673" y3="-0.286520" z3="1.176138" />
<atom id="a7" elementType="C" x3="-0.449516" y3="-0.751866" z3="0.358780" />
<atom id="a8" elementType="H" x3="-0.323002" y3="-1.829641" z3="0.524780" />
<atom id="a9" elementType="H" x3="-0.674105" y3="-0.639312" z3="-0.706166" />

<atom id="a10" elementType="C" x3="0.849152" y3="-0.027272" z3="0.690851" />
<atom id="a11" elementType="H" x3="1.066394" y3="-0.143099" z3="1.757081" />
<atom id="a12" elementType="H" x3="0.713766" y3="1.043941" z3="0.517815" />
<atom id="a13" elementType="O" x3="-3.039369" y3="1.100124" z3="-0.773041" />
<atom id="a14" elementType="O" x3="-2.586784" y3="1.778728" z3="0.355238" />
<atom id="a15" elementType="H" x3="-1.446768" y3="-0.256389" z3="2.249912" />
<atom id="a16" elementType="H" x3="-1.828367" y3="0.948718" z3="0.884690" />
<atom id="a17" elementType="C" x3="2.033812" y3="-0.526919" z3="-0.125268" />
<atom id="a18" elementType="H" x3="2.165702" y3="-1.599975" z3="0.046114" />
<atom id="a19" elementType="H" x3="1.811205" y3="-0.413114" z3="-1.190561" />
<atom id="a20" elementType="C" x3="3.333083" y3="0.198822" z3="0.198233" />
<atom id="a21" elementType="H" x3="3.200013" y3="1.271742" z3="0.027519" />
<atom id="a22" elementType="H" x3="3.555944" y3="0.085737" z3="1.264111" />
<atom id="a23" elementType="C" x3="4.520408" y3="-0.296347" z3="-0.618416" />
<atom id="a24" elementType="H" x3="4.296267" y3="-0.181950" z3="-1.682396" />
<atom id="a25" elementType="H" x3="4.651348" y3="-1.368476" z3="-0.447888" />
<atom id="a26" elementType="C" x3="5.814141" y3="0.435475" z3="-0.287262" />
<atom id="a27" elementType="H" x3="6.075362" y3="0.310789" z3="0.765119" />
<atom id="a28" elementType="H" x3="6.647082" y3="0.063948" z3="-0.883949" />
<atom id="a29" elementType="H" x3="5.717450" y3="1.505587" z3="-0.478605" />
</atomArray>
<bondArray>
<bond atomRefs2="a24 a23" order="1" />
<bond atomRefs2="a2 a1" order="2" />
<bond atomRefs2="a19 a17" order="1" />
<bond atomRefs2="a28 a26" order="1" />
<bond atomRefs2="a13 a1" order="1" />
<bond atomRefs2="a13 a14" order="1" />
<bond atomRefs2="a9 a7" order="1" />
<bond atomRefs2="a23 a25" order="1" />
<bond atomRefs2="a23 a26" order="1" />
<bond atomRefs2="a23 a20" order="1" />
<bond atomRefs2="a1 a3" order="1" />
<bond atomRefs2="a29 a26" order="1" />
<bond atomRefs2="a26 a27" order="1" />
<bond atomRefs2="a17 a18" order="1" />
<bond atomRefs2="a17 a20" order="1" />
<bond atomRefs2="a17 a10" order="1" />
<bond atomRefs2="a21 a20" order="1" />
<bond atomRefs2="a20 a22" order="1" />
<bond atomRefs2="a14 a16" order="1" />
<bond atomRefs2="a7 a8" order="1" />
<bond atomRefs2="a7 a10" order="1" />
<bond atomRefs2="a7 a6" order="1" />
<bond atomRefs2="a12 a10" order="1" />
<bond atomRefs2="a5 a3" order="1" />

<bond atomRefs2="a10 a11" order="1" />
<bond atomRefs2="a3 a6" order="1" />
<bond atomRefs2="a3 a4" order="1" />
<bond atomRefs2="a6 a15" order="1" />
</bondArray>
<propertyList>
<property title="program">
<scalar>Gaussian 16, Revision C.01</scalar>
</property>
<property title="basis">
<scalar>Aug-CC-pVTZ (5D, 7F)</scalar>
</property>
<property title="File Format">
<scalar>g03</scalar>
</property>
<property title="Energy" dictRef="me:ZPE">
<scalar units="kcal/mol" >20.47</scalar>
</property>
<property title="SpinMultiplicity" dictRef="me:spinMultiplicity">
<scalar>2.00</scalar>
</property>
<property title="Vibrational Frequencies" dictRef="me:vibFreqs">
<array units="cm-1">26.95 37.57 52.43 75.42 98.00 113.57 144.64 164.33 194.77 235.15 244.05 284.87 332.33 431.94 471.12 483.89 520.18 555.33 590.52 704.29 732.22 740.51 773.65 794.62 864.05 894.70 916.08 940.94 1009.12 1021.88 1041.66 1061.22 1069.80 1076.50 1085.42 1090.11 1143.59 1159.99 1192.59 1221.00 1228.97 1238.31 1266.14 1285.96 1305.04 1321.48 1335.66 1343.82 1351.64 1359.39 1400.99 1421.19 1423.69 1429.44 1457.84 1474.69 1493.09 1493.78 1500.39 1505.67 1509.27 1516.84 1585.68 1873.69 3001.16 3021.02 3024.80 3032.50 3037.25 3042.13 3046.44 3053.26 3061.56 3075.20 3076.01 3086.90 3112.05 3120.31 3122.09 3130.48</array>
</property>
<property title="ImaginaryFrequency" dictRef="me:imFreqs">
<scalar units="cm-1">1683.96</scalar>
</property>
<property title="Rotational Constants" dictRef="me:rotConsts">
<array units="cm-1">0.075 0.009 0.009</array>
</property>
<property title="Symmetry Number" dictRef="me:symmetryNumber">
<scalar>1</scalar>
</property>
<property dictRef="me:frequenciesScaleFactor" >
<scalar>1</scalar>
</property>
</propertyList>
<me:DOSCMethod name="ClassicalRotors" />
</molecule>
<molecule id="Nonanal-17-rad" spinMultiplicity="2">

<atomArray>
<atom id="a1" elementType="C" x3="2.471112" y3="-1.185592" z3="0.346903" />
<atom id="a2" elementType="H" x3="3.464620" y3="-1.635138" z3="0.364805" />
<atom id="a3" elementType="H" x3="2.059207" y3="-1.282286" z3="1.352241" />
<atom id="a4" elementType="C" x3="1.594725" y3="-1.949650" z3="-0.661771" />
<atom id="a5" elementType="H" x3="1.634919" y3="-3.010750" z3="-0.387665" />
<atom id="a6" elementType="C" spinMultiplicity="2" x3="0.175778" y3="-1.503368" z3="-
0.710150" />
<atom id="a7" elementType="H" x3="-0.182602" y3="-0.960575" z3="-1.575222" />
<atom id="a8" elementType="C" x3="2.622147" y3="0.296194" z3="0.008066" />
<atom id="a9" elementType="H" x3="3.407334" y3="0.738960" z3="0.624310" />
<atom id="a10" elementType="H" x3="2.904270" y3="0.435061" z3="-1.035115" />
<atom id="a11" elementType="C" x3="1.384255" y3="1.092392" z3="0.300940" />
<atom id="a12" elementType="O" x3="0.758426" y3="1.101298" z3="1.331752" />
<atom id="a13" elementType="C" x3="-0.766313" y3="-1.828133" z3="0.392518" />
<atom id="a14" elementType="H" x3="-0.730623" y3="-2.909695" z3="0.581756" />
<atom id="a15" elementType="H" x3="-0.420345" y3="-1.364905" z3="1.327518" />
<atom id="a16" elementType="O" x3="1.054269" y3="1.884738" z3="-0.732454" />
<atom id="a17" elementType="H" x3="2.049053" y3="-1.863610" z3="-1.651233" />
<atom id="a18" elementType="O" x3="-0.068481" y3="2.688788" z3="-0.430916" />
<atom id="a19" elementType="C" x3="-2.209961" y3="-1.402265" z3="0.136910" />
<atom id="a20" elementType="H" x3="-2.850094" y3="-1.829131" z3="0.913271" />
<atom id="a21" elementType="H" x3="-2.549886" y3="-1.829746" z3="-0.811504" />
<atom id="a22" elementType="C" x3="-2.406545" y3="0.109100" z3="0.110628" />
<atom id="a23" elementType="H" x3="-1.768948" y3="0.551818" z3="-0.657865" />
<atom id="a24" elementType="H" x3="-2.066001" y3="0.523839" z3="1.063438" />
<atom id="a25" elementType="C" x3="-3.853297" y3="0.510853" z3="-0.140875" />
<atom id="a26" elementType="H" x3="-4.204396" y3="0.126017" z3="-1.100094" />
<atom id="a27" elementType="H" x3="-4.511266" y3="0.114034" z3="0.634592" />
<atom id="a28" elementType="H" x3="-3.968994" y3="1.594614" z3="-0.155377" />
<atom id="a29" elementType="H" x3="-0.291370" y3="2.365712" z3="0.466073" />
</atomArray>
<bondArray>
<bond atomRefs2="a17 a4" order="1" />
<bond atomRefs2="a7 a6" order="1" />
<bond atomRefs2="a26 a25" order="1" />
<bond atomRefs2="a10 a8" order="1" />
<bond atomRefs2="a21 a19" order="1" />
<bond atomRefs2="a16 a18" order="1" />
<bond atomRefs2="a16 a11" order="1" />
<bond atomRefs2="a6 a4" order="1" />
<bond atomRefs2="a6 a13" order="1" />
<bond atomRefs2="a4 a5" order="1" />
<bond atomRefs2="a4 a1" order="1" />
<bond atomRefs2="a23 a22" order="1" />
<bond atomRefs2="a18 a29" order="1" />

<bond atomRefs2="a28 a25" order="1" />
<bond atomRefs2="a25 a22" order="1" />
<bond atomRefs2="a25 a27" order="1" />
<bond atomRefs2="a8 a11" order="1" />
<bond atomRefs2="a8 a1" order="1" />
<bond atomRefs2="a8 a9" order="1" />
<bond atomRefs2="a22 a19" order="1" />
<bond atomRefs2="a22 a24" order="1" />
<bond atomRefs2="a19 a13" order="1" />
<bond atomRefs2="a19 a20" order="1" />
<bond atomRefs2="a11 a12" order="2" />
<bond atomRefs2="a1 a2" order="1" />
<bond atomRefs2="a1 a3" order="1" />
<bond atomRefs2="a13 a14" order="1" />
<bond atomRefs2="a13 a15" order="1" />
</bondArray>
<propertyList>
<property title="program">
<scalar>Gaussian 16, Revision C.01</scalar>
</property>
<property title="basis">
<scalar>Aug-CC-pVTZ (5D, 7F)</scalar>
</property>
<property title="File Format">
<scalar>g03</scalar>
</property>
<property title="Energy" dictRef="me:ZPE">
<scalar units="kcal/mol" >1.38</scalar>
</property>
<property title="SpinMultiplicity" dictRef="me:spinMultiplicity">
<scalar>2.00</scalar>
</property>
<property title="Vibrational Frequencies" dictRef="me:vibFreqs">
<array units="cm-1">29.97 56.05 71.24 77.16 103.30 105.92 149.29 182.10 230.17 242.04 253.39 260.05 299.57 311.50 353.54 371.85 439.19 469.69 488.12 563.72 618.25 724.96 758.18 815.01 820.93 864.78 878.21 931.40 940.27 943.97 985.18 1010.32 1042.59 1061.72 1073.65 1090.02 1104.48 1135.96 1158.75 1188.88 1227.47 1237.68 1249.70 1284.33 1303.19 1331.16 1339.54 1352.82 1366.48 1385.83 1404.14 1413.86 1421.11 1436.36 1468.85 1476.16 1487.88 1496.42 1497.24 1503.48 1505.49 1522.11 1528.95 1815.30 2977.37 2995.28 3004.38 3031.34 3039.92 3043.33 3064.50 3064.77 3070.23 3076.28 3086.14 3111.27 3111.73 3118.80 3126.59 3188.73 3564.07</array>
</property>
<property title="Rotational Constants" dictRef="me:rotConsts">
<array units="cm-1">0.035 0.023 0.016</array>
</property>
<property title="Symmetry Number" dictRef="me:symmetryNumber">
<scalar>1</scalar>

```
      </property>
    </propertyList>
  </molecule>
  <molecule id="17Hshift-TS" spinMultiplicity="2">
    <atomArray>
      <atom id="a1" elementType="C" x3="1.592546" y3="1.614252" z3="0.513054" />
      <atom id="a2" elementType="H" x3="0.897147" y3="2.167906" z3="1.144925" />
      <atom id="a3" elementType="H" x3="2.503791" y3="2.209475" z3="0.454077" />
      <atom id="a4" elementType="C" x3="1.025678" y3="1.475982" z3="-0.902537" />
      <atom id="a5" elementType="H" x3="1.802967" y3="1.101951" z3="-1.573140" />
      <atom id="a6" elementType="C" spinMultiplicity="2" x3="-0.199689" y3="0.607590" z3="-1.043742" />
      <atom id="a7" elementType="H" x3="-0.487367" y3="0.452728" z3="-2.084164" />
      <atom id="a8" elementType="C" x3="1.921255" y3="0.286245" z3="1.222662" />
      <atom id="a9" elementType="H" x3="2.527907" y3="0.490746" z3="2.101412" />
      <atom id="a10" elementType="H" x3="1.014652" y3="-0.224927" z3="1.531741" />
      <atom id="a11" elementType="C" x3="2.747222" y3="-0.599064" z3="0.329453" />
      <atom id="a12" elementType="O" x3="3.935009" y3="-0.559424" z3="0.222034" />
      <atom id="a13" elementType="C" x3="-1.373180" y3="0.863297" z3="-0.135889" />
      <atom id="a14" elementType="H" x3="-1.057489" y3="0.816862" z3="0.909886" />
      <atom id="a15" elementType="H" x3="-1.714625" y3="1.894312" z3="-0.298589" />
      <atom id="a16" elementType="O" x3="2.088514" y3="-1.468451" z3="-0.512512" />
      <atom id="a17" elementType="H" x3="0.778647" y3="2.478136" z3="-1.271536" />
      <atom id="a18" elementType="O" x3="0.729925" y3="-1.601263" z3="-0.269903" />
      <atom id="a19" elementType="H" x3="0.214680" y3="-0.581077" z3="-0.731671" />
      <atom id="a20" elementType="C" x3="-2.534256" y3="-0.098460" z3="-0.358360" />
      <atom id="a21" elementType="H" x3="-2.185910" y3="-1.124093" z3="-0.207479" />
      <atom id="a22" elementType="H" x3="-2.860205" y3="-0.035982" z3="-1.400991" />
      <atom id="a23" elementType="C" x3="-3.719210" y3="0.173346" z3="0.559547" />
      <atom id="a24" elementType="H" x3="-4.061940" y3="1.201073" z3="0.410460" />
      <atom id="a25" elementType="H" x3="-3.388903" y3="0.109122" z3="1.599892" />
      <atom id="a26" elementType="C" x3="-4.875887" y3="-0.790130" z3="0.330883" />
      <atom id="a27" elementType="H" x3="-5.243782" y3="-0.722713" z3="-0.694323" />
      <atom id="a28" elementType="H" x3="-4.564283" y3="-1.821623" z3="0.502775" />
      <atom id="a29" elementType="H" x3="-5.709746" y3="-0.577136" z3="0.999351" />
    </atomArray>
    <bondArray>
      <bond atomRefs2="a7 a6" order="1" />
      <bond atomRefs2="a5 a4" order="1" />
      <bond atomRefs2="a22 a20" order="1" />
      <bond atomRefs2="a17 a4" order="1" />
      <bond atomRefs2="a6 a4" order="1" />
      <bond atomRefs2="a6 a13" order="1" />
      <bond atomRefs2="a4 a1" order="1" />
      <bond atomRefs2="a19 a18" order="1" />
      <bond atomRefs2="a27 a26" order="1" />
```

<bond atomRefs2="a16 a18" order="1" />
<bond atomRefs2="a16 a11" order="1" />
<bond atomRefs2="a20 a21" order="1" />
<bond atomRefs2="a20 a13" order="1" />
<bond atomRefs2="a20 a23" order="1" />
<bond atomRefs2="a15 a13" order="1" />
<bond atomRefs2="a13 a14" order="1" />
<bond atomRefs2="a12 a11" order="2" />
<bond atomRefs2="a11 a8" order="1" />
<bond atomRefs2="a26 a28" order="1" />
<bond atomRefs2="a26 a23" order="1" />
<bond atomRefs2="a26 a29" order="1" />
<bond atomRefs2="a24 a23" order="1" />
<bond atomRefs2="a3 a1" order="1" />
<bond atomRefs2="a1 a2" order="1" />
<bond atomRefs2="a1 a8" order="1" />
<bond atomRefs2="a23 a25" order="1" />
<bond atomRefs2="a8 a10" order="1" />
<bond atomRefs2="a8 a9" order="1" />
</bondArray>
<propertyList>
<property title="program">
<scalar>Gaussian 16, Revision C.01</scalar>
</property>
<property title="basis">
<scalar>Aug-CC-pVTZ (5D, 7F)</scalar>
</property>
<property title="File Format">
<scalar>g03</scalar>
</property>
<property title="Energy" dictRef="me:ZPE">
<scalar units="kcal/mol" >17.57</scalar>
</property>
<property title="SpinMultiplicity" dictRef="me:spinMultiplicity">
<scalar>2.00</scalar>
</property>
<property title="Vibrational Frequencies" dictRef="me:vibFreqs">
<array units="cm-1">37.79 59.90 65.48 101.77 120.73 154.12 176.39 214.12 246.54 247.37 272.31 334.11 374.51 393.27 451.63 509.27 562.17 580.90 588.47 729.48 734.76 753.73 777.56 819.16 828.53 882.15 912.46 939.37 969.88 1010.99 1017.99 1051.82 1079.78 1085.33 1093.02 1108.49 1139.03 1147.18 1175.36 1204.56 1231.13 1240.33 1266.91 1281.88 1310.53 1327.28 1342.68 1346.98 1368.78 1385.73 1398.19 1410.90 1423.18 1425.50 1462.04 1475.99 1484.26 1495.30 1500.42 1503.01 1505.74 1508.87 1515.01 1869.36 2997.74 3020.12 3029.41 3036.84 3044.29 3055.44 3063.55 3068.56 3076.43 3084.64 3099.18 3109.64 3114.77 3115.42 3122.73 3169.18</array>
</property>
<property title="ImaginaryFrequency" dictRef="me:imFreqs">

<scalar units="cm-1">1765.73</scalar>
</property>
<property title="Rotational Constants" dictRef="me:rotConsts">
<array units="cm-1">0.061 0.013 0.012</array>
</property>
<property title="Symmetry Number" dictRef="me:symmetryNumber">
<scalar>1</scalar>
</property>
<property dictRef="me:frequenciesScaleFactor" >
<scalar>1</scalar>
</property>
</propertyList>
<me:DOSCMethod name="ClassicalRotors" />
</molecule>
<molecule id="Nonanal-19-rad" spinMultiplicity="2">
<atomArray>
<atom id="a1" elementType="C" x3="-1.875234" y3="-0.450329" z3="0.392331" />
<atom id="a2" elementType="O" x3="-1.724722" y3="0.405595" z3="1.228403" />
<atom id="a3" elementType="C" x3="-1.373689" y3="-1.862196" z3="0.460744" />
<atom id="a4" elementType="H" x3="-1.800000" y3="-2.301104" z3="1.363143" />
<atom id="a5" elementType="H" x3="-1.737791" y3="-2.422519" z3="-0.399365" />
<atom id="a6" elementType="C" x3="0.155930" y3="-1.891028" z3="0.528551" />
<atom id="a7" elementType="H" x3="0.471823" y3="-2.913149" z3="0.745535" />
<atom id="a8" elementType="H" x3="0.474862" y3="-1.276521" z3="1.371316" />
<atom id="a9" elementType="C" x3="0.810352" y3="-1.416192" z3="-0.764353" />
<atom id="a10" elementType="C" x3="2.334425" y3="-1.369385" z3="-0.707119" />
<atom id="a11" elementType="H" x3="2.716114" y3="-1.180484" z3="-1.712674" />
<atom id="a12" elementType="H" x3="2.723167" y3="-2.345777" z3="-0.403332" />
<atom id="a13" elementType="C" x3="2.895513" y3="-0.285757" z3="0.236385" />
<atom id="a14" elementType="H" x3="2.610559" y3="-0.513782" z3="1.266548" />
<atom id="a15" elementType="H" x3="3.987328" y3="-0.350312" z3="0.198756" />
<atom id="a16" elementType="O" x3="-2.524706" y3="-0.205797" z3="-0.755979" />
<atom id="a17" elementType="O" x3="-2.971445" y3="1.133778" z3="-0.823196" />
<atom id="a18" elementType="H" x3="0.438860" y3="-0.419756" z3="-1.022530" />
<atom id="a19" elementType="H" x3="0.499171" y3="-2.076273" z3="-1.577911" />
<atom id="a20" elementType="C" spinMultiplicity="2" x3="2.445766" y3="1.087113" z3="-
0.118389" />
<atom id="a21" elementType="H" x3="-2.631363" y3="1.485697" z3="0.024929" />
<atom id="a22" elementType="H" x3="2.865918" y3="1.551972" z3="-1.004328" />
<atom id="a23" elementType="C" x3="1.418465" y3="1.838869" z3="0.651060" />
<atom id="a24" elementType="H" x3="1.906046" y3="2.521836" z3="1.362049" />
<atom id="a25" elementType="H" x3="0.835157" y3="1.148645" z3="1.265548" />
<atom id="a26" elementType="C" x3="0.473522" y3="2.654560" z3="-0.229760" />
<atom id="a27" elementType="H" x3="-0.087072" y3="2.008650" z3="-0.907303" />
<atom id="a28" elementType="H" x3="-0.243235" y3="3.208492" z3="0.376686" />
<atom id="a29" elementType="H" x3="1.027135" y3="3.371839" z3="-0.837589" />

```
</atomArray>
<bondArray>
  <bond atomRefs2="a11 a10" order="1" />
  <bond atomRefs2="a19 a9" order="1" />
  <bond atomRefs2="a18 a9" order="1" />
  <bond atomRefs2="a22 a20" order="1" />
  <bond atomRefs2="a27 a26" order="1" />
  <bond atomRefs2="a29 a26" order="1" />
  <bond atomRefs2="a17 a16" order="1" />
  <bond atomRefs2="a17 a21" order="1" />
  <bond atomRefs2="a9 a10" order="1" />
  <bond atomRefs2="a9 a6" order="1" />
  <bond atomRefs2="a16 a1" order="1" />
  <bond atomRefs2="a10 a12" order="1" />
  <bond atomRefs2="a10 a13" order="1" />
  <bond atomRefs2="a5 a3" order="1" />
  <bond atomRefs2="a26 a28" order="1" />
  <bond atomRefs2="a26 a23" order="1" />
  <bond atomRefs2="a20 a13" order="1" />
  <bond atomRefs2="a20 a23" order="1" />
  <bond atomRefs2="a15 a13" order="1" />
  <bond atomRefs2="a13 a14" order="1" />
  <bond atomRefs2="a1 a3" order="1" />
  <bond atomRefs2="a1 a2" order="2" />
  <bond atomRefs2="a3 a6" order="1" />
  <bond atomRefs2="a3 a4" order="1" />
  <bond atomRefs2="a6 a7" order="1" />
  <bond atomRefs2="a6 a8" order="1" />
  <bond atomRefs2="a23 a25" order="1" />
  <bond atomRefs2="a23 a24" order="1" />
</bondArray>
<propertyList>
  <property title="program">
    <scalar>Gaussian 16, Revision C.01</scalar>
  </property>
  <property title="basis">
    <scalar>Aug-CC-pVTZ (5D, 7F)</scalar>
  </property>
  <property title="File Format">
    <scalar>g03</scalar>
  </property>
  <property title="Energy" dictRef="me:ZPE">
    <scalar units="kcal/mol" >2.27</scalar>
  </property>
  <property title="SpinMultiplicity" dictRef="me:spinMultiplicity">
    <scalar>2.00</scalar>
```

</property>
<property title="Vibrational Frequencies" dictRef="me:vibFreqs">
<array units="cm-1">34.54 36.49 57.99 62.58 81.39 92.49 162.18 202.66 225.21 228.65 251.88 295.95 328.94 338.17 368.80 427.40 445.18 457.82 484.86 531.66 638.26 719.28 765.46 786.51 821.76 844.49 873.57 912.43 952.00 970.60 990.45 1023.33 1048.92 1058.42 1061.12 1084.65 1095.97 1130.09 1158.38 1175.54 1201.09 1234.11 1279.42 1289.84 1292.71 1316.07 1341.19 1349.57 1369.56 1388.50 1394.14 1416.10 1419.58 1437.64 1480.39 1485.07 1492.06 1493.60 1500.18 1501.77 1502.79 1506.65 1512.30 1817.90 2962.38 3023.26 3035.65 3038.87 3043.53 3061.54 3065.13 3068.53 3074.55 3080.11 3087.28 3111.34 3114.73 3120.78 3135.49 3157.64 3555.62</array>
</property>
<property title="Rotational Constants" dictRef="me:rotConsts">
<array units="cm-1">0.037 0.022 0.016</array>
</property>
<property title="Symmetry Number" dictRef="me:symmetryNumber">
<scalar>1</scalar>
</property>
</propertyList>
</molecule>
<molecule id="19Hshift-TS" spinMultiplicity="2">
<atomArray>
<atom id="a1" elementType="C" x3="-1.870594" y3="-1.053754" z3="-0.074349" />
<atom id="a2" elementType="O" x3="-2.643232" y3="-1.383216" z3="-0.922497" />
<atom id="a3" elementType="C" x3="-2.014454" y3="0.172835" z3="0.788056" />
<atom id="a4" elementType="H" x3="-3.002136" y3="0.114435" z3="1.244313" />
<atom id="a5" elementType="H" x3="-1.276616" y3="0.188583" z3="1.580240" />
<atom id="a6" elementType="C" x3="-1.942732" y3="1.423204" z3="-0.102179" />
<atom id="a7" elementType="H" x3="-2.063785" y3="2.305410" z3="0.530199" />
<atom id="a8" elementType="H" x3="-2.802858" y3="1.394610" z3="-0.770731" />
<atom id="a9" elementType="C" x3="-0.670946" y3="1.557731" z3="-0.947837" />
<atom id="a10" elementType="C" x3="0.517715" y3="2.257514" z3="-0.287789" />
<atom id="a11" elementType="H" x3="0.198871" y3="3.255461" z3="0.023995" />
<atom id="a12" elementType="H" x3="1.283040" y3="2.416307" z3="-1.051085" />
<atom id="a13" elementType="C" x3="1.167363" y3="1.579526" z3="0.917394" />
<atom id="a14" elementType="H" x3="1.972505" y3="2.235566" z3="1.269846" />
<atom id="a15" elementType="H" x3="0.465321" y3="1.524754" z3="1.752718" />
<atom id="a16" elementType="O" x3="-0.738828" y3="-1.830896" z3="0.040132" />
<atom id="a17" elementType="O" x3="0.049057" y3="-1.560881" z3="1.145750" />
<atom id="a18" elementType="H" x3="-0.922548" y3="2.134580" z3="-1.839068" />
<atom id="a19" elementType="H" x3="-0.370598" y3="0.574303" z3="-1.319812" />
<atom id="a20" elementType="C" spinMultiplicity="2" x3="1.780261" y3="0.215059" z3="0.692971" />
<atom id="a21" elementType="H" x3="0.851920" y3="-0.677936" z3="0.814202" />
<atom id="a22" elementType="H" x3="2.362995" y3="-0.107996" z3="1.558629" />
<atom id="a23" elementType="C" x3="2.484534" y3="-0.075692" z3="-0.606507" />
<atom id="a24" elementType="H" x3="3.277603" y3="0.672504" z3="-0.733696" />
<atom id="a25" elementType="H" x3="1.803863" y3="0.069968" z3="-1.446910" />

<atom id="a26" elementType="C" x3="3.080561" y3="-1.476080" z3="-0.662333" />
<atom id="a27" elementType="H" x3="3.596011" y3="-1.643658" z3="-1.607044" />
<atom id="a28" elementType="H" x3="3.798901" y3="-1.627688" z3="0.144628" />
<atom id="a29" elementType="H" x3="2.301284" y3="-2.231317" z3="-0.562069" />
</atomArray>
<bondArray>
<bond atomRefs2="a18 a9" order="1" />
<bond atomRefs2="a27 a26" order="1" />
<bond atomRefs2="a25 a23" order="1" />
<bond atomRefs2="a19 a9" order="1" />
<bond atomRefs2="a12 a10" order="1" />
<bond atomRefs2="a9 a10" order="1" />
<bond atomRefs2="a9 a6" order="1" />
<bond atomRefs2="a2 a1" order="2" />
<bond atomRefs2="a8 a6" order="1" />
<bond atomRefs2="a24 a23" order="1" />
<bond atomRefs2="a26 a23" order="1" />
<bond atomRefs2="a26 a29" order="1" />
<bond atomRefs2="a26 a28" order="1" />
<bond atomRefs2="a23 a20" order="1" />
<bond atomRefs2="a10 a11" order="1" />
<bond atomRefs2="a10 a13" order="1" />
<bond atomRefs2="a6 a7" order="1" />
<bond atomRefs2="a6 a3" order="1" />
<bond atomRefs2="a1 a16" order="1" />
<bond atomRefs2="a1 a3" order="1" />
<bond atomRefs2="a16 a17" order="1" />
<bond atomRefs2="a20 a13" order="1" />
<bond atomRefs2="a20 a22" order="1" />
<bond atomRefs2="a3 a4" order="1" />
<bond atomRefs2="a3 a5" order="1" />
<bond atomRefs2="a21 a17" order="1" />
<bond atomRefs2="a13 a14" order="1" />
<bond atomRefs2="a13 a15" order="1" />
</bondArray>
<propertyList>
<property title="program">
<scalar>Gaussian 16, Revision C.01</scalar>
</property>
<property title="basis">
<scalar>Aug-CC-pVTZ (5D, 7F)</scalar>
</property>
<property title="File Format">
<scalar>g03</scalar>
</property>
<property title="Energy" dictRef="me:ZPE">

<scalar units="kcal/mol" >20.67</scalar>
</property>
<property title="SpinMultiplicity" dictRef="me:spinMultiplicity">
<scalar>2.00</scalar>
</property>
<property title="Vibrational Frequencies" dictRef="me:vibFreqs">
<array units="cm-1">49.77 72.65 85.44 131.30 139.97 176.62 187.84 217.04 242.27 274.57 336.00 365.10 377.45 385.91 441.28 460.64 512.73 577.86 600.85 699.06 754.61 766.37 779.73 799.91 856.63 882.23 896.98 950.10 986.54 993.77 1031.98 1046.10 1073.80 1082.75 1092.77 1106.96 1137.05 1152.93 1162.85 1193.39 1208.82 1234.15 1266.99 1289.24 1306.81 1329.67 1337.50 1375.17 1386.34 1394.52 1406.36 1417.06 1426.11 1428.61 1452.22 1476.65 1479.57 1491.36 1503.10 1504.14 1507.22 1507.90 1510.64 1865.74 3001.79 3010.73 3043.50 3047.29 3051.46 3056.69 3065.60 3073.28 3084.59 3087.40 3093.03 3100.98 3107.46 3124.77 3134.62 3198.23</array>
</property>
<property title="ImaginaryFrequency" dictRef="me:imFreqs">
<scalar units="cm-1">1776.02</scalar>
</property>
<property title="Rotational Constants" dictRef="me:rotConsts">
<array units="cm-1">0.037 0.024 0.018</array>
</property>
<property title="Symmetry Number" dictRef="me:symmetryNumber">
<scalar>1</scalar>
</property>
<property dictRef="me:frequenciesScaleFactor" >
<scalar>1</scalar>
</property>
</propertyList>
<me:DOSCMethod name="ClassicalRotors" />
</molecule>
<molecule id="Nonanal-16-rad" spinMultiplicity="2">
<atomArray>
<atom id="a1" elementType="C" x3="1.163550" y3="1.328559" z3="0.299031" />
<atom id="a2" elementType="O" x3="0.585650" y3="1.220754" z3="1.351641" />
<atom id="a3" elementType="C" x3="2.499781" y3="0.745798" z3="-0.052814" />
<atom id="a4" elementType="H" x3="2.748050" y3="0.998745" z3="-1.082258" />
<atom id="a5" elementType="H" x3="3.234096" y3="1.218826" z3="0.601097" />
<atom id="a6" elementType="C" x3="2.507405" y3="-0.776299" z3="0.154711" />
<atom id="a7" elementType="H" x3="2.250154" y3="-0.993999" z3="1.192870" />
<atom id="a8" elementType="H" x3="3.541342" y3="-1.115126" z3="0.014591" />
<atom id="a9" elementType="C" spinMultiplicity="2" x3="1.589252" y3="-1.497600" z3="-0.768443" />
<atom id="a10" elementType="C" x3="0.649967" y3="-2.543097" z3="-0.281033" />
<atom id="a11" elementType="H" x3="1.189161" y3="-3.247513" z3="0.363263" />
<atom id="a12" elementType="H" x3="0.264378" y3="-3.119692" z3="-1.124098" />
<atom id="a13" elementType="C" x3="-0.534981" y3="-1.975175" z3="0.522318" />
<atom id="a14" elementType="H" x3="-1.096328" y3="-2.806303" z3="0.957104" />

<atom id="a15" elementType="H" x3="-0.153100" y3="-1.385736" z3="1.359989" />
<atom id="a16" elementType="O" x3="0.637914" y3="2.017837" z3="-0.725466" />
<atom id="a17" elementType="H" x3="1.609005" y3="-1.231182" z3="-1.817989" />
<atom id="a18" elementType="C" x3="-1.470607" y3="-1.110783" z3="-0.312109" />
<atom id="a19" elementType="H" x3="-1.907051" y3="-1.716606" z3="-1.113227" />
<atom id="a20" elementType="H" x3="-0.898634" y3="-0.322029" z3="-0.810132" />
<atom id="a21" elementType="C" x3="-2.584889" y3="-0.473112" z3="0.507255" />
<atom id="a22" elementType="H" x3="-3.149458" y3="-1.256149" z3="1.021361" />
<atom id="a23" elementType="H" x3="-2.135682" y3="0.141830" z3="1.292405" />
<atom id="a24" elementType="C" x3="-3.529808" y3="0.372928" z3="-0.335835" />
<atom id="a25" elementType="H" x3="-4.017125" y3="-0.233228" z3="-1.101758" />
<atom id="a26" elementType="H" x3="-4.310242" y3="0.828533" z3="0.274058" />
<atom id="a27" elementType="H" x3="-2.986686" y3="1.172275" z3="-0.842401" />
<atom id="a28" elementType="O" x3="-0.621975" y3="2.563002" z3="-0.389557" />
<atom id="a29" elementType="H" x3="-0.732607" y3="2.227292" z3="0.523696" />
</atomArray>
<bondArray>
<bond atomRefs2="a17 a9" order="1" />
<bond atomRefs2="a12 a10" order="1" />
<bond atomRefs2="a19 a18" order="1" />
<bond atomRefs2="a25 a24" order="1" />
<bond atomRefs2="a4 a3" order="1" />
<bond atomRefs2="a27 a24" order="1" />
<bond atomRefs2="a20 a18" order="1" />
<bond atomRefs2="a9 a10" order="1" />
<bond atomRefs2="a9 a6" order="1" />
<bond atomRefs2="a16 a28" order="1" />
<bond atomRefs2="a16 a1" order="1" />
<bond atomRefs2="a28 a29" order="1" />
<bond atomRefs2="a24 a26" order="1" />
<bond atomRefs2="a24 a21" order="1" />
<bond atomRefs2="a18 a21" order="1" />
<bond atomRefs2="a18 a13" order="1" />
<bond atomRefs2="a10 a11" order="1" />
<bond atomRefs2="a10 a13" order="1" />
<bond atomRefs2="a3 a6" order="1" />
<bond atomRefs2="a3 a1" order="1" />
<bond atomRefs2="a3 a5" order="1" />
<bond atomRefs2="a8 a6" order="1" />
<bond atomRefs2="a6 a7" order="1" />
<bond atomRefs2="a1 a2" order="2" />
<bond atomRefs2="a21 a22" order="1" />
<bond atomRefs2="a21 a23" order="1" />
<bond atomRefs2="a13 a14" order="1" />
<bond atomRefs2="a13 a15" order="1" />
</bondArray>

<propertyList>
<property title="program">
<scalar>Gaussian 16, Revision C.01</scalar>
</property>
<property title="basis">
<scalar>Aug-CC-pVTZ (5D, 7F)</scalar>
</property>
<property title="File Format">
<scalar>g03</scalar>
</property>
<property title="Energy" dictRef="me:ZPE">
<scalar units="kcal/mol" >1.03</scalar>
</property>
<property title="SpinMultiplicity" dictRef="me:spinMultiplicity">
<scalar>2.00</scalar>
</property>
<property title="Vibrational Frequencies" dictRef="me:vibFreqs">
<array units="cm-1">31.30 48.15 63.66 86.54 109.01 114.32 153.13 171.23 195.69 226.91 248.97 274.48 326.07 344.40 385.07 403.90 431.64 460.78 488.01 527.13 631.77 723.73 737.35 784.50 840.63 866.32 905.55 922.77 927.49 957.84 984.23 1002.28 1043.78 1053.30 1061.75 1086.79 1122.82 1134.82 1148.87 1194.59 1216.46 1242.54 1247.16 1285.76 1308.22 1318.36 1339.09 1340.14 1361.57 1382.90 1393.75 1416.76 1420.99 1433.07 1477.48 1484.92 1488.91 1493.37 1498.36 1505.47 1507.43 1518.42 1523.42 1820.02 2998.23 3006.66 3022.62 3032.69 3041.13 3042.01 3053.06 3068.39 3076.08 3077.56 3086.41 3090.75 3112.66 3119.64 3134.56 3180.07 3558.36</array>
</property>
<property title="Rotational Constants" dictRef="me:rotConsts">
<array units="cm-1">0.032 0.026 0.016</array>
</property>
<property title="Symmetry Number" dictRef="me:symmetryNumber">
<scalar>1</scalar>
</property>
</propertyList>
</molecule>
<molecule id="16Hshift-TS" spinMultiplicity="2">
<atomArray>
<atom id="a1" elementType="C" x3="2.474621" y3="-0.803265" z3="-0.148198" />
<atom id="a2" elementType="O" x3="3.245669" y3="-1.320788" z3="-0.896621" />
<atom id="a3" elementType="C" x3="2.664132" y3="0.530780" z3="0.526486" />
<atom id="a4" elementType="H" x3="2.495906" y3="0.444864" z3="1.598824" />
<atom id="a5" elementType="H" x3="3.699052" y3="0.809675" z3="0.350179" />
<atom id="a6" elementType="C" x3="1.717690" y3="1.585598" z3="-0.082253" />
<atom id="a7" elementType="H" x3="1.776787" y3="1.524898" z3="-1.170711" />
<atom id="a8" elementType="H" x3="2.090727" y3="2.574221" z3="0.195149" />
<atom id="a9" elementType="C" spinMultiplicity="2" x3="0.275163" y3="1.459617" z3="0.360472" />
<atom id="a10" elementType="C" x3="-0.781746" y3="1.767541" z3="-0.664605" />

<atom id="a11" elementType="H" x3="-0.650187" y3="1.102224" z3="-1.522058" />
<atom id="a12" elementType="H" x3="-0.587879" y3="2.777370" z3="-1.048073" />
<atom id="a13" elementType="C" x3="-2.216471" y3="1.700708" z3="-0.146045" />
<atom id="a14" elementType="H" x3="-2.347358" y3="2.462602" z3="0.627112" />
<atom id="a15" elementType="H" x3="-2.889190" y3="1.973704" z3="-0.962706" />
<atom id="a16" elementType="O" x3="1.259838" y3="-1.425221" z3="0.018270" />
<atom id="a17" elementType="O" x3="0.506650" y3="-0.914466" z3="1.065612" />
<atom id="a18" elementType="H" x3="0.085745" y3="1.900050" z3="1.341568" />
<atom id="a19" elementType="H" x3="0.179320" y3="0.219034" z3="0.689414" />
<atom id="a20" elementType="C" x3="-2.635985" y3="0.342772" z3="0.415287" />
<atom id="a21" elementType="H" x3="-3.668153" y3="0.419730" z3="0.766851" />
<atom id="a22" elementType="H" x3="-2.037876" y3="0.102124" z3="1.298840" />
<atom id="a23" elementType="C" x3="-2.534952" y3="-0.809115" z3="-0.578153" />
<atom id="a24" elementType="H" x3="-3.124706" y3="-0.569354" z3="-1.467966" />
<atom id="a25" elementType="H" x3="-1.501514" y3="-0.924176" z3="-0.910987" />
<atom id="a26" elementType="C" x3="-3.004235" y3="-2.129503" z3="0.016703" />
<atom id="a27" elementType="H" x3="-2.391719" y3="-2.399092" z3="0.878225" />
<atom id="a28" elementType="H" x3="-4.042230" y3="-2.065196" z3="0.348674" />
<atom id="a29" elementType="H" x3="-2.933281" y3="-2.939681" z3="-0.708586" />
</atomArray>
<bondArray>
<bond atomRefs2="a11 a10" order="1" />
<bond atomRefs2="a24 a23" order="1" />
<bond atomRefs2="a7 a6" order="1" />
<bond atomRefs2="a12 a10" order="1" />
<bond atomRefs2="a15 a13" order="1" />
<bond atomRefs2="a25 a23" order="1" />
<bond atomRefs2="a2 a1" order="2" />
<bond atomRefs2="a29 a26" order="1" />
<bond atomRefs2="a10 a13" order="1" />
<bond atomRefs2="a10 a9" order="1" />
<bond atomRefs2="a23 a26" order="1" />
<bond atomRefs2="a23 a20" order="1" />
<bond atomRefs2="a1 a16" order="1" />
<bond atomRefs2="a1 a3" order="1" />
<bond atomRefs2="a13 a20" order="1" />
<bond atomRefs2="a13 a14" order="1" />
<bond atomRefs2="a6 a8" order="1" />
<bond atomRefs2="a6 a9" order="1" />
<bond atomRefs2="a6 a3" order="1" />
<bond atomRefs2="a26 a28" order="1" />
<bond atomRefs2="a26 a27" order="1" />
<bond atomRefs2="a16 a17" order="1" />
<bond atomRefs2="a5 a3" order="1" />
<bond atomRefs2="a9 a18" order="1" />
<bond atomRefs2="a20 a21" order="1" />

```
<bond atomRefs2="a20 a22" order="1" />
<bond atomRefs2="a3 a4" order="1" />
<bond atomRefs2="a19 a17" order="1" />
</bondArray>
<propertyList>
<property title="program">
<scalar>Gaussian 16, Revision C.01</scalar>
</property>
<property title="basis">
<scalar>Aug-CC-pVTZ (5D, 7F)</scalar>
</property>
<property title="File Format">
<scalar>g03</scalar>
</property>
<property title="Energy" dictRef="me:ZPE">
<scalar units="kcal/mol" >19.14</scalar>
</property>
<property title="SpinMultiplicity" dictRef="me:spinMultiplicity">
<scalar>2.00</scalar>
</property>
<property title="Vibrational Frequencies" dictRef="me:vibFreqs">
<array units="cm-1">30.34 37.14 44.98 107.89 119.72 159.57 212.81 226.65 248.08 278.43
295.19 305.96 370.54 401.02 433.26 502.28 512.97 556.82 583.15 733.03 737.16 769.23 783.00 846.41
860.63 892.04 902.97 937.14 959.72 1011.45 1050.09 1055.44 1062.50 1076.30 1090.84 1124.21
1137.03 1143.56 1172.24 1192.88 1234.17 1254.59 1263.11 1291.90 1309.10 1320.00 1338.15 1352.87
1366.31 1380.89 1398.43 1413.25 1420.82 1420.87 1450.60 1479.26 1482.57 1495.84 1496.34 1498.12
1505.04 1507.91 1516.74 1872.70 3003.80 3032.06 3037.58 3042.21 3044.39 3048.20 3061.12 3066.64
3076.19 3084.95 3089.72 3093.33 3104.27 3115.43 3123.76 3161.66</array>
</property>
<property title="ImaginaryFrequency" dictRef="me:imFreqs">
<scalar units="cm-1">1757.36</scalar>
</property>
<property title="Rotational Constants" dictRef="me:rotConsts">
<array units="cm-1">0.043 0.019 0.015</array>
</property>
<property title="Symmetry Number" dictRef="me:symmetryNumber">
<scalar>1</scalar>
</property>
<property dictRef="me:frequenciesScaleFactor" >
<scalar>1</scalar>
</property>
</propertyList>
<me:DOSCMethod name="ClassicalRotors" />
</molecule>
<molecule id="Nonanal-18-rad" spinMultiplicity="2">
<atomArray>
```

<atom id="a1" elementType="C" x3="2.032562" y3="-0.219898" z3="0.322302" />
<atom id="a2" elementType="O" x3="1.397576" y3="-1.231182" z3="0.151978" />
<atom id="a3" elementType="C" x3="1.642930" y3="0.958296" z3="1.167404" />
<atom id="a4" elementType="H" x3="1.857527" y3="0.676167" z3="2.200507" />
<atom id="a5" elementType="H" x3="2.285169" y3="1.803424" z3="0.921226" />
<atom id="a6" elementType="C" x3="0.165390" y3="1.302955" z3="1.007140" />
<atom id="a7" elementType="H" x3="-0.099219" y3="2.055550" z3="1.752275" />
<atom id="a8" elementType="H" x3="-0.431127" y3="0.417262" z3="1.229079" />
<atom id="a9" elementType="C" x3="-0.178530" y3="1.818037" z3="-0.385392" />
<atom id="a10" elementType="C" x3="-1.680442" y3="2.060166" z3="-0.571806" />
<atom id="a11" elementType="H" x3="-1.833619" y3="2.454625" z3="-1.587484" />
<atom id="a12" elementType="H" x3="-2.001668" y3="2.855904" z3="0.104773" />
<atom id="a13" elementType="C" spinMultiplicity="2" x3="-2.533350" y3="0.856405" z3="-
0.357827" />
<atom id="a14" elementType="H" x3="-3.507239" y3="0.993173" z3="0.098272" />
<atom id="a15" elementType="O" x3="3.233036" y3="-0.039756" z3="-0.248739" />
<atom id="a16" elementType="H" x3="0.169841" y3="1.104802" z3="-1.136659" />
<atom id="a17" elementType="H" x3="0.360717" y3="2.748901" z3="-0.576858" />
<atom id="a18" elementType="C" x3="-2.244476" y3="-0.461615" z3="-0.984613" />
<atom id="a19" elementType="H" x3="-1.165594" y3="-0.642355" z3="-1.018813" />
<atom id="a20" elementType="H" x3="-2.571898" y3="-0.449218" z3="-2.035678" />
<atom id="a21" elementType="C" x3="-2.913559" y3="-1.636341" z3="-0.269897" />
<atom id="a22" elementType="H" x3="-3.992874" y3="-1.465452" z3="-0.228689" />
<atom id="a23" elementType="H" x3="-2.766041" y3="-2.542306" z3="-0.860747" />
<atom id="a24" elementType="C" x3="-2.369784" y3="-1.852736" z3="1.136990" />
<atom id="a25" elementType="H" x3="-1.293103" y3="-2.030282" z3="1.109172" />
<atom id="a26" elementType="H" x3="-2.546259" y3="-0.975625" z3="1.762414" />
<atom id="a27" elementType="H" x3="-2.842772" y3="-2.708052" z3="1.619659" />
<atom id="a28" elementType="O" x3="3.620591" y3="-1.166395" z3="-1.009691" />
<atom id="a29" elementType="H" x3="2.844103" y3="-1.749479" z3="-0.886632" />
</atomArray>
<bondArray>
<bond atomRefs2="a20 a18" order="1" />
<bond atomRefs2="a11 a10" order="1" />
<bond atomRefs2="a16 a9" order="1" />
<bond atomRefs2="a19 a18" order="1" />
<bond atomRefs2="a28 a29" order="1" />
<bond atomRefs2="a28 a15" order="1" />
<bond atomRefs2="a18 a13" order="1" />
<bond atomRefs2="a18 a21" order="1" />
<bond atomRefs2="a23 a21" order="1" />
<bond atomRefs2="a17 a9" order="1" />
<bond atomRefs2="a10 a9" order="1" />
<bond atomRefs2="a10 a13" order="1" />
<bond atomRefs2="a10 a12" order="1" />
<bond atomRefs2="a9 a6" order="1" />

<bond atomRefs2="a13 a14" order="1" />
<bond atomRefs2="a21 a22" order="1" />
<bond atomRefs2="a21 a24" order="1" />
<bond atomRefs2="a15 a1" order="1" />
<bond atomRefs2="a2 a1" order="2" />
<bond atomRefs2="a1 a3" order="1" />
<bond atomRefs2="a5 a3" order="1" />
<bond atomRefs2="a6 a3" order="1" />
<bond atomRefs2="a6 a8" order="1" />
<bond atomRefs2="a6 a7" order="1" />
<bond atomRefs2="a25 a24" order="1" />
<bond atomRefs2="a24 a27" order="1" />
<bond atomRefs2="a24 a26" order="1" />
<bond atomRefs2="a3 a4" order="1" />
</bondArray>
<propertyList>
<property title="program">
<scalar>Gaussian 16, Revision C.01</scalar>
</property>
<property title="basis">
<scalar>Aug-CC-pVTZ (5D, 7F)</scalar>
</property>
<property title="File Format">
<scalar>g03</scalar>
</property>
<property title="Energy" dictRef="me:ZPE">
<scalar units="kcal/mol" >1.83</scalar>
</property>
<property title="SpinMultiplicity" dictRef="me:spinMultiplicity">
<scalar>2.00</scalar>
</property>
<property title="Vibrational Frequencies" dictRef="me:vibFreqs">
<array units="cm-1">20.91 29.75 44.09 51.18 86.78 130.73 164.11 204.84 217.29 227.69 246.37 282.63 305.87 329.48 378.83 413.01 452.79 472.97 494.91 583.39 625.57 700.56 779.11 786.88 836.24 842.44 881.62 909.43 932.65 953.08 987.91 1013.80 1051.48 1067.46 1078.85 1083.72 1089.97 1133.14 1163.53 1181.65 1213.88 1246.29 1255.53 1279.96 1294.55 1326.15 1339.10 1351.80 1378.25 1386.77 1403.90 1415.78 1419.86 1436.62 1473.44 1476.30 1477.00 1494.95 1499.04 1504.05 1507.95 1514.13 1524.79 1815.63 2954.12 2960.48 3037.91 3039.61 3044.51 3053.01 3059.89 3061.28 3070.39 3080.24 3082.87 3110.80 3111.46 3118.72 3125.44 3166.59 3559.23</array>
</property>
<property title="Rotational Constants" dictRef="me:rotConsts">
<array units="cm-1">0.040 0.016 0.014</array>
</property>
<property title="Symmetry Number" dictRef="me:symmetryNumber">
<scalar>1</scalar>
</property>

</propertyList>
</molecule>
<molecule id="18Hshift-TS" spinMultiplicity="2">
<atomArray>
<atom id="a1" elementType="C" x3="1.734188" y3="-1.285512" z3="-0.233067" />
<atom id="a2" elementType="O" x3="2.053799" y3="-1.794640" z3="-1.265143" />
<atom id="a3" elementType="C" x3="2.603624" y3="-0.352776" z3="0.568808" />
<atom id="a4" elementType="H" x3="2.268110" y3="-0.285570" z3="1.598977" />
<atom id="a5" elementType="H" x3="3.588393" y3="-0.817906" z3="0.548279" />
<atom id="a6" elementType="C" x3="2.719902" y3="1.046204" z3="-0.055591" />
<atom id="a7" elementType="H" x3="2.950176" y3="0.940343" z3="-1.118005" />
<atom id="a8" elementType="H" x3="3.585425" y3="1.527439" z3="0.401666" />
<atom id="a9" elementType="C" x3="1.512164" y3="1.966018" z3="0.124246" />
<atom id="a10" elementType="C" x3="0.246613" y3="1.553784" z3="-0.649958" />
<atom id="a11" elementType="H" x3="0.480850" y3="0.783269" z3="-1.389740" />
<atom id="a12" elementType="H" x3="-0.117180" y3="2.405062" z3="-1.236122" />
<atom id="a13" elementType="C" spinMultiplicity="2" x3="-0.900672" y3="1.088395" z3="0.208225" />
<atom id="a14" elementType="H" x3="-0.435365" y3="0.109619" z3="0.897609" />
<atom id="a15" elementType="H" x3="-1.158356" y3="1.789950" z3="1.005283" />
<atom id="a16" elementType="O" x3="0.476888" y3="-1.603799" z3="0.229125" />
<atom id="a17" elementType="O" x3="0.089688" y3="-0.914421" z3="1.365772" />
<atom id="a18" elementType="H" x3="1.815393" y3="2.965069" z3="-0.190299" />
<atom id="a19" elementType="H" x3="1.278589" y3="2.043727" z3="1.189349" />
<atom id="a20" elementType="C" x3="-2.091066" y3="0.506794" z3="-0.503371" />
<atom id="a21" elementType="H" x3="-1.757943" y3="-0.322503" z3="-1.134648" />
<atom id="a22" elementType="H" x3="-2.488215" y3="1.266377" z3="-1.189905" />
<atom id="a23" elementType="C" x3="-3.199637" y3="0.033075" z3="0.429129" />
<atom id="a24" elementType="H" x3="-2.791511" y3="-0.716489" z3="1.110212" />
<atom id="a25" elementType="H" x3="-3.530517" y3="0.870629" z3="1.049200" />
<atom id="a26" elementType="C" x3="-4.387426" y3="-0.548923" z3="-0.324501" />
<atom id="a27" elementType="H" x3="-4.083687" y3="-1.406927" z3="-0.926127" />
<atom id="a28" elementType="H" x3="-5.166931" y3="-0.881256" z3="0.360484" />
<atom id="a29" elementType="H" x3="-4.826362" y3="0.189700" z3="-0.997763" />
</atomArray>
<bondArray>
<bond atomRefs2="a11 a10" order="1" />
<bond atomRefs2="a2 a1" order="2" />
<bond atomRefs2="a12 a10" order="1" />
<bond atomRefs2="a22 a20" order="1" />
<bond atomRefs2="a21 a20" order="1" />
<bond atomRefs2="a7 a6" order="1" />
<bond atomRefs2="a29 a26" order="1" />
<bond atomRefs2="a27 a26" order="1" />
<bond atomRefs2="a10 a9" order="1" />
<bond atomRefs2="a10 a13" order="1" />

<bond atomRefs2="a20 a13" order="1" />
<bond atomRefs2="a20 a23" order="1" />
<bond atomRefs2="a26 a28" order="1" />
<bond atomRefs2="a26 a23" order="1" />
<bond atomRefs2="a1 a16" order="1" />
<bond atomRefs2="a1 a3" order="1" />
<bond atomRefs2="a18 a9" order="1" />
<bond atomRefs2="a6 a9" order="1" />
<bond atomRefs2="a6 a8" order="1" />
<bond atomRefs2="a6 a3" order="1" />
<bond atomRefs2="a9 a19" order="1" />
<bond atomRefs2="a13 a15" order="1" />
<bond atomRefs2="a16 a17" order="1" />
<bond atomRefs2="a23 a25" order="1" />
<bond atomRefs2="a23 a24" order="1" />
<bond atomRefs2="a5 a3" order="1" />
<bond atomRefs2="a3 a4" order="1" />
<bond atomRefs2="a14 a17" order="1" />
</bondArray>
<propertyList>
<property title="program">
<scalar>Gaussian 16, Revision C.01</scalar>
</property>
<property title="basis">
<scalar>Aug-CC-pVTZ (5D, 7F)</scalar>
</property>
<property title="File Format">
<scalar>g03</scalar>
</property>
<property title="Energy" dictRef="me:ZPE">
<scalar units="kcal/mol">18.11</scalar>
</property>
<property title="SpinMultiplicity" dictRef="me:spinMultiplicity">
<scalar>2.00</scalar>
</property>
<property title="Vibrational Frequencies" dictRef="me:vibFreqs">
<array units="cm-1">37.63 65.50 69.48 88.74 105.42 144.18 165.21 232.89 243.98 255.48 296.70 343.91 369.33 394.57 461.09 472.91 518.78 559.32 593.83 694.13 741.94 774.54 782.30 841.60 873.84 884.14 914.34 945.95 988.55 1003.67 1037.14 1062.61 1088.64 1092.65 1096.82 1108.62 1132.00 1135.01 1169.28 1170.72 1224.07 1248.39 1273.04 1279.27 1295.16 1318.63 1340.72 1350.82 1382.61 1393.51 1398.37 1410.01 1423.71 1424.83 1448.16 1472.74 1483.74 1489.53 1500.23 1500.91 1502.05 1506.40 1513.66 1861.76 2997.28 3017.06 3040.57 3043.72 3045.45 3054.71 3055.24 3061.64 3078.93 3085.93 3091.56 3095.70 3103.18 3115.11 3124.54 3164.44</array>
</property>
<property title="ImaginaryFrequency" dictRef="me:imFreqs">
<scalar units="cm-1">1687.27</scalar>

</property>
<property title="Rotational Constants" dictRef="me:rotConsts">
<array units="cm-1">0.047 0.018 0.015</array>
</property>
<property title="Symmetry Number" dictRef="me:symmetryNumber">
<scalar>1</scalar>
</property>
<property dictRef="me:frequenciesScaleFactor">
<scalar>1</scalar>
</property>
</propertyList>
<me:DOSCMethod name="ClassicalRotors" />
</molecule>
<molecule id="Nonanal-AcylOO-rad" spinMultiplicity="2">
<atomArray>
<atom id="a1" elementType="C" x3="-6.404728" y3="0.095781" z3="0.000000" />
<atom id="a2" elementType="H" x3="-7.246056" y3="0.788757" z3="0.000000" />
<atom id="a3" elementType="H" x3="-6.497563" y3="-0.542334" z3="0.880620" />
<atom id="a4" elementType="H" x3="-6.497563" y3="-0.542334" z3="-0.880620" />
<atom id="a5" elementType="C" x3="-5.072735" y3="0.834019" z3="0.000000" />
<atom id="a6" elementType="H" x3="-5.016105" y3="1.488309" z3="0.874322" />
<atom id="a7" elementType="H" x3="-5.016105" y3="1.488309" z3="-0.874322" />
<atom id="a8" elementType="C" x3="-3.873062" y3="-0.105454" z3="0.000000" />
<atom id="a9" elementType="H" x3="-3.928455" y3="-0.761279" z3="0.874811" />
<atom id="a10" elementType="H" x3="-3.928455" y3="-0.761279" z3="-0.874811" />
<atom id="a11" elementType="C" x3="-2.534971" y3="0.622086" z3="0.000000" />
<atom id="a12" elementType="H" x3="-2.479969" y3="1.277567" z3="0.874979" />
<atom id="a13" elementType="H" x3="-2.479969" y3="1.277567" z3="-0.874979" />
<atom id="a14" elementType="C" x3="-1.337288" y3="-0.318944" z3="0.000000" />
<atom id="a15" elementType="H" x3="-1.391246" y3="-0.974259" z3="0.874771" />
<atom id="a16" elementType="H" x3="-1.391246" y3="-0.974259" z3="-0.874771" />
<atom id="a17" elementType="C" x3="0.000000" y3="0.410091" z3="0.000000" />
<atom id="a18" elementType="H" x3="0.054720" y3="1.065260" z3="-0.875105" />
<atom id="a19" elementType="H" x3="0.054720" y3="1.065260" z3="0.875105" />
<atom id="a20" elementType="C" x3="1.191981" y3="-0.538255" z3="0.000000" />
<atom id="a21" elementType="H" x3="1.145738" y3="-1.192613" z3="0.872486" />
<atom id="a22" elementType="H" x3="1.145738" y3="-1.192613" z3="-0.872486" />
<atom id="a23" elementType="C" x3="2.514951" y3="0.213277" z3="0.000000" />
<atom id="a24" elementType="H" x3="2.602225" y3="0.872065" z3="0.866347" />
<atom id="a25" elementType="H" x3="2.602225" y3="0.872065" z3="-0.866347" />
<atom id="a26" elementType="C" x3="3.688737" y3="-0.712012" z3="0.000000" />
<atom id="a27" elementType="O" x3="3.704783" y3="-1.894432" z3="0.000000" />
<atom id="a28" elementType="O" x3="4.984258" y3="-0.096425" z3="0.000000" />
<atom id="a29" elementType="O" spinMultiplicity="2" x3="4.964716" y3="1.208642" z3="0.000000" />
</atomArray>

```
<bondArray>
  <bond atomRefs2="a4 a1" order="1" />
  <bond atomRefs2="a18 a17" order="1" />
  <bond atomRefs2="a13 a11" order="1" />
  <bond atomRefs2="a10 a8" order="1" />
  <bond atomRefs2="a16 a14" order="1" />
  <bond atomRefs2="a7 a5" order="1" />
  <bond atomRefs2="a22 a20" order="1" />
  <bond atomRefs2="a25 a23" order="1" />
  <bond atomRefs2="a1 a2" order="1" />
  <bond atomRefs2="a1 a5" order="1" />
  <bond atomRefs2="a1 a3" order="1" />
  <bond atomRefs2="a5 a8" order="1" />
  <bond atomRefs2="a5 a6" order="1" />
  <bond atomRefs2="a8 a11" order="1" />
  <bond atomRefs2="a8 a9" order="1" />
  <bond atomRefs2="a11 a14" order="1" />
  <bond atomRefs2="a11 a12" order="1" />
  <bond atomRefs2="a14 a17" order="1" />
  <bond atomRefs2="a14 a15" order="1" />
  <bond atomRefs2="a17 a20" order="1" />
  <bond atomRefs2="a17 a19" order="1" />
  <bond atomRefs2="a20 a23" order="1" />
  <bond atomRefs2="a20 a21" order="1" />
  <bond atomRefs2="a23 a26" order="1" />
  <bond atomRefs2="a23 a24" order="1" />
  <bond atomRefs2="a26 a27" order="2" />
  <bond atomRefs2="a26 a28" order="1" />
  <bond atomRefs2="a28 a29" order="1" />
</bondArray>
<propertyList>
  <property title="program">
    <scalar>Gaussian 16, Revision C.01</scalar>
  </property>
  <property title="basis">
    <scalar>Aug-CC-pVTZ (5D, 7F)</scalar>
  </property>
  <property title="File Format">
    <scalar>g03</scalar>
  </property>
  <property title="Energy" dictRef="me:ZPE">
    <scalar units="kcal/mol" >0.0</scalar>
  </property>
  <property title="SpinMultiplicity" dictRef="me:spinMultiplicity">
    <scalar>2.00</scalar>
  </property>
```

<property title="Vibrational Frequencies" dictRef="me:vibFreqs">
<array units="cm-1">22.19 35.08 46.64 75.45 94.60 121.50 124.23 141.64 151.63 166.23 189.18 226.54 244.88 343.52 357.55 468.74 485.13 529.91 563.96 611.89 732.27 736.44 758.90 808.41 826.54 878.69 911.74 958.90 1014.70 1037.69 1038.43 1056.32 1072.17 1089.09 1090.61 1096.86 1143.54 1155.87 1221.03 1246.84 1253.99 1261.41 1298.53 1303.92 1335.24 1336.81 1343.88 1350.60 1352.71 1384.31 1413.17 1420.55 1425.68 1430.03 1452.49 1491.68 1492.45 1495.97 1499.42 1505.51 1506.43 1513.58 1518.16 1921.68 3019.44 3021.81 3023.30 3030.94 3036.40 3042.01 3042.95 3049.31 3058.75 3059.43 3073.13 3073.27 3083.23 3096.15 3111.98 3115.13 3119.36</array>
</property>
<property title="Rotational Constants" dictRef="me:rotConsts">
<array units="cm-1">0.128 0.006 0.006</array>
</property>
<property title="Symmetry Number" dictRef="me:symmetryNumber">
<scalar>1</scalar>
</property>
<property dictRef="me:sigma" >
<scalar>8.02</scalar>
</property>
<property dictRef="me:epsilon" >
<scalar>362.0</scalar>
</property>
<property dictRef="me:frequenciesScaleFactor" >
<scalar>1</scalar>
</property>
</propertyList>
<me:DOSCMethod name="ClassicalRotors" />
<me:DistributionCalcMethod name="Boltzmann" />
<me:energyTransferModel name="ExponentialDown" />
<me:deltaEDown >225.0</me:deltaEDown>
</molecule>
<molecule id="Nonanal-14-rad" spinMultiplicity="2">
<atomArray>
<atom id="a1" elementType="C" x3="2.149851" y3="-0.508511" z3="0.203786" />
<atom id="a2" elementType="O" x3="1.466395" y3="-1.058323" z3="1.046817" />
<atom id="a3" elementType="C" spinMultiplicity="2" x3="2.256700" y3="0.899428" z3="-0.052566" />
<atom id="a4" elementType="O" x3="2.932221" y3="-1.226374" z3="-0.638866" />
<atom id="a5" elementType="O" x3="2.815784" y3="-2.606479" z3="-0.380312" />
<atom id="a6" elementType="H" x3="2.900179" y3="1.211701" z3="-0.864479" />
<atom id="a7" elementType="H" x3="2.178171" y3="-2.602228" z3="0.364103" />
<atom id="a8" elementType="C" x3="1.471963" y3="1.896374" z3="0.705979" />
<atom id="a9" elementType="H" x3="0.990094" y3="1.413316" z3="1.554344" />
<atom id="a10" elementType="H" x3="2.155012" y3="2.653435" z3="1.102707" />
<atom id="a11" elementType="C" x3="0.426513" y3="2.611398" z3="-0.173708" />
<atom id="a12" elementType="H" x3="-0.079164" y3="3.356075" z3="0.445094" />
<atom id="a13" elementType="H" x3="0.945876" y3="3.160432" z3="-0.962469" />

<atom id="a14" elementType="C" x3="-0.605646" y3="1.678307" z3="-0.801680" />
<atom id="a15" elementType="H" x3="-1.250508" y3="2.268221" z3="-1.458029" />
<atom id="a16" elementType="H" x3="-0.097914" y3="0.955366" z3="-1.448614" />
<atom id="a17" elementType="C" x3="-1.470577" y3="0.927367" z3="0.204301" />
<atom id="a18" elementType="H" x3="-0.843103" y3="0.295986" z3="0.837571" />
<atom id="a19" elementType="H" x3="-1.959057" y3="1.648286" z3="0.868406" />
<atom id="a20" elementType="C" x3="-2.527240" y3="0.054866" z3="-0.460644" />
<atom id="a21" elementType="H" x3="-3.172142" y3="0.676131" z3="-1.090791" />
<atom id="a22" elementType="H" x3="-2.035279" y3="-0.654497" z3="-1.133903" />
<atom id="a23" elementType="C" x3="-3.385764" y3="-0.714862" z3="0.535293" />
<atom id="a24" elementType="H" x3="-2.737675" y3="-1.334140" z3="1.160872" />
<atom id="a25" elementType="H" x3="-3.875519" y3="-0.006783" z3="1.209597" />
<atom id="a26" elementType="C" x3="-4.435463" y3="-1.589211" z3="-0.137511" />
<atom id="a27" elementType="H" x3="-5.113258" y3="-0.988285" z3="-0.746622" />
<atom id="a28" elementType="H" x3="-3.967606" y3="-2.325403" z3="-0.793550" />
<atom id="a29" elementType="H" x3="-5.035335" y3="-2.129140" z3="0.595143" />
</atomArray>
<bondArray>
<bond atomRefs2="a15 a14" order="1" />
<bond atomRefs2="a16 a14" order="1" />
<bond atomRefs2="a22 a20" order="1" />
<bond atomRefs2="a21 a20" order="1" />
<bond atomRefs2="a13 a11" order="1" />
<bond atomRefs2="a6 a3" order="1" />
<bond atomRefs2="a14 a11" order="1" />
<bond atomRefs2="a14 a17" order="1" />
<bond atomRefs2="a28 a26" order="1" />
<bond atomRefs2="a27 a26" order="1" />
<bond atomRefs2="a4 a5" order="1" />
<bond atomRefs2="a4 a1" order="1" />
<bond atomRefs2="a20 a17" order="1" />
<bond atomRefs2="a20 a23" order="1" />
<bond atomRefs2="a5 a7" order="1" />
<bond atomRefs2="a11 a12" order="1" />
<bond atomRefs2="a11 a8" order="1" />
<bond atomRefs2="a26 a23" order="1" />
<bond atomRefs2="a26 a29" order="1" />
<bond atomRefs2="a3 a1" order="1" />
<bond atomRefs2="a3 a8" order="1" />
<bond atomRefs2="a1 a2" order="2" />
<bond atomRefs2="a17 a18" order="1" />
<bond atomRefs2="a17 a19" order="1" />
<bond atomRefs2="a23 a24" order="1" />
<bond atomRefs2="a23 a25" order="1" />
<bond atomRefs2="a8 a10" order="1" />
<bond atomRefs2="a8 a9" order="1" />

</bondArray>
<propertyList>
<property title="program">
<scalar>Gaussian 16, Revision C.01</scalar>
</property>
<property title="basis">
<scalar>Aug-CC-pVTZ (5D, 7F)</scalar>
</property>
<property title="File Format">
<scalar>g03</scalar>
</property>
<property title="Energy" dictRef="me:ZPE">
<scalar units="kcal/mol">-2.32</scalar>
</property>
<property title="SpinMultiplicity" dictRef="me:spinMultiplicity">
<scalar>2.00</scalar>
</property>
<property title="Vibrational Frequencies" dictRef="me:vibFreqs">
<array units="cm-1">22.76 29.39 62.18 63.82 96.25 113.91 144.32 166.45 185.65 242.27 251.06 257.63 334.41 367.38 384.08 417.44 468.23 500.90 512.30 621.67 711.52 738.26 757.18 769.21 811.44 852.15 892.00 913.16 927.55 985.50 999.76 1018.02 1047.29 1070.81 1088.26 1091.41 1118.32 1130.89 1159.82 1189.50 1227.47 1240.98 1269.35 1289.50 1317.79 1342.67 1344.53 1349.33 1367.62 1389.72 1408.81 1420.61 1427.03 1463.02 1488.69 1492.64 1495.23 1498.86 1503.33 1505.51 1508.98 1517.03 1532.85 1746.85 3017.85 3021.27 3030.17 3035.68 3036.10 3041.01 3046.73 3048.41 3064.81 3073.47 3086.14 3092.31 3110.82 3119.23 3125.26 3205.14 3539.70</array>
</property>
<property title="Rotational Constants" dictRef="me:rotConsts">
<array units="cm-1">0.034 0.015 0.011</array>
</property>
<property title="Symmetry Number" dictRef="me:symmetryNumber">
<scalar>1</scalar>
</property>
</propertyList>
</molecule>
<molecule id="14Hshift-TS" spinMultiplicity="2">
<atomArray>
<atom id="a1" elementType="C" x3="2.142869" y3="1.092467" z3="0.221033" />
<atom id="a2" elementType="O" x3="1.410308" y3="2.019079" z3="0.357640" />
<atom id="a3" elementType="C" x3="2.151458" y3="-0.194025" z3="1.007530" />
<atom id="a4" elementType="O" x3="3.113500" y3="1.073355" z3="-0.725351" />
<atom id="a5" elementType="O" spinMultiplicity="2" x3="3.782422" y3="-0.162944" z3="-0.669778" />
<atom id="a6" elementType="H" x3="2.583545" y3="-0.061740" z3="1.997396" />
<atom id="a7" elementType="H" x3="3.087475" y3="-0.656826" z3="0.278679" />
<atom id="a8" elementType="C" x3="0.892279" y3="-1.012763" z3="0.962848" />
<atom id="a9" elementType="H" x3="1.036847" y3="-1.924626" z3="1.545639" />

<atom id="a10" elementType="H" x3="0.121000" y3="-0.435359" z3="1.484630" />
<atom id="a11" elementType="C" x3="0.427710" y3="-1.348049" z3="-0.451399" />
<atom id="a12" elementType="H" x3="0.302196" y3="-0.422372" z3="-1.018183" />
<atom id="a13" elementType="H" x3="1.213389" y3="-1.910788" z3="-0.959693" />
<atom id="a14" elementType="C" x3="-0.870700" y3="-2.151498" z3="-0.489951" />
<atom id="a15" elementType="H" x3="-1.117775" y3="-2.348262" z3="-1.535909" />
<atom id="a16" elementType="H" x3="-0.701717" y3="-3.127418" z3="-0.026628" />
<atom id="a17" elementType="C" x3="-2.067838" y3="-1.480793" z3="0.184395" />
<atom id="a18" elementType="H" x3="-2.941847" y3="-2.123394" z3="0.051037" />
<atom id="a19" elementType="H" x3="-1.906801" y3="-1.424808" z3="1.265251" />
<atom id="a20" elementType="C" x3="-2.387821" y3="-0.087899" z3="-0.345927" />
<atom id="a21" elementType="H" x3="-1.553046" y3="0.591243" z3="-0.148685" />
<atom id="a22" elementType="H" x3="-2.493362" y3="-0.130183" z3="-1.435110" />
<atom id="a23" elementType="C" x3="-3.653139" y3="0.505197" z3="0.261689" />
<atom id="a24" elementType="H" x3="-4.497985" y3="-0.156758" z3="0.052356" />
<atom id="a25" elementType="H" x3="-3.548602" y3="0.531296" z3="1.350067" />
<atom id="a26" elementType="C" x3="-3.957344" y3="1.905004" z3="-0.254912" />
<atom id="a27" elementType="H" x3="-4.864614" y3="2.308279" z3="0.194856" />
<atom id="a28" elementType="H" x3="-3.138330" y3="2.590040" z3="-0.030024" />
<atom id="a29" elementType="H" x3="-4.095064" y3="1.899898" z3="-1.337598" />
</atomArray>
<bondArray>
<bond atomRefs2="a15 a14" order="1" />
<bond atomRefs2="a22 a20" order="1" />
<bond atomRefs2="a29 a26" order="1" />
<bond atomRefs2="a12 a11" order="1" />
<bond atomRefs2="a13 a11" order="1" />
<bond atomRefs2="a4 a5" order="1" />
<bond atomRefs2="a4 a1" order="1" />
<bond atomRefs2="a14 a11" order="1" />
<bond atomRefs2="a14 a16" order="1" />
<bond atomRefs2="a14 a17" order="1" />
<bond atomRefs2="a11 a8" order="1" />
<bond atomRefs2="a20 a21" order="1" />
<bond atomRefs2="a20 a17" order="1" />
<bond atomRefs2="a20 a23" order="1" />
<bond atomRefs2="a26 a28" order="1" />
<bond atomRefs2="a26 a27" order="1" />
<bond atomRefs2="a26 a23" order="1" />
<bond atomRefs2="a18 a17" order="1" />
<bond atomRefs2="a24 a23" order="1" />
<bond atomRefs2="a17 a19" order="1" />
<bond atomRefs2="a1 a2" order="2" />
<bond atomRefs2="a1 a3" order="1" />
<bond atomRefs2="a23 a25" order="1" />
<bond atomRefs2="a7 a3" order="1" />

<bond atomRefs2="a8 a3" order="1" />
<bond atomRefs2="a8 a10" order="1" />
<bond atomRefs2="a8 a9" order="1" />
<bond atomRefs2="a3 a6" order="1" />
</bondArray>
<propertyList>
<property title="program">
<scalar>Gaussian 16, Revision C.01</scalar>
</property>
<property title="basis">
<scalar>Aug-CC-pVTZ (5D, 7F)</scalar>
</property>
<property title="File Format">
<scalar>g03</scalar>
</property>
<property title="Energy" dictRef="me:ZPE">
<scalar units="kcal/mol" >26.86</scalar>
</property>
<property title="SpinMultiplicity" dictRef="me:spinMultiplicity">
<scalar>2.00</scalar>
</property>
<property title="Vibrational Frequencies" dictRef="me:vibFreqs">
<array units="cm-1">15.45 34.01 55.34 78.55 89.60 130.36 158.86 183.53 210.05 219.36 249.22 263.52 328.24 406.27 431.08 505.92 524.40 567.66 661.06 689.12 729.63 750.70 780.18 827.84 842.30 872.71 918.89 922.33 966.33 1007.76 1016.59 1032.72 1067.94 1078.10 1086.51 1119.19 1138.39 1152.25 1170.85 1184.14 1212.12 1242.26 1285.39 1301.49 1322.92 1325.50 1345.78 1350.48 1354.19 1385.97 1401.22 1419.96 1422.55 1427.15 1467.23 1492.21 1495.96 1500.98 1505.58 1506.80 1509.53 1518.49 1766.07 1911.25 3021.66 3027.00 3031.24 3035.16 3040.03 3042.81 3048.27 3052.86 3065.51 3075.45 3079.31 3086.38 3098.40 3113.23 3120.66 3134.99</array>
</property>
<property title="ImaginaryFrequency" dictRef="me:imFreqs">
<scalar units="cm-1">1628.11</scalar>
</property>
<property title="Rotational Constants" dictRef="me:rotConsts">
<array units="cm-1">0.045 0.014 0.012</array>
</property>
<property title="Symmetry Number" dictRef="me:symmetryNumber">
<scalar>1</scalar>
</property>
<property dictRef="me:frequenciesScaleFactor" >
<scalar>1</scalar>
</property>
</propertyList>
<me:DOSCMethod name="ClassicalRotors" />
</molecule>
</moleculeList>

```
<reactionList>
  <reaction id="R1">
    <reactantList>
      <reactant>
        <molecule ref="Nonanal-AcylOO-rad" role="modelled" />
      </reactant>
    </reactantList>
    <productList>
      <product>
        <molecule ref="Nonanal-14-rad" role="sink" />
      </product>
    </productList>
    <me:transitionState>
      <molecule ref="14Hshift-TS" role="transitionState" />
    </me:transitionState>
    <me:MCRCMethod name="RRKM" />
          <me:tunneling name="Eckart" />
  </reaction>
  <reaction id="R2">
    <reactantList>
      <reactant>
        <molecule ref="Nonanal-AcylOO-rad" role="modelled" />
      </reactant>
    </reactantList>
    <productList>
      <product>
        <molecule ref="Nonanal-15-rad" role="sink" />
      </product>
    </productList>
    <me:transitionState>
      <molecule ref="15Hshift-TS" role="transitionState" />
    </me:transitionState>
    <me:MCRCMethod name="RRKM" />
          <me:tunneling name="Eckart" />
  </reaction>
        <reaction id="R3">
    <reactantList>
      <reactant>
        <molecule ref="Nonanal-AcylOO-rad" role="modelled" />
      </reactant>
    </reactantList>
    <productList>
      <product>
        <molecule ref="Nonanal-16-rad" role="sink" />
      </product>
    </productList>
```

```
  <me:transitionState>
    <molecule ref="16Hshift-TS" role="transitionState" />
  </me:transitionState>
  <me:MCRCMethod name="RRKM" />
        <me:tunneling name="Eckart" />
</reaction>
<reaction id="R4">
  <reactantList>
    <reactant>
      <molecule ref="Nonanal-AcylOO-rad" role="modelled" />
    </reactant>
  </reactantList>
  <productList>
    <product>
      <molecule ref="Nonanal-17-rad" role="sink" />
    </product>
  </productList>
  <me:transitionState>
    <molecule ref="17Hshift-TS" role="transitionState" />
  </me:transitionState>
  <me:MCRCMethod name="RRKM" />
        <me:tunneling name="Eckart" />
</reaction>
        <reaction id="R5">
  <reactantList>
    <reactant>
      <molecule ref="Nonanal-AcylOO-rad" role="modelled" />
    </reactant>
  </reactantList>
  <productList>
    <product>
      <molecule ref="Nonanal-18-rad" role="sink" />
    </product>
  </productList>
  <me:transitionState>
    <molecule ref="18Hshift-TS" role="transitionState" />
  </me:transitionState>
  <me:MCRCMethod name="RRKM" />
        <me:tunneling name="Eckart" />
</reaction>
<reaction id="R6">
  <reactantList>
    <reactant>
      <molecule ref="Nonanal-AcylOO-rad" role="modelled" />
    </reactant>
  </reactantList>
```

```
    <productList>
      <product>
        <molecule ref="Nonanal-19-rad" role="sink" />
      </product>
    </productList>
    <me:transitionState>
      <molecule ref="19Hshift-TS" role="transitionState" />
    </me:transitionState>
    <me:MCRCMethod name="RRKM" />
          <me:tunneling name="Eckart" />
  </reaction>
</reactionList>
<me:conditions>
  <me:bathGas>N2</me:bathGas>
  <me:PTs>
    <me:PTpair units="Torr" P="760." T="298." precision="qd"  bathGas="N2" />
  </me:PTs>
</me:conditions>
<me:modelParameters>
  <!--Specify grain size directly...-->
  <me:grainSize units="cm-1">100</me:grainSize>
  <!--...or by the total number of grains
      <me:numberOfGrains> 500 </me:numberOfGrains>-->
  <!--Specify increased energy range
      <me:maxTemperature>6000</me:maxTemperature>-->
  <me:energyAboveTheTopHill>25.0</me:energyAboveTheTopHill>
</me:modelParameters>
<me:control>
  <me:printSpeciesProfile />
  <!--<me:testMicroRates />-->
  <!--<me:testRateConstant />
  <me:printGrainDOS />
  <me:printCellDOS />
        <me:testDOS />-->
  <!--<me:printReactionOperatorColumnSums />-->
  <!--<me:printTunnellingCoefficients />
  <me:printGrainkfE />-->
  <!--<me:printGrainBoltzmann />
  <me:printGrainkbE />-->
  <me:eigenvalues>3</me:eigenvalues>
  <!-- <me:hideInactive/>  Molecules and reactions with attribute active="false" are not shown
  <me:diagramEnergyOffset>0</me:diagramEnergyOffset>-->
  <!--Adjusts displayed energies to this values for the lowest species. -->
  <me:calcMethod  name="simpleCalc" />
  <me:ForceMacroDetailedBalance >true</me:ForceMacroDetailedBalance>
</me:control>
```

<metadataList xmlns:dc="http://purl.org/dc/elements/1.1/">
<dc:title>Project name</dc:title>
<dc:source>Acylperoxyrad-Nonanal.xml</dc:source>
<dc:creator>Mesmer v6.0</dc:creator>
<dc:date>20220515_210950</dc:date>
<dc:contributor>ndprse</dc:contributor>
</metadataList>
</me:mesmer>
